# Mechanism‐Guided Precision Hydrolysis of Early Transition Metals to Access (Mixed‐Metal) Oxo Clusters

**DOI:** 10.1002/anie.202525769

**Published:** 2026-02-24

**Authors:** Muhammed Jibin Parammal, Jikson Pulparayil Mathew, Alessandro Prescimone, Ajmal Roshan Unniram Parambil, Harry Wilson, Jonathan De Roo

**Affiliations:** ^1^ Department of Chemistry University of Basel Basel Switzerland

**Keywords:** hafnium, heterometallic, metal oxo clusters, niobium, precision hydrolysis, tantalum, zirconium

## Abstract

Zirconium and hafnium oxo clusters are catalysts, and building blocks for MOFs, 3D‐printing and polymer composites. However, their synthesis is often a matter of trial and error, and new structures are hard to design retrosynthetically. Here, we use our recent insight in the formation mechanism of ${\mathrm{Zr}}_{6}O_{4}$(OH)
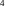
(OOCR)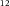 oxo clusters to redesign their synthesis with higher reaction rates, more economical precursors, more sustainable solvents, and higher atom economy, all at room temperature at gram scale. We add exactly 1.33 equivalents of water for the precision hydrolysis of the metals toward **Zr6**‐carboxylate or **Hf6**‐carboxylate oxo clusters (carboxylate  =  acetate, oleate, and 2‐methylbutanoate). Furthermore, we use precision hydrolysis to synthesize the elusive bimetallic Zr/Hf oxo clusters and to expand rational oxo cluster synthesis to the group 5, accessing the new ${\mathrm{Nb}}_{8}O_{12}$(OEt)
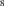
(OBz)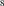 and ${\mathrm{Ta}}_{8}O_{12}$(OEt)
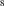
(OBz)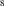 clusters (OBz = benzoate). Precision hydrolysis allows for a rational design and an economic production of oxo clusters on a multigram scale, with potential to become the workhorse for cluster synthesis.

## Graphical abstract

Precision hydrolysis enables the rational, room‐temperature synthesis of Zr and Hf oxo clusters with improved atom economy, sustainable solvents, and gram‐scale yields. By adding exactly 1.33 equivalents of water, Zr_6_, Hf_6_, and elusive bimetallic clusters are accessed, alongside new group 5 oxo clusters, providing a versatile and economical platform for advanced cluster design and materials applications.

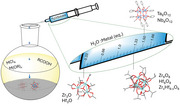

## 1 Introduction

Metal oxo clusters consist of an inorganic core containing several metal atoms, held together with bridging oxo, hydroxo and/or alkoxo moieties. They mark the transition from the molecular regime of coordination complexes to the nanoscale domain of nanoparticles [1, 2]. Indeed, oxo clusters come in different core sizes, ranging from ${\mathrm{Zr}}_{3}O$ complexes to ${\mathrm{Ce}}_{100}O_{167}$ nanoparticles [3]. One can distinguish water soluble polyoxometallates with a negatively charged core and positive counterions [4, 5], from oxo clusters with a cationic core, coordinated by anionic ligands (e.g., carboxylates or phosphinates) which provides solubility in organic solvents [6, 7, 8, 9]. The cationic oxo clusters are typical building blocks of MOFs [10], and as discrete clusters, they have been used in catalysis [11, 12, 13, 14, 15, 16], inorganic–organic hybrid materials [17, 18], and 3D‐printing [19]. However, there is still a limited understanding of their synthetic design [7].

The most prevalent cationic oxo cluster type is $M_{6}O_{8}^{n+}$, which is found for 23 metals [6]. For most metals, hydrolysis is the main synthesis route [6]. Early on, Sanchez et al. have shown that the hydrolysis of Ce($O^{i}\mathrm{Pr}$)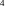 in isopropanol along with acetylacetone (Hacac) led to the formation of ${\mathrm{Ce}}_{6}O_{4}$(OH)
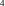
(acac)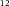 clusters [20]. Hydrolysis has been widely employed in the synthesis of lanthanide and actinide based clusters and also in MOFs, where oxo clusters act as nodes [21, 22, 23]. The group 4 and 5 metal alkoxides and salts are highly sensitive to hydrolysis and can easily react to form amorphous, bulk oxide [24]. Nevertheless, by the precise and controlled addition of water, titanium isopropoxide was hydrolyzed in the presence of carboxylate ligands, yielding ${[\mathrm{Ti}}_{6}O_{6}$(OR)
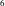
(piv)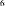] (piv = pivalate) clusters under mild conditions [25]. Titanium oxo clusters with lower nuclearity [26, 27], and zinc oxo clusters [28, 29, 30] have also been reported using the same approach.

The preferred synthesis route for $M_{6}O_{4}$(OH)
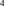
(OOCR)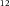 (M = Zr, Hf) has been the reaction of metal alkoxide with carboxylic acid, which generates water in situ through esterification [1, 31]. As a major drawback of this strategy, the water production rate is limited by the reactivity of the carboxylic acid and the alkoxide [32]. Steric hindrance severely affects the esterification rate and even for the least hindered reaction of zirconium propoxide with acetic acid, the reaction takes about 8 h at 25 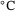 to complete [32]. The esterification rate is also metal‐dependent (e.g., hafnium being slower than zirconium), complicating the synthetic design of mixed metal oxo clusters. Furthermore, it is unclear how group 5 metal alkoxides react. Reports on ${\mathrm{Nb}}_{8}O_{12}$(OEt)
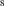
(OOCR)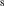 and ${\mathrm{Ta}}_{8}O_{12}$(OEt)
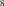
(OOCR)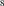 clusters are rare and usually obtained from high temperature reactions or very long reaction times [33, 34].

$M_{6}O_{4}$(OH)
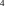
(OOCR)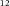 clusters have also been synthesized from ${\mathrm{ZrCl}}_{4}[$ 35, 36, 37, 38, 39], ${\mathrm{ZrOCl}}_{2}$ [40, 41], or ${\mathrm{HfOCl}}_{2}[$ 42] in dimethylformamide (DMF), at elevated temperatures with reaction times often exceeding 24 h. In some cases, the cluster is partially capped by chloride instead of only carboxylate. Jerozal et al. has synthesized ${\mathrm{Zr}}_{6}O_{4}$(OH)
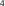
(piv)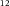 and ${\mathrm{Hf}}_{6}O_{4}$(OH)
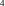
(piv)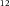 clusters from ${\mathrm{ZrCl}}_{4}$ and ${\mathrm{HfCl}}_{4}$, respectively, at 120 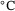 in DMF, with 3 equivalents of water (with respect to the metal) [43]. While the synthesis is highly reproducible in our hands, the pivalate capped clusters are insoluble in several organic solvents, even at elevated temperatures. Our attempts to synthesize clusters with different ligands using this method was not successful. There is thus a need for a versatile synthesis of $M_{6}O_{4}$(OH)
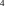
(OOCR)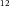 clusters, which takes place under mild conditions, in benign solvents.

Here, we leverage our recent insights in the reaction mechanism of $M_{6}O_{4}$(OH)
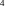
(OOCR)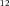 clusters, which require only 4/3 equivalents of water per metal to form [32]. We thus design a method of precision hydrolysis, providing the exact amount of water necessary to hydrolyze the metal alkoxide in the presence of carboxylic acids, forming $M_{6}O_{4}$(OH)
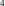
(OOCR)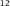 (M = Zr, Hf; OOCR = 2‐methylbutanoate, oleate, acetate). These clusters are denoted as **Zr6**‐methylbutanoate, or **Zr12**‐oleate and **Zr12**‐acetate, since the latter two can form dimers of **Zr6**. We also show how ${\mathrm{HfCl}}_{4}$ and ${\mathrm{ZrCl}}_{4}$ can be used to synthesize clusters under mild reaction conditions. To prove the broader applicability of this method, we demonstrate the synthesis of niobium and tantalum oxo clusters and mixed metal ${\mathrm{Zr}}_{3}{\mathrm{Hf}}_{3}$ oxo clusters.

## 2 Results and Discussion

**FIGURE 1:**
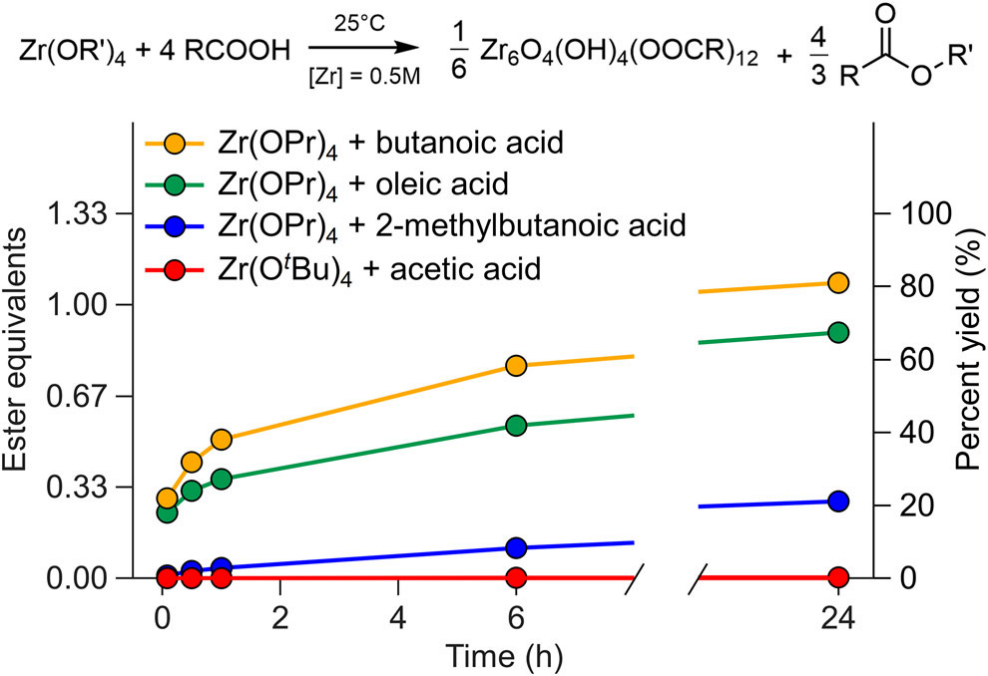
Kinetics of ester formation (determined by NMR spectroscopy) by reacting zirconium alkoxide with carboxylic acids in dichloromethane. The alcohol by‐product of the reaction is not shown in the chemical equation.

In situ esterification from zirconium propoxide and acetic acid is a convenient method to synthesize the **Zr12**‐acetate cluster but the reaction proceeds more sluggishly for other carboxylic acids. Figure 1 shows the build‐up of ester in solution, which is an indirect measure of the kinetics of cluster formation [32]. The kinetics become slower as the length of carbon chain increases from butanoic acid to oleic acid. The reaction of zirconium propoxide with oleic acid only produced 0.9 equivalents of ester after 24 h, while 1.33 equivalents would correspond to full yield. The reaction with 2‐methylbutanoic acid is even slower, producing only 0.3 equivalents after 24 h. Zirconium *tert*‐butoxide does not significantly react with acetic acid. These four results clearly point out the influence of sterics on the esterification kinetics. Such varying kinetics are hampering the effective synthesis of oxo clusters.

**SCHEME 1:**
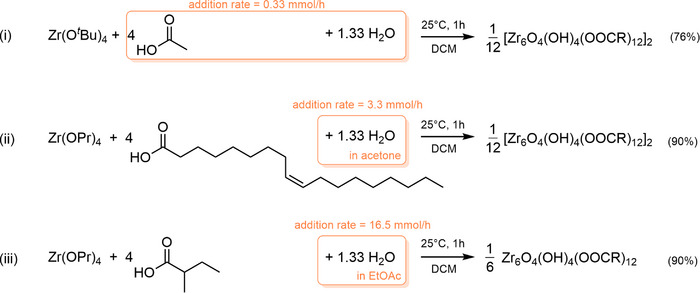
Synthesis of zirconium oxo clusters from zirconium alkoxides via precision hydrolysis. The isolated yield is indicated between brackets.

To decouple cluster formation from the esterification kinetics, we decided to add water as a reagent. To control the mixing, we slowly add the necessary 1.33 equivalents of water dissolved in a liquid via a syringe pump. Given that zirconium *tert*‐butoxide does not form any ester in situ, we took it as a starting point to validate the method, see reaction (i) in Scheme 1. Water was mixed with acetic acid and added over the course of 1 h (at a rate of 0.33 mmol water per hour) to a solution of zirconium *tert*‐butoxide in DCM. The **Zr12**‐acetate cluster is insoluble in DCM and is isolated by centrifugation in good yield (76%) and its characterization by nuclear magnetic resonance (NMR), fouriertransform infrared spectroscopy (FTIR), and pair distribution function (PDF) analysis matches the previous reports where it was synthesized by esterification (Figure S7).

**FIGURE 2:**
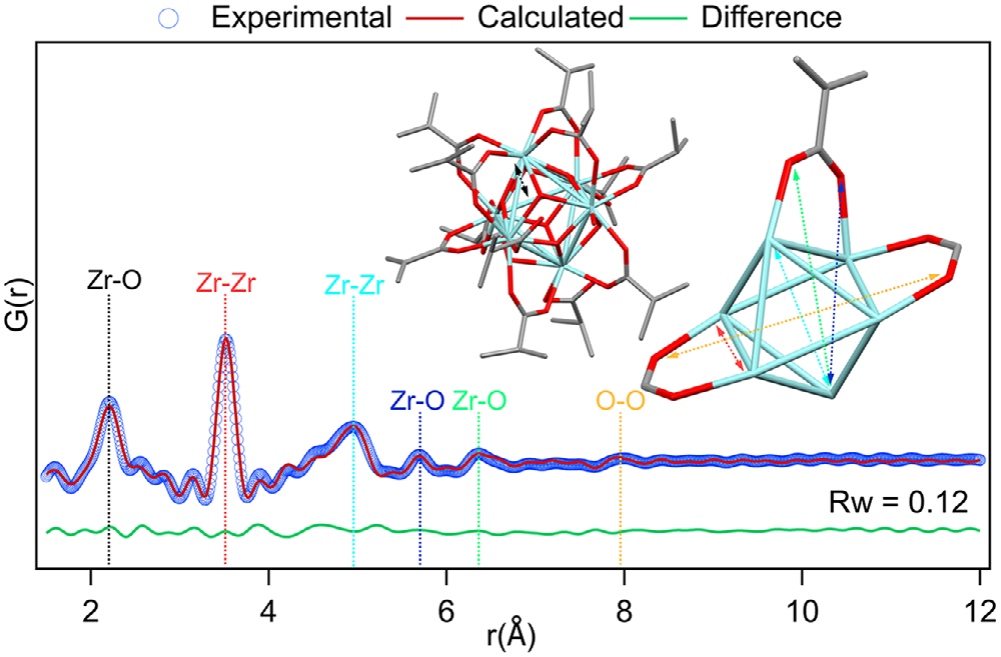
PDF analysis of **Zr6**‐methylbutanoate synthesized from ${\mathrm{ZrCl}}_{4}$. The displayed structure is the result of single‐crystal XRD (X‐ray diffraction) and was used as input model for the PDF refinement. Note that the methyl group, furthest away from the carbonyl, is omitted due to significant disorder and was also not included in the PDF refinement.

**FIGURE 3:**
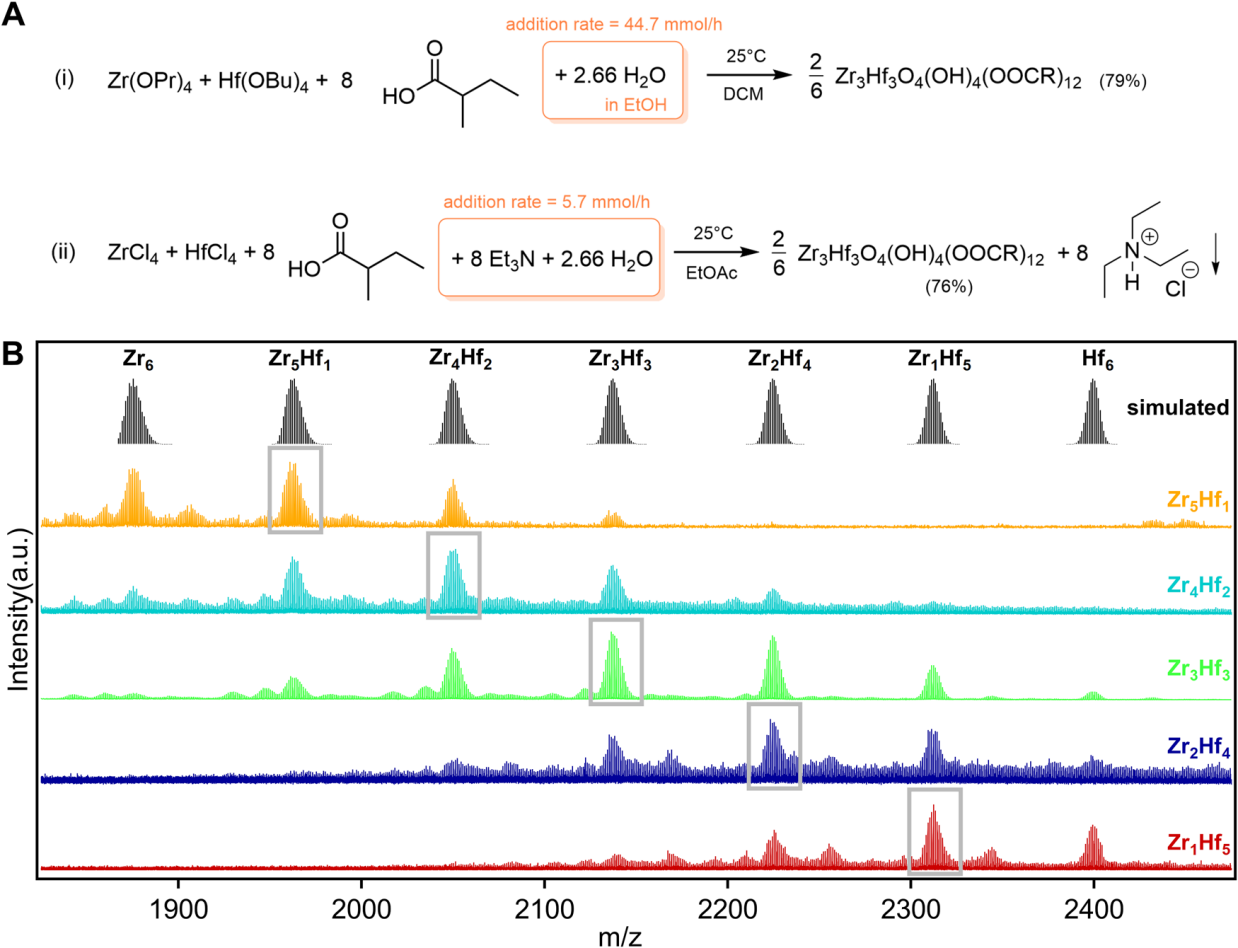
(A) Synthesis of mixed Zr/Hf oxo clusters from alkoxide or chloride precursors via precision hydrolysis. The isolated yield is indicated between brackets. The product that spontaneously precipitates from solution is indicated with a downward arrow. (B) HRMS study of mixed clusters of different Zr:Hf target ratios. The simulated patterns at the top corresponds to ${[\mathrm{Zr}}_{x}{\mathrm{Hf}}_{\left(6-x\right)}O_{4}$(OH)
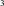
($C_{4}H_{9}\mathrm{COO}$)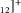 fragments, that is, one OH‐ removed from the discrete cluster. Grey boxes indicate the target cluster in the measured HRMS spectrum.

**FIGURE 4:**
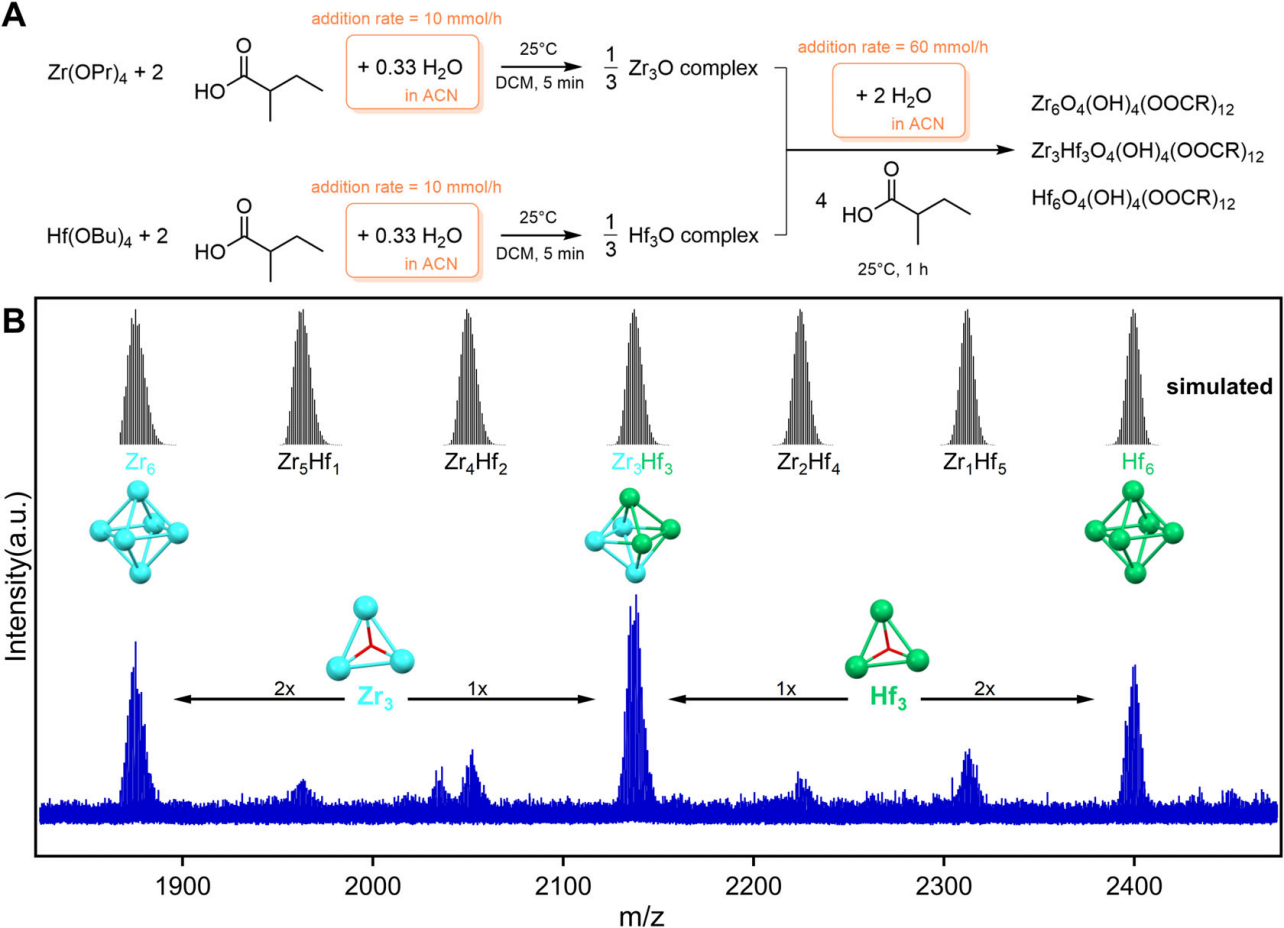
(A) Synthesis of mixed Zr/Hf oxo clusters from alkoxide precursors via precision hydrolysis through homometallic trimeric intermediates. (B) HRMS spectrum of mixed clusters synthesized by combining trimers to form hexamers (blue spectrum). The simulated patterns at the top corresponds to ${[\mathrm{Zr}}_{x}{\mathrm{Hf}}_{\left(6-x\right)}O_{4}$(OH)
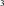
($C_{4}H_{9}\mathrm{COO}$)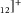 fragments, that is, one ${\mathrm{OH}}^{-}$ removed from the discrete cluster.

**FIGURE 5:**
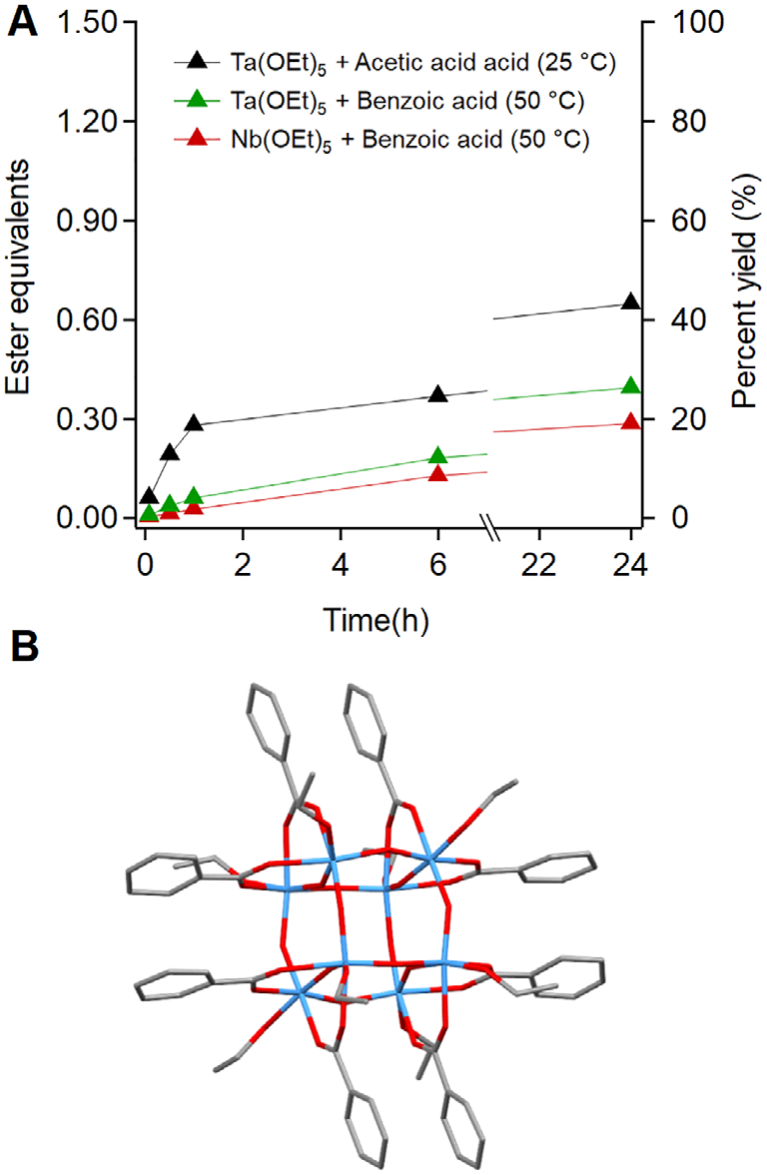
(A) Esterification kinetics (determined by NMR spectroscopy) of tantalum ethoxide and niobium ethoxide precursors with different carboxylic acids at different temperatures, [M] = 0.5 M and [acid] = 2 M. (B) Crystal structure of cluster synthesized using controlled water addition method: ${\mathrm{Ta}}_{8}O_{12}$($C_{6}H_{5}\mathrm{COO}$)
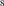
(OEt)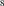 (CCDC deposition number 2312389).

Encouraged by these results, we sought to significantly speed up the synthesis of clusters with carboxylate ligands that provide slow esterification kinetics, for example, oleic acid and 2‐methylbutanoic acid. Given the poor miscibility of water and the aliphatic carboxylic acids, we dissolved water in a dry organic solvent (acetone or ethyl acetate) and added it to a mixture of zirconium propoxide and carboxylic acid in DCM, see Scheme 1. In case of oleic acid, we increased the water addition rate to 3.3 mmol/h and in case of 2‐methylbutanoate, the addition rate was further increased to 16.5 mmol/h (which corresponds on our reaction scale to an addition time of 5 min). Both **Zr12**‐oleate and **Zr6**‐methylbutanoate were soluble in DCM, but were purified and isolated with a high yield via precipitation with acetonitrile, see Scheme 1. The characterization matches the previous reports (Figures S8 and S9). The synthesis and purification of **Zr6**‐methylbutanoate is thus completed in 2 h, compared to 48 h in previous reports [44]. In addition, this method does not require a large excess of carboxylic acid, as is usual in the in situ esterification strategy. Hence, less waste is generated.

**SCHEME 2:**
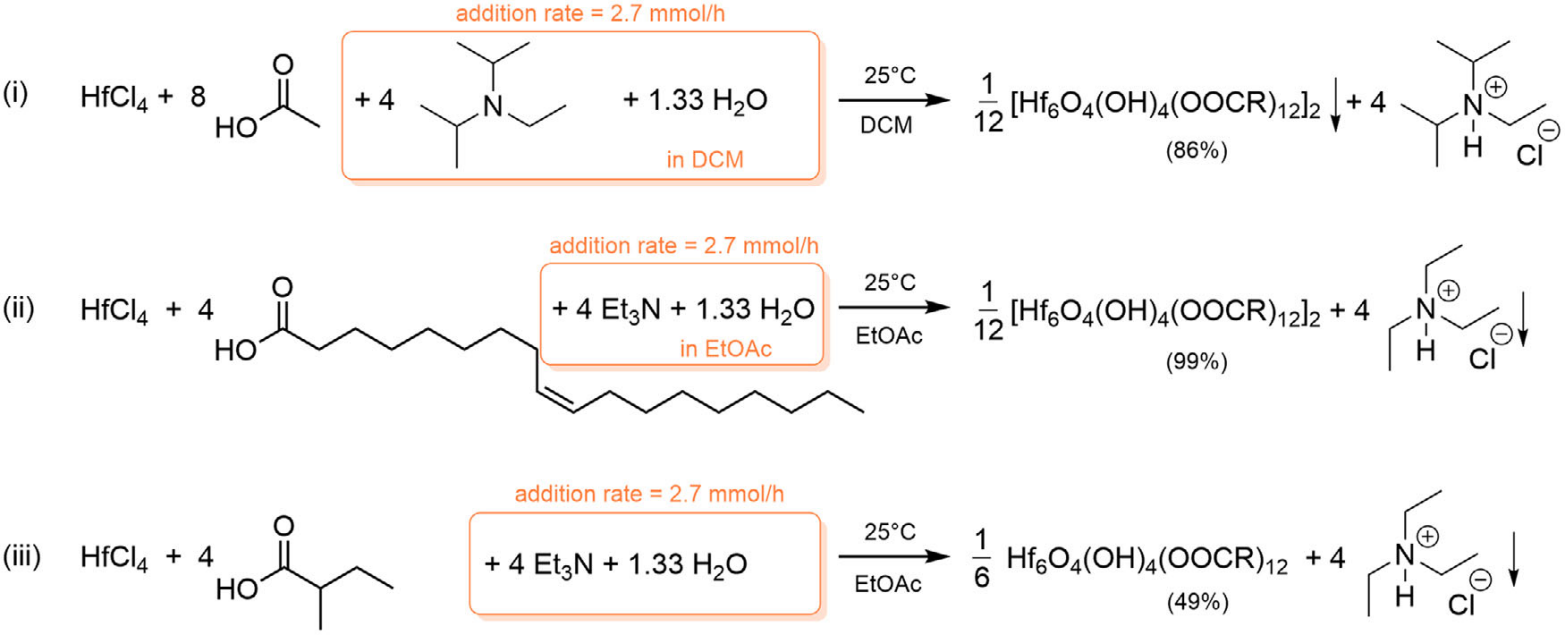
Synthesis of hafnium oxo clusters from hafnium chloride via precision hydrolysis. The isolated yield is indicated between brackets. The product that spontaneously precipitates from solution is indicated with a downward arrow. The same reaction scheme can be used to make zirconium oxo clusters from zirconium chloride.

Zirconium propoxide is the most economical zirconium source (at the time of writing; 20% cheaper per mole compared to ${\mathrm{ZrCl}}_{4}$). However, the reverse is true for hafnium; Hf(OBu)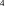 is 10 times more expensive than ${\mathrm{HfCl}}_{4}$. This makes ${\mathrm{HfCl}}_{4}$ an attractive choice for cost‐efficient synthesis. Simply mixing ${\mathrm{HfCl}}_{4}$ with carboxylic acid and water, would result in the formation of HCl, which opposes the reaction progress. Four equivalents of organic base are thus added (mixed with the 1.33 water equivalents) to neutralize the released acid, see Scheme 2. As such, we synthesized **Hf12**‐acetate clusters which precipitate from DCM, while we keep the ammonium chloride salt soluble with Hunnig's base (to facilitate purification of the oxo cluster). In case of **Hf12**‐oleate and **Hf6**‐methylbutanoate, we use ethylacetate as a much more sustainable alternative to DCM and triethylamine as base. Triethylammonium chloride precipitates at the end of the reaction, while the oxo cluster stays soluble, again facilitating cluster purification. The same strategy can be used to make the zirconium oxo clusters from ${\mathrm{ZrCl}}_{4}$. The reaction yields are moderate to high and the final products are characterized by NMR, FTIR, PDF and thermogravimetric analysis (TGA) (see Figures S7–S9, S11). We keep the water addition rates modest (2.7 mmol/h) due to the formation of precipitates during the reaction, and thus carefully avoid a high local concentration of water. The scalability of our method was demonstrated by synthesizing more than 12 g of **Hf12**‐acetate clusters in a single synthesis (see Figure S1).

**Zr6**‐methylbutanoate and **Hf6**‐methylbutanoate were previously synthesized [44], but their exact structures were not solved. Here, we crystallized **Zr6**‐methylbutanoate and **Hf6**‐methylbutanoate by slow evaporation and obtained two quasi‐identical crystal structures, with the metal being the only obvious difference, see Figures S2–S5. The clusters synthesized from the chloride and alkoxide precursors gave quasi‐identical crystals, with some disorder in the methyl group furthest away from the carbonyl, due to the flexibility of the butyl chain. The structure without this terminal methyl group is shown in Figure 2. Based on prior literature, the particular FTIR pattern of the asymmetric carboxylate stretch suggests the occurrence of both chelating and bridging carboxylate ligands [44]. Indeed, the single crystal structure contains three chelating carboxylates on one face of the octahedron. The coordination is completed by eight bridging and one monodentate carboxylate, and one water molecule, bringing the molecular formula to $M_{6}O_{4}$(OH)
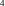
(OOCR)
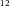
($H_{2}O$). There are also two hydrogen bonded carboxylic acids in the crystal structure. The oxo clusters were also characterized by x‐ray total scattering and PDF analysis, as this is a bulk technique and it is not prone to crystal selection bias. Using the single‐crystal structure as input (without the terminal methyl group), we obtain excellent refinements of the PDFs, see Figure 2 for **Zr6**‐methylbutanoate. The peak at 2.1–2.3 Å corresponds to Zr–O distances, peaks at 3.4–3.8 and 5 Å corresponds to Zr–Zr distances. The distances beyond 5 Å are assigned to various Zr–O and O–O distances.

Having successfully synthesized monometallic oxo clusters, we turned our attention to mixed Zr/Hf oxo clusters. The latter have not been reported in the typical hexanuclear, octahedral geometry [6]. ${\mathrm{Zr}}^{4+}$ and ${\mathrm{Hf}}^{4+}$ have nearly identical ionic radii (0.84 and 0.83 Å, respectively). In the homometallic 2‐methylbutyrate‐capped clusters, the *cis* metal–metal distances are 3.50(3) Å for Zr and 3.48(3) Å for Hf, while the *trans* metal–metal distances are 4.96(2) and 4.93(2) Å, respectively. These very small differences suggest that incorporating both metals into a single cluster should not induce significant structural distortion. We, therefore, first focus on clusters with a target Zr:Hf ratio of 3:3, and with 2‐methylbutanoate ligands, denoted as **Zr3Hf3**‐methylbutanoate. Identical products are obtained from the alkoxide or the chloride precursors (Figure 3A). The NMR and FTIR spectra of **Zr3Hf3** clusters (Figure S12A,B) are highly similar to the homometallic **Zr6** and **Hf6** clusters, suggesting that the surface chemistry remains the same. TGA analysis (Figure S13) delivers an inorganic content of 41.4 % for the **Zr3Hf3** clusters. The theoretical value for the ${\mathrm{Zr}}_{3}{\mathrm{Hf}}_{3}O_{4}$(OH)
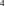
($C_{4}H_{9}\mathrm{COO}$)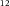 cluster is 46.5 %, while for the **Zr6** and **Hf6** it is 34.7 % and 52.3 %, respectively. Our observed value lies in the middle of these two limits, which is a strong indication of the presence of both metals in the bulk sample in a nearly equal ratio. ICP‐MS analysis further confirms the 3:3 ratio of Zr:Hf, see Table 1. The slightly lower than expected inorganic content in TGA is due to the presence of hydrogen‐bonded carboxylic acid, as also indicated by the FTIR spectrum (Figure S12A), which features a carboxylic acid stretch at 1715 ${\mathrm{cm}}^{-1}$. The amount of excess carboxylic acid is also calculated from the quantitative ICP‐MS analysis (see Supporting Information for details) and we find about three molecules of 2‐methylbutanoic acid per **M6** unit.

**TABLE 1:** Average Zr and Hf composition ($n_{\mathrm{Zr}}$, $n_{\mathrm{Hf}}$) and number of extra 2‐methylbutanoic acid molecules per **M6** cluster for the mixed metal clusters, calculated from the ICP‐MS data, see Supporting Information for the detailed calculations.

Cluster	$n_{\mathrm{Zr}}$	$n_{\mathrm{Hf}}$	Extra acid per cluster
${\mathrm{Zr}}_{5}{\mathrm{Hf}}_{1}$	4.97	1.03	2.44
${\mathrm{Zr}}_{4}{\mathrm{Hf}}_{2}$	4.00	2.00	3.43
${\mathrm{Zr}}_{3}{\mathrm{Hf}}_{3}$	2.93	3.07	3.10
${\mathrm{Zr}}_{2}{\mathrm{Hf}}_{4}$	1.97	4.03	2.93
${\mathrm{Zr}}_{1}{\mathrm{Hf}}_{5}$	1.04	4.96	3.04

*Note*: The first column indicates the target ratio.

The **Zr3Hf3** cluster was also analyzed using PDF (see an extensive discussion in the Supporting Information), where we observed the characteristic peaks of $M_{6}O_{8}$ octahedra (Figure S15). We generated a series of structural models based on the **Zr6**‐methylbutanoate structure (Figure 2) incorporating both Zr and Hf in different stoichiometries and atomic arrangements. The best fit with the lowest Rw (0.15), is obtained for a ${\mathrm{Zr}}_{3}{\mathrm{Hf}}_{3}$ configuration (Figure S15). The different ${\mathrm{Zr}}_{3}{\mathrm{Hf}}_{3}$ configurations span an Rw range of 0.15–0.17 (Figure S16). While the difference between the extremes is appreciable, the Rw difference between several of the best models is trivial. In addition, an excellent fit (Rw = 0.16) was obtained for a dual phase model with an equimolar mixture of **Zr6** and **Hf6** clusters (Figure S18). PDF analysis thus identifies the average composition, but is not the best technique to unambiguously assign the clusters as ${\mathrm{Zr}}_{3}{\mathrm{Hf}}_{3}$.

The most conclusive evidence for discrete **Zr3Hf3** clusters was obtained from high resolution mass spectrometry (HRMS, see Figure 3B). For the nominal ${\mathrm{Zr}}_{3}{\mathrm{Hf}}_{3}$ sample, we detected fragments of ${\mathrm{Zr}}_{3}{\mathrm{Hf}}_{3}$ clusters with matching exact mass and isotopic patterns along with the mixed clusters of other ratios, albeit with lower intensities. The cluster intensities follow an almost gaussian distribution, centered around the nominal ${\mathrm{Zr}}_{3}{\mathrm{Hf}}_{3}$ stoichiometry. This is not surprising given the chemical similarity between Zr and Hf, which allows them to mix stochastically. We also prepared clusters with other Zr:Hf ratios by varying the ratio of ${\mathrm{ZrCl}}_{4}$ and ${\mathrm{HfCl}}_{4}$ in the reaction mixture. The NMR and FTIR of these clusters were also similar to the homometallic clusters. It is clear from the TGA (Figure S14) and ICP‐MS analysis (see Table 1) that the average metal ratio is preserved in the bulk sample. HRMS again shows a distribution of cluster stoichiometries, centered around the average.

To break free from the stochastic cluster stoichiometry, we use the insights from our work on the mechanism of oxo cluster formation [32]. First, we use precision hydrolysis to form homometallic trinuclear intermediates (**Zr3** and **Hf3**) by adding 0.33 equivalents of water to a mixture of M(OR)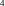 and 2 equivalents of 2‐methylbutanoic acid. Second, these trinuclear intermediates are mixed together and the additional 1 equivalent of water and 2 equivalents of 2‐methylbutanoic acid are added to form **M6** clusters (see Figure 4A). Theoretically, this procedure should selectively produce three species: **Zr6**, **Zr3Hf3**, and **Hf6**. The products were characterized by HRMS (Figure 4B), and indeed, the three expected species are dominant in the mass spectrum. This is a highly exciting result as it deviates from the randomness of usual mixed metal clusters formation.

**SCHEME 3:**
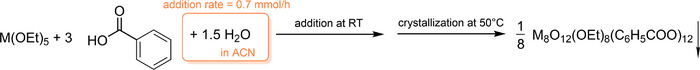
Synthesis of Nb or Ta (denoted as M) oxo clusters from their corresponding ethoxide precursors via precision hydrolysis. The isolated yield is indicated between brackets. The final product that crystallizes out is indicated with a downward arrow.

Finally, we explored the synthesis of group 5 oxo clusters via precision hydrolysis (Scheme 3). Reports on ${\mathrm{Nb}}_{8}O_{12}$(OEt)
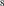
(OOCR)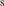 and ${\mathrm{Ta}}_{8}O_{12}$(OEt)
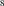
(OOCR)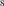 clusters are rare and usually obtained from high temperature reactions or very long reaction times [33, 34]. This is understood by monitoring the room temperature esterification kinetics of Ta(OEt)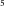 with acetic acid, which should be a sterically unhindered reaction. However, we observe a very slow conversion into ester (Figure 5A). With benzoic acid, the kinetics are slower, even at an elevated temperature of 50 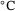, rendering the esterification strategy undesired for oxo cluster formation. Using precision hydrolysis, we synthesized the novel benzoate capped **Nb8** and **Ta8** clusters from Nb(OEt)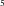 and Ta(OEt)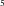, respectively. We added water at room temperature under stirring, which forms a waxy precipitate. The latter dissolves at 50 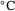 and crystallizes into single crystals over the course of 2 h. The Nb and Ta ethoxide precursors are extremely sensitive to hydrolysis and therefore slow, dropwise addition of a precise amount of water is key to the success of this synthesis. Even a small excess of water resulted in the formation of insoluble amorphous product.

The structure of the resulting clusters are shown in Figures 5B and S6. The core of the clusters can be formulated as $M_{8}O_{12}$ (M = Nb or Ta). The 8 metal atoms form the vertices of a cube and the 12 O atoms occupy edges of the cube. Eight benzoates bind in bridging mode, forming a plane. The remaining ethoxides bind exclusively in monodentate mode, protruding out diagonally from each vertex of the cube. Using a higher acid ratio (9 eq.) resulted in two additional OEt groups being replaced by two monodentally binding benzoates (see Figure S6B). The FTIR spectrum of both Nb and Ta clusters were found to be similar (see Figure S19) as expected from their crystal structures and the inorganic content was verified by TGA analysis (see Figure S20).

## 3 Conclusion

We demonstrated a fast, economical, and scalable approach for synthesizing metal oxo clusters through controlled water addition. This approach allows for the synthesis of Zr and Hf oxo clusters from both alkoxide and chloride precursors under mild conditions, even when using bulky ligands. The approach does not use the toxic solvent DMF and has a high atom economy. Beyond homometallic clusters, controlled water addition also enabled the synthesis of mixed‐metal ${\mathrm{Zr}}_{x}{\mathrm{Hf}}_{6-x}$ oxo clusters, which were previously unreported. By using the insights of the reaction mechanism, we could obtain a non‐stochastic mixture of mixed cluster stoichometries. Finally, we successfully synthesized Nb and Ta oxo‐clusters using controlled hydrolysis, under unprecedentedly mild conditions. These findings provide a workshorse synthesis method for existing oxo clusters and a broadly applicable strategy for the design of novel metal oxo clusters.

## 4 Experimental Section

### 4.1 General Characterization

NMR spectra were recorded at 298.15 K on a Bruker UltraShield 500 spectrometer operating at a 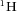 frequency of 500.13 MHz. Regular 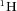 NMR spectra were acquired using the standard pulse sequences (zg30) with a 30 degree pulse and a recycle delay of 1.5 s from the Bruker library. 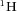 NMR spectra were acquired with 64 scans and post‐processed with a line broadening of 1 Hz. Chemical shifts (δ) are given in parts per million (ppm), and the residual solvent peak was used as an internal standard (${\mathrm{CDCl}}_{3}$: δH = 7.26 ppm). The IR spectra were recorded on a PerklinElmer spectrum 2 ATR‐FTIR with a diamond crystal. The TGA was performed on a TGA5500 (TA instruments) instrument. All TGA measurements were done under air (oxidizing atmosphere). The samples were heated to 900 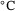 at a ramping rate of 5 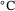/min. At the end, an isotherm of 15 min is given to ensure that all the organics are burned out. Quantitative elemental analysis was performed using Vario MICRO CUBE from Elementar.

### 4.2 Single Crystal XRD

Single crystal data were collected on a STOE STADIVARI diffractometer. Suitable crystals were selected and mounted on a MITIGEN holder in perfluoroether oil. The crystals were kept at 150 K during data collection. Using Olex2 [45], the structure was solved with the SHELXT [46] structure solution program using intrinsic phasing and refined with the SHELXL [47] refinement package using least squares minimisation.

### 4.3 Synchrotron X‐Ray Total Scattering Experiments

Samples were prepared in 1 mm polyamide Kapton tubes and were measured at beamline P21.1 at DESY (Hamburg, Germany) or at the DanMAX beamline (Sweden). At P21.1, data were collected at room temperature in rapid acquisition mode using a Perkin Elmer digital x‐ray flat panel amorphous silicon detector (2048 × 2048 pixels, 200 × 200 μm pixel size, sample‐to‐detector distance 380 mm) with an incident wavelength of λ  =  0.1220 Å, and calibration was performed using a Ni standard. At DanMAX, data were collected at room temperature using a DECTRIS PILATUS3 X CdTe 2M area detector (incident energy 35 keV, Q‐range ∼0.5–19.8 Å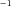) and calibration was done using a Si standard.

### 4.4 Analysis of Synchrotron X‐Ray Total Scattering Data

Raw 2D data were corrected for geometrical effects and polarization, then azimuthally integrated to produce 1D scattering intensities versus the magnitude of the momentum transfer Q (where Q=4πsinθ/λ for elastic scattering) using pyFAI and xpdtools [48, 49]. The program xPDFsuite with PDFgetX3 was used to perform the background subtraction, further corrections, and normalization to obtain the reduced total scattering structure function F(Q), and Fourier transformation to obtain the PDF, G(r) [50, 51]. For data reduction, the following parameters were used after proper background subtraction: Qmin = 0.8 Å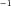, Qmax = 18 to 22 Å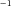, and Rpoly = 0.9–1.7 Å. Modeling and fitting were carried out using Diffpy‐CMI [52]. The Debye scattering equation was used to generate the calculated PDF from discrete structure models. The structure models are supplied as xyz files in the Supporting Information. The refinements were carried out by refining the scale factor, isotropic atomic displacement parameters (Uiso), and delta2 (coefficient for 1/$r^{2}$ contribution to the peak sharpening). The exponentially dampening sine‐wave contribution was calculated according to the following equation.

$$y=A\;e^{-{\left(\frac{r-r_{0}}{2\sigma_{eff}}\right)}^{2}}sin\left(2\pi (\frac{r}{\lambda }-\phi )\right)$$

*A*—Amplitude of oscillation, *r*—the distance in PDF, λ—wavelength, ϕ—phase shift, σ—effective dampening with σ_eff_ = σ/*a* for *r* < *r*_0_ and σ_eff_ = σ×a for *r* > *r*_0_, and *a* is the asymmetry parameter. *r*_0_ is not a physical parameter in real space and is used to describe different dampening behavior [53].

### 4.5 High‐Resolution Mass Spectrometry (HRMS)

HRMS spectra were acquired using a Bruker maXis4G high resolution mass spectrometer equipped with an electronspray ionization source. The cluster was dissolved in a mixture of 1:1 ACN:DCM at a concentration of 160μg/mL. The samples were directly introduced into the instrument at a rate of 30 μL /min using a syringe pump. The *m/z* scan range was from 1000 to 5000 *m/z*. The heated capillary temperature was 180 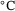 and the capillary voltage was 4.5 kV. The applied collision energy was 10 eV. The samples were analyzed in positive ion mode. DataAnalysis 4.4 from Bruker was used to process the raw data. Simulations were carried out using enviPat [54].

## Conflicts of Interest

The authors declare no conflicts of interest.

## Supporting information


**Supporting File 1**: Deposition numbers 2312388 (for ${\mathrm{Nb}}_{8}O_{12}$(OEt)
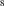
(OBz)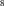), 2312389 (for ${\mathrm{Ta}}_{8}O_{12}$(OEt)
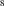
(OBz)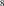), 2453142 (for ${\mathrm{Zr}}_{6}O_{4}$(OH)
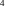
($C_{4}H_{9}\mathrm{COO}$)
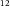
($H_{2}O$)), 2453143 (for ${\mathrm{Hf}}_{6}O_{4}$(OH)
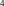
($C_{4}H_{9}\mathrm{COO}$)
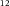
($H_{2}O$)), 2495248 (for ${\mathrm{Zr}}_{6}O_{4}$(OH)
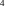
($C_{4}H_{9}\mathrm{COO}$)
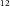
($H_{2}O$)), 2495249 (for ${\mathrm{Nb}}_{8}O_{12}$(OEt)
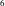
(OBz)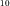) contains the supplementary crystallographic data for this paper. These data are provided free of charge by the Cambridge Crystallographic Data Centre. Supporting information contains additional experimental methods and characterizations.

**Supporting File 2**: anie71298–sup–0002–Data.zip.

## Data Availability

The data that support the findings of this study are openly available in Zenedo at 10.5281/zenodo.17351804, reference number 17351804.
