# Supplementary material for: Mechanism‐Guided Precision Hydrolysis of Early Transition Metals to Access (Mixed‐Metal) Oxo Clusters
Source: Angew Chem Int Ed Engl. 2026 Feb 24;65(15):e25769. doi: 10.1002/anie.202525769 (PMC13053926; doi:10.1002/anie.202525769)
Supplement: Supplementary file 1 — Supporting File 1: Deposition numbers 2312388 (for Nb8O12(OEt)(OBz)), 2312389 (for Ta8O12(OEt)(OBz)), 2453142 (for Zr6O4(OH)(C4H9COO)(H2O)), 2453143 (for Hf6O4(OH)(C4H9COO)(H2O)), 2495248 (for Zr6O4(OH)(C4H9COO)(H2O)), 2495249 (for Nb8O12(OEt)(OBz)) contains the supplementary crystallographic data for this paper. These data are provided free of charge by the Cambridge Crystallographic Data Centre. Supporting information contains additional experimental methods and characterizations. [file ANIE-65-e25769-s001.pdf]

Supporting Information: Mechanism-Guided Precision Hydrolysis of Early Transition Metals to Access (Mixed-Metal) Oxo Clusters

Muhammed Jibin Parammal, Jikson Pulparayil Mathew, Alessandro Prescimone, Ajmal Roshan Unniram Parambil, Harry Wilson, and Jonathan De Roo*

Department of Chemistry, University of Basel, Mattenstrasse 22, 4058 Basel, Switzerland

E-mail: jonathan.deroo@unibas.ch

Contents

1 Materials and methods **S-2**

1.1 Materials S-2

1.2 Synthesis of clusters S-3

1.3 Crystallization of 2-methylbutyrate capped clusters S-14

1.4 Insights about water addition rate S-15

2 Crystal structure of newly crystallized clusters **S-15**

3 Monometallic Zr and Hf oxo clusters **S-19**

3.1 NMR, FTIR and PDF characterization of clusters S-19

3.2 Discussion of FTIR spectrum of synthesized clusters S-22

3.3 TGA of clusters S-23

4 Bimetallic oxo clusters S-24

4.1 NMR, FTIR and TGA analsysis S-24

4.2 PDF analsysis S-26

4.3 Calculation of metal ratios and extra acid per cluster from ICP-MS data . . S-30

5 Refined parameters from PDF fitting S-32

6 Additional characterization of Nb and Ta clusters S-33

References S-35

1 Materials and methods

1.1 Materials

All chemical reagents and solvents were purchased from commercial sources and unless mentioned, used as received without further purification. Zirconium *n*-propoxide (70 w% in 1-propanol), hafnium *n*-butoxide (99%), Acetic acid (>99%), 2-methyl butanoic acid (98%) and benzoic (>99.5%) acid were purchased from Sigma-Aldrich. Acetic acid was dried overnight using $CaSO_4$ and vacuum distilled to remove the water content and any impurities. Benzoic acid was recrystallized in dry acetonitrile and dried under high vacuum before loading to the glovebox. Dry dichloromethane (99.8%), dry acetonitrile (99.9%), dry ethylacetate (99.9%), dry acetone (99.8%), Hunig's base (N-Ethyldiisopropylamine, 99%) and oleic acid (90%) were purchased from Thermo Fisher Scientific. Triethylamine (anhydrous, 99%) was purchased from Fluorochem. Acetone (100%) and acetonitrile (99.9%) were obtained from VWR Chemicals. Hafnium(IV) chloride (99.9%), zirconium(IV) chloride (99.5%), $Nb(OEt)_5$ (>99.9%) and $Ta(OEt)_5$(>99.9%) were purchased form Strem Chemicals. Deuterated chloroform ($CDCl_3$, 99.8%) was received from Eurisotop and treated with 4 Å molecular sieves

(Sigma-Aldrich) for 24 hours before use. Water is used from the in-house distilled water source. Zirconium (IV) tert-butoxide was synthesized according to Dhaene et al.[S1]

1.2 Synthesis of clusters

$Zr_{12}O_8(OH)_8(CH_3COO)_{24} \cdot 6CH_3COOH \cdot 3.5CH_2Cl_2$ from $Zr(OtBu)_4$

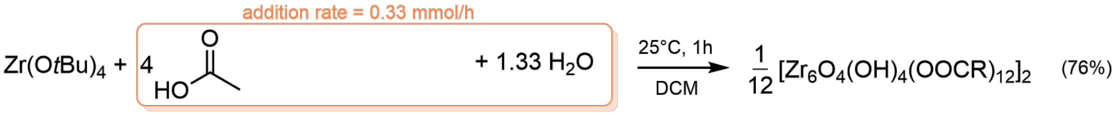

Zirconium tert-butoxide (95.9 mg, 0.25 mmol, 1 eq.) was dissolved in dry DCM (0.33 mL) in a 3 mL vial containing a stirring bar, and the vial was sealed with a septum inside the glovebox to ensure an air and moisture-free environment. The mixture was stirred at room temperature. Separately, a solution was prepared by mixing 5.9 μL (0.33 mmol, 1.33 eq.) of water with 57 μL (1 mmol, 4 eq.) of acetic acid. This solution was loaded into a syringe and injected into the vial containing the zirconium tert-butoxide solution over the course of 1 hour. The reaction mixture was stirred continuously at room temperature to ensure thorough mixing and reaction. After the addition of the water-acetic acid solution, the mixture was stirred for another 1 hour and the precipitated products were recovered by centrifugation. The solution was centrifuged again twice, with 0.5 mL of DCM added during each step, and subsequently dried on high vacuum. The final product was obtained as a white precipitate. The yield of the product was 54 mg (76 %). $Zr_{12}C_{63.5}H_{111}Cl_7O_{76}$, found (calc.) C: 22.70 (22.21); H: 3.68 (3.26). TGA found (calc.): 48.7 (53.3)

$Zr_{12}O_8(OH)_8(oleate)_{24}$ from $Zr(OPr)_4$

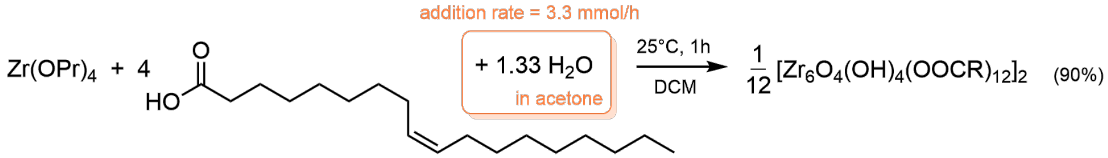

Oleic acid (3530 mg, 10 mmol, 4 eq.) was weighed into a 20 mL vial containing a stirring bar, and the vial was sealed with a septum to maintain an air and moisture-free environment. The sealed vial was then dried under high vacuum for 1 hour to remove any residual moisture. Following this, 2000 μL of dry dichloromethane (DCM) was added to the vial. Zirconium propoxide (1120 μL, 2.5 mmol, 1 eq.) was introduced into the solution immediately before the addition of water. Separately, water (59.85 μL, 3.325 mmol, 1.33 eq.) was combined with 300 μL of acetone, and the resulting solution was added to the vial using a syringe pump over the course of 1 hour, while stirring at room temperature to ensure thorough mixing and reaction. The purification process was carried out after 16 hrs in a 15 mL centrifuge tube. The reaction mixture was concentrated by rotary evaporation, and the residue was dissolved in 1.5 mL of dry DCM. Precipitation was induced by adding 4.5 mL of acetonitrile (ACN), followed by centrifugation and removal of the top layer. The clusters were re-dissolved in 1.5 mL dry DCM and precipitated out using 4.5 mL acetone. This process was again repeated twice. Finally, the product was dried under high vacuum with stirring to remove any residual solvent. The final product was obtained as a slightly yellow viscous liquid. The yield was 1509 mg (90 %). $Zr_{12}C_{432}H_{800}O_{64}$, found (calc.) C: 64.37 (63.95); H: 9.57 (9.94). TGA found (calc.): 19.0 (18.2)

$Zr_6O_4(OH)_4(C_4H_9COO)_{12}(H_2O) \cdot (C_4H_9COO)_3$ from $Zr(OPr)_4$

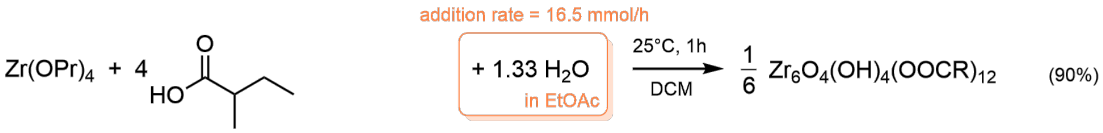

A 20 mL vial was flushed with argon and vacuum to maintain an inert atmosphere. Dry DCM was added first, followed by zirconium propoxide (1120 μL, 2.5 mmol, 1 eq.), and 2-methyl butyric acid (1086 μL, 10 mmol, 4 eq.) was added just before the water. Water (59.4 μL, 3.30 mmol, 1.33 eq.) was then added after being diluted in 1.2 mL of dry ethyl acetate over 5 minutes using a syringe pump, while stirring, ensuring a controlled and steady addition. Purification was performed immediately after the water addition. The DCM/ethyl acetate solvent mixture was first evaporated using a rotary evaporator. The remaining sample was then dissolved in 1 mL of DCM and precipitated out using 3 mL of ACN. To improve yield, the sample was placed in the fridge for 5 minutes. This precipitation process was repeated three times. Finally, the product was dried under high vacuum. The final product was obtained as a white precipitate. The yield of the reaction was 0.712 g (90.3%). The entire procedure from start of the reaction to final dried product was completed in 2 hrs. $Zr_6C_{75}H_{141}O_{39}$, found (calc.) C: 40.51 (40.68); H: 6.44 (6.42). TGA found (calc.): 35.2 (33.4)

$Hf_{12}O_8(OH)_8(CH_3COO)_{24} \cdot 6CH_3COOH \cdot 3.5CH_2Cl_2$ from $HfCl_4$

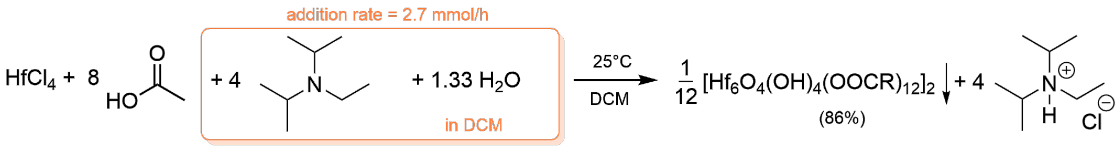

Small scale. Hafnium tetrachloride ($HfCl_4$, 640.58 mg, 2 mmol, 1 eq.) was mixed with dry dichloromethane (DCM, 3 mL) in a sealed vial under an air and moisture-free environment. Acetic acid (920 μL, 16 mmol, 8 eq.) was added directly to the vial. A mixture of water (47.9 μL, 2.66 mmol, 1.33 eq.) and *N,N*-diisopropylethylamine (DIPEA, 1420 μL, 8 mmol, 4 eq.) in dry DCM (0.5 mL) was added slowly over a period of 1 hr using a syringe pump while stirring. The solution was stirred for 16 hours at room temperature. For purification, the mixture was centrifuged thrice with additional dry DCM (1.5 mL added each time except for the first time), and the supernatant was removed. The remaining solid was dried under high vacuum. The final product was obtained as a white precipitate. The final yield was 547.6 mg, corresponding to an 86% yield. $Hf_{12}C_{63.5}H_{111}Cl_7O_{76}$, found (calc.) C: 17.55 (17.02); H: 2.88 (2.50). TGA found (calc.): 67.2 (66.1)

Large scale. A 200 mL round-bottom (RB) flask equipped with an appropriate stir bar was loaded with hafnium tetrachloride ($HfCl_4$, 12.8116 g, 40 mmol, 1.0 eq.) inside a glovebox. After sealing the flask with a septum, it was removed from the glovebox. Dry dichloromethane (DCM, 55 mL) was added, followed by dry acetic acid (18.4 mL, 320 mmol, 8.0 eq.). Separately, a solution of water (958 μL, 53.2 mmol, 1.33 eq.), N,N-diisopropylethylamine (DIPEA, 28.4 mL, 160 mmol, 4.0 eq.), and dry DCM (5 mL) was prepared and added to the reaction mixture in two equal portions using a 20 mL syringe over 1 hour. The temperature of the reaction flask increased during the addition, likely due to acid-base neutralization. White precipitates began to form toward the end of the water addition. The reaction mixture was stirred at room temperature for 16 hours. Purification was performed by centrifugation. The

crude product was washed three times with 10 mL portions of dry DCM, followed by a final wash using a mixture of 9 mL DCM and 6 mL acetic acid . The solution was stirred again using another 40 mL of fresh DCM for 2 hrs, which is finally centrifuged and dried under high vacuum. This final step removes trace amounts of salt remaining in the sample. The isolated product was obtained as a white powder. The experimental yield was 12.13 g (92%)

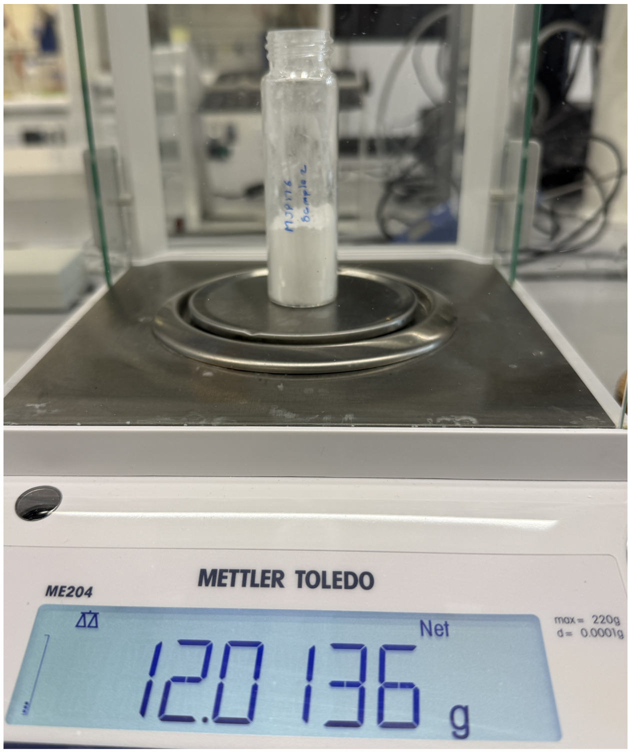

Figure S1: Final product from the scaled up synthesis of **Hf12**-acetate clusters from $HfCl_4$

$Hf_{12}O_8(OH)_8(oleate)_{24}$ from $HfCl_4$

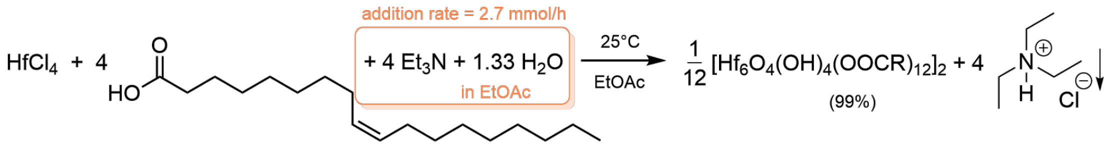

$HfCl_4$ (640.58 mg, 2 mmol, 1 eq.) was taken in a 40 mL vial sealed with a septum to maintain an air and moisture-free environment. Oleic acid (2.8 mL, 8 mmol, 4 eq., 90 %) was added along with dry ethyl acetate (5 mL). Water (47.9 μL, 2.66 mmol, 1.33 eq.), mixed with Et_3N (1091 μL, 8 mmol, 4 eq., density = 0.726 g/mL), was added dropwise using a syringe pump over 1 hour. White fumes were observed upon the addition of the water-Et_3N mixture. The solution was stirred at room temperature for an additional 16 hours after the water addition. To remove the triethylammonium chloride salt, the reaction mixture was centrifuged with an additional 10 mL of dry ethyl acetate. The supernatant was collected, filtered using a syringe filter, and dried first using a rotary evaporator and then under high vacuum to obtain the crude product. Purification was performed by dissolving the crude product in 1 mL of dry DCM and precipitating it using 3 mL of dry ACN and removing the supernatent. The clusters were re-dissolved in 1 mL dry DCM and precipitated out using 3 mL acetone. This process was repeated twice. Finally, the sample was dried under high vacuum with stirring, yielding 1.5116 g (99 %). The final product was obtained as a slightly yellow viscous liquid.$Hf_{12}C_{432}H_{800}O_{64}$, found (calc.) C: 57.94 (56.64); H: 9.04 (8.80). TGA found (calc.): 25.0 (27.6)

$Zr_{12}O_8(OH)_8(oleate)_{24}$ was synthesized by using $ZrCl_4$ (466 mg, 2 mmol, 1 eq.) instead of $HfCl_4$ and final yield was 1.3091 g (97 %). $Zr_{12}C_{432}H_{800}O_{64}$, found (calc.) C: 65.42 (63.95); H: 10.00 (9.94). TGA found (calc.): 17.7 (18.2)

$Hf_6O_4(OH)_4(C_4H_9COO)_{12}(H_2O)\cdot(C_4H_9COO)_3)$ from $HfCl_4$

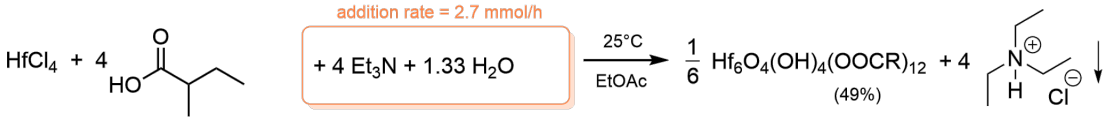

Hafnium tetrachloride ($HfCl_4$, 687 mg, 2.14 mmol, 1 eq.) was taken in a 40 mL vial sealed with a septum to maintain an air and moisture-free environment and sonicated in dry ethyl acetate (7 mL) for 15 minutes. Following this, 2-methyl butyric acid (956 μL, 8.6 mmol, 2 eq.) was added directly to the vial. Water (51.4 μL, 2.85 mmol, 1.33 eq.), mixed with triethylamine (Et_3N, 1166 μL, 8.6 mmol, 4 eq.), was added while stirring, slowly over 1 hour using a syringe pump without any additional solvent. White fumes were observed upon the addition of the water-Et_3N mixture. The solution was stirred overnight at room temperature. For separation, the mixture was centrifuged with an additional 20 mL of dry ethyl acetate, divided into two steps of 10 mL each. The supernatant was collected and filtered using a syringe filter. The filtered solution was dried first using a rotary evaporator, followed by high vacuum drying. Purification was achieved by dissolving the crude product in 0.5 mL of dry DCM, ensuring complete dissolution. The product was then precipitated out by adding 1.5 mL of dry ACN. The resulting white precipitate was collected, and the sample was dried under high vacuum. The final yield was 423 mg (49%). $Hf_6C_{75}H_{141}O_{39}$, found (calc.) C: 32.73 (32.90); H: 5.29 (5.19). TGA found (calc.): 47.9 (46.1)

$Zr_6O_4(OH)_4(C_4H_9COO)_{12}(H_2O)\cdot(C_4H_9COO)_3)$ was synthesized by using $ZrCl_4$ (500 mg, 2.14 mmol, 1 eq.) instead of $HfCl_4$ and final yield was 427.7 mg (62 %). $Zr_6C_{75}H_{141}O_{39}$, found (calc.) C: 41.34 (40.68); H: 6.62 (6.42). TGA found (calc.): 34.3 (33.4)

$Zr_3Hf_3O_4(OH)_4(C_4H_9COO)_{12}(H_2O)\cdot(C_4H_9COO)_3)$ from $ZrCl_4$ and $HfCl_4$

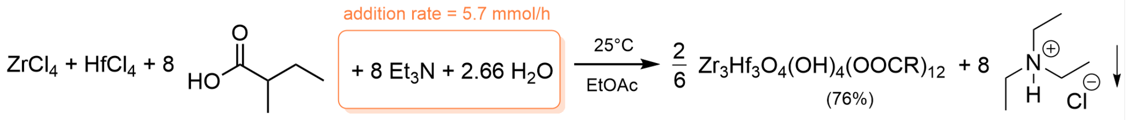

Hafnium and zirconium tetrachloride ($HfCl_4$ and $ZrCl_4$, 686.6 mg and 500.0 mg, respectively, 2.14 mmol each, 1 eq. each) were taken together in a 40 mL vial sealed with a septum to maintain an air and moisture-free environment and sonicated in dry ethyl acetate (14 mL) for 15 minutes. Following this, 2-methyl butanoic acid (1912 μL, 17.12 mmol, 8 eq.) was added directly to the vial. Water (102.4 μL, 5.692 mmol, 2.66 eq.), mixed with triethylamine (Et_3N, 2336 μL, 17.12 mmol, 8 eq.), was added while stirring, slowly over 1 hour using a syringe pump without any additional solvent. White fumes were observed upon the addition of the water-Et_3N mixture. The solution was stirred overnight after the water addition at room temperature. The resulting mixture consists of cluster dissolved in solvent and triethylammonium chloride salt as white precipitate. For separation, the mixture was centrifuged in two steps using a total of 40 mL of dry ethyl acetate, divided into two portions of 20 mL each. The supernatant was collected and filtered using a syringe filter. The filtered solution was dried first using a rotary evaporator, followed by high vacuum drying. Purification was performed by dissolving the crude product in 0.5 mL of dry DCM, ensuring complete dissolution. The product was precipitated out by adding 1.5 mL of dry ACN. This procedure was repeated twice. The purified product was dried under high vacuum. The final product was obtained as a white precipitate. The final yield was 1.1664 g (76%). $Zr_3Hf_3C_{75}H_{141}O_{39}$, found (calc.) C: 35.97 (36.38); H: 5.77 (5.74). TGA found (calc.): 41.4 (40.4). The remaining mixed cluster combinations were synthesized using same procedure by adjusting the ratios of $ZrCl_4$ and $HfCl_4$.

$Zr_3Hf_3O_4(OH)_4(C_4H_9COO)_{12}(H_2O) \cdot (C_4H_9COO)_3)$ from $Zr(OPr)_4$ and $Hf(OnBu)_4$

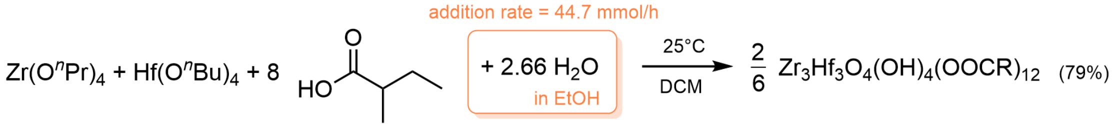

Zirconium propoxide ($Zr(OPr)_4$, 627 μL, 1.4 mmol, 1 eq.), hafnium n-butoxide ($Hf(OBu)_4$, 533 μL, 1.4 mmol, 1 eq.), and a stirring bar were taken in a 40 mL vial inside a glove box and sealed with a septum to maintain an air and moisture-free environment. Dry dichloromethane (DCM, 2700 μL) was added, followed by 2-methylbutyric acid (2-MBA, 1216 μL, 11.2 mmol, 8 eq.). Water (66.5 μL, 2.66 eq.), diluted in 500 μL dry ethanol, was added using syringe pump over 5 minutes while stirring. The reaction was stirred for an additional 10 minutes before purification. For purification, the solvent was removed using rotary evaporation. The product was dissolved in 500 μL DCM and precipitated out using 5 mL ACN, repeated thrice. After each round, the mixture was centrifuged, and the supernatant was discarded. The purified product was transferred using 5 mL toluene and dried under high vacuum overnight. The final product was obtained as a white precipitate. The final yield was 793.5 mg (79%). $Zr_3Hf_3C_{75}H_{141}O_{39}$, found (calc.) C: 36.84 (36.38); H: 5.88 (5.74). TGA found (calc.): 41.4 (40.4)

Zr_3Hf_3-methylbutyrate by mixing of trimers

Hafnium and zirconium alkoxides ($Hf(OBu)_4$ and $Zr(OPr)_4$, 952 μL and 1120 μL, respectively, 2.50 mmol each, 1 eq. each) were used as metal precursors. In two separate 20 mL vials, each compound was combined with 2-methylbutyric acid (543 μL, 5.00 mmol, 2 eq.) and dry dichloromethane (2.88 mL) under an inert atmosphere. A premixed solution of water (14.9 μL, 0.825 mmol, 0.33 eq.) and dry acetonitrile (300 μL) was added dropwise in 5 minutes to each vial. The mixtures were stirred for 5 minutes at room temperature.

The two solutions were then combined into a single vial, and an additional portion of water (90 μL, 5.00 mmol, 2 eq.) in dry acetonitrile (500 μL), and additional 2-methylbutyric acid (1086 μL, 10.00 mmol, 4 eq.) was subsequently added slowly with stirring in 5 minutes. The reaction mixture (total volume $\sim$ 10 mL) was stirred for 1 hour at room temperature. Purification was performed after 1 hour. DCM was first evaporated using a rotary evaporator and then sample was dried under high vacuum for 5 minutes. The remaining sample was then dissolved in 1 mL of DCM and precipitated out using 3 mL of ACN. This precipitation process was repeated three times. Finally, the product was dried under high vacuum and the final yield was found to be 1.2980 g (72.3 %).

$Nb_8O_{12}(OEt)_8(C_6H_5COO)_8 \cdot CH_3CN$ from $Nb(OEt)_5$

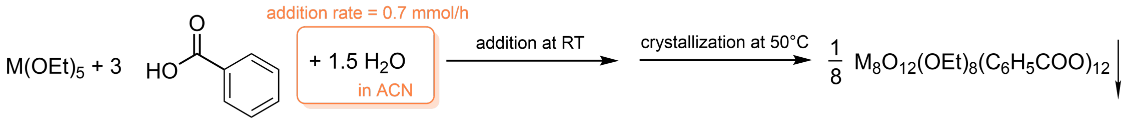

Niobium ethoxide $Nb(OEt)_5$ (125 μL, 0.5 mmol, 1 eq.), benzoic acid (183 mg, 3 eq.), a stirring bar, and 1 mL dry acetonitrile (ACN) were taken in a 4 mL glass vial, capped with a septum to maintain an air and moisture-free environment. The solution was fully clear after the addition of the reagents. Water (12.6 μL, 0.7 mmol, 1.4 eq.) was then added dropwise after being diluted in 300 μL dry ACN using a syringe pump over 1 hour at room temperature, with stirring. The total reaction volume was approximately 1.5 mL. After the addition of water, the vial turned turbid, forming a greyish sticky precipitate, which indicated the formation of an intermediate species. The temperature was then increased to 50°C, and the vials were left undisturbed. Single crystals formed within 2 hours. To improve the yield of crystals, the solution was left overnight, yielding 87 % after isolation and drying. $Nb_8C_{72}H_{80}O_{36}$, found (calc.) C: 38.17 (38.19); H: 3.57 (3.56). TGA found (calc.): 46.2 (46.9). The exact same procedure was followed for tantalum by using $Ta(OEt)_5$ (130 μL, 0.5 mmol, 1 eq.) precursor and benzoic acid (183 mg, 3 eq.). The final products were obtained as transparent crystals with a yield of 71 %. $Ta_8C_{72}H_{80}O_{36}$, found (calc.) C: 29.01 (29.13); H: 2.73 (2.72). TGA found (calc.): 58.7 (59.5)

1.3 Crystallization of 2-methylbutyrate capped clusters

Purified Hf6-methylbutyrate clusters synthesized from $HfCl_4$: To prepare the crystallization solution, dissolve 500 mg of purified Hf_6-MBA clusters in 0.5 mL of dichloromethane (DCM). To this solution, add 0.5 mL of dry toluene and 92 μL of methyl butyrate (corresponding to 4 equivalents per 1 equivalent of cluster). Partially cover the vial containing the solution with a piece of aluminum foil punctured with small holes to allow gradual solvent evaporation. Crystals form within 4 days.

Purified Zr6-methylbutyrate clusters synthesized from $ZrCl_4$ To prepare the crystallization solution, dissolve 390 mg of purified **Zr6**-MBA clusters in 0.4 mL of dichloromethane (DCM). To this solution, add 0.4 mL of dry toluene and 92 μL of methyl butyrate (corresponding to 4 equivalents per 1 equivalent of cluster). Partially cover the vial containing the solution with a piece of aluminum foil punctured with small holes to allow gradual solvent evaporation. Crystals form within 4 days.

Hf6-methylbutyrate using esterification from $Hf(OBu)_4$ A solution of $Hf(OBu)_4$ (5 mmol, 1.902 mL, 1 eq.) and 2-methylbutyric acid (20 mmol, 2.180 mL, 4 eq) was added in a vial along with dry dichloromethane (5.9 mL), giving a total reaction volume of 10 mL ([Hf] = 0.5 M). All additions were carried out under air and moisture-free conditions using glovebox/schlenk techniques. The mixture was stirred for 48 hours at room temperature, after which it was kept open to air along with addition of 3 mL of toluene. Crystallization occurred over the course of one week, yielding single crystals suitable for analysis.

Zr6-methylbutyrate using esterification from $Zr(OPr)_4$ A solution of $Zr(OPr)_4$ (5 mmol, 2.25 mL, 1 eq.) and 2-methylbutyric acid (40 mmol, 4.35 mL, 8 eq) was added in a vial along with dry toluene (5.4 mL), giving a total reaction volume of 10 mL ([Zr] = 0.5 M). All additions were carried out under air and moisture-free conditions using glovebox/schlenk techniques. The mixture was kept at 30 °C for 48 hours without stirring, after which it was

kept open to air. Crystallization occurred over the course of 3 days, yielding single crystals suitable for analysis.

1.4 Insights about water addition rate

The rate at which water is added during the synthesis of clusters has an influence on the outcome of the reaction and needs to be considered carefully. There is no single 'optimal' addition rate that applies universally, as this depends on the specific system and scale of the reaction. The most critical factor is the sensitivity of the metal precursor to hydrolysis. This is particularly important for group 5 alkoxides, such as $Nb(OEt)_5$ and $Ta(OEt)_5$, which are highly reactive and prone to forming amorphous oxides if hydrolysis occurs too rapidly. In systems where both the precursor and the product are fully soluble (e.g., Zr alkoxide with 2-MBA or oleic acid), water can generally be added at a faster rate without adverse effects. When precipitates form during the reaction, slower water addition is recommended to ensure that unreacted dissolved species are thoroughly mixed and react uniformly. Maintaining a low local water concentration helps prevent uncontrolled amorphous oxide formation, making it prudent to add water slowly. Overall, some optimization is required for each system, but in general, slow, dropwise addition provides reproducible formation of discrete clusters across all systems studied here.

2 Crystal structure of newly crystallized clusters

A high degree of disorder is present in structures of clusters with 2-MBA ligands due to the flexible butyl chain. The crystals are weakly diffracting at medium and high resolution, which further complicates structure determination. As a result, some atoms on one or more ligands were not found in the difference map. Finding and placing the missing C atoms proved particularly challenging, given how disordered and crowded the structures are and how flexible the ligands can be. Consequently, hydrogen atoms on these ligands were omitted,

as some carbon sites could not be unambiguously assigned as either CH_2 or CH_3 groups. To obtain a reasonable structure, restraints and constraints were used while modeling. In some cases, the disordered atoms had to be refined isotropically. A lot of SADI, EADP, DFIX and DANG were applied to treat several atoms in the periphery of the clusters. This affects the accuracy of all the bonds and distances in the structures.

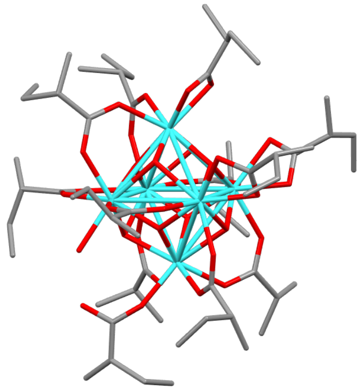

Figure S2: Crystal structure of **Zr6**-methylbutanoate cluster synthesized from $Zr(OPr)_4$ by esterification (CCDC deposition number 2453142).

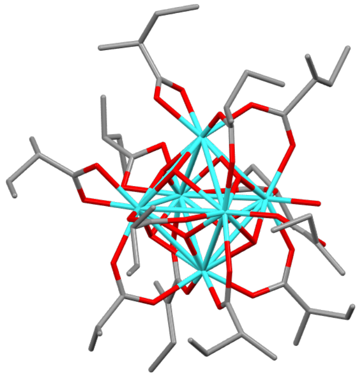

Figure S3: Crystal structure of **Zr6**-methylbutanoate cluster synthesized from $ZrCl_4$ (CCDC deposition number 2495248).

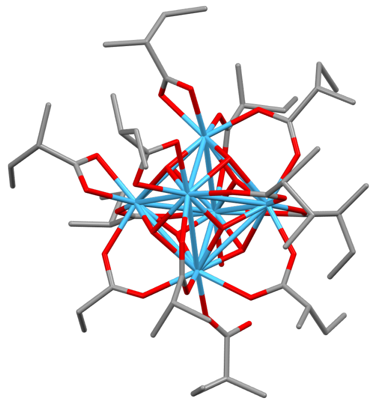

Figure S4: Crystal structure of **Hf6**-methylbutanoate cluster synthesized from $Hf(OBu)_4$ by esterification (CCDC deposition number 2453143).

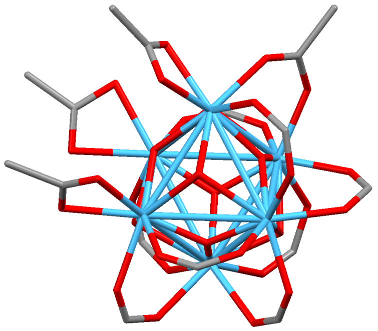

Figure S5: The crystal structure of the **Hf6**-methylbutanoate cluster synthesized from $HfCl_4$. The structure could not be fully solved because the diffraction data revealed substantial disorder and pronounced thermal motion among the peripheral ligands compared to other clusters, compounded by weak high-angle reflections. Despite these challenges, the Hf_6O_8 metal–oxo core and the oxygen atoms directly coordinated to the carboxylate ligands are clearly defined, with their positional and atomic displacement parameters considered reliable. In contrast, the remaining atomic positions could not be unambiguously modeled, as the diffuse electron density made it difficult—if not impossible—to accurately assign all non-core atoms. The available structural model, provided in .xyz format along with other data, depicts the Hf_6O_8 core and its immediate coordination environment, including the binding modes of all twelve carboxylate ligands.

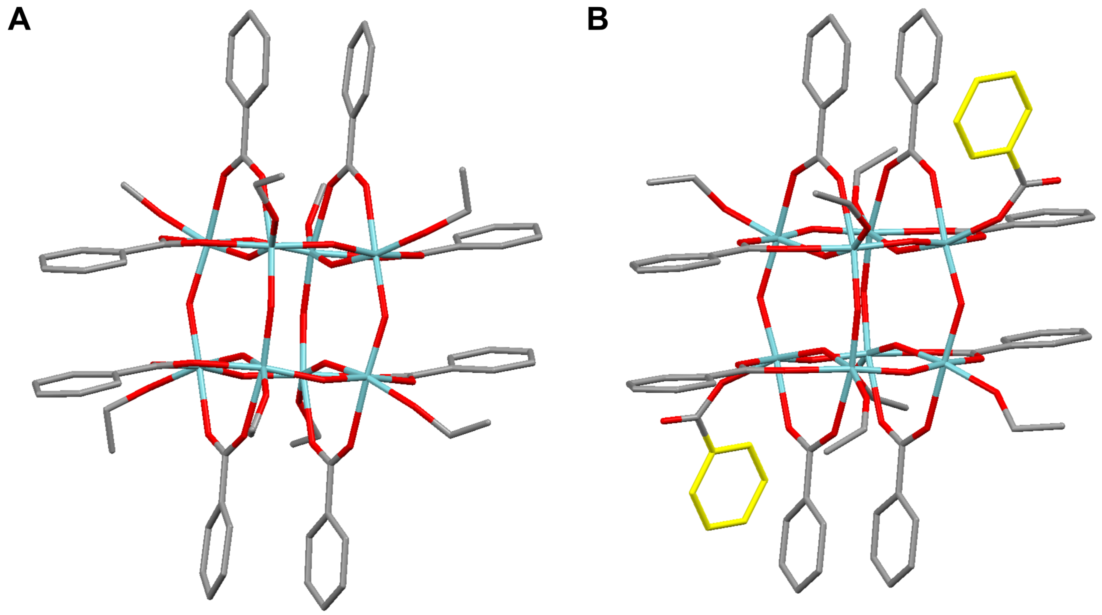

Figure S6: Single-Crystal structures of Nb clusters with formula (A)$Nb_8O_{12}(C_6H_5COO)_8(OEt)_8$ (CCDC deposition number 2312388) and (B) $Nb_8O_{12}(C_6H_5COO)_{10}(OEt)_6$ (CCDC deposition number 2495249). The two additional benzoate groups in B is shown using yellow color.

3 Monometallic Zr and Hf oxo clusters

3.1 NMR, FTIR and PDF characterization of clusters

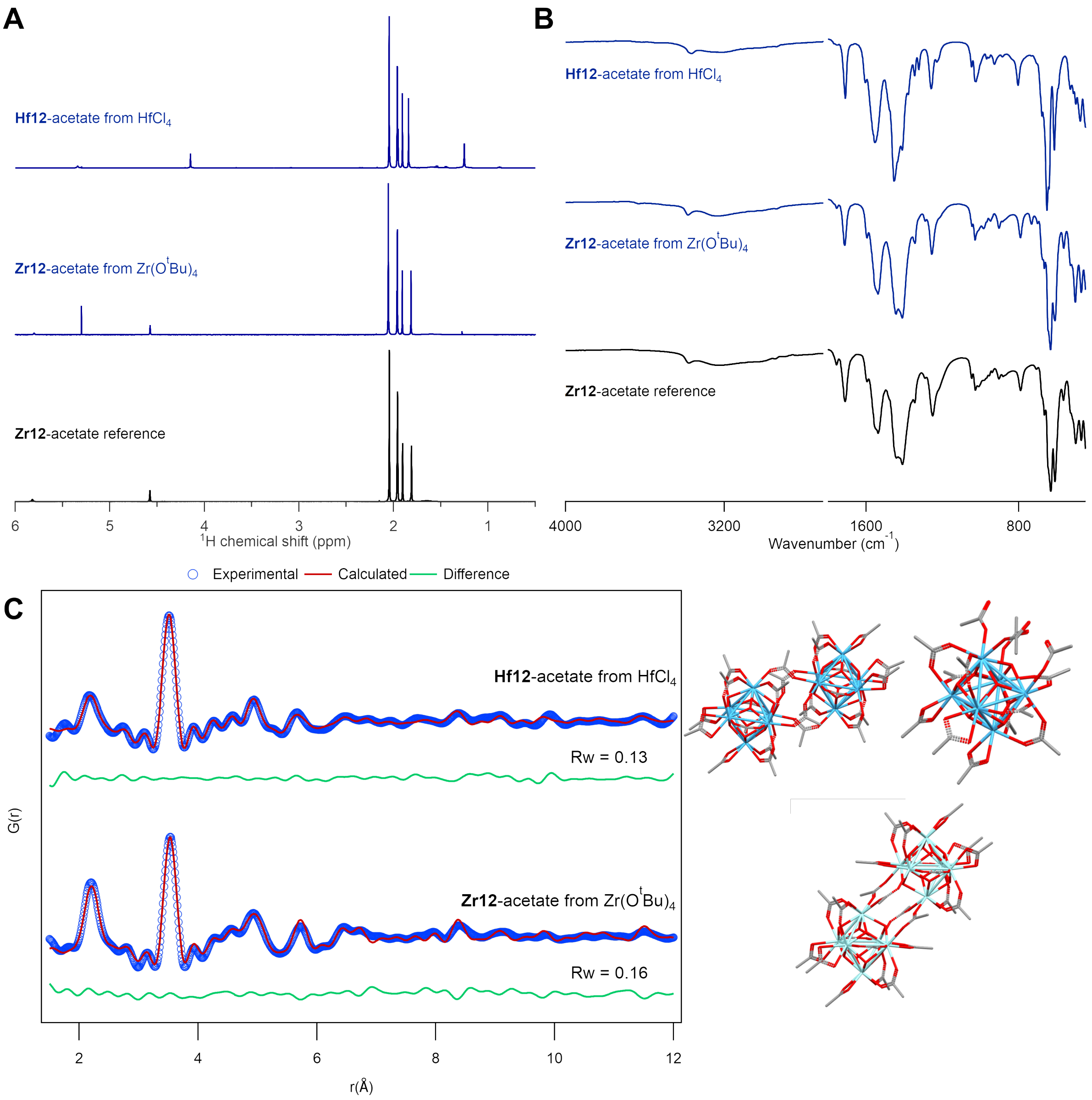

Figure S7: Comparison of (A) NMR spectra, (B) FT-IR spectra and (C)PDF data of **Zr12** and **Hf12**-acetate clusters synthesized from different precursors. The crystal structures used for fitting is shown on the right side of the corresponding PDF fit. Both the structures used for **Hf12**-acetate are derived from structure with CCDC deposition number 604528 and the structure used for **Zr12**-acetate is derived from structure with CCDC number 604529. Two structures indicate that a dual phase fitting method is used for fitting with the structure on the left being the first phase. The reference spectra corresponds to clusters synthesized using esterification method.[S2]

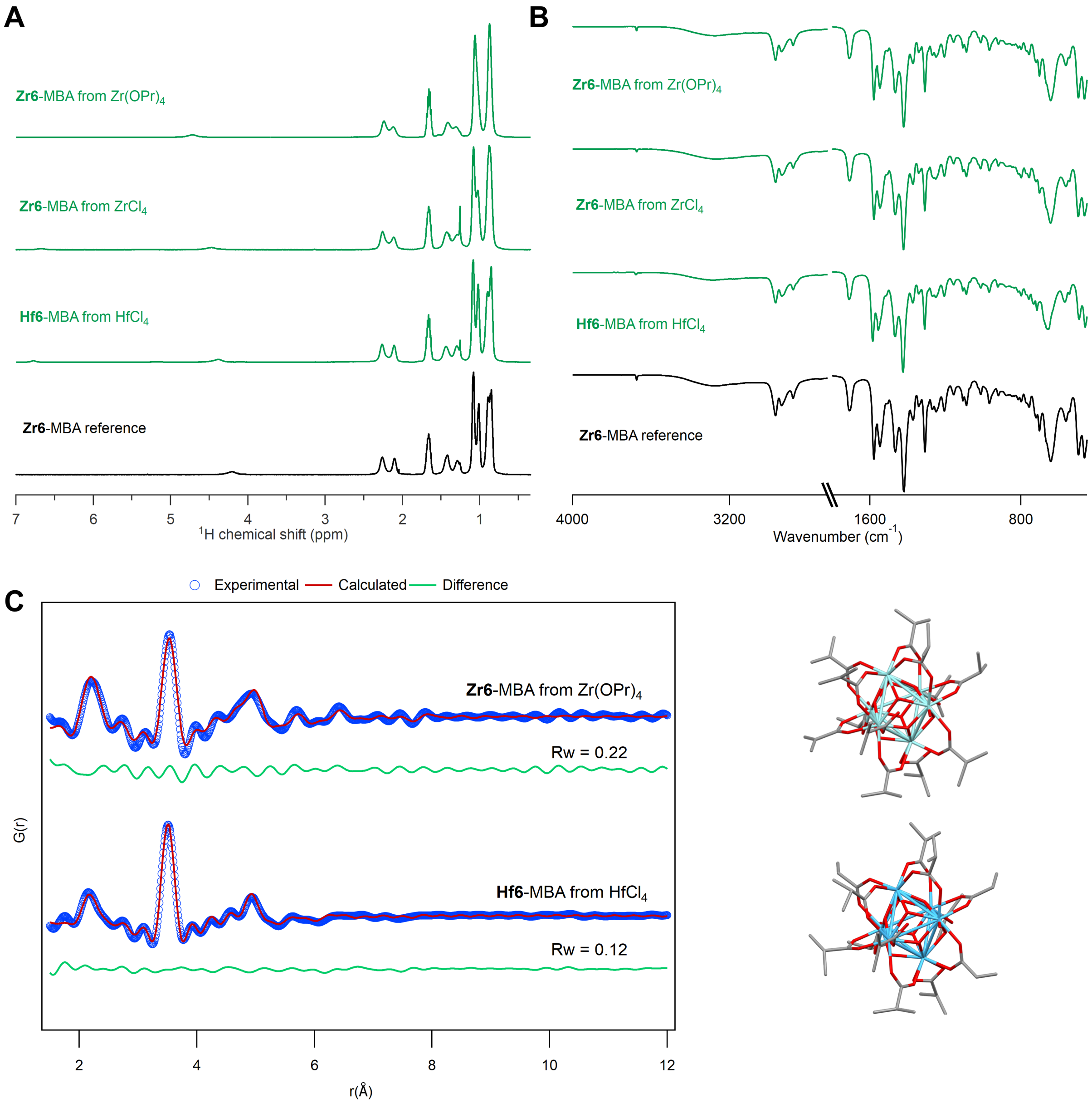

Figure S8: Comparison of (A) NMR spectra, (B) FT-IR spectra and (C) PDF data of **Zr6**-methylbutyrate clusters synthesized from different precursors. The crystal structures used for fitting (same structures as in Figure S2 and Figure S4) is shown on the right side of the corresponding PDF fit. The reference spectra corresponds to clusters synthesized using esterification method.[S2]

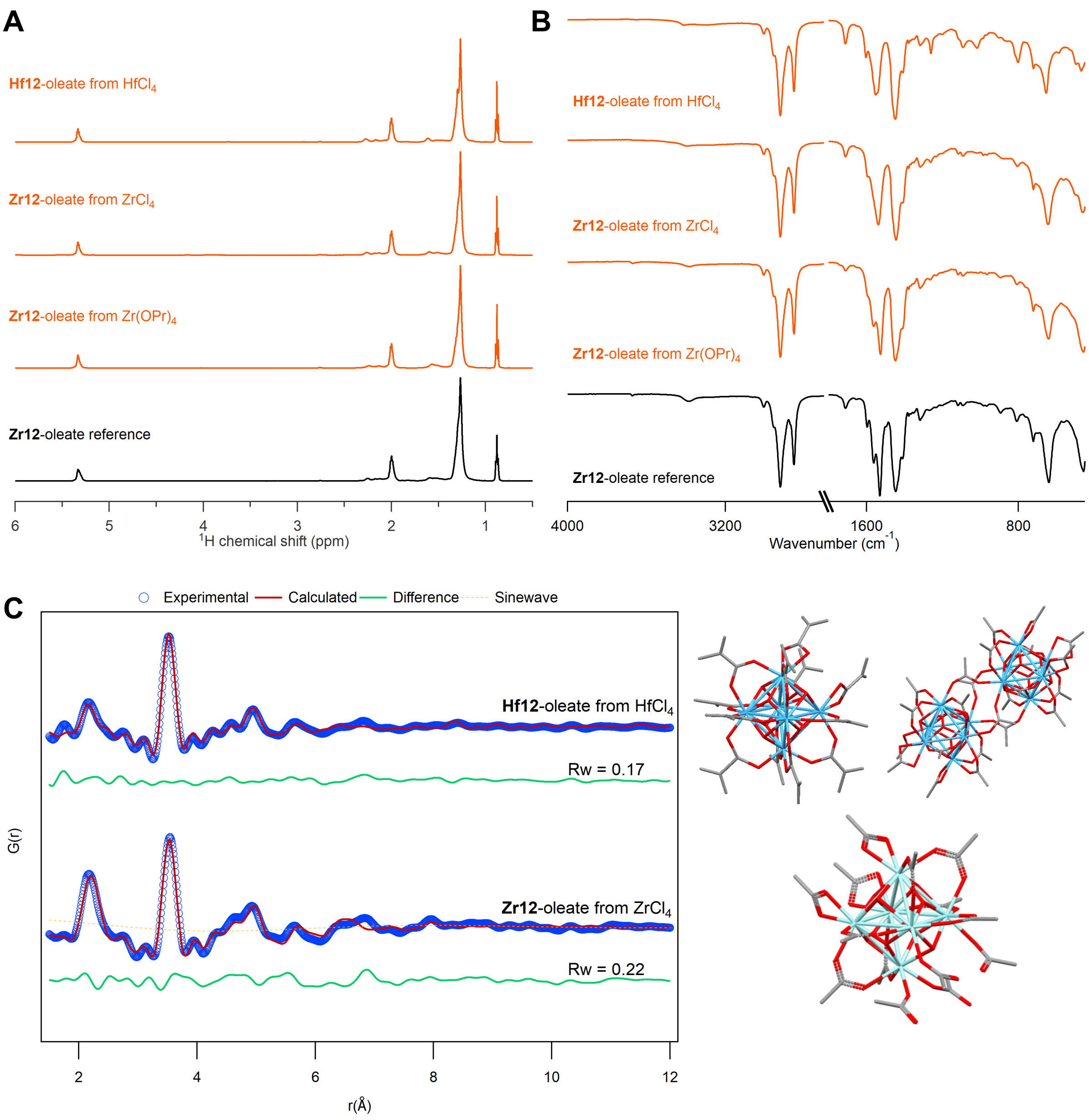

Figure S9: Comparison of (A) NMR spectra, (B) FT-IR spectra and (C)PDF data of **Zr12**-oleate clusters synthesized from different precursors. The crystal structures used for fitting is shown on the right side of the corresponding PDF fit. The structures used for **Hf12**-oleate are derived from structures with CCDC deposition number 106826 and 604534 respectively the structure used for **Zr12**-oleate is derived from structure with CCDC deposition number 604529. Two structures indicate that a dual phase fitting method is used for fitting with the structure on the left being the first phase. The reference spectra corresponds to clusters synthesized using esterification method.[S2]

3.2 Discussion of FTIR spectrum of synthesized clusters

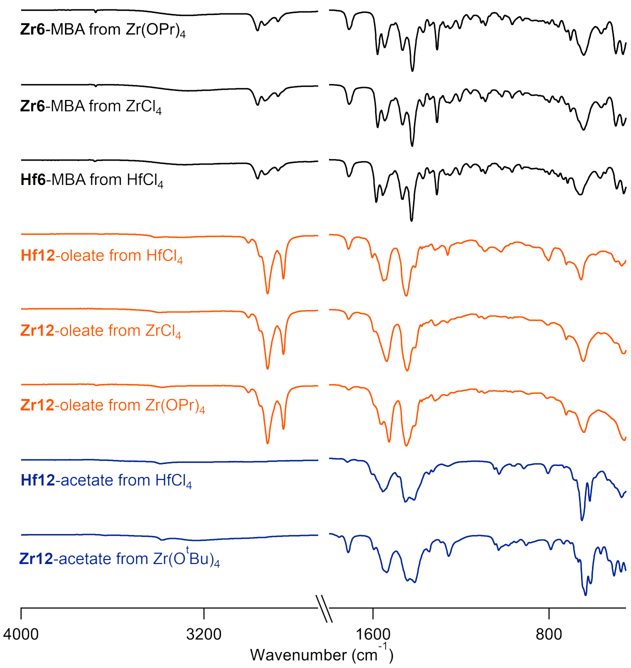

Figure S10: FTIR spectra of various clusters synthesized from different precursors

The FTIR spectra of M_6O_8 clusters capped with acetate, 2-methylbutyrate, and oleate ligands exhibit characteristic vibrational modes associated with the M_6O_8 core, coordinated carboxylate ligands, and μ_3-OH groups. The μ_3-OH groups, which bridge the zirconium centers, give rise to a broad O–H stretching band in the 3000–3500 cm^{-1} range, along with a small, sharp peak at around 3450 cm^{-1}. The free carboxylate, originating from uncoordinated carboxyl groups, is observed around 1715 cm^{-1}. The carboxylate ligands contribute to strong asymmetric (ν_{asym}(COO)) and symmetric (ν_{sym}(COO)) stretching vibrations in the 1500–1650 cm^{-1} and 1350–1500 cm^{-1} regions, respectively. The difference ($\Delta\nu$) between these two bands provides insights into the binding mode of the carboxylate groups. While all three ligands share these fundamental features, key differences arise due to their chain length and branching. The asymmetric stretching vibrations of acetate and oleate appear as multiple broad bands, characteristic of an $(M_6O_8)_2$ dimer containing more complex binding modes. In contrast, 2-methylbutyrate exhibits a split asymmetric stretching band, indicative

of an M_6O_8 monomer containing both bridging and chelating carboxylate binding modes. Oleate, as a long-chain unsaturated fatty acid, displays prominent C–H stretching bands in the 2800–3000 cm^{-1} region and a characteristic C=C stretching vibration around 1650 cm^{-1}, which is absent in acetate and 2-methylbutyrate.

3.3 TGA of clusters

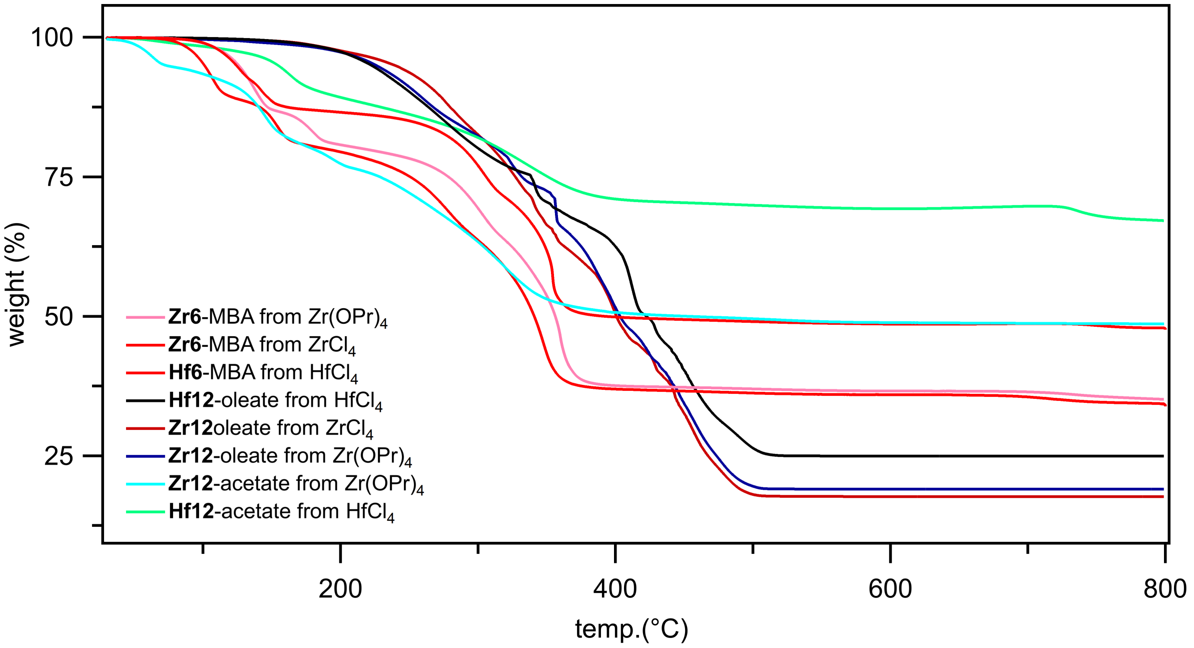

Figure S11: TGA spectra of various clusters synthesized from different precursors using different acids. The endpoint shows how much weightpercent of inorganic content remains compared to the weight of initial species.

4 Bimetallic oxo clusters

4.1 NMR, FTIR and TGA analsysis

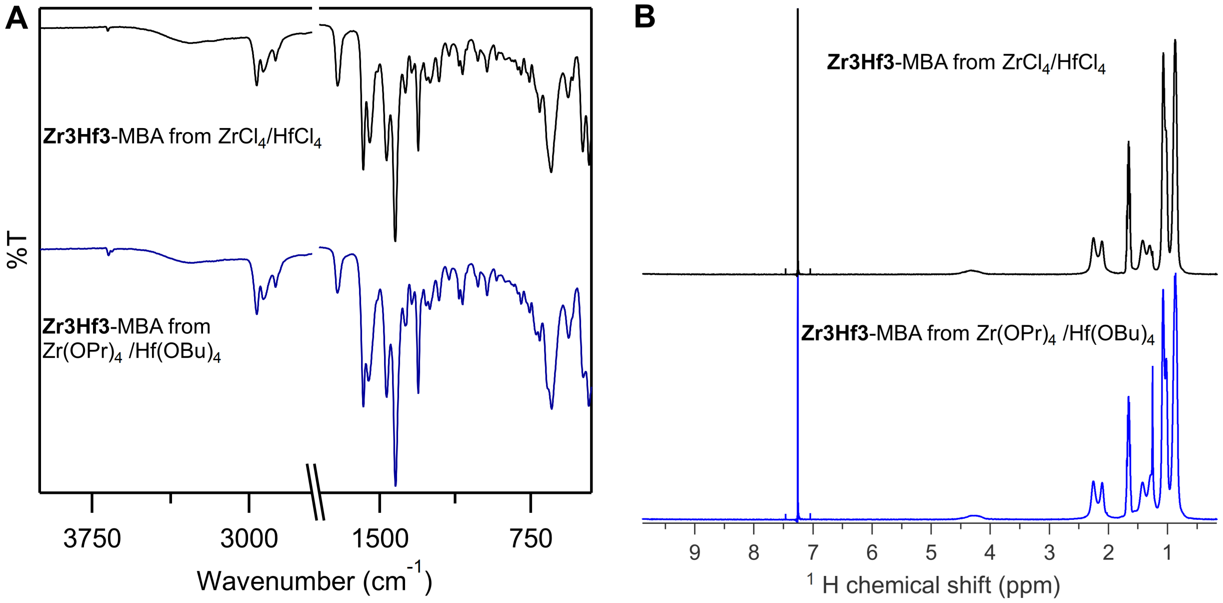

Figure S12: (A)FT-IR and (B)NMR spectra of mixed metal Zr3Hf3 clusters made from chloride and alkoxide precursors

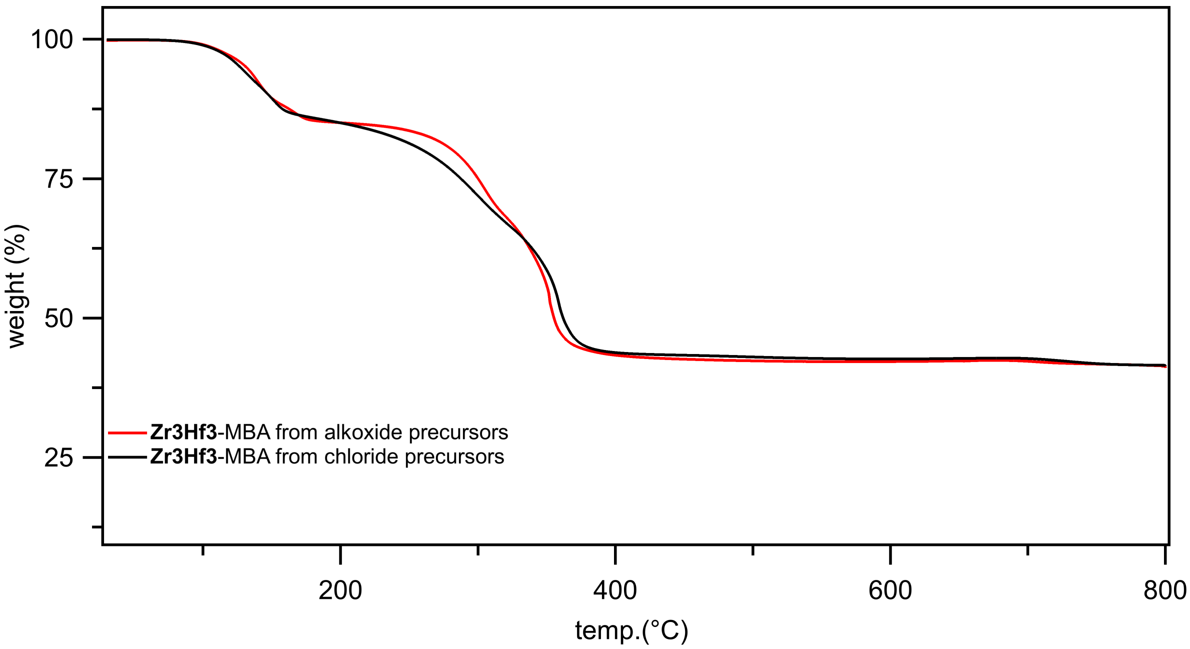

Figure S13: TGA of Zr3Hf3 mixed metal clusters capped with methylbutyrate synthesized from different precursors. Notice the similarity in final weight percent suggesting that the Zr and Hf content is uniform in both the samples.

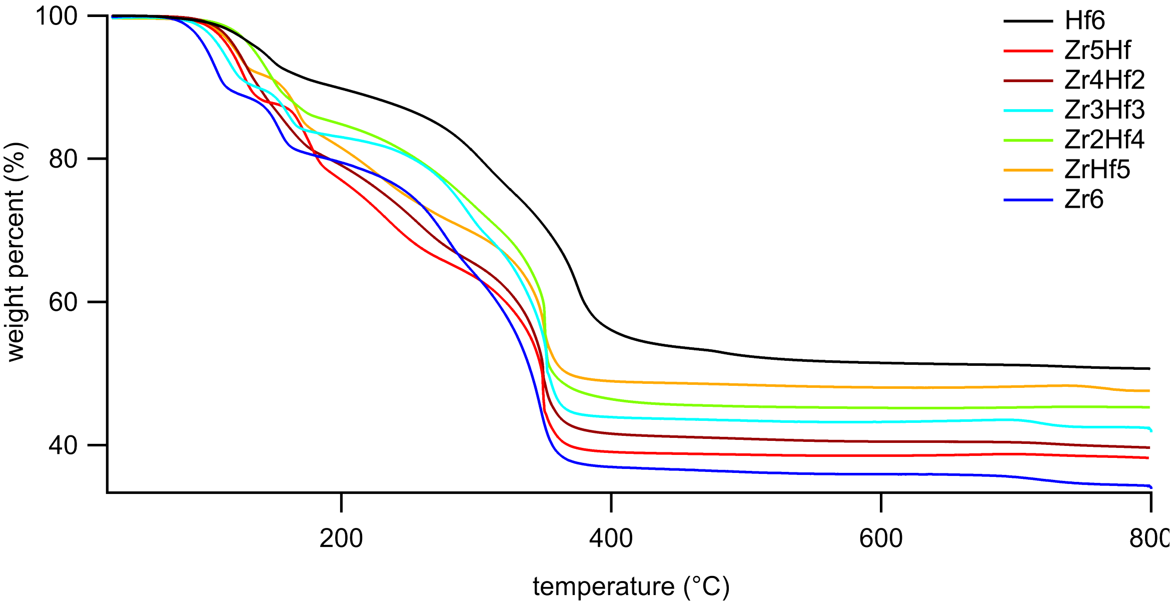

Figure S14: TGA of Zr-Hf mixed metal clusters capped with 2-methylbutyrate synthesized using different starting metal ratios. The final weight confirms that the bulk ratio of metals in the final product is same as the initial target ratio.

4.2 PDF analsysis

The **Zr3Hf3** cluster was also analyzed using PDF, where we observed the characteristic peaks of M_6O_8 octahedra (Figure S15). Due to the presence of both Zr and Hf (having a different atomic form factor), we observed a change in the relative ratio of the M-O and M-M peaks, compared to the **Zr6** and **Hf6** clusters. We generated a series of structural models based on the **Zr6**-methylbutanoate structure (Figure 2) incorporating both Zr and Hf in different stoichiometries and atomic arrangements. Given the asymmetric nature of the M_6O_8 octahedra, this resulted in 64 unique structures with compositions ranging from Zr_6 to Hf_6, including intermediate ratios (Zr_5Hf, Zr_4Hf_2, Zr_3Hf_3, Zr_2Hf_4, and $ZrHf_5$). All models were fitted to the experimental PDF data while maintaining physically reasonable parameters for atomic displacement (Uiso) and correlated atomic motion (delta 2). The best fit with the lowest Rw (0.15), is obtained for Zr_3Hf_3 (Figure S15). The products from the alkoxide and chloride route are just slightly different in the position of the metal atoms within the octahedron. In the case of the mixed clusters made from alkoxide precursors, the *mer* isomer provides the best fit, while from the chloride precursors the *fac* isomer is obtained. To put these results into perspective, the Rw values of all 64 structure models, fitted to the products from the chloride and the alkoxide route, are plotted in Figure S16. The Rw is clearly the worst (highest) for pure Hf_6 and Zr_6 composition, and a minimum is found (Rw = 0.15) for Zr_3Hf_3 (Figure S18). However, for the **Zr3Hf3** composition, there is a spread in Rw values due to the different possible configurations. While the difference between the extremes is appreciable, several lower lying structures are potential true representatives of the real structure. Or differently put, a mixture of structures cannot be excluded. Alternatively, the PDF was modeled by a dual phase model with an equimolar mixture of **Zr6** and **Hf6**. In this case, the Rw value was 0.16 (red dot in Figure S18), which is only slightly worse/higher than the best single phase fit (Figure S18). This close fit is expected since a linear combination of PDFs of two homometallic phases would be similar to the single phase representing their average atomic ratio. PDF analysis thus gives clearly the average composition but is not the

best technique to unambiguously assign the clusters as Zr_3Hf_3.

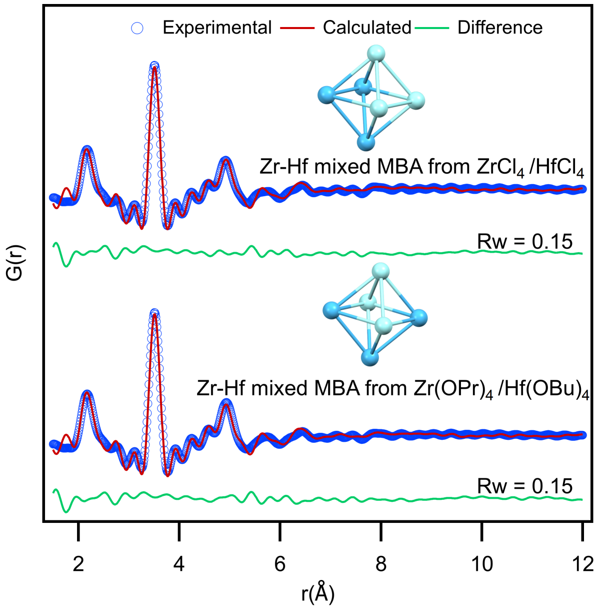

Figure S15: Best PDF fits of $\mathbf{Hf_3Zr_3}$-MBA synthesized from alkoxide and chloride precursors. These represent the best fits obtained from fitting 64 different structural models generated by incorporating both Zr and Hf in different stoichiometries and atomic arrangements, based on the Zr6-methylbutanoate structure. The skeleton indicates the relative arrangement of Zr and Hf atoms for the best structural model determined using PDF.

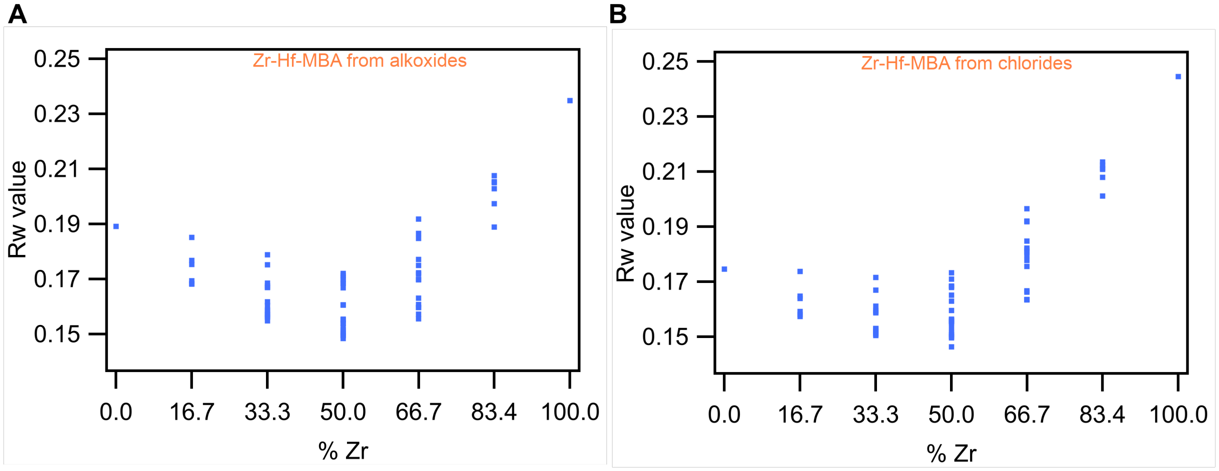

Figure S16: The distribution of Rw values for all the 64 cluster models used to model the mixed metal $\mathbf{Hf_3Zr_3}$-MBA clusters synthesized from (A) alkoxide precursors and (B) chloride precursors. Each data point represents the Rw value from a PDF fit of same data with a different structure of the form $Zr_xHf_{6-x}O_4(OH)_4(C_4H_9COO)_{12}$. The difference between panel A and B is the measured data itself, corresponding to clusters made from the indicated precursors. The subtle differences in Rw value distribution between the samples could be due to slight difference in the ratio of each mixed cluster phase that were identified from HRMS.

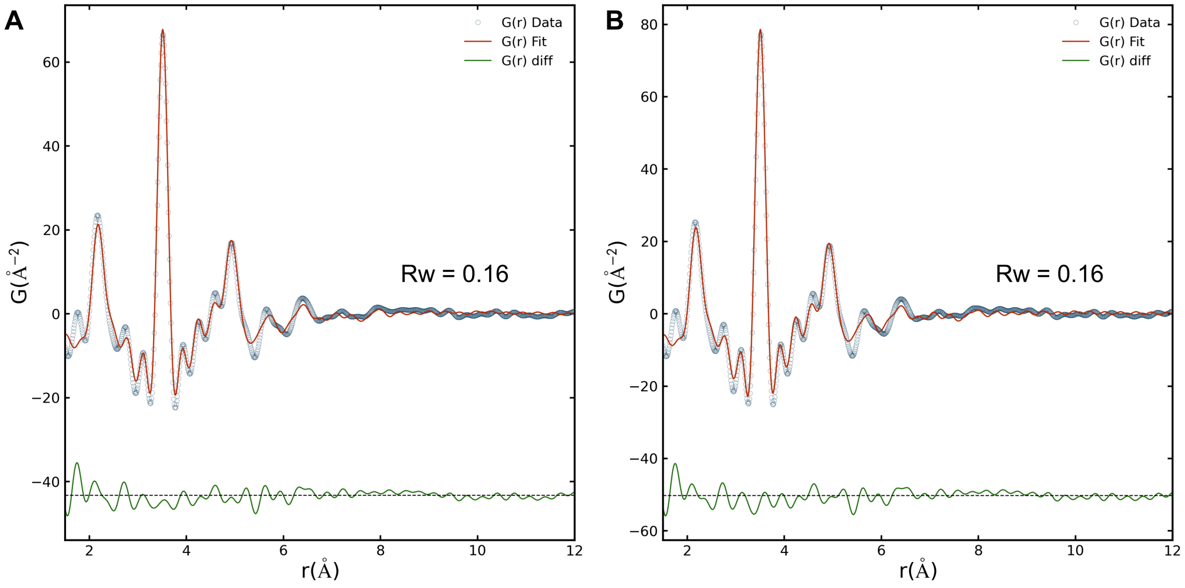

Figure S17: Dual phase PDF fit of mixed clusters synthesized from (A) alkoxide and (B) chloride precursor with **Zr6**-MBA and **Hf6**-MBA (see Figure S2 and Figure S4) as input structures.

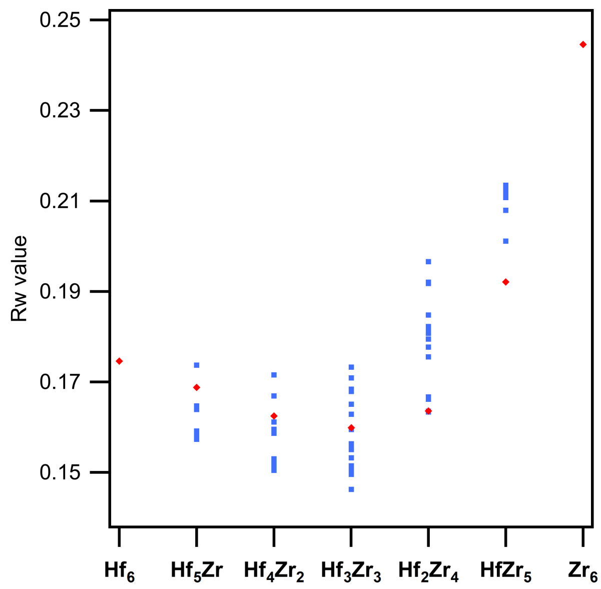

Figure S18: Comparison of Rw values for dual phase and single phase models for $\mathbf{Hf_3Zr_3}$-MBA synthesized from chloride precursors. The blue data point is the Rw value from single phase fits of the experimental data fitted with 64 non-redundant cluster models (since the tetrahedral core is not perfectly symmetric). The red data point represents a dual phase fit with Zr6 and Hf6 as the two phases and scale of each phases fixed to their corresponding metal ratio in the cluster.

4.3 Calculation of metal ratios and extra acid per cluster from ICP-MS data

Table S1: Zr and Hf mass percentages in different samples measured by ICP-MS. Each sample was measured twice, and the values from the two measurements are reported in the table

Sample	Unit	Zr (%)	Hf (%)
Zr5Hf1	mass-%	20.5|20.1	8.32|8.19
Zr4Hf2	mass-%	15.1|15.0	14.8|14.8
Zr3Hf3	mass-%	10.8|10.8	22.1|22.1
Zr2Hf4	mass-%	7.05|7.05	28.3|28.3
Zr1Hf5	mass-%	3.57|3.60	33.5|33.7

The cluster composition was analyzed using ICP–MS to obtain the mass fractions of Zr and Hf in each sample. The general cluster formula was assumed to be

$$\mathrm{Zr}_x\mathrm{Hf}_{6-x}\mathrm{O}_4(\mathrm{OH})_4(\mathrm{C}_4\mathrm{H}_9\mathrm{COO})_{12}\cdot(\mathrm{C}_4\mathrm{H}_9\mathrm{COOH})_\mathrm{n}$$

where n denotes the number of additional hydrogen-bonded 2-methylbutyric acid molecules per cluster.

Determination of Zr:Hf ratio: From the measured ICP–MS mass fractions of Zr and Hf, the corresponding molar amounts were calculated using the atomic weights $M_{\mathrm{Zr}} = 91.224$ g mol^{-1} and $M_{\mathrm{Hf}} = 178.49$ g mol^{-1}. The molar ratio Zr:Hf was then obtained as

$$\frac{n_{\mathrm{Zr}}}{n_{\mathrm{Hf}}} = \frac{w_{\mathrm{Zr}}/M_{\mathrm{Zr}}}{w_{\mathrm{Hf}}/M_{\mathrm{Hf}}}$$

where w_{Zr} and w_{Hf} are the measured mass fractions (g per 100 g of sample). Normalization to six metal atoms per cluster gives the average number x of Zr atoms in $\mathrm{Zr}_x\mathrm{Hf}_{6-x}$.

Cluster molar mass without extra acid: The molar mass of the core cluster (excluding extra 2-methylbutyric acid) was calculated as

$$M_{\mathrm{cluster}} = xM_{\mathrm{Zr}} + (6-x)M_{\mathrm{Hf}} + M_{\mathrm{O_4(OH)_4}} + 12\,M_{\mathrm{MBA^-}}$$

with $M_{O_4(OH)_4} = 4M_O + 4(M_O + M_H)$ and $M_{MBA^-} = 101.122$ g mol^{-1} for the 2-methylbutyrate anion. This yields the theoretical cluster molar mass without extra 2-methylbutyric acid.

Estimation of extra acid content: The total number of clusters per 100 g sample was obtained from the total metal content:

$$n_{\text{cluster}} = \frac{(n_{\text{Zr}} + n_{\text{Hf}})}{6}$$

The mass of the $Zr_xHf_{6-x}O_4(OH)_4(C_4H_9COO)_{12}$ clusters (without extra acid) present in 100 g was then

$$m_{\text{cluster}} = n_{\text{cluster}} \times M_{\text{cluster}}$$

The residual mass

$$m_{\text{extra}} = 100 - m_{\text{cluster}}$$

was attributed to additional hydrogen-bonded 2-methylbutyric acid. Dividing by the molar mass of 2-methylbutyric acid ($M_{\text{MBAH}} = 102.13$ g mol^{-1}) and normalizing per cluster gave the number of extra acid molecules per cluster:

$$n = \frac{m_{\text{extra}}/M_{\text{MBAH}}}{n_{\text{cluster}}}.$$

Following this procedure, the experimentally determined Zr:Hf ratios and corresponding extra acid contents n were calculated for each sample (see Table 1 in the main text).

5 Refined parameters from PDF fitting

Table S2: Refined parameters for the PDF fits where a single phase model is used

Description	Scale	Uiso_Zr	Uiso_Hf	Uiso_C	Uiso_O	delta2	zoom	Rw value	wA	wasyn	wlam	wphi	wr0	wsig
Zr_6-MBA from $ZrCl_4$	0.13	0.002	-	0.009	0.009	2.5	1.003	0.12	-	-	-	-	-	-
Zr_3Hf_3-MBA from chlorides	4.31	0.004	0.003	0.010	0.010	3.5	1.000	0.15	-	-	-	-	-	-
Zr_3Hf_3-MBA from alkoxides	3.89	0.004	0.003	0.012	0.010	3.5	1.000	0.15	-	-	-	-	-	-
Zr_{12}-acetate from $Zr(OtBu)_4$	0.59	0.002	-	0.011	0.009	2.5	1.000	0.16	-	-	-	-	-	-
Zr_6-MBA_from $Zr(OPr)_4$	1.20	0.004	-	0.010	0.010	2.3	1.005	0.22	-	-	-	-	-	-
Hf_6-MBA from $HfCl_4$	2.07	-	0.003	0.030	0.010	3.5	1.000	0.12	-	-	-	-	-	-
Zr_{12}-oleate from $ZrCl_4$	0.45	0.003	-	0.010	0.009	2.5	1.000	0.22	21.464	-2.831	7.255	-0.109	-36.928	3.383

Table S3: Refined parameters for the PDF fits where a dual-phase model is used.

Description	Scale_G1	Scale_G2	Uiso_Hf_G1	Uiso_Hf_G2	Uiso_O_G1	Uiso_O_G2	Uiso_C_G1	Uiso_C_G2	delta2_G1	delta2_G2	Rw
Hf_{12} acetate from $HfCl_4$	1.25	0.688	0.003	0.003	0.010	0.010	0.010	0.010	3.50	1.759	0.13
Hf_{12} oleate from $HfCl_4$	1.347	0.851	0.003	0.003	0.010	0.010	0.090	0.090	1.92	3.500	0.17

For all other PDF fits, the parameters in the following range were used:

Uiso Zr = 0.002 to 0.007

Uiso Hf = 0.002 to 0.006

Uiso O = 0.009 to 0.08

Uiso C = 0.009 to 0.08

Delta 2 = 0 to 2.5 (Zr) and 0 to 3.5 (Hf)

zoom = 0.99 to 1.01

6 Additional characterization of Nb and Ta clusters

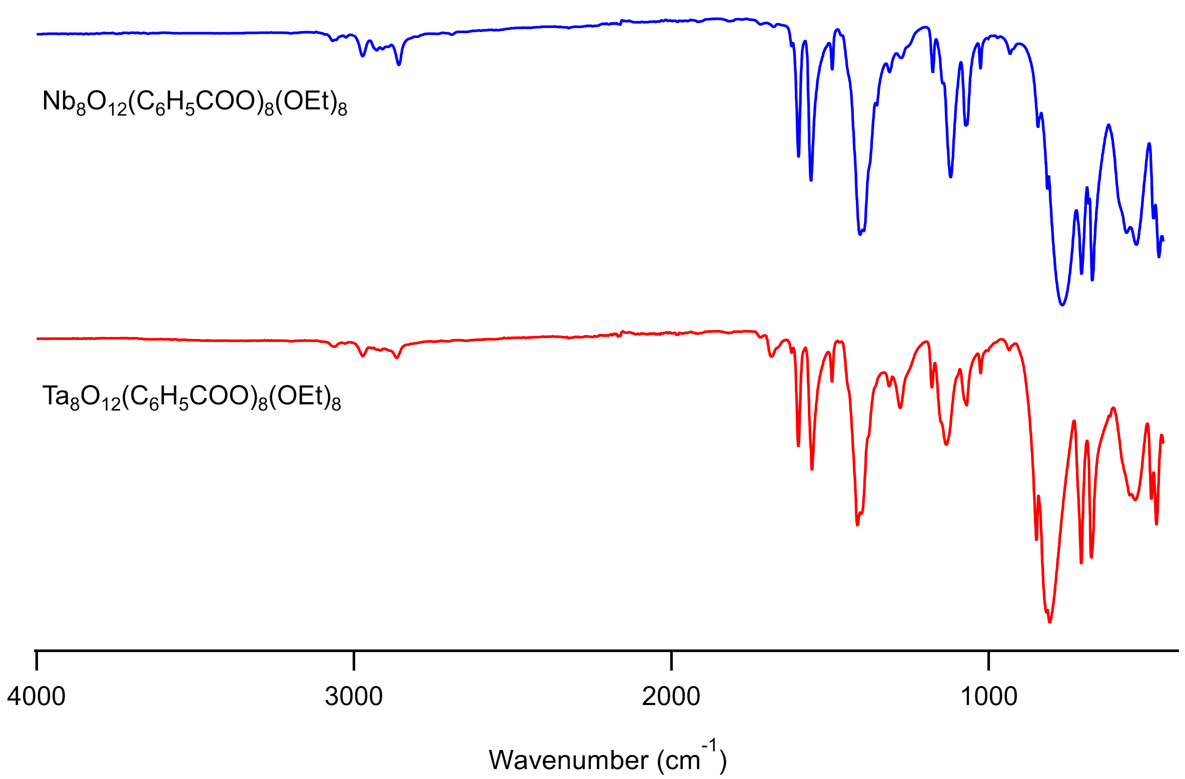

Figure S19: FTIR spectrum of $Nb_8O_{12}(C_6H_5COO)_8(OEt)_8$ and $Ta_8O_{12}(C_6H_5COO)_8(OEt)_8$ clusters.

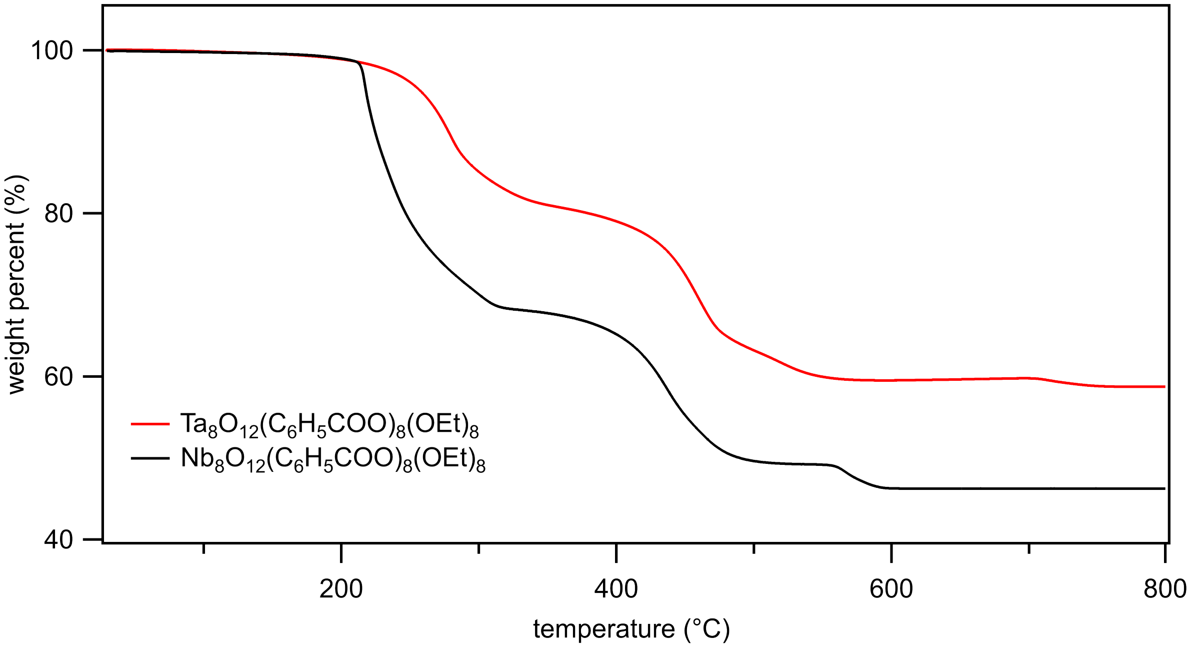

Figure S20: TGA of $Nb_8O_{12}(C_6H_5COO)_8(OEt)_8$ and $Ta_8O_{12}(C_6H_5COO)_8(OEt)_8$ clusters. The remaining percent weight measured by TGA is 46.18 % and 58.73 % respectively for Nb and Ta clusters. The corresponding theoretical values are 46.95 % and 59.53 % respectively.

References

(S1) Dhaene, E.; Seno, C.; De Roo, J. Synthesis of zirconium(IV) and hafnium(IV) isopropoxide, sec-butoxide and tert-butoxide. *Dalton Trans.* **2024**, *53*, 11769–11777.

(S2) Van den Eynden, D.; Pokratath, R.; Mathew, J. P.; Goossens, E.; De Buysser, K.; De Roo, J. Fatty acid capped, metal oxo clusters as the smallest conceivable nanocrystal prototypes. *Chem. Sci.* **2023**, *14*, 573–585.
