# Supplementary material for: Mechanism‐Guided Precision Hydrolysis of Early Transition Metals to Access (Mixed‐Metal) Oxo Clusters
Source: Angew Chem Int Ed Engl. 2026 Feb 24;65(15):e25769. doi: 10.1002/anie.202525769 (PMC13053926; doi:10.1002/anie.202525769)

checkCIF/PLATON report

Structure factors have been supplied for datablock(s) mjp091_150k

THIS REPORT IS FOR GUIDANCE ONLY. IF USED AS PART OF A REVIEW PROCEDURE FOR PUBLICATION, IT SHOULD NOT REPLACE THE EXPERTISE OF AN EXPERIENCED CRYSTALLOGRAPHIC REFEREE.

No syntax errors found. CIF dictionary Interpreting this report

Datablock: mjp091_150k

Bond precision:	C-C = 0.0113 A		Wavelength=1.34143
Cell:	a=15.2795(5)	b=27.0474(6)	c=11.1015(4)
	alpha=90	beta=100.348(3)	gamma=90
Temperature:	150 K		

	Calculated	Reported
Volume	4513.3(2)	4513.3(2)
Space group	P 21/c	P 1 21/c 1
Hall group	-P 2ybc	-P 2ybc
Moiety formula	C72 H80 Nb8 O36, 1.8(C2 H3 N)	C72 H80 Nb8 O36, 1.8(C2 H3 N)
Sum formula	C75.60 H85.40 N1.80 Nb8 O36	C75.60 H85.40 N1.80 Nb8 O36
Mr	2338.54	2338.53
Dx,g cm-3	1.721	1.721
Z	2	2
Mu (mm-1)	5.871	5.871
F000	2335.2	2335.0
F000'	2334.94	
h,k,lmax	18,33,13	18,32,13
Nref	8820	8683
Tmin,Tmax	0.400,0.556	0.082,0.112
Tmin'	0.285	

Correction method= # Reported T Limits: Tmin=0.082 Tmax=0.112
AbsCorr = MULTI-SCAN

Data completeness= 0.984 Theta(max)= 55.692

R(reflections)= 0.0545(6678) wR2(reflections)= 0.1473(8683)

S = 1.011 Npar= 562

The following ALERTS were generated. Each ALERT has the format
test-name_ALERT_alert-type_alert-level.
Click on the hyperlinks for more details of the test.

Alert level C

PLAT077_ALERT_4_C Unitcell Contains Non-integer Number of Atoms .. Please Check
PLAT220_ALERT_2_C NonSolvent Resd 1 C Ueq(max)/Ueq(min) Range 4.7 Ratio
PLAT222_ALERT_3_C NonSolvent Resd 1 H Uiso(max)/Uiso(min) Range 5.0 Ratio
PLAT234_ALERT_4_C Large Hirshfeld Difference O2 --C28 . 0.16 Ang.
PLAT241_ALERT_2_C High 'MainMol' Ueq as Compared to Neighbors of C28 Check
PLAT242_ALERT_2_C Low 'MainMol' Ueq as Compared to Neighbors of O2 Check
PLAT242_ALERT_2_C Low 'MainMol' Ueq as Compared to Neighbors of C8 Check
PLAT242_ALERT_2_C Low 'MainMol' Ueq as Compared to Neighbors of C19 Check
PLAT342_ALERT_3_C Low Bond Precision on C-C Bonds .... 0.01129 Ang.
PLAT360_ALERT_2_C Short C(sp3)-C(sp3) Bond C19 - C20 . 1.42 Ang.
PLAT906_ALERT_3_C Large K Value in the Analysis of Variance ...... 3.110 Check
PLAT911_ALERT_3_C Missing FCF Refl Between Thmin & STh/L= 0.600 17 Report
PLAT971_ALERT_2_C Check Calcd Resid. Dens. 0.84Ang From Nb4 2.27 eA-3
PLAT971_ALERT_2_C Check Calcd Resid. Dens. 0.90Ang From Nb1 1.80 eA-3
PLAT976_ALERT_2_C Check Calcd Resid. Dens. 1.04Ang From O6 . -0.55 eA-3

Alert level G

ABSMU01_ALERT_1_G Calculation of _exptl_absorpt_correction_mu
not performed for this radiation type.
PLAT002_ALERT_2_G Number of Distance or Angle Restraints on AtSite 6 Note
PLAT003_ALERT_2_G Number of Uiso or Uij Restrained non-H Atoms ... 9 Report
PLAT083_ALERT_2_G SHELXL Second Parameter in WGHT Unusually Large 9.56 Why ?
PLAT176_ALERT_4_G The CIF-Embedded .res File Contains SADI Records 3 Report
PLAT178_ALERT_4_G The CIF-Embedded .res File Contains SIMU Records 3 Report
PLAT186_ALERT_4_G The CIF-Embedded .res File Contains ISOR Records 2 Report
PLAT187_ALERT_4_G The CIF-Embedded .res File Contains RIGU Records 3 Report
PLAT191_ALERT_3_G A Non-default SADI Restraint Value has been used 0.0400 Report
PLAT300_ALERT_4_G Atom Site Occupancy of C29 Constrained at 0.5 Check
PLAT300_ALERT_4_G Atom Site Occupancy of C30 Constrained at 0.5 Check
PLAT300_ALERT_4_G Atom Site Occupancy of H28A Constrained at 0.5 Check
PLAT300_ALERT_4_G Atom Site Occupancy of H28B Constrained at 0.5 Check
PLAT300_ALERT_4_G Atom Site Occupancy of H28C Constrained at 0.5 Check
PLAT300_ALERT_4_G Atom Site Occupancy of H28D Constrained at 0.5 Check
PLAT300_ALERT_4_G Atom Site Occupancy of H29A Constrained at 0.5 Check
PLAT300_ALERT_4_G Atom Site Occupancy of H29B Constrained at 0.5 Check
PLAT300_ALERT_4_G Atom Site Occupancy of H29C Constrained at 0.5 Check
PLAT300_ALERT_4_G Atom Site Occupancy of H30A Constrained at 0.5 Check
PLAT300_ALERT_4_G Atom Site Occupancy of H30B Constrained at 0.5 Check
PLAT300_ALERT_4_G Atom Site Occupancy of H30C Constrained at 0.5 Check
PLAT300_ALERT_4_G Atom Site Occupancy of N1 Constrained at 0.5 Check
PLAT300_ALERT_4_G Atom Site Occupancy of C38 Constrained at 0.5 Check
PLAT300_ALERT_4_G Atom Site Occupancy of C39 Constrained at 0.5 Check
PLAT300_ALERT_4_G Atom Site Occupancy of H39A Constrained at 0.5 Check
PLAT300_ALERT_4_G Atom Site Occupancy of H39B Constrained at 0.5 Check
PLAT300_ALERT_4_G Atom Site Occupancy of H39C Constrained at 0.5 Check
PLAT300_ALERT_4_G Atom Site Occupancy of N2 Constrained at 0.4 Check
PLAT300_ALERT_4_G Atom Site Occupancy of C40 Constrained at 0.4 Check
PLAT300_ALERT_4_G Atom Site Occupancy of C41 Constrained at 0.4 Check
PLAT300_ALERT_4_G Atom Site Occupancy of H41A Constrained at 0.4 Check

```
PLAT300_ALERT_4_G Atom Site Occupancy of H41B       Constrained at        0.4 Check
PLAT300_ALERT_4_G Atom Site Occupancy of H41C       Constrained at        0.4 Check
PLAT301_ALERT_3_G Main Residue  Disorder .............(Resd  1  )          2% Note
PLAT302_ALERT_4_G Anion/Solvent/Minor-Residue Disorder (Resd  2  )       100% Note
PLAT302_ALERT_4_G Anion/Solvent/Minor-Residue Disorder (Resd  3  )       100% Note
PLAT304_ALERT_4_G Non-Integer Number of Atoms in ..... (Resd  3  )       2.40 Check
PLAT794_ALERT_5_G Tentative Bond Valency for Nb2       (V)       .       5.12 Info
PLAT794_ALERT_5_G Tentative Bond Valency for Nb3       (V)       .       5.13 Info
PLAT860_ALERT_3_G Number of Least-Squares Restraints .............         93 Note
PLAT910_ALERT_3_G Missing # of FCF Reflection(s) Below Theta(Min).          1 Note
PLAT912_ALERT_4_G Missing # of FCF Reflections Above STh/L=  0.600        114 Note
PLAT913_ALERT_3_G Missing # of Very Strong Reflections in FCF ....          1 Note
PLAT933_ALERT_2_G Number of HKL-OMIT Records in Embedded .res File          8 Note
PLAT941_ALERT_3_G Average HKL Measurement Multiplicity ..........         4.7 Low
PLAT978_ALERT_2_G Number C-C Bonds with Positive Residual Density.          0 Info
```

---

0 **ALERT level A** = Most likely a serious problem - resolve or explain
0 **ALERT level B** = A potentially serious problem, consider carefully
15 **ALERT level C** = Check. Ensure it is not caused by an omission or oversight
46 **ALERT level G** = General information/check it is not something unexpected

1 ALERT type 1 CIF construction/syntax error, inconsistent or missing data
14 ALERT type 2 Indicator that the structure model may be wrong or deficient
10 ALERT type 3 Indicator that the structure quality may be low
34 ALERT type 4 Improvement, methodology, query or suggestion
2 ALERT type 5 Informative message, check

It is advisable to attempt to resolve as many as possible of the alerts in all categories. Often the minor alerts point to easily fixed oversights, errors and omissions in your CIF or refinement strategy, so attention to these fine details can be worthwhile. In order to resolve some of the more serious problems it may be necessary to carry out additional measurements or structure refinements. However, the purpose of your study may justify the reported deviations and the more serious of these should normally be commented upon in the discussion or experimental section of a paper or in the "special_details" fields of the CIF. checkCIF was carefully designed to identify outliers and unusual parameters, but every test has its limitations and alerts that are not important in a particular case may appear. Conversely, the absence of alerts does not guarantee there are no aspects of the results needing attention. It is up to the individual to critically assess their own results and, if necessary, seek expert advice.

Publication of your CIF in IUCr journals

A basic structural check has been run on your CIF. These basic checks will be run on all CIFs submitted for publication in IUCr journals (*Acta Crystallographica*, *Journal of Applied Crystallography*, *Journal of Synchrotron Radiation*); however, if you intend to submit to *Acta Crystallographica Section C* or *E* or *IUCrData*, you should make sure that full publication checks are run on the final version of your CIF prior to submission.

Publication of your CIF in other journals

Please refer to the *Notes for Authors* of the relevant journal for any special instructions relating to CIF submission.

PLATON version of 06/07/2023; check.def file version of 30/06/2023

Datablock mjp091_150k - ellipsoid plot

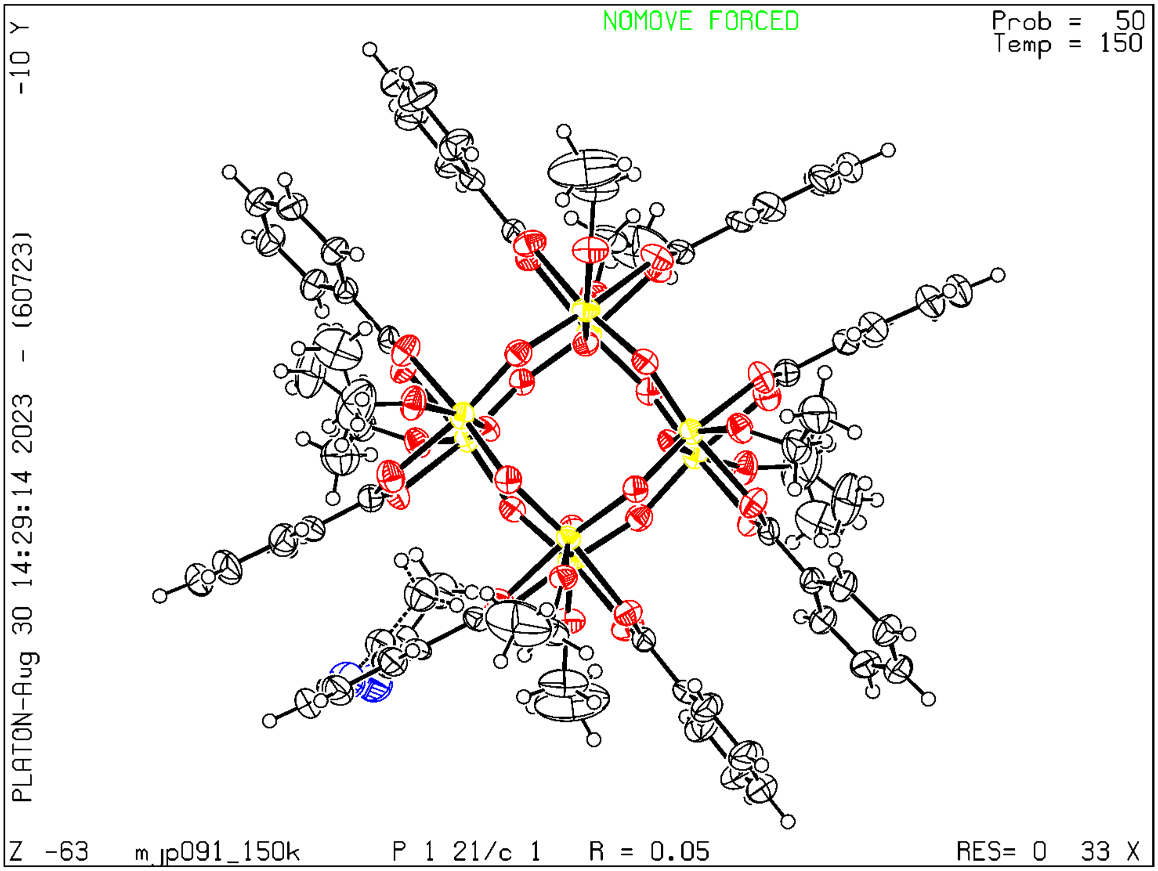

Supplement: Supplementary file 2 — Supporting File 2: anie71298–sup–0002–Data.zip. [file ANIE-65-e25769-s002.zip › CCDC_2312388/MJP091_150K_cifreport.pdf]
