# Supplementary material for: Mechanism‐Guided Precision Hydrolysis of Early Transition Metals to Access (Mixed‐Metal) Oxo Clusters
Source: Angew Chem Int Ed Engl. 2026 Feb 24;65(15):e25769. doi: 10.1002/anie.202525769 (PMC13053926; doi:10.1002/anie.202525769)
Supplement: Supplementary file 2 — Supporting File 2: anie71298–sup–0002–Data.zip. [file ANIE-65-e25769-s002.zip › CCDC_2312388/MJP091_150K_tables.html]

MJP091\_150K


# MJP091\_150K

b"\n \n \n "

Table 1 Crystal data and structure refinement for MJP091\_150K.

| Identification code | MJP091\_150K |
| Empirical formula | C75.6H85.4N1.8Nb8O36 |
| Formula weight | 2338.53 |
| Temperature/K | 150 |
| Crystal system | monoclinic |
| Space group | P21/c |
| a/Å | 15.2795(5) |
| b/Å | 27.0474(6) |
| c/Å | 11.1015(4) |
| α/° | 90 |
| β/° | 100.348(3) |
| γ/° | 90 |
| Volume/Å3 | 4513.3(2) |
| Z | 2 |
| ρcalcg/cm3 | 1.721 |
| μ/mm‑1 | 5.871 |
| F(000) | 2335.0 |
| Crystal size/mm3 | 0.19 × 0.15 × 0.1 |
| Radiation | GaKα (λ = 1.34143) |
| 2Θ range for data collection/° | 5.686 to 111.384 |
| Index ranges | -18 ≤ h ≤ 18, -32 ≤ k ≤ 16, -13 ≤ l ≤ 12 |
| Reflections collected | 40957 |
| Independent reflections | 8683 [Rint = 0.0873, Rsigma = 0.0497] |
| Data/restraints/parameters | 8683/93/562 |
| Goodness-of-fit on F2 | 1.011 |
| Final R indexes [I>=2σ (I)] | R1 = 0.0545, wR2 = 0.1349 |
| Final R indexes [all data] | R1 = 0.0775, wR2 = 0.1473 |
| Largest diff. peak/hole / e Å-3 | 2.19/-1.30 |

Table 2 Fractional Atomic Coordinates (×104) and Equivalent Isotropic Displacement Parameters (Å2×103) for MJP091\_150K. Ueq is defined as 1/3 of the trace of the orthogonalised UIJ tensor.

| Atom | *x* | *y* | *z* | U(eq) |
| --- | --- | --- | --- | --- |
| Nb3 | 6509.1(3) | 5614.1(2) | 4080.2(5) | 29.23(14) |
| Nb2 | 4356.4(3) | 6103.0(2) | 4485.7(5) | 30.81(14) |
| Nb1 | 3246.3(3) | 5023.9(2) | 2973.2(5) | 32.84(15) |
| Nb4 | 5395.5(3) | 4528.0(2) | 2552.2(5) | 32.15(14) |
| O1 | 5469(3) | 6009.4(14) | 3974(4) | 32.9(9) |
| O4 | 4593(3) | 5628.6(14) | 5766(4) | 31.3(9) |
| O6 | 3647(3) | 5685.0(15) | 3320(4) | 34.8(9) |
| O8 | 3685(3) | 4763.7(14) | 4544(4) | 32.9(9) |
| O10 | 4226(3) | 4808.8(15) | 2283(4) | 36.3(10) |
| O12 | 7109(3) | 6008.7(15) | 3085(4) | 36.5(10) |
| O14 | 6039(3) | 5128.8(14) | 2909(4) | 34.3(9) |
| O16 | 5009(3) | 3405.1(14) | 4073(4) | 39.1(10) |
| O18 | 7757(3) | 5224.5(15) | 4405(4) | 37.2(10) |
| O9 | 4064(3) | 6719.3(15) | 3702(4) | 40.7(10) |
| O5 | 2622(3) | 4306.4(16) | 2681(4) | 42.1(11) |
| O11 | 7890(3) | 4812.9(17) | 6182(4) | 41.4(11) |
| O3 | 2789(3) | 3901.9(14) | 4474(4) | 36.3(10) |
| O13 | 3378(3) | 5903.3(16) | 7140(5) | 43.2(11) |
| O7 | 3207(3) | 6257.3(17) | 5295(5) | 44.1(11) |
| O15 | 4791(3) | 3809.0(15) | 2283(4) | 40.1(10) |
| O2 | 5517(3) | 4470.0(16) | 906(4) | 44.9(11) |
| O17 | 2412(3) | 5166.3(19) | 1537(5) | 49.3(12) |
| C22 | 8854(4) | 4609(2) | 4823(7) | 36.2(14) |
| C2 | 2028(4) | 3506(2) | 2692(7) | 37.7(14) |
| C21 | 8112(4) | 4900(2) | 5149(6) | 33.4(13) |
| C31 | 4718(4) | 3438(2) | 2949(6) | 33.5(13) |
| C32 | 4271(4) | 2999(2) | 2324(6) | 33.3(13) |
| C33 | 3800(4) | 3035(2) | 1137(6) | 38.0(14) |
| C34 | 3421(5) | 2618(2) | 529(7) | 44.0(16) |
| C1 | 2523(4) | 3935(2) | 3355(7) | 35.7(14) |
| C11 | 2233(4) | 6494(2) | 6615(7) | 40.7(15) |
| C37 | 4347(5) | 2543(2) | 2912(7) | 40.6(15) |
| C36 | 3972(5) | 2119(2) | 2304(7) | 46.2(17) |
| C35 | 3516(5) | 2157(2) | 1115(7) | 48.1(18) |
| C10 | 2983(4) | 6193(2) | 6322(7) | 38.8(15) |
| C23 | 9186(5) | 4715(3) | 3775(7) | 45.7(16) |
| C27 | 9209(5) | 4219(3) | 5569(8) | 51.1(18) |
| C12 | 1890(5) | 6875(3) | 5826(8) | 51.2(18) |
| C17 | 4019(5) | 7227(3) | 3990(9) | 55(2) |
| C3 | 1908(5) | 3083(2) | 3325(8) | 54(2) |
| C5 | 1105(5) | 2710(3) | 1507(8) | 57(2) |
| C25 | 10214(5) | 4050(3) | 4206(9) | 60(2) |
| C24 | 9887(5) | 4436(3) | 3495(8) | 54.2(19) |
| C19 | 7946(5) | 6175(3) | 2921(9) | 58(2) |
| C16 | 1902(5) | 6415(3) | 7675(8) | 54.9(19) |
| C15 | 1217(5) | 6716(3) | 7935(10) | 63(2) |
| C4 | 1458(5) | 2682(3) | 2717(9) | 61(2) |
| C26 | 9895(5) | 3941(3) | 5252(9) | 55(2) |
| C18 | 3470(7) | 7512(3) | 3024(12) | 83(3) |
| C13 | 1220(5) | 7177(3) | 6127(10) | 63(2) |
| C6 | 1201(6) | 3131(3) | 873(8) | 65(2) |
| C14 | 904(5) | 7101(3) | 7183(10) | 64(2) |
| C7 | 1658(6) | 3535(3) | 1453(8) | 57(2) |
| N1 | 507(11) | 6554(7) | 1638(19) | 73(5) |
| C8 | 1532(6) | 5063(4) | 980(10) | 80(3) |
| C38 | 1239(11) | 6490(7) | 1620(20) | 55(4) |
| C39 | 2154(12) | 6403(7) | 1600(20) | 66(5) |
| C20 | 8022(10) | 6196(8) | 1665(15) | 146(7) |
| C29 | 5201(16) | 3987(10) | -700(20) | 88(6) |
| C28 | 5864(11) | 4222(5) | -22(12) | 108(4) |
| C30 | 5650(20) | 3773(9) | -400(20) | 93(7) |
| C9 | 1345(11) | 5232(7) | -276(14) | 153(8) |
| C41 | 1991(13) | 6422(8) | 2090(30) | 59(6) |
| C40 | 1079(12) | 6569(7) | 2020(20) | 47(5) |
| N2 | 383(12) | 6699(9) | 2030(20) | 72(6) |

Table 3 Anisotropic Displacement Parameters (Å2×103) for MJP091\_150K. The Anisotropic displacement factor exponent takes the form: -2π2[h2a\*2U11+2hka\*b\*U12+…].

| Atom | U11 | U22 | U33 | U23 | U13 | U12 |
| --- | --- | --- | --- | --- | --- | --- |
| Nb3 | 26.1(2) | 26.9(2) | 35.8(3) | 2.89(18) | 8.36(19) | -0.93(17) |
| Nb2 | 30.1(3) | 29.4(2) | 33.9(3) | 0.73(19) | 8.3(2) | 2.64(17) |
| Nb1 | 28.6(3) | 37.6(3) | 32.6(3) | 2.1(2) | 6.2(2) | -4.61(19) |
| Nb4 | 37.7(3) | 27.5(2) | 33.8(3) | -0.47(19) | 13.2(2) | -3.50(19) |
| O1 | 35(2) | 30.4(19) | 35(2) | 2.2(17) | 11.8(17) | -0.8(16) |
| O4 | 26.1(19) | 35(2) | 35(2) | 1.1(17) | 11.2(17) | 3.0(15) |
| O6 | 30(2) | 37(2) | 37(3) | 1.2(18) | 7.9(18) | 1.3(16) |
| O8 | 32(2) | 30.0(19) | 38(3) | -0.3(17) | 8.6(17) | -3.0(16) |
| O10 | 38(2) | 34(2) | 37(3) | 0.4(17) | 7.6(18) | -3.2(17) |
| O12 | 32(2) | 33.0(19) | 46(3) | 0.6(18) | 9.2(18) | -6.4(16) |
| O14 | 38(2) | 34(2) | 33(3) | 4.0(17) | 11.2(18) | -1.4(17) |
| O16 | 53(3) | 29.3(19) | 34(3) | -1.5(17) | 5(2) | -4.7(18) |
| O18 | 30(2) | 36(2) | 47(3) | 8.7(19) | 10.0(18) | 1.0(17) |
| O9 | 40(2) | 36(2) | 46(3) | 5.6(19) | 7(2) | 10.0(18) |
| O5 | 43(2) | 45(2) | 38(3) | 2(2) | 7(2) | -13.8(19) |
| O11 | 27(2) | 55(3) | 43(3) | 11(2) | 7.4(19) | 2.5(19) |
| O3 | 37(2) | 33(2) | 37(3) | 0.7(17) | 1.2(19) | -3.1(17) |
| O13 | 47(3) | 43(2) | 45(3) | 4(2) | 21(2) | 3(2) |
| O7 | 39(2) | 48(2) | 48(3) | 1(2) | 15(2) | 13(2) |
| O15 | 58(3) | 30(2) | 34(3) | 1.7(18) | 13(2) | -9.3(19) |
| O2 | 60(3) | 37(2) | 44(3) | -5(2) | 26(2) | -4(2) |
| O17 | 39(2) | 60(3) | 47(3) | 4(2) | 2(2) | -11(2) |
| C22 | 19(3) | 34(3) | 55(4) | -1(3) | 3(3) | 0(2) |
| C2 | 23(3) | 43(3) | 48(4) | -8(3) | 10(3) | -1(2) |
| C21 | 27(3) | 35(3) | 39(4) | 5(3) | 8(3) | -1(2) |
| C31 | 37(3) | 28(3) | 40(4) | -5(2) | 19(3) | -1(2) |
| C32 | 35(3) | 29(3) | 37(4) | -1(2) | 11(3) | -1(2) |
| C33 | 44(3) | 30(3) | 40(4) | 1(3) | 8(3) | -5(3) |
| C34 | 44(4) | 39(3) | 47(4) | 1(3) | 0(3) | -4(3) |
| C1 | 29(3) | 32(3) | 48(4) | -7(3) | 12(3) | -2(2) |
| C11 | 30(3) | 43(3) | 52(4) | -8(3) | 15(3) | -4(3) |
| C37 | 54(4) | 29(3) | 40(4) | 1(3) | 10(3) | -3(3) |
| C36 | 57(4) | 31(3) | 50(5) | 0(3) | 6(3) | -2(3) |
| C35 | 47(4) | 37(3) | 57(5) | -7(3) | 3(3) | -2(3) |
| C10 | 37(3) | 33(3) | 50(5) | -2(3) | 16(3) | -3(2) |
| C23 | 39(3) | 52(4) | 46(4) | -3(3) | 8(3) | 3(3) |
| C27 | 38(4) | 52(4) | 63(5) | 9(4) | 10(3) | 2(3) |
| C12 | 38(4) | 53(4) | 64(5) | -6(4) | 11(3) | 6(3) |
| C17 | 56(4) | 39(3) | 72(6) | 5(3) | 13(4) | 9(3) |
| C3 | 52(4) | 34(3) | 70(6) | -5(3) | -10(4) | -1(3) |
| C5 | 48(4) | 57(4) | 68(6) | -22(4) | 12(4) | -11(3) |
| C25 | 38(4) | 59(4) | 82(7) | -24(4) | 10(4) | 10(3) |
| C24 | 40(4) | 70(5) | 55(5) | -9(4) | 13(3) | 3(3) |
| C19 | 37(3) | 57(4) | 81(6) | 20(4) | 12(4) | -8(3) |
| C16 | 55(4) | 50(4) | 66(6) | -11(4) | 28(4) | -8(3) |
| C15 | 47(4) | 64(5) | 89(7) | -12(5) | 40(4) | -1(4) |
| C4 | 54(4) | 33(3) | 90(7) | -1(4) | -6(4) | -6(3) |
| C26 | 34(3) | 39(3) | 91(7) | 11(4) | 5(4) | 9(3) |
| C18 | 71(6) | 51(5) | 121(10) | 9(5) | 0(6) | 10(4) |
| C13 | 44(4) | 56(4) | 88(7) | -11(4) | 10(4) | 12(3) |
| C6 | 62(5) | 85(6) | 49(5) | -23(4) | 17(4) | -33(4) |
| C14 | 46(4) | 59(5) | 92(7) | -21(5) | 28(4) | 1(4) |
| C7 | 61(5) | 67(5) | 45(5) | -17(4) | 18(4) | -18(4) |
| C8 | 51(5) | 102(7) | 76(6) | 16(5) | -14(4) | -19(5) |
| C20 | 95(9) | 250(20) | 108(9) | 27(11) | 56(8) | -36(11) |
| C29 | 82(11) | 113(13) | 75(12) | 34(10) | 29(8) | -7(9) |
| C28 | 148(10) | 110(8) | 80(8) | -33(6) | 57(7) | -22(7) |
| C30 | 141(16) | 80(9) | 71(13) | -25(9) | 51(12) | 8(10) |
| C9 | 128(12) | 221(17) | 86(9) | 53(10) | -51(8) | -61(12) |

Table 4 Bond Lengths for MJP091\_150K.

| Atom | Atom | Length/Å |  | Atom | Atom | Length/Å |
| --- | --- | --- | --- | --- | --- | --- |
| Nb3 | O1 | 1.902(4) |  | C22 | C23 | 1.380(11) |
| Nb3 | O81 | 1.904(4) |  | C22 | C27 | 1.391(10) |
| Nb3 | O12 | 1.887(4) |  | C2 | C1 | 1.503(8) |
| Nb3 | O14 | 1.896(4) |  | C2 | C3 | 1.371(10) |
| Nb3 | O18 | 2.152(4) |  | C2 | C7 | 1.393(11) |
| Nb3 | O31 | 2.196(4) |  | C31 | C32 | 1.479(8) |
| Nb2 | O1 | 1.903(4) |  | C32 | C33 | 1.387(9) |
| Nb2 | O4 | 1.901(4) |  | C32 | C37 | 1.390(8) |
| Nb2 | O6 | 1.904(4) |  | C33 | C34 | 1.386(9) |
| Nb2 | O161 | 2.171(4) |  | C34 | C35 | 1.403(10) |
| Nb2 | O9 | 1.896(4) |  | C11 | C10 | 1.488(9) |
| Nb2 | O7 | 2.152(5) |  | C11 | C12 | 1.391(11) |
| Nb1 | O6 | 1.907(4) |  | C11 | C16 | 1.380(11) |
| Nb1 | O8 | 1.888(4) |  | C37 | C36 | 1.398(9) |
| Nb1 | O10 | 1.892(5) |  | C36 | C35 | 1.381(11) |
| Nb1 | O5 | 2.161(4) |  | C23 | C24 | 1.390(10) |
| Nb1 | O111 | 2.162(5) |  | C27 | C26 | 1.385(11) |
| Nb1 | O17 | 1.893(5) |  | C12 | C13 | 1.397(11) |
| Nb4 | O41 | 1.911(4) |  | C17 | C18 | 1.458(12) |
| Nb4 | O10 | 1.914(4) |  | C3 | C4 | 1.391(10) |
| Nb4 | O14 | 1.903(4) |  | C5 | C4 | 1.358(13) |
| Nb4 | O131 | 2.181(5) |  | C5 | C6 | 1.360(13) |
| Nb4 | O15 | 2.151(4) |  | C25 | C24 | 1.349(12) |
| Nb4 | O2 | 1.876(5) |  | C25 | C26 | 1.370(13) |
| O12 | C19 | 1.398(8) |  | C19 | C20 | 1.421(18) |
| O16 | C31 | 1.250(8) |  | C16 | C15 | 1.396(11) |
| O18 | C21 | 1.259(7) |  | C15 | C14 | 1.368(13) |
| O9 | C17 | 1.413(8) |  | C13 | C14 | 1.361(14) |
| O5 | C1 | 1.278(8) |  | C6 | C7 | 1.392(11) |
| O11 | C21 | 1.275(8) |  | N1 | C38 | 1.13(2) |
| O3 | C1 | 1.239(8) |  | C8 | C9 | 1.445(17) |
| O13 | C10 | 1.268(8) |  | C38 | C39 | 1.42(2) |
| O7 | C10 | 1.260(9) |  | C29 | C28 | 1.31(3) |
| O15 | C31 | 1.264(7) |  | C28 | C30 | 1.31(3) |
| O2 | C28 | 1.410(13) |  | C41 | C40 | 1.44(2) |
| O17 | C8 | 1.404(9) |  | C40 | N2 | 1.12(2) |
| C22 | C21 | 1.477(9) |  |  |  |  |

11-X,1-Y,1-Z

Table 5 Bond Angles for MJP091\_150K.

| Atom | Atom | Atom | Angle/˚ |  | Atom | Atom | Atom | Angle/˚ |
| --- | --- | --- | --- | --- | --- | --- | --- | --- |
| O1 | Nb3 | O81 | 95.89(18) |  | C19 | O12 | Nb3 | 144.1(4) |
| O1 | Nb3 | O18 | 172.52(18) |  | Nb3 | O14 | Nb4 | 148.4(3) |
| O1 | Nb3 | O31 | 90.19(17) |  | C31 | O16 | Nb21 | 136.4(4) |
| O81 | Nb3 | O18 | 82.17(17) |  | C21 | O18 | Nb3 | 135.7(4) |
| O81 | Nb3 | O31 | 81.93(17) |  | C17 | O9 | Nb2 | 140.1(5) |
| O12 | Nb3 | O1 | 97.70(18) |  | C1 | O5 | Nb1 | 135.6(4) |
| O12 | Nb3 | O81 | 159.14(18) |  | C21 | O11 | Nb11 | 135.8(4) |
| O12 | Nb3 | O14 | 98.89(19) |  | C1 | O3 | Nb31 | 136.0(4) |
| O12 | Nb3 | O18 | 82.31(17) |  | C10 | O13 | Nb41 | 136.7(4) |
| O12 | Nb3 | O31 | 82.28(18) |  | C10 | O7 | Nb2 | 136.5(4) |
| O14 | Nb3 | O1 | 97.86(18) |  | C31 | O15 | Nb4 | 135.9(4) |
| O14 | Nb3 | O81 | 94.83(18) |  | C28 | O2 | Nb4 | 150.2(8) |
| O14 | Nb3 | O18 | 89.51(17) |  | C8 | O17 | Nb1 | 141.0(6) |
| O14 | Nb3 | O31 | 171.61(16) |  | C23 | C22 | C21 | 120.6(6) |
| O18 | Nb3 | O31 | 82.40(16) |  | C23 | C22 | C27 | 119.7(6) |
| O1 | Nb2 | O6 | 98.50(18) |  | C27 | C22 | C21 | 119.6(7) |
| O1 | Nb2 | O161 | 89.83(18) |  | C3 | C2 | C1 | 119.6(6) |
| O1 | Nb2 | O7 | 171.81(19) |  | C3 | C2 | C7 | 119.0(6) |
| O4 | Nb2 | O1 | 94.22(17) |  | C7 | C2 | C1 | 121.3(6) |
| O4 | Nb2 | O6 | 96.65(18) |  | O18 | C21 | O11 | 124.5(6) |
| O4 | Nb2 | O161 | 82.12(17) |  | O18 | C21 | C22 | 118.0(6) |
| O4 | Nb2 | O7 | 83.02(18) |  | O11 | C21 | C22 | 117.5(5) |
| O6 | Nb2 | O161 | 171.66(19) |  | O16 | C31 | O15 | 126.0(5) |
| O6 | Nb2 | O7 | 89.49(19) |  | O16 | C31 | C32 | 117.6(5) |
| O9 | Nb2 | O1 | 97.45(19) |  | O15 | C31 | C32 | 116.4(6) |
| O9 | Nb2 | O4 | 159.06(19) |  | C33 | C32 | C31 | 120.4(5) |
| O9 | Nb2 | O6 | 98.73(19) |  | C33 | C32 | C37 | 119.6(6) |
| O9 | Nb2 | O161 | 80.60(18) |  | C37 | C32 | C31 | 120.0(6) |
| O9 | Nb2 | O7 | 83.00(19) |  | C34 | C33 | C32 | 120.4(6) |
| O7 | Nb2 | O161 | 82.17(19) |  | C33 | C34 | C35 | 119.9(7) |
| O6 | Nb1 | O5 | 171.67(19) |  | O5 | C1 | C2 | 115.0(6) |
| O6 | Nb1 | O111 | 88.54(18) |  | O3 | C1 | O5 | 126.2(5) |
| O8 | Nb1 | O6 | 96.62(18) |  | O3 | C1 | C2 | 118.8(6) |
| O8 | Nb1 | O10 | 94.82(18) |  | C12 | C11 | C10 | 119.0(7) |
| O8 | Nb1 | O5 | 82.97(17) |  | C16 | C11 | C10 | 121.1(7) |
| O8 | Nb1 | O111 | 81.64(17) |  | C16 | C11 | C12 | 119.8(7) |
| O8 | Nb1 | O17 | 158.0(2) |  | C32 | C37 | C36 | 120.5(6) |
| O10 | Nb1 | O6 | 96.98(18) |  | C35 | C36 | C37 | 119.6(6) |
| O10 | Nb1 | O5 | 91.35(18) |  | C36 | C35 | C34 | 120.0(6) |
| O10 | Nb1 | O111 | 173.78(17) |  | O13 | C10 | C11 | 117.9(6) |
| O10 | Nb1 | O17 | 100.6(2) |  | O7 | C10 | O13 | 124.2(6) |
| O5 | Nb1 | O111 | 83.16(18) |  | O7 | C10 | C11 | 117.9(6) |
| O17 | Nb1 | O6 | 97.0(2) |  | C22 | C23 | C24 | 119.4(7) |
| O17 | Nb1 | O5 | 81.03(19) |  | C26 | C27 | C22 | 119.6(8) |
| O17 | Nb1 | O111 | 81.5(2) |  | C11 | C12 | C13 | 119.5(8) |
| O41 | Nb4 | O10 | 94.89(18) |  | O9 | C17 | C18 | 113.1(7) |
| O41 | Nb4 | O131 | 82.61(17) |  | C2 | C3 | C4 | 120.1(8) |
| O41 | Nb4 | O15 | 82.18(17) |  | C4 | C5 | C6 | 119.8(7) |
| O10 | Nb4 | O131 | 171.00(17) |  | C24 | C25 | C26 | 120.8(7) |
| O10 | Nb4 | O15 | 88.38(18) |  | C25 | C24 | C23 | 120.6(8) |
| O14 | Nb4 | O41 | 94.13(18) |  | O12 | C19 | C20 | 112.3(9) |
| O14 | Nb4 | O10 | 97.25(17) |  | C11 | C16 | C15 | 119.3(8) |
| O14 | Nb4 | O131 | 91.56(18) |  | C14 | C15 | C16 | 120.8(9) |
| O14 | Nb4 | O15 | 173.53(18) |  | C5 | C4 | C3 | 120.8(8) |
| O15 | Nb4 | O131 | 82.72(18) |  | C25 | C26 | C27 | 119.9(7) |
| O2 | Nb4 | O41 | 161.37(18) |  | C14 | C13 | C12 | 120.4(9) |
| O2 | Nb4 | O10 | 97.7(2) |  | C5 | C6 | C7 | 120.7(9) |
| O2 | Nb4 | O14 | 97.85(19) |  | C13 | C14 | C15 | 120.1(7) |
| O2 | Nb4 | O131 | 82.8(2) |  | C6 | C7 | C2 | 119.5(8) |
| O2 | Nb4 | O15 | 84.48(18) |  | O17 | C8 | C9 | 111.7(10) |
| Nb3 | O1 | Nb2 | 146.9(2) |  | N1 | C38 | C39 | 179(2) |
| Nb2 | O4 | Nb41 | 147.9(2) |  | C29 | C28 | O2 | 107.3(17) |
| Nb2 | O6 | Nb1 | 144.8(2) |  | C30 | C28 | O2 | 124.7(16) |
| Nb1 | O8 | Nb31 | 149.3(2) |  | N2 | C40 | C41 | 175(3) |
| Nb1 | O10 | Nb4 | 147.4(3) |  |  |  |  |  |

11-X,1-Y,1-Z

Table 6 Torsion Angles for MJP091\_150K.

| A | B | C | D | Angle/˚ |  | A | B | C | D | Angle/˚ |
| --- | --- | --- | --- | --- | --- | --- | --- | --- | --- | --- |
| Nb3 | O12 | C19 | C20 | 141.3(11) |  | O131 | Nb4 | O2 | C28 | 22.3(13) |
| Nb3 | O18 | C21 | O11 | -21.6(10) |  | O7 | Nb2 | O9 | C17 | 65.4(7) |
| Nb3 | O18 | C21 | C22 | 160.0(4) |  | O15 | Nb4 | O2 | C28 | -61.0(13) |
| Nb31 | O3 | C1 | O5 | 1.6(10) |  | O15 | C31 | C32 | C33 | -13.2(9) |
| Nb31 | O3 | C1 | C2 | -178.0(4) |  | O15 | C31 | C32 | C37 | 164.6(6) |
| Nb21 | O16 | C31 | O15 | 6.0(10) |  | O17 | Nb1 | O8 | Nb31 | 3.0(9) |
| Nb21 | O16 | C31 | C32 | -176.2(4) |  | O17 | Nb1 | O10 | Nb4 | -173.5(4) |
| Nb2 | O9 | C17 | C18 | -161.2(7) |  | C22 | C23 | C24 | C25 | 3.1(11) |
| Nb2 | O7 | C10 | O13 | -15.8(10) |  | C22 | C27 | C26 | C25 | -0.4(11) |
| Nb2 | O7 | C10 | C11 | 161.2(4) |  | C2 | C3 | C4 | C5 | -2.4(13) |
| Nb1 | O5 | C1 | O3 | 1.0(10) |  | C21 | C22 | C23 | C24 | 179.7(6) |
| Nb1 | O5 | C1 | C2 | -179.3(4) |  | C21 | C22 | C27 | C26 | 179.0(6) |
| Nb11 | O11 | C21 | O18 | 17.4(10) |  | C31 | C32 | C33 | C34 | 176.6(6) |
| Nb11 | O11 | C21 | C22 | -164.3(4) |  | C31 | C32 | C37 | C36 | -176.2(6) |
| Nb1 | O17 | C8 | C9 | -169.8(11) |  | C32 | C33 | C34 | C35 | -0.3(11) |
| Nb41 | O13 | C10 | O7 | 9.8(10) |  | C32 | C37 | C36 | C35 | -0.6(11) |
| Nb41 | O13 | C10 | C11 | -167.2(4) |  | C33 | C32 | C37 | C36 | 1.6(10) |
| Nb4 | O15 | C31 | O16 | 1.3(10) |  | C33 | C34 | C35 | C36 | 1.3(12) |
| Nb4 | O15 | C31 | C32 | -176.5(4) |  | C1 | C2 | C3 | C4 | -179.4(7) |
| Nb4 | O2 | C28 | C29 | 107.3(19) |  | C1 | C2 | C7 | C6 | -179.7(7) |
| Nb4 | O2 | C28 | C30 | 66(3) |  | C11 | C12 | C13 | C14 | 0.1(12) |
| O1 | Nb3 | O12 | C19 | 145.0(8) |  | C11 | C16 | C15 | C14 | -3.2(12) |
| O1 | Nb3 | O14 | Nb4 | -85.1(5) |  | C37 | C32 | C33 | C34 | -1.2(10) |
| O1 | Nb2 | O9 | C17 | -106.4(7) |  | C37 | C36 | C35 | C34 | -0.8(12) |
| O4 | Nb2 | O9 | C17 | 16.9(10) |  | C10 | C11 | C12 | C13 | -176.5(7) |
| O41 | Nb4 | O2 | C28 | -16.6(16) |  | C10 | C11 | C16 | C15 | 178.0(7) |
| O6 | Nb2 | O9 | C17 | 153.8(7) |  | C23 | C22 | C21 | O18 | 4.6(9) |
| O6 | Nb1 | O8 | Nb31 | 131.0(5) |  | C23 | C22 | C21 | O11 | -173.9(6) |
| O6 | Nb1 | O10 | Nb4 | 88.0(4) |  | C23 | C22 | C27 | C26 | 0.1(10) |
| O6 | Nb1 | O17 | C8 | -133.2(10) |  | C27 | C22 | C21 | O18 | -174.3(6) |
| O81 | Nb3 | O12 | C19 | 14.8(11) |  | C27 | C22 | C21 | O11 | 7.3(9) |
| O81 | Nb3 | O14 | Nb4 | 11.5(5) |  | C27 | C22 | C23 | C24 | -1.4(10) |
| O8 | Nb1 | O10 | Nb4 | -9.3(4) |  | C12 | C11 | C10 | O13 | 169.0(6) |
| O8 | Nb1 | O17 | C8 | -5.3(14) |  | C12 | C11 | C10 | O7 | -8.3(9) |
| O10 | Nb1 | O8 | Nb31 | -131.4(5) |  | C12 | C11 | C16 | C15 | 0.7(11) |
| O10 | Nb1 | O17 | C8 | 128.3(10) |  | C12 | C13 | C14 | C15 | -2.6(13) |
| O10 | Nb4 | O2 | C28 | -148.6(13) |  | C3 | C2 | C1 | O5 | 178.1(6) |
| O12 | Nb3 | O14 | Nb4 | 175.7(4) |  | C3 | C2 | C1 | O3 | -2.2(9) |
| O14 | Nb3 | O12 | C19 | -115.7(8) |  | C3 | C2 | C7 | C6 | -2.1(12) |
| O14 | Nb4 | O2 | C28 | 112.9(13) |  | C5 | C6 | C7 | C2 | 0.7(13) |
| O161 | Nb2 | O9 | C17 | -17.8(7) |  | C24 | C25 | C26 | C27 | 2.1(12) |
| O16 | C31 | C32 | C33 | 168.8(6) |  | C16 | C11 | C10 | O13 | -8.3(9) |
| O16 | C31 | C32 | C37 | -13.4(9) |  | C16 | C11 | C10 | O7 | 174.4(6) |
| O18 | Nb3 | O12 | C19 | -27.4(8) |  | C16 | C11 | C12 | C13 | 0.8(11) |
| O18 | Nb3 | O14 | Nb4 | 93.6(5) |  | C16 | C15 | C14 | C13 | 4.1(13) |
| O5 | Nb1 | O8 | Nb31 | -40.6(5) |  | C4 | C5 | C6 | C7 | -0.1(13) |
| O5 | Nb1 | O10 | Nb4 | -92.3(4) |  | C26 | C25 | C24 | C23 | -3.5(12) |
| O5 | Nb1 | O17 | C8 | 38.6(10) |  | C6 | C5 | C4 | C3 | 1.0(14) |
| O111 | Nb1 | O8 | Nb31 | 43.5(5) |  | C7 | C2 | C1 | O5 | -4.3(9) |
| O111 | Nb1 | O17 | C8 | -45.7(10) |  | C7 | C2 | C1 | O3 | 175.4(6) |
| O31 | Nb3 | O12 | C19 | 55.9(8) |  | C7 | C2 | C3 | C4 | 2.9(11) |

11-X,1-Y,1-Z

Table 7 Hydrogen Atom Coordinates (Å×104) and Isotropic Displacement Parameters (Å2×103) for MJP091\_150K.

| Atom | *x* | *y* | *z* | U(eq) |
| --- | --- | --- | --- | --- |
| H33 | 3736.81 | 3346.89 | 738.65 | 46 |
| H34 | 3096.41 | 2644.88 | -282.56 | 53 |
| H37 | 4656.09 | 2518.62 | 3732.35 | 49 |
| H36 | 4031.23 | 1808.18 | 2706.91 | 55 |
| H35 | 3266.95 | 1869.51 | 693.81 | 58 |
| H23 | 8938.04 | 4976.75 | 3250.88 | 55 |
| H27 | 8983.22 | 4143.05 | 6290.41 | 61 |
| H12 | 2109.99 | 6928.86 | 5088.99 | 61 |
| H17A | 4616.02 | 7362.1 | 4130.88 | 66 |
| H17B | 3768.32 | 7257.6 | 4729.68 | 66 |
| H3 | 2132.59 | 3063.92 | 4178.71 | 65 |
| H5 | 790.47 | 2435.88 | 1103.42 | 69 |
| H25 | 10670.25 | 3851.96 | 3977.79 | 72 |
| H24 | 10137.59 | 4518.35 | 2797.6 | 65 |
| H19A | 8398.03 | 5953.03 | 3340.65 | 70 |
| H19B | 8041.13 | 6504.03 | 3269.15 | 70 |
| H16 | 2137.24 | 6158.25 | 8222.91 | 66 |
| H15 | 966.23 | 6652.02 | 8642.94 | 76 |
| H4 | 1398.22 | 2386.14 | 3154.75 | 73 |
| H26 | 10143.8 | 3675.11 | 5759.23 | 66 |
| H18A | 3674.43 | 7456.82 | 2247.88 | 125 |
| H18B | 3517.32 | 7864.39 | 3232.17 | 125 |
| H18C | 2848.03 | 7406.99 | 2944.32 | 125 |
| H13 | 982.74 | 7437.06 | 5591.05 | 75 |
| H6 | 953.86 | 3148.67 | 25.96 | 77 |
| H14 | 465.23 | 7316.63 | 7398.13 | 76 |
| H7 | 1717.07 | 3829.14 | 1005.74 | 68 |
| H8A | 1119.13 | 5226.96 | 1447.56 | 96 |
| H8B | 1427.56 | 4702.22 | 1002.55 | 96 |
| H39A | 2271.33 | 6466.76 | 773.68 | 98 |
| H39B | 2301.25 | 6058.19 | 1822.97 | 98 |
| H39C | 2520.81 | 6623.31 | 2183.88 | 98 |
| H20A | 7781.51 | 5892.03 | 1253.77 | 220 |
| H20B | 8649.44 | 6230.66 | 1596.51 | 220 |
| H20C | 7686.61 | 6481.03 | 1277.71 | 220 |
| H29A | 4930.08 | 3762.84 | -208.48 | 132 |
| H29B | 4770.28 | 4227.94 | -1062.38 | 132 |
| H29C | 5410.18 | 3806.54 | -1338.78 | 132 |
| H28A | 6301.67 | 3982.94 | 354.17 | 130 |
| H28B | 6140.37 | 4453.04 | -508.63 | 130 |
| H28C | 6496.27 | 4196.74 | 296.37 | 130 |
| H28D | 5768.87 | 4436.44 | -734.84 | 130 |
| H30A | 5959.03 | 3533.92 | 188.3 | 140 |
| H30B | 5816.88 | 3720.12 | -1201.55 | 140 |
| H30C | 5003.4 | 3727.92 | -472.84 | 140 |
| H9A | 940.76 | 4997.95 | -770.84 | 230 |
| H9B | 1900.95 | 5252.33 | -595.28 | 230 |
| H9C | 1065.1 | 5558.66 | -311.75 | 230 |
| H41A | 2216.53 | 6550.31 | 1377.54 | 88 |
| H41B | 2030.12 | 6059.93 | 2097.56 | 88 |
| H41C | 2349.33 | 6554.2 | 2840.63 | 88 |

Table 8 Atomic Occupancy for MJP091\_150K.

| Atom | *Occupancy* |  | Atom | *Occupancy* |  | Atom | *Occupancy* |
| --- | --- | --- | --- | --- | --- | --- | --- |
| N1 | 0.5 |  | C38 | 0.5 |  | C39 | 0.5 |
| H39A | 0.5 |  | H39B | 0.5 |  | H39C | 0.5 |
| C29 | 0.5 |  | H29A | 0.5 |  | H29B | 0.5 |
| H29C | 0.5 |  | H28A | 0.5 |  | H28B | 0.5 |
| H28C | 0.5 |  | H28D | 0.5 |  | C30 | 0.5 |
| H30A | 0.5 |  | H30B | 0.5 |  | H30C | 0.5 |
| C41 | 0.4 |  | H41A | 0.4 |  | H41B | 0.4 |
| H41C | 0.4 |  | C40 | 0.4 |  | N2 | 0.4 |

Experimental

Single crystals of C75.6H85.4N1.8Nb8O36
[MJP091\_150K]
were
[?].
A suitable crystal was selected and
[]
on a
STOE STADIVARI
diffractometer. The crystal was kept at 150 K during data collection.
Using Olex2 [1], the structure was solved with the
SHELXT
[2] structure solution program using
Intrinsic Phasing
and refined with the
SHELXL
[3] refinement package using
Least Squares
minimisation.

1. Dolomanov, O.V., Bourhis, L.J., Gildea, R.J, Howard, J.A.K. & Puschmann, H.
   (2009), J. Appl. Cryst. 42, 339-341.
2. Sheldrick, G.M. (2015). Acta Cryst. A71, 3-8.
3. Sheldrick, G.M. (2015). Acta Cryst. C71, 3-8.

Crystal structure determination of
[MJP091\_150K]

**Crystal Data**
for C75.6H85.4N1.8Nb8O36 (*M*=2338.53 g/mol):
monoclinic, space group P21/c (no. 14),
*a* = 15.2795(5) Å, *b* = 27.0474(6) Å, *c* = 11.1015(4) Å, *β* = 100.348(3)°,
*V*= 4513.3(2) Å3,
*Z* = 2,
*T* = 150 K,
μ(GaKα) = 5.871 mm-1,
*Dcalc* = 1.721 g/cm3,
40957 reflections measured (5.686° ≤ 2Θ ≤ 111.384°),
8683 unique (*R*int = 0.0873, Rsigma = 0.0497) which were used in all calculations.
The final *R*1 was 0.0545
(I > 2σ(I)) and *wR*2 was 0.1473 (all data).

Refinement model description

Number of restraints - 93,
number of constraints - unknown.

Details:

```
1. Fixed Uiso
```

This report has been created with Olex2, compiled on
2023.08.24 svn.re1ec1418 for OlexSys. Please
let us know
if there are any errors or if you would like to have additional features.
