# Supplementary material for: Mechanism‐Guided Precision Hydrolysis of Early Transition Metals to Access (Mixed‐Metal) Oxo Clusters
Source: Angew Chem Int Ed Engl. 2026 Feb 24;65(15):e25769. doi: 10.1002/anie.202525769 (PMC13053926; doi:10.1002/anie.202525769)

checkCIF/PLATON report

Structure factors have been supplied for datablock(s) mjp089-2_150k

THIS REPORT IS FOR GUIDANCE ONLY. IF USED AS PART OF A REVIEW PROCEDURE FOR PUBLICATION, IT SHOULD NOT REPLACE THE EXPERTISE OF AN EXPERIENCED CRYSTALLOGRAPHIC REFEREE.

No syntax errors found. CIF dictionary Interpreting this report

Datablock: mjp089-2_150k

```
Bond precision:   C-C = 0.0403 A          Wavelength=1.34143

Cell:            a=15.1819(5)     b=27.0298(7)        c=11.1441(4)
                 alpha=90         beta=100.527(3)     gamma=90
Temperature:     150 K

                Calculated                     Reported
Volume          4496.2(3)                      4496.2(3)
Space group     P 21/c                         P 1 21/c 1
Hall group      -P 2ybc                        -P 2ybc
Moiety formula  C72 H80 O36 Ta8, C2 H3 N       C72 H80 O36 Ta8, C2 H3 N
Sum formula     C74 H83 N O36 Ta8              C74 H83 N O36 Ta8
Mr              3010.02                        3010.01
Dx,g cm-3       2.223                          2.223
Z               2                              2
Mu (mm-1)       12.372                         12.373
F000            2812.0                         2812.0
F000'           2689.26
h,k,lmax        18,32,13                       18,32,13
Nref            8277                           8235
Tmin,Tmax       0.233,0.372                    0.000,0.003
Tmin'           0.142

Correction method= # Reported T Limits: Tmin=0.000 Tmax=0.003
AbsCorr = MULTI-SCAN

Data completeness= 0.995           Theta(max)= 53.999

                                                       wR2(reflections)=
R(reflections)= 0.0918( 4593)                          0.2617( 8235)

S = 0.979                  Npar= 555
```

The following ALERTS were generated. Each ALERT has the format
test-name_ALERT_alert-type_alert-level.
Click on the hyperlinks for more details of the test.

Alert level B

RINTA01_ALERT_3_B The value of Rint is greater than 0.18
Rint given 0.201

Author Response: all the crystals attempted were weakly diffracting at high angles and suffered of radiation damage in the metaljet beam

PLAT020_ALERT_3_B The Value of Rint is Greater Than 0.12 .... 0.201 Report

Author Response: all the crystals attempted were weakly diffracting at high angles and suffered of radiation damage in the metaljet beam

PLAT342_ALERT_3_B Low Bond Precision on C-C Bonds .... 0.04031 Ang.

Author Response: all the crystals attempted were weakly diffracting at high angles and suffered of radiation damage in the metaljet beam

PLAT971_ALERT_2_B Check Calcd Resid. Dens. 0.90Ang From Ta4 3.42 eA-3

Author Response: this is very likely and absorption effect due to the presence of very heavy atoms such as Ta. The absorption correction has been done but it could not correct for everything.

PLAT971_ALERT_2_B Check Calcd Resid. Dens. 0.93Ang From Ta2 3.01 eA-3

Author Response: this is very likely and absorption effect due to the presence of very heavy atoms such as Ta. The absorption correction has been done but it could not correct for everything.

PLAT971_ALERT_2_B Check Calcd Resid. Dens. 0.90Ang From Ta3 2.92 eA-3

Author Response: this is very likely and absorption effect due to the presence of very heavy atoms such as Ta. The absorption correction has been done but it could not correct for everything.

PLAT971_ALERT_2_B Check Calcd Resid. Dens. 1.01Ang From Ta1 2.83 eA-3

Author Response: this is very likely and absorption effect due to the presence of very heavy atoms such as Ta. The absorption correction has been done but it could not correct for everything.

```
PLAT971_ALERT_2_B Check Calcd Resid. Dens.  0.99Ang From Ta2            2.67 eA-3
```

Author Response: this is very likely and absorption effect due to the presence of very heavy atoms such as Ta. The absorption correction has been done but it could not correct for everything.

```
PLAT972_ALERT_2_B Check Calcd Resid. Dens.  0.94Ang From Ta3           -2.98 eA-3
```

Author Response: this is very likely and absorption effect due to the presence of very heavy atoms such as Ta. The absorption correction has been done but it could not correct for everything.

```
PLAT972_ALERT_2_B Check Calcd Resid. Dens.  0.96Ang From Ta4           -2.91 eA-3
```

Author Response: this is very likely and absorption effect due to the presence of very heavy atoms such as Ta. The absorption correction has been done but it could not correct for everything.

```
PLAT972_ALERT_2_B Check Calcd Resid. Dens.  1.01Ang From Ta4           -2.75 eA-3
```

Author Response: this is very likely and absorption effect due to the presence of very heavy atoms such as Ta. The absorption correction has been done but it could not correct for everything.

```
PLAT972_ALERT_2_B Check Calcd Resid. Dens.  0.97Ang From Ta1           -2.67 eA-3
```

Author Response: this is very likely and absorption effect due to the presence of very heavy atoms such as Ta. The absorption correction has been done but it could not correct for everything.

```
PLAT972_ALERT_2_B Check Calcd Resid. Dens.  0.94Ang From Ta3           -2.65 eA-3
```

Author Response: this is very likely and absorption effect due to the presence of very heavy atoms such as Ta. The absorption correction has been done but it could not correct for everything.

```
PLAT972_ALERT_2_B Check Calcd Resid. Dens.  0.98Ang From Ta1           -2.57 eA-3
```

Author Response: this is very likely and absorption effect due to the presence of very heavy atoms such as Ta. The absorption correction has been done but it could not correct for everything.

```
PLAT972_ALERT_2_B Check Calcd Resid. Dens.  1.00Ang From Ta2           -2.53 eA-3
```

Author Response: this is very likely and absorption effect due to the presence of very heavy atoms such as Ta. The absorption correction has been done but it could not correct for everything.

Alert level C

```
PLAT084_ALERT_3_C High wR2 Value (i.e. > 0.25) ..................        0.26 Report
PLAT220_ALERT_2_C NonSolvent   Resd 1  C   Ueq(max)/Ueq(min) Range        4.1 Ratio
PLAT222_ALERT_3_C NonSolvent Resd 1  H   Uiso(max)/Uiso(min) Range        4.5 Ratio
PLAT234_ALERT_4_C Large Hirshfeld Difference Ta4      --O3       .       0.18 Ang.
PLAT234_ALERT_4_C Large Hirshfeld Difference Ta4      --O8       .       0.16 Ang.
PLAT234_ALERT_4_C Large Hirshfeld Difference O6       --C28      .       0.19 Ang.
PLAT234_ALERT_4_C Large Hirshfeld Difference O10      --C3       .       0.20 Ang.
PLAT234_ALERT_4_C Large Hirshfeld Difference O14      --C12      .       0.19 Ang.
PLAT234_ALERT_4_C Large Hirshfeld Difference C13      --C14      .       0.22 Ang.
PLAT242_ALERT_2_C Low    'MainMol' Ueq as Compared to Neighbors of         C1 Check
PLAT242_ALERT_2_C Low    'MainMol' Ueq as Compared to Neighbors of        C19 Check
PLAT242_ALERT_2_C Low    'MainMol' Ueq as Compared to Neighbors of        C35 Check
PLAT332_ALERT_2_C Large Phenyl C-C Range     C4        -C9       .       0.20 Ang.
PLAT360_ALERT_2_C Short  C(sp3)-C(sp3) Bond  C1       - C2       .       1.41 Ang.
PLAT360_ALERT_2_C Short  C(sp3)-C(sp3) Bond  C10      - C11      .       1.43 Ang.
PLAT360_ALERT_2_C Short  C(sp3)-C(sp3) Bond  C19      - C20      .       1.42 Ang.
PLAT360_ALERT_2_C Short  C(sp3)-C(sp3) Bond  C35      - C36      .       1.43 Ang.
PLAT369_ALERT_2_C Long   C(sp2)-C(sp2) Bond  C28      - C29      .       1.53 Ang.
PLAT906_ALERT_3_C Large K Value in the Analysis of Variance ......     18.652 Check
PLAT906_ALERT_3_C Large K Value in the Analysis of Variance ......      2.900 Check
PLAT911_ALERT_3_C Missing FCF Refl Between Thmin & STh/L=    0.600         40 Report
PLAT971_ALERT_2_C Check Calcd Resid. Dens.  0.92Ang From Ta4            2.48 eA-3
```

Author Response: this is very likely and absorption effect due to the presence of very heavy atoms such as Ta. The absorption correction has been done but it could not correct for everything.

```
PLAT971_ALERT_2_C Check Calcd Resid. Dens.  0.95Ang From Ta1            2.46 eA-3
```

Author Response: this is very likely and absorption effect due to the presence of very heavy atoms such as Ta. The absorption correction has been done but it could not correct for everything.

```
PLAT971_ALERT_2_C Check Calcd Resid. Dens.  0.90Ang From Ta3            2.44 eA-3
```

Author Response: this is very likely and absorption effect due to the presence of very heavy atoms such as Ta. The absorption correction has been done but it could not correct for everything.

```
PLAT971_ALERT_2_C Check Calcd Resid. Dens.  0.92Ang From O4             1.72 eA-3
```

Author Response: this is very likely and absorption effect due to the presence of very heavy atoms such as Ta. The absorption correction has been done but it could not correct for everything.

```
PLAT971_ALERT_2_C Check Calcd Resid. Dens.  1.01Ang From Ta3            1.68 eA-3
```

Author Response: this is very likely and absorption effect due to the presence of very heavy atoms such as Ta. The absorption correction has been done but it could not correct for everything.

```
PLAT971_ALERT_2_C Check Calcd Resid. Dens.  1.00Ang From Ta4            1.67 eA-3
```

Author Response: this is very likely and absorption effect due to the presence of very heavy atoms such as Ta. The absorption correction has been done but it could not correct for everything.

```
PLAT971_ALERT_2_C Check Calcd Resid. Dens.  0.96Ang From Ta4            1.65 eA-3
```

Author Response: this is very likely and absorption effect due to the presence of very heavy atoms such as Ta. The absorption correction has been done but it could not correct for everything.

```
PLAT971_ALERT_2_C Check Calcd Resid. Dens.  0.95Ang From Ta3            1.54 eA-3
```

Author Response: this is very likely and absorption effect due to the presence of very heavy atoms such as Ta. The absorption correction has been done but it could not correct for everything.

```
PLAT972_ALERT_2_C Check Calcd Resid. Dens.  1.04Ang From Ta2           -2.38 eA-3
```

Author Response: this is very likely and absorption effect due to the presence of very heavy atoms such as Ta. The absorption correction has been done but it could not correct for everything.

```
PLAT972_ALERT_2_C Check Calcd Resid. Dens.  0.97Ang From O18           -1.97 eA-3
```

Author Response: this is very likely and absorption effect due to the presence of very heavy atoms such as Ta. The absorption correction has been done but it could not correct for everything.

```
PLAT972_ALERT_2_C Check Calcd Resid. Dens.  1.24Ang From O6           -1.80 eA-3
```

Author Response: this is very likely and absorption effect due to the presence of very heavy atoms such as Ta. The absorption correction has been done but it could not correct for everything.

```
PLAT972_ALERT_2_C Check Calcd Resid. Dens.  1.39Ang From O13          -1.69 eA-3
```

Author Response: this is very likely and absorption effect due to the presence of very heavy atoms such as Ta. The absorption correction has been done but it could not correct for everything.

```
PLAT972_ALERT_2_C Check Calcd Resid. Dens.  1.27Ang From O14          -1.67 eA-3
```

Author Response: this is very likely and absorption effect due to the presence of very heavy atoms such as Ta. The absorption correction has been done but it could not correct for everything.

```
PLAT972_ALERT_2_C Check Calcd Resid. Dens.  1.27Ang From O11          -1.56 eA-3
```

Author Response: this is very likely and absorption effect due to the presence of very heavy atoms such as Ta. The absorption correction has been done but it could not correct for everything.

```
PLAT972_ALERT_2_C Check Calcd Resid. Dens.  1.05Ang From O8           -1.55 eA-3
```

Author Response: this is very likely and absorption effect due to the presence of very heavy atoms such as Ta. The absorption correction has been done but it could not correct for everything.

```
PLAT973_ALERT_2_C Check Calcd Positive Resid. Density on       Ta2       1.43 eA-3
PLAT973_ALERT_2_C Check Calcd Positive Resid. Density on       Ta4       1.32 eA-3
PLAT975_ALERT_2_C Check Calcd Resid. Dens.  1.07Ang From O11     .       1.10 eA-3
PLAT976_ALERT_2_C Check Calcd Resid. Dens.  1.05Ang From O1      .      -1.12 eA-3
```

Author Response: this is very likely and absorption effect due to the presence of very heavy atoms such as Ta. The absorption correction has been done but it could not correct for everything.

```
PLAT977_ALERT_2_C Check Negative Difference Density on H1A       .      -0.49 eA-3
PLAT977_ALERT_2_C Check Negative Difference Density on H1B       .      -0.57 eA-3
PLAT977_ALERT_2_C Check Negative Difference Density on H10A      .      -0.38 eA-3
PLAT977_ALERT_2_C Check Negative Difference Density on H35A      .      -0.34 eA-3
PLAT977_ALERT_2_C Check Negative Difference Density on H35B      .      -0.40 eA-3
PLAT977_ALERT_2_C Check Negative Difference Density on H36A      .      -0.47 eA-3
PLAT977_ALERT_2_C Check Negative Difference Density on H38A      .      -0.36 eA-3
```

Alert level G

```
ABSMU01_ALERT_1_G  Calculation of _exptl_absorpt_correction_mu
                not performed for this radiation type.
PLAT002_ALERT_2_G Number of Distance or Angle Restraints on AtSite         20 Note
PLAT003_ALERT_2_G Number of Uiso or Uij Restrained non-H Atoms ...         31 Report
PLAT072_ALERT_2_G SHELXL First  Parameter in WGHT  Unusually Large       0.16 Report
PLAT176_ALERT_4_G The CIF-Embedded .res File Contains SADI Records          3 Report
PLAT178_ALERT_4_G The CIF-Embedded .res File Contains SIMU Records          7 Report
PLAT186_ALERT_4_G The CIF-Embedded .res File Contains ISOR Records          2 Report
PLAT187_ALERT_4_G The CIF-Embedded .res File Contains RIGU Records          7 Report
PLAT300_ALERT_4_G Atom Site Occupancy of N019       Constrained at        0.5 Check
PLAT300_ALERT_4_G Atom Site Occupancy of C37        Constrained at        0.5 Check
PLAT300_ALERT_4_G Atom Site Occupancy of C38        Constrained at        0.5 Check
PLAT300_ALERT_4_G Atom Site Occupancy of H38A       Constrained at        0.5 Check
PLAT300_ALERT_4_G Atom Site Occupancy of H38B       Constrained at        0.5 Check
PLAT300_ALERT_4_G Atom Site Occupancy of H38C       Constrained at        0.5 Check
PLAT302_ALERT_4_G Anion/Solvent/Minor-Residue Disorder (Resd  2  )       100% Note
PLAT720_ALERT_4_G Number of Unusual/Non-Standard Labels ..........          1 Note
PLAT722_ALERT_1_G Angle   Calc      108.00, Rep      109.50 Dev...       1.50 Degree
              H36A    -C36     -H36C       1_555  1_555  1_555     #  192 Check
PLAT794_ALERT_5_G Tentative Bond Valency for Ta1       (V)       .       5.04 Info
PLAT794_ALERT_5_G Tentative Bond Valency for Ta3       (V)       .       5.14 Info
PLAT794_ALERT_5_G Tentative Bond Valency for Ta4       (V)       .       5.34 Info
PLAT802_ALERT_4_G CIF Input Record(s) with more than 80 Characters          1 Info
PLAT860_ALERT_3_G Number of Least-Squares Restraints ............         360 Note
PLAT908_ALERT_2_G Max. Perc. Data with I > 2*s(I) per Res.Shell .      73.51% Note
PLAT910_ALERT_3_G Missing # of FCF Reflection(s) Below Theta(Min).          1 Note
PLAT912_ALERT_4_G Missing # of FCF Reflections Above STh/L=  0.600          2 Note
PLAT913_ALERT_3_G Missing # of Very Strong Reflections in FCF ....          1 Note
PLAT933_ALERT_2_G Number of HKL-OMIT Records in Embedded .res File         12 Note
PLAT978_ALERT_2_G Number C-C Bonds with Positive Residual Density.          0 Info
```

```
   0 ALERT level A = Most likely a serious problem - resolve or explain
  15 ALERT level B = A potentially serious problem, consider carefully
  47 ALERT level C = Check. Ensure it is not caused by an omission or oversight
  28 ALERT level G = General information/check it is not something unexpected

   2 ALERT type 1 CIF construction/syntax error, inconsistent or missing data
  54 ALERT type 2 Indicator that the structure model may be wrong or deficient
  11 ALERT type 3 Indicator that the structure quality may be low
  20 ALERT type 4 Improvement, methodology, query or suggestion
   3 ALERT type 5 Informative message, check
```

It is advisable to attempt to resolve as many as possible of the alerts in all categories. Often the minor alerts point to easily fixed oversights, errors and omissions in your CIF or refinement strategy, so attention to these fine details can be worthwhile. In order to resolve some of the more serious problems it may be necessary to carry out additional measurements or structure refinements. However, the purpose of your study may justify the reported deviations and the more serious of these should normally be commented upon in the discussion or experimental section of a paper or in the "special_details" fields of the CIF. checkCIF was carefully designed to identify outliers and unusual parameters, but every test has its limitations and alerts that are not important in a particular case may appear. Conversely, the absence of alerts does not guarantee there are no aspects of the results needing attention. It is up to the individual to critically assess their own results and, if necessary, seek expert advice.

Publication of your CIF in IUCr journals

A basic structural check has been run on your CIF. These basic checks will be run on all CIFs submitted for publication in IUCr journals (*Acta Crystallographica*, *Journal of Applied Crystallography*, *Journal of Synchrotron Radiation*); however, if you intend to submit to *Acta Crystallographica Section C* or *E* or *IUCrData*, you should make sure that full publication checks are run on the final version of your CIF prior to submission.

Publication of your CIF in other journals

Please refer to the *Notes for Authors* of the relevant journal for any special instructions relating to CIF submission.

PLATON version of 06/07/2023; check.def file version of 30/06/2023

Datablock mjp089-2_150k - ellipsoid plot

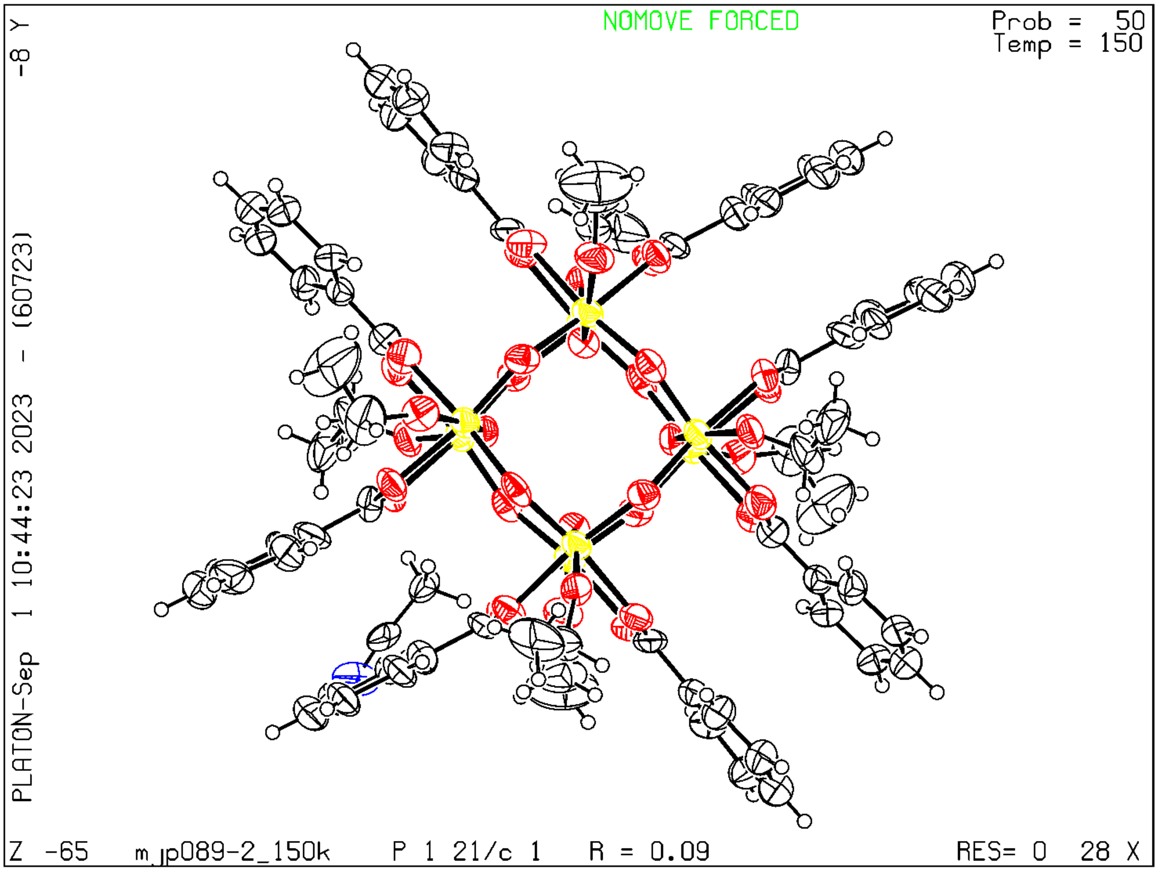

Supplement: Supplementary file 2 — Supporting File 2: anie71298–sup–0002–Data.zip. [file ANIE-65-e25769-s002.zip › CCDC_2312389/MJP089-2_150K_cifreport.pdf]
