# Supplementary material for: Mechanism‐Guided Precision Hydrolysis of Early Transition Metals to Access (Mixed‐Metal) Oxo Clusters
Source: Angew Chem Int Ed Engl. 2026 Feb 24;65(15):e25769. doi: 10.1002/anie.202525769 (PMC13053926; doi:10.1002/anie.202525769)
Supplement: Supplementary file 2 — Supporting File 2: anie71298–sup–0002–Data.zip. [file ANIE-65-e25769-s002.zip › CCDC_2312389/MJP089-2_150K_tables.html]

MJP089-2\_150K


# MJP089-2\_150K

b"\n \n \n "

Table 1 Crystal data and structure refinement for MJP089-2\_150K.

| Identification code | MJP089-2\_150K |
| Empirical formula | C74H83NO36Ta8 |
| Formula weight | 3010.01 |
| Temperature/K | 150 |
| Crystal system | monoclinic |
| Space group | P21/c |
| a/Å | 15.1819(5) |
| b/Å | 27.0298(7) |
| c/Å | 11.1441(4) |
| α/° | 90 |
| β/° | 100.527(3) |
| γ/° | 90 |
| Volume/Å3 | 4496.2(3) |
| Z | 2 |
| ρcalcg/cm3 | 2.223 |
| μ/mm‑1 | 12.373 |
| F(000) | 2812.0 |
| Crystal size/mm3 | 0.14 × 0.113 × 0.08 |
| Radiation | GaKα (λ = 1.34143) |
| 2Θ range for data collection/° | 5.69 to 107.998 |
| Index ranges | -18 ≤ h ≤ 18, -32 ≤ k ≤ 20, -13 ≤ l ≤ 13 |
| Reflections collected | 42827 |
| Independent reflections | 8235 [Rint = 0.2008, Rsigma = 0.1338] |
| Data/restraints/parameters | 8235/360/555 |
| Goodness-of-fit on F2 | 0.979 |
| Final R indexes [I>=2σ (I)] | R1 = 0.0918, wR2 = 0.2166 |
| Final R indexes [all data] | R1 = 0.1633, wR2 = 0.2617 |
| Largest diff. peak/hole / e Å-3 | 3.05/-2.82 |

Table 2 Fractional Atomic Coordinates (×104) and Equivalent Isotropic Displacement Parameters (Å2×103) for MJP089-2\_150K. Ueq is defined as 1/3 of the trace of the orthogonalised UIJ tensor.

| Atom | *x* | *y* | *z* | U(eq) |
| --- | --- | --- | --- | --- |
| Ta2 | 4350.3(7) | 6103.6(4) | 4480.0(11) | 45.9(3) |
| Ta4 | 3483.8(7) | 4387.6(4) | 5916.5(11) | 45.1(3) |
| Ta1 | 4605.3(7) | 5476.8(4) | 7445.0(11) | 46.4(3) |
| Ta3 | 3231.6(7) | 5022.6(4) | 2967.4(11) | 47.9(3) |
| O1 | 3359(11) | 5908(6) | 7155(17) | 57(4) |
| O4 | 4024(11) | 6709(6) | 3648(15) | 55(4) |
| O6 | 2102(11) | 5193(6) | 3814(16) | 51(4) |
| O8 | 3689(9) | 4766(4) | 4562(13) | 41(3) |
| O10 | 4977(12) | 6594(6) | 5904(19) | 61(4) |
| O12 | 2884(11) | 3996(5) | 6926(17) | 54(4) |
| O14 | 3193(11) | 6254(5) | 5296(16) | 53(4) |
| O16 | 3636(11) | 5688(5) | 3313(15) | 51(4) |
| O18 | 4574(10) | 5630(5) | 5741(13) | 45(3) |
| O9 | 5206(12) | 6200(6) | 7688(17) | 61(4) |
| O5 | 2597(11) | 4302(7) | 2669(17) | 60(4) |
| O11 | 4210(11) | 4809(5) | 2268(16) | 52(4) |
| O3 | 3951(10) | 4861(5) | 7089(16) | 52(4) |
| O13 | 4535(10) | 3989(5) | 6077(15) | 49(3) |
| O7 | 2790(10) | 3913(6) | 4468(17) | 52(4) |
| O15 | 2220(10) | 4771(6) | 5554(16) | 51(4) |
| O2 | 2417(12) | 5178(7) | 1490(20) | 71(5) |
| C4 | 5765(15) | 7003(8) | 7650(20) | 46(4) |
| C5 | 6196(16) | 6958(9) | 8840(20) | 52(4) |
| O17 | 4519(13) | 5535(6) | 9087(15) | 59(4) |
| C28 | 1866(14) | 5098(8) | 4760(30) | 55(6) |
| C21 | 2516(15) | 3938(8) | 3360(30) | 51(5) |
| C8 | 6053(17) | 7875(9) | 7640(30) | 55(5) |
| C12 | 2965(17) | 6198(7) | 6300(20) | 46(5) |
| C13 | 2231(15) | 6497(9) | 6570(30) | 67(8) |
| C35 | 2077(19) | 3832(10) | 7160(30) | 69(6) |
| C10 | 4020(20) | 7213(9) | 3900(30) | 73(8) |
| C3 | 5332(16) | 6564(8) | 7060(20) | 48(5) |
| C22 | 2018(15) | 3510(7) | 2711(19) | 52(4) |
| C29 | 1110(17) | 5394(9) | 5160(20) | 65(5) |
| C14 | 1897(17) | 6879(11) | 5780(30) | 68(8) |
| C18 | 1870(20) | 6419(12) | 7590(30) | 72(8) |
| C30 | 778(15) | 5778(7) | 4370(30) | 67(5) |
| C9 | 5686(18) | 7453(8) | 7060(20) | 53(5) |
| C25 | 1110(20) | 2697(10) | 1500(20) | 76(6) |
| C7 | 6464(18) | 7839(9) | 8730(30) | 60(5) |
| C6 | 6598(18) | 7403(9) | 9460(30) | 58(5) |
| C31 | 83(17) | 6055(9) | 4700(30) | 78(6) |
| C32 | -240(19) | 5975(10) | 5760(30) | 81(6) |
| C15 | 1204(19) | 7183(11) | 6100(40) | 76(8) |
| N019 | 420(30) | 6588(17) | 1790(40) | 69(11) |
| C17 | 1250(20) | 6717(12) | 7930(40) | 81(9) |
| C24 | 1183(19) | 3136(8) | 900(30) | 72(6) |
| C23 | 1661(17) | 3525(10) | 1490(20) | 67(5) |
| C27 | 1888(18) | 3088(6) | 3320(30) | 67(6) |
| C34 | 814(15) | 5296(9) | 6240(20) | 66(5) |
| C26 | 1486(19) | 2682(9) | 2720(20) | 70(6) |
| C16 | 890(20) | 7100(12) | 7140(40) | 83(10) |
| C33 | 134(16) | 5592(9) | 6530(30) | 73(6) |
| C11 | 3450(30) | 7500(12) | 3000(30) | 109(16) |
| C38 | 2060(40) | 6404(16) | 1690(50) | 60(10) |
| C37 | 1150(30) | 6528(13) | 1770(50) | 56(9) |
| C36 | 2000(30) | 3810(20) | 8420(30) | 118(14) |
| C1 | 4150(30) | 5763(14) | 10040(40) | 100(9) |
| C19 | 1540(30) | 5069(14) | 940(40) | 99(10) |
| C20 | 1360(40) | 5270(30) | -260(40) | 160(20) |
| C2 | 4720(50) | 6100(30) | 10780(50) | 190(20) |

Table 3 Anisotropic Displacement Parameters (Å2×103) for MJP089-2\_150K. The Anisotropic displacement factor exponent takes the form: -2π2[h2a\*2U11+2hka\*b\*U12+…].

| Atom | U11 | U22 | U33 | U23 | U13 | U12 |
| --- | --- | --- | --- | --- | --- | --- |
| Ta2 | 39.2(6) | 40.2(5) | 62.0(7) | 2.6(4) | 19.4(5) | 3.5(4) |
| Ta4 | 35.9(6) | 39.7(5) | 63.6(7) | 4.4(4) | 19.4(5) | -0.2(4) |
| Ta1 | 44.4(6) | 39.0(5) | 61.4(7) | 1.7(4) | 24.1(5) | -2.0(4) |
| Ta3 | 37.3(6) | 47.5(6) | 61.8(7) | 3.4(4) | 16.5(5) | -2.3(4) |
| O1 | 43(9) | 56(9) | 79(12) | -2(8) | 29(9) | 8(7) |
| O4 | 48(9) | 62(9) | 56(9) | -11(7) | 13(8) | 16(7) |
| O6 | 44(9) | 52(8) | 62(10) | -1(7) | 25(8) | 7(6) |
| O8 | 44(7) | 28(6) | 54(8) | 23(5) | 16(6) | -1(5) |
| O10 | 54(10) | 41(8) | 90(13) | 6(8) | 23(10) | 5(7) |
| O12 | 54(8) | 36(7) | 81(11) | 2(7) | 34(8) | -11(6) |
| O14 | 47(9) | 44(8) | 69(10) | -7(7) | 15(8) | 9(6) |
| O16 | 45(9) | 51(8) | 61(9) | 9(7) | 23(8) | 11(6) |
| O18 | 46(8) | 36(7) | 50(8) | -8(6) | 4(7) | 11(6) |
| O9 | 63(11) | 55(9) | 74(11) | -2(8) | 38(9) | -14(7) |
| O5 | 42(9) | 68(10) | 67(11) | 7(8) | 1(8) | -11(7) |
| O11 | 46(9) | 44(8) | 64(10) | 13(6) | 4(8) | -5(6) |
| O3 | 42(8) | 46(8) | 78(11) | 18(7) | 37(8) | 12(6) |
| O13 | 45(8) | 47(8) | 60(9) | 6(6) | 20(8) | -6(6) |
| O7 | 33(8) | 52(8) | 70(11) | 3(7) | 8(8) | 0(6) |
| O15 | 36(8) | 51(8) | 68(10) | 14(7) | 18(7) | 5(6) |
| O2 | 47(9) | 58(9) | 100(14) | 14(9) | -7(9) | 5(7) |
| C4 | 40(10) | 42(7) | 59(9) | -1(7) | 20(7) | -1(7) |
| C5 | 41(10) | 52(9) | 65(9) | -6(7) | 11(8) | -9(8) |
| O17 | 66(11) | 65(9) | 51(8) | 6(7) | 20(8) | -9(7) |
| C28 | 25(10) | 41(11) | 100(20) | 3(11) | 15(12) | 4(8) |
| C21 | 31(11) | 43(11) | 75(17) | 3(10) | -1(11) | -8(8) |
| C8 | 49(11) | 48(8) | 70(10) | -3(7) | 14(9) | -3(8) |
| C12 | 63(14) | 36(10) | 49(12) | 0(8) | 37(12) | -2(9) |
| C13 | 25(11) | 58(14) | 120(20) | 12(14) | 14(13) | 2(9) |
| C35 | 55(11) | 56(13) | 107(16) | 6(12) | 44(12) | -11(9) |
| C10 | 69(18) | 51(14) | 110(20) | 26(13) | 30(17) | 28(12) |
| C3 | 53(13) | 52(12) | 42(11) | -1(9) | 19(11) | -8(9) |
| C22 | 36(10) | 42(8) | 77(10) | -2(8) | 13(8) | -1(7) |
| C29 | 49(12) | 56(11) | 92(14) | -8(9) | 24(10) | 6(8) |
| C14 | 37(13) | 90(19) | 84(19) | -35(15) | 25(13) | 4(12) |
| C18 | 63(17) | 82(18) | 90(20) | -20(14) | 50(16) | -12(13) |
| C30 | 49(12) | 42(10) | 108(15) | -7(9) | 9(10) | -3(7) |
| C9 | 61(12) | 41(8) | 61(10) | 0(6) | 20(9) | 1(7) |
| C25 | 60(14) | 75(11) | 96(13) | -23(10) | 25(11) | -8(10) |
| C7 | 57(12) | 51(9) | 73(10) | -5(8) | 12(9) | -3(8) |
| C6 | 54(12) | 49(9) | 70(11) | -6(7) | 13(9) | -12(8) |
| C31 | 51(12) | 52(12) | 130(17) | -3(11) | 13(12) | 2(8) |
| C32 | 50(13) | 64(13) | 131(18) | -6(11) | 16(11) | 9(9) |
| C15 | 48(15) | 65(16) | 120(30) | -2(15) | 16(17) | 6(12) |
| N019 | 56(14) | 70(20) | 70(20) | 48(19) | -14(16) | 0(13) |
| C17 | 58(17) | 79(19) | 110(30) | 19(17) | 28(18) | -13(14) |
| C24 | 59(13) | 85(12) | 71(12) | -22(8) | 10(11) | -7(10) |
| C23 | 48(12) | 73(11) | 79(10) | -5(9) | 11(9) | -10(9) |
| C27 | 63(13) | 37(8) | 101(13) | 3(7) | 15(11) | -6(8) |
| C34 | 36(11) | 66(12) | 99(14) | 2(10) | 23(10) | 12(8) |
| C26 | 64(14) | 49(9) | 99(12) | -12(9) | 19(11) | -3(9) |
| C16 | 67(19) | 80(19) | 120(30) | -24(18) | 50(20) | -7(15) |
| C33 | 42(11) | 62(12) | 119(16) | -4(10) | 30(11) | 9(8) |
| C11 | 120(30) | 80(20) | 100(30) | -43(19) | -50(20) | 30(20) |
| C38 | 62(16) | 34(18) | 70(30) | -7(18) | -18(17) | 6(14) |
| C37 | 55(13) | 18(13) | 80(20) | 12(14) | -16(15) | -7(11) |
| C36 | 80(20) | 180(40) | 111(19) | 10(20) | 63(18) | -20(20) |
| C1 | 130(20) | 89(17) | 98(17) | -6(13) | 71(17) | -15(15) |
| C19 | 71(14) | 82(17) | 130(20) | 33(16) | -30(15) | -10(13) |
| C20 | 120(30) | 200(50) | 130(30) | 70(30) | -40(20) | -30(30) |
| C2 | 230(40) | 220(40) | 140(30) | -100(30) | 120(30) | -100(40) |

Table 4 Bond Lengths for MJP089-2\_150K.

| Atom | Atom | Length/Å |  | Atom | Atom | Length/Å |
| --- | --- | --- | --- | --- | --- | --- |
| Ta2 | O4 | 1.901(17) |  | C4 | C5 | 1.37(4) |
| Ta2 | O10 | 2.152(19) |  | C4 | C3 | 1.45(3) |
| Ta2 | O14 | 2.160(16) |  | C4 | C9 | 1.38(3) |
| Ta2 | O16 | 1.900(17) |  | C5 | C6 | 1.46(3) |
| Ta2 | O18 | 1.885(15) |  | O17 | C1 | 1.43(4) |
| Ta2 | O131 | 1.922(15) |  | C28 | C29 | 1.53(3) |
| Ta4 | O8 | 1.896(12) |  | C21 | C22 | 1.49(3) |
| Ta4 | O12 | 1.895(14) |  | C8 | C9 | 1.38(3) |
| Ta4 | O3 | 1.873(18) |  | C8 | C7 | 1.27(4) |
| Ta4 | O13 | 1.907(16) |  | C12 | C13 | 1.45(3) |
| Ta4 | O7 | 2.177(17) |  | C13 | C14 | 1.39(4) |
| Ta4 | O15 | 2.152(15) |  | C13 | C18 | 1.37(4) |
| Ta1 | O1 | 2.194(16) |  | C35 | C36 | 1.44(3) |
| Ta1 | O18 | 1.936(15) |  | C10 | C11 | 1.42(3) |
| Ta1 | O9 | 2.152(16) |  | C22 | C23 | 1.374(17) |
| Ta1 | O111 | 1.929(16) |  | C22 | C27 | 1.363(17) |
| Ta1 | O3 | 1.943(16) |  | C29 | C30 | 1.390(18) |
| Ta1 | O17 | 1.864(16) |  | C29 | C34 | 1.384(18) |
| Ta3 | O6 | 2.153(15) |  | C14 | C15 | 1.43(4) |
| Ta3 | O8 | 1.916(13) |  | C18 | C17 | 1.34(5) |
| Ta3 | O16 | 1.918(16) |  | C30 | C31 | 1.393(18) |
| Ta3 | O5 | 2.173(17) |  | C25 | C24 | 1.376(18) |
| Ta3 | O11 | 1.890(17) |  | C25 | C26 | 1.374(18) |
| Ta3 | O2 | 1.917(19) |  | C7 | C6 | 1.42(4) |
| O1 | C12 | 1.29(3) |  | C31 | C32 | 1.384(18) |
| O4 | C10 | 1.39(3) |  | C32 | C33 | 1.396(18) |
| O6 | C28 | 1.20(3) |  | C15 | C16 | 1.35(5) |
| O10 | C3 | 1.31(3) |  | N019 | C37 | 1.12(7) |
| O12 | C35 | 1.37(3) |  | C17 | C16 | 1.40(5) |
| O14 | C12 | 1.24(3) |  | C24 | C23 | 1.373(17) |
| O9 | C3 | 1.24(3) |  | C27 | C26 | 1.370(17) |
| O5 | C21 | 1.27(3) |  | C34 | C33 | 1.395(17) |
| O7 | C21 | 1.23(3) |  | C38 | C37 | 1.43(8) |
| O15 | C28 | 1.30(3) |  | C1 | C2 | 1.43(3) |
| O2 | C19 | 1.40(4) |  | C19 | C20 | 1.43(3) |

11-X,1-Y,1-Z

Table 5 Bond Angles for MJP089-2\_150K.

| Atom | Atom | Atom | Angle/˚ |  | Atom | Atom | Atom | Angle/˚ |
| --- | --- | --- | --- | --- | --- | --- | --- | --- |
| O4 | Ta2 | O10 | 82.6(7) |  | C3 | O10 | Ta2 | 137.5(14) |
| O4 | Ta2 | O14 | 83.3(6) |  | C35 | O12 | Ta4 | 146.7(18) |
| O4 | Ta2 | O131 | 97.0(7) |  | C12 | O14 | Ta2 | 137.7(16) |
| O10 | Ta2 | O14 | 81.8(6) |  | Ta2 | O16 | Ta3 | 144.4(9) |
| O16 | Ta2 | O4 | 96.3(7) |  | Ta2 | O18 | Ta1 | 147.7(7) |
| O16 | Ta2 | O10 | 171.4(6) |  | C3 | O9 | Ta1 | 139.1(17) |
| O16 | Ta2 | O14 | 89.6(6) |  | C21 | O5 | Ta3 | 133.8(17) |
| O16 | Ta2 | O131 | 97.3(7) |  | Ta3 | O11 | Ta11 | 146.4(10) |
| O18 | Ta2 | O4 | 160.6(6) |  | Ta4 | O3 | Ta1 | 147.4(9) |
| O18 | Ta2 | O10 | 82.7(6) |  | Ta4 | O13 | Ta21 | 144.5(8) |
| O18 | Ta2 | O14 | 82.2(6) |  | C21 | O7 | Ta4 | 137.8(14) |
| O18 | Ta2 | O16 | 96.4(6) |  | C28 | O15 | Ta4 | 134.5(14) |
| O18 | Ta2 | O131 | 95.9(6) |  | C19 | O2 | Ta3 | 138(2) |
| O131 | Ta2 | O10 | 91.3(7) |  | C5 | C4 | C3 | 117(2) |
| O131 | Ta2 | O14 | 173.0(7) |  | C5 | C4 | C9 | 122(2) |
| O8 | Ta4 | O13 | 97.1(6) |  | C9 | C4 | C3 | 121(2) |
| O8 | Ta4 | O7 | 81.7(6) |  | C4 | C5 | C6 | 118(2) |
| O8 | Ta4 | O15 | 82.0(6) |  | C1 | O17 | Ta1 | 151(2) |
| O12 | Ta4 | O8 | 160.2(7) |  | O6 | C28 | O15 | 126(2) |
| O12 | Ta4 | O13 | 96.5(7) |  | O6 | C28 | C29 | 120(2) |
| O12 | Ta4 | O7 | 83.7(7) |  | O15 | C28 | C29 | 113(2) |
| O12 | Ta4 | O15 | 82.6(7) |  | O5 | C21 | C22 | 114(2) |
| O3 | Ta4 | O8 | 95.2(6) |  | O7 | C21 | O5 | 127(2) |
| O3 | Ta4 | O12 | 97.4(7) |  | O7 | C21 | C22 | 119.3(19) |
| O3 | Ta4 | O13 | 96.7(7) |  | C7 | C8 | C9 | 118(2) |
| O3 | Ta4 | O7 | 171.9(6) |  | O1 | C12 | C13 | 117(2) |
| O3 | Ta4 | O15 | 90.8(6) |  | O14 | C12 | O1 | 125(2) |
| O13 | Ta4 | O7 | 91.1(6) |  | O14 | C12 | C13 | 118(2) |
| O13 | Ta4 | O15 | 172.5(7) |  | C14 | C13 | C12 | 119(3) |
| O15 | Ta4 | O7 | 81.4(6) |  | C18 | C13 | C12 | 122(3) |
| O18 | Ta1 | O1 | 82.9(6) |  | C18 | C13 | C14 | 119(2) |
| O18 | Ta1 | O9 | 82.1(6) |  | O12 | C35 | C36 | 116(3) |
| O18 | Ta1 | O3 | 93.6(7) |  | O4 | C10 | C11 | 115(3) |
| O9 | Ta1 | O1 | 82.6(7) |  | O10 | C3 | C4 | 117.8(19) |
| O111 | Ta1 | O1 | 171.5(6) |  | O9 | C3 | O10 | 122(2) |
| O111 | Ta1 | O18 | 95.9(7) |  | O9 | C3 | C4 | 120(2) |
| O111 | Ta1 | O9 | 89.0(7) |  | C23 | C22 | C21 | 121.9(18) |
| O111 | Ta1 | O3 | 96.7(6) |  | C27 | C22 | C21 | 121.0(19) |
| O3 | Ta1 | O1 | 91.7(6) |  | C27 | C22 | C23 | 117(2) |
| O3 | Ta1 | O9 | 173.2(7) |  | C30 | C29 | C28 | 114.5(19) |
| O17 | Ta1 | O1 | 83.5(8) |  | C34 | C29 | C28 | 122.1(19) |
| O17 | Ta1 | O18 | 162.0(7) |  | C34 | C29 | C30 | 123(2) |
| O17 | Ta1 | O9 | 84.5(7) |  | C13 | C14 | C15 | 118(3) |
| O17 | Ta1 | O111 | 95.8(8) |  | C17 | C18 | C13 | 123(3) |
| O17 | Ta1 | O3 | 98.4(7) |  | C29 | C30 | C31 | 116(2) |
| O6 | Ta3 | O5 | 83.9(7) |  | C8 | C9 | C4 | 121(3) |
| O8 | Ta3 | O6 | 81.6(6) |  | C26 | C25 | C24 | 117(2) |
| O8 | Ta3 | O16 | 96.0(6) |  | C8 | C7 | C6 | 127(2) |
| O8 | Ta3 | O5 | 83.9(6) |  | C7 | C6 | C5 | 114(2) |
| O8 | Ta3 | O2 | 160.7(7) |  | C32 | C31 | C30 | 123(3) |
| O16 | Ta3 | O6 | 87.8(6) |  | C31 | C32 | C33 | 118(3) |
| O16 | Ta3 | O5 | 171.6(7) |  | C16 | C15 | C14 | 121(3) |
| O11 | Ta3 | O6 | 174.5(6) |  | C18 | C17 | C16 | 119(3) |
| O11 | Ta3 | O8 | 95.3(6) |  | C23 | C24 | C25 | 121(2) |
| O11 | Ta3 | O16 | 97.0(6) |  | C24 | C23 | C22 | 122(2) |
| O11 | Ta3 | O5 | 91.3(7) |  | C22 | C27 | C26 | 121(2) |
| O11 | Ta3 | O2 | 98.4(8) |  | C29 | C34 | C33 | 118(2) |
| O2 | Ta3 | O6 | 83.6(8) |  | C27 | C26 | C25 | 122(2) |
| O2 | Ta3 | O16 | 95.7(7) |  | C15 | C16 | C17 | 120(3) |
| O2 | Ta3 | O5 | 82.3(7) |  | C34 | C33 | C32 | 121(2) |
| C12 | O1 | Ta1 | 134.7(14) |  | N019 | C37 | C38 | 175(5) |
| C10 | O4 | Ta2 | 139.2(17) |  | C2 | C1 | O17 | 115(3) |
| C28 | O6 | Ta3 | 136.7(15) |  | O2 | C19 | C20 | 109(3) |
| Ta4 | O8 | Ta3 | 148.1(8) |  |  |  |  |  |

11-X,1-Y,1-Z

Table 6 Torsion Angles for MJP089-2\_150K.

| A | B | C | D | Angle/˚ |  | A | B | C | D | Angle/˚ |
| --- | --- | --- | --- | --- | --- | --- | --- | --- | --- | --- |
| Ta2 | O4 | C10 | C11 | -162(3) |  | O13 | Ta4 | O12 | C35 | -149(3) |
| Ta2 | O10 | C3 | O9 | -15(4) |  | O13 | Ta4 | O3 | Ta1 | 87.8(15) |
| Ta2 | O10 | C3 | C4 | 174.3(15) |  | O7 | Ta4 | O8 | Ta3 | 40.7(15) |
| Ta2 | O14 | C12 | O1 | -17(4) |  | O7 | Ta4 | O12 | C35 | -58(3) |
| Ta2 | O14 | C12 | C13 | 160.1(18) |  | O7 | C21 | C22 | C23 | 177(2) |
| Ta4 | O12 | C35 | C36 | -140(3) |  | O7 | C21 | C22 | C27 | -2(3) |
| Ta4 | O7 | C21 | O5 | 2(4) |  | O15 | Ta4 | O8 | Ta3 | -41.8(15) |
| Ta4 | O7 | C21 | C22 | -177.2(15) |  | O15 | Ta4 | O12 | C35 | 24(3) |
| Ta4 | O15 | C28 | O6 | 18(4) |  | O15 | Ta4 | O3 | Ta1 | -92.0(15) |
| Ta4 | O15 | C28 | C29 | -158.4(17) |  | O15 | C28 | C29 | C30 | 174(2) |
| Ta1 | O1 | C12 | O14 | 10(4) |  | O15 | C28 | C29 | C34 | -3(4) |
| Ta1 | O1 | C12 | C13 | -167.3(17) |  | O2 | Ta3 | O11 | Ta11 | -174.7(15) |
| Ta1 | O9 | C3 | O10 | 7(4) |  | C4 | C5 | C6 | C7 | 1(3) |
| Ta1 | O9 | C3 | C4 | 177.7(16) |  | C5 | C4 | C3 | O10 | -175(2) |
| Ta1 | O17 | C1 | C2 | -104(6) |  | C5 | C4 | C3 | O9 | 14(3) |
| Ta3 | O6 | C28 | O15 | -11(4) |  | C5 | C4 | C9 | C8 | 1(4) |
| Ta3 | O6 | C28 | C29 | 165.3(17) |  | C28 | C29 | C30 | C31 | 180(2) |
| Ta3 | O5 | C21 | O7 | 2(4) |  | C28 | C29 | C34 | C33 | 179(3) |
| Ta3 | O5 | C21 | C22 | -179.3(15) |  | C21 | C22 | C23 | C24 | -178(3) |
| Ta3 | O2 | C19 | C20 | -174(4) |  | C21 | C22 | C27 | C26 | -175(2) |
| O1 | Ta1 | O17 | C1 | -16(4) |  | C8 | C7 | C6 | C5 | -1(4) |
| O1 | C12 | C13 | C14 | 168(2) |  | C12 | C13 | C14 | C15 | -177(3) |
| O1 | C12 | C13 | C18 | -11(4) |  | C12 | C13 | C18 | C17 | 173(3) |
| O4 | Ta2 | O18 | Ta1 | -2(3) |  | C13 | C14 | C15 | C16 | 0(4) |
| O6 | C28 | C29 | C30 | -3(4) |  | C13 | C18 | C17 | C16 | 7(5) |
| O6 | C28 | C29 | C34 | 180(3) |  | C3 | C4 | C5 | C6 | -177(2) |
| O8 | Ta4 | O12 | C35 | -16(4) |  | C3 | C4 | C9 | C8 | 176(2) |
| O8 | Ta4 | O3 | Ta1 | -9.9(16) |  | C22 | C27 | C26 | C25 | -7(4) |
| O8 | Ta3 | O11 | Ta11 | -8.2(15) |  | C29 | C30 | C31 | C32 | 3(4) |
| O10 | Ta2 | O18 | Ta1 | 38.8(16) |  | C29 | C34 | C33 | C32 | 0(4) |
| O12 | Ta4 | O8 | Ta3 | -2(3) |  | C14 | C13 | C18 | C17 | -6(5) |
| O12 | Ta4 | O3 | Ta1 | -174.7(15) |  | C14 | C15 | C16 | C17 | 1(5) |
| O14 | Ta2 | O18 | Ta1 | -43.9(16) |  | C18 | C13 | C14 | C15 | 2(4) |
| O14 | C12 | C13 | C14 | -9(4) |  | C18 | C17 | C16 | C15 | -4(5) |
| O14 | C12 | C13 | C18 | 172(3) |  | C30 | C29 | C34 | C33 | 1(4) |
| O16 | Ta2 | O18 | Ta1 | -132.6(16) |  | C30 | C31 | C32 | C33 | -2(5) |
| O16 | Ta3 | O11 | Ta11 | 88.5(15) |  | C9 | C4 | C5 | C6 | -2(3) |
| O18 | Ta1 | O17 | C1 | 25(5) |  | C9 | C4 | C3 | O10 | 10(3) |
| O9 | Ta1 | O17 | C1 | 67(4) |  | C9 | C4 | C3 | O9 | -161(2) |
| O5 | Ta3 | O11 | Ta11 | -92.2(15) |  | C9 | C8 | C7 | C6 | 0(4) |
| O5 | C21 | C22 | C23 | -2(3) |  | C25 | C24 | C23 | C22 | -6(5) |
| O5 | C21 | C22 | C27 | 179(2) |  | C7 | C8 | C9 | C4 | 0(4) |
| O111 | Ta1 | O17 | C1 | 156(4) |  | C31 | C32 | C33 | C34 | 0(5) |
| O3 | Ta4 | O8 | Ta3 | -131.8(15) |  | C24 | C25 | C26 | C27 | 2(4) |
| O3 | Ta4 | O12 | C35 | 113(3) |  | C23 | C22 | C27 | C26 | 5(4) |
| O3 | Ta1 | O17 | C1 | -106(4) |  | C27 | C22 | C23 | C24 | 1(4) |
| O131 | Ta2 | O18 | Ta1 | 129.3(16) |  | C34 | C29 | C30 | C31 | -3(4) |
| O13 | Ta4 | O8 | Ta3 | 130.8(15) |  | C26 | C25 | C24 | C23 | 4(4) |

11-X,1-Y,1-Z

Table 7 Hydrogen Atom Coordinates (Å×104) and Isotropic Displacement Parameters (Å2×103) for MJP089-2\_150K.

| Atom | *x* | *y* | *z* | U(eq) |
| --- | --- | --- | --- | --- |
| H5 | 6234.52 | 6647.3 | 9242.93 | 63 |
| H8 | 5999.37 | 8184.94 | 7227.94 | 66 |
| H35A | 1971.61 | 3506.58 | 6799.32 | 83 |
| H35B | 1619.81 | 4058.88 | 6761.32 | 83 |
| H10A | 4620.18 | 7337.69 | 3944.96 | 88 |
| H10B | 3829.08 | 7259.59 | 4675.96 | 88 |
| H14 | 2122 | 6936 | 5052.68 | 82 |
| H18 | 2058.6 | 6138.94 | 8085.82 | 87 |
| H30 | 1012.52 | 5847.76 | 3658.46 | 80 |
| H9 | 5373.71 | 7471.82 | 6243.14 | 64 |
| H25 | 812.55 | 2418.35 | 1093.46 | 91 |
| H7 | 6710.16 | 8135.91 | 9108.72 | 73 |
| H6 | 6920.65 | 7400.7 | 10271.9 | 69 |
| H31 | -179.87 | 6309.28 | 4161.14 | 94 |
| H32 | -704.48 | 6175.56 | 5968.67 | 98 |
| H15 | 962.94 | 7446.61 | 5580.84 | 92 |
| H17 | 1055.5 | 6669.43 | 8685.49 | 97 |
| H24 | 898.18 | 3170.39 | 73.38 | 86 |
| H23 | 1748.52 | 3812.52 | 1031.22 | 80 |
| H27 | 2079.59 | 3074.39 | 4183.65 | 80 |
| H34 | 1066.78 | 5034.23 | 6757.19 | 79 |
| H26 | 1467.76 | 2381.63 | 3156.07 | 84 |
| H16 | 422.85 | 7301.42 | 7336.68 | 100 |
| H33 | -78.19 | 5533 | 7270.92 | 87 |
| H11A | 3650.29 | 7467.7 | 2213.25 | 164 |
| H11B | 3477.21 | 7847.85 | 3246.42 | 164 |
| H11C | 2831.55 | 7380.62 | 2912.26 | 164 |
| H38A | 2057.47 | 6148.22 | 1071.96 | 91 |
| H38B | 2364.9 | 6280.73 | 2486.14 | 91 |
| H38C | 2363.25 | 6699.82 | 1471.36 | 91 |
| H36A | 1460.67 | 3616.78 | 8500.4 | 177 |
| H36B | 1951.85 | 4140.79 | 8741.11 | 177 |
| H36C | 2528.07 | 3641.51 | 8887.01 | 177 |
| H1A | 3564.45 | 5892.28 | 9670.19 | 120 |
| H1B | 4074.55 | 5508.49 | 10625.99 | 120 |
| H19A | 1120.66 | 5216.59 | 1400.21 | 119 |
| H19B | 1451.36 | 4713.1 | 904.91 | 119 |
| H20A | 1014.35 | 5034.42 | -825.65 | 241 |
| H20B | 1927.88 | 5343.02 | -528.8 | 241 |
| H20C | 1016.18 | 5579.21 | -262.89 | 241 |
| H2A | 5300.13 | 5986.58 | 11178.71 | 282 |
| H2B | 4795.33 | 6366.38 | 10232.91 | 282 |
| H2C | 4396.33 | 6221.98 | 11386.81 | 282 |

Table 8 Atomic Occupancy for MJP089-2\_150K.

| Atom | *Occupancy* |  | Atom | *Occupancy* |  | Atom | *Occupancy* |
| --- | --- | --- | --- | --- | --- | --- | --- |
| N019 | 0.5 |  | C38 | 0.5 |  | H38A | 0.5 |
| H38B | 0.5 |  | H38C | 0.5 |  | C37 | 0.5 |

Experimental

Single crystals of C74H83NO36Ta8
[MJP089-2\_150K]
were
[].
A suitable crystal was selected and
[]
on a
STOE STADIVARI
diffractometer. The crystal was kept at 150 K during data collection.
Using Olex2 [1], the structure was solved with the
SHELXT
[2] structure solution program using
Intrinsic Phasing
and refined with the
SHELXL
[3] refinement package using
Least Squares
minimisation.

1. Dolomanov, O.V., Bourhis, L.J., Gildea, R.J, Howard, J.A.K. & Puschmann, H.
   (2009), J. Appl. Cryst. 42, 339-341.
2. Sheldrick, G.M. (2015). Acta Cryst. A71, 3-8.
3. Sheldrick, G.M. (2015). Acta Cryst. C71, 3-8.

Crystal structure determination of
[MJP089-2\_150K]

**Crystal Data**
for C74H83NO36Ta8 (*M*=3010.01 g/mol):
monoclinic, space group P21/c (no. 14),
*a* = 15.1819(5) Å, *b* = 27.0298(7) Å, *c* = 11.1441(4) Å, *β* = 100.527(3)°,
*V*= 4496.2(3) Å3,
*Z* = 2,
*T* = 150 K,
μ(GaKα) = 12.373 mm-1,
*Dcalc* = 2.223 g/cm3,
42827 reflections measured (5.69° ≤ 2Θ ≤ 107.998°),
8235 unique (*R*int = 0.2008, Rsigma = 0.1338) which were used in all calculations.
The final *R*1 was 0.0918
(I > 2σ(I)) and *wR*2 was 0.2617 (all data).

Refinement model description

Number of restraints - 360,
number of constraints - unknown.

Details:

```
1. Fixed Uiso
```

This report has been created with Olex2, compiled on
2023.08.24 svn.re1ec1418 for OlexSys. Please
let us know
if there are any errors or if you would like to have additional features.
