# Supplementary material for: Mechanism‐Guided Precision Hydrolysis of Early Transition Metals to Access (Mixed‐Metal) Oxo Clusters
Source: Angew Chem Int Ed Engl. 2026 Feb 24;65(15):e25769. doi: 10.1002/anie.202525769 (PMC13053926; doi:10.1002/anie.202525769)
Supplement: Supplementary file 2 — Supporting File 2: anie71298–sup–0002–Data.zip. [file ANIE-65-e25769-s002.zip › CCDC_2453142/hw07_150k_new.rtf]

;;;;;;;fLine0fBehindDocument1shapeType1pibhw07_150k_new

Submitted by: 	Harry Wilson
		University of Basel
Solved by: 	Alessandro Prescimone
Sample ID: 	HW07_150K
R1=12.44%


Crystal Data and Experimental


Experimental. Single colourless block-shaped crystals of hw07_150k_new were used as supplied. A suitable crystal with dimensions 0.20 × 0.17 × 0.12 mm was selected and mounted on a STOE STADIVARI diffractometer. The crystal was kept at a steady T = 150 K during data collection. The structure was solved with the ShelXT 2018/2 (Sheldrick, 2018) solution program using iterative methods and by using Olex2 1.5 (Dolomanov et al., 2009) as the graphical interface. The model was refined with ShelXL 2019/3 (Sheldrick, 2015) using full matrix least squares minimisation on |F|2.
Crystal Data. C72.3H126.8O38.6Zr6, Mr = 2161.05, triclinic, P-1 (No. 2), a = 14.1011(3) Å, b = 15.6314(3) Å, c = 24.0684(5) Å, a = 94.094(2)°, b = 96.378(2)°, g = 109.376(2)°, V = 4940.44(19) Å3, T = 150 K, Z = 2, Z' = 1, m(GaKa) = 3.794, 75558 reflections measured, 19060 unique (Rint = 0.0923) which were used in all calculations. The final wR2 was 0.3107 (all data) and R1 was 0.1244 (I≥2s(I)).
Compound 	hw07_150k_new 	
 	 	
Formula 	C72.3H126.8O38.6Zr6 	
Dcalc./ g cm-3 	1.453 	
m/mm-1 	3.794 	
Formula Weight 	2161.05 	
Colour 	colourless 	
Shape 	block-shaped 	
Size/mm 	0.20×0.17×0.12 	
T/K 	150 	
Crystal System 	triclinic 	
Space Group 	P-1 	
a/Å 	14.1011(3) 	
b/Å 	15.6314(3) 	
c/Å 	24.0684(5) 	
a/° 	94.094(2) 	
b/° 	96.378(2) 	
g/° 	109.376(2) 	
V/Å3 	4940.44(19) 	
Z 	2 	
Z' 	1 	
Wavelength/Å 	1.34143 	
Radiation type 	GaKa 	
Qmin/° 	3.926 	
Qmax/° 	55.883 	
Index range h 	-11 ≥ h ≥ 17 	
Index range k 	-19 ≥ k ≥ 18 	
Index range l 	-29 ≥ l ≥ 28 	
Measured Refl's. 	75558 	
Indep't Refl's 	19060 	
Refl's I≥2s(I) 	12576 	
Rint 	0.0923 	
Parameters 	785 	
Restraints 	285 	
Largest Peak/eÅ3 	1.715 	
Deepest Hole/eÅ3 	-1.229 	
GooF 	1.119 	
R1 (I≥2s(I) / all) 	0.1244 / 0.1493 	
wR2 (I≥2s(I) / all) 	0.2960 / 0.3107 	

Structure Quality Indicators
Reflections:		
Refinement:		
A colourless block-shaped crystal with dimensions 0.20 × 0.17 × 0.12 mm was mounted. Data were collected using a STOE STADIVARI diffractometer equipped with an Oxford Cryosystems low-temperature device operating at T = 150 K.
Data were measured using rotation method, w scans with GaKa radiation. The diffraction pattern was indexed and the total number of runs and images was based on the strategy calculation from the program X-Area Pilatus3_SV 1.31.186.0 (STOE, 2022) . The maximum resolution achieved was Q = 55.883° (0.81 Å).
The unit cell was refined using X-Area Pilatus3_SV 1.31.186.0 (STOE, 2022) on 45569 reflections, 60% of the observed reflections.
Data reduction, scaling and absorption corrections were performed using X-Area Pilatus3_SV 1.31.186.0 (STOE, 2022). The final completeness is 99.40 % out to 55.883° in Q. A multi-scan absorption correction was performed using STOE. The absorption coefficient m of this material is 3.794 mm-1 at this wavelength (l = 1.34143Å) and the minimum and maximum transmissions are 0.000 and 0.016.
The structure was solved in the space group P-1 (# 2) by ShelXT 2018/2 (Sheldrick, 2018) using iterative methods. It was refined by full matrix least squares minimisation on |F|2 using version 2019/3 of ShelXL 2019/3 (Sheldrick, 2015). All non-hydrogen atoms were refined anisotropically.
Most hydrogen atom positions were calculated geometrically and refined using the riding model, but some hydrogen atoms were refined freely.
_refine_special_details: crystals were all weakly diffracting at high angles even with a metaljet source.there is a lot of disder in the carboxylate ligands.so much so that in those involving C20 and C14 it was not possible to find the terminal CH3 of the longest tail end of the ligand. So no H were placed on the involved atoms as they could either be CH2 or CH3. Chemical formulae take this into account and have the right amount of C and H atoms.A lot of SADI, EADP, DFIX, D&nbsp;&Aring; had to be used. Some atoms had to be refined isotropically.
_exptl_absorpt_process_details: STOE X-Red32, absorption correction by Gaussian integration, analogous toP. Coppens in: F. R. Ahmed (Editor), "Crystallographic Computing", Munksgaard,Copenhagen (1970), 255 - 270. Afterwards scaling of reflection intensities wasperformed within STOE LANA. J. Koziskova, F. Hahn, J. Richter, J. Kozisek, ActaChimica Slovaca, vol. 9, no. 2, 2016, pp. 136 - 140.Finally a spherical absorption correction was done within STOE LANA.
There is a single formula unit in the asymmetric unit, which is represented by the reported sum formula. In other words: Z is 2 and Z' is 1. The moiety formula is C58 H98 O33 Zr6, C4.8 H9.8 O1.6, C4.5 H9 O2, C5 H10 O2.
Data Plots: Diffraction Data
 	 	
 	 	
Data Plots: Refinement and Data
 	 	
Reflection Statistics

Total reflections (after filtering) 	75558 	Unique reflections 	19060 	
Completeness 	0.979 	Mean I/s 	11.2 	
hklmax collected 	(17, 18, 28) 	hklmin collected 	(-11, -19, -29) 	
hklmax used 	(17, 19, 29) 	hklmin used 	(-17, -19, 0) 	
Lim dmax collected 	100.0 	Lim dmin collected 	0.67 	
dmax used 	9.8 	dmin used 	0.81 	
Friedel pairs 	4069 	Friedel pairs merged 	1 	
Inconsistent equivalents 	1094 	Rint 	0.0923 	
Rsigma 	0.0634 	Intensity transformed 	0 	
Omitted reflections 	0 	Omitted by user (OMIT hkl) 	15 	
Multiplicity 	(4744, 5120, 4009, 3485, 2596, 1653, 879, 407, 171, 56, 13, 5, 1) 	Maximum multiplicity 	13 	
Removed systematic absences 	0 	Filtered off (Shel/OMIT) 	0 	


Table 0: Fractional Atomic Coordinates (×104) and Equivalent Isotropic Displacement Parameters (Å2×103) for hw07_150k_new. Ueq is defined as 1/3 of the trace of the orthogonalised Uij.

Atom	x	y	z	Ueq	
Zr1	2467.7(7)	1504.3(7)	7537.1(4)	85.5(3)	
Zr4	4930.9(6)	3069.2(8)	7694.6(4)	90.1(3)	
Zr6	1535.9(6)	3305.0(7)	7767.2(4)	87.8(3)	
Zr3	3371.6(7)	3029.5(7)	8753.4(4)	84.9(3)	
Zr2	3067.9(7)	3316.9(8)	6713.6(4)	91.6(3)	
Zr5	3987.3(7)	4890.0(7)	7919.3(4)	88.2(3)	
O1	4174(5)	3883(5)	7386(3)	84.0(18)	
O4	4705(5)	4046(5)	8431(3)	86.7(19)	
O6	2024(5)	2531(5)	7193(3)	83.4(18)	
O8	3546(5)	2200(6)	6987(3)	91(2)	
O10	4777(6)	2798(6)	9143(3)	105(2)	
O12	2346(7)	676(6)	8250(4)	104(2)	
O14	3695(5)	2389(5)	8062(3)	82.7(18)	
O16	942(6)	3275(7)	8581(4)	112(3)	
O18	6251(6)	4293(7)	7811(4)	111(3)	
O20	1360(7)	4650(7)	7947(4)	117(3)	
O22	296(6)	3328(7)	7075(4)	118(3)	
O24	2893(7)	1698(6)	9035(4)	108(2)	
O26	1859(5)	2187(5)	8165(3)	86.0(19)	
O28	831(6)	539(6)	7391(4)	111(3)	
O30	5052(6)	1714(7)	7545(4)	115(3)	
O32	2949(7)	5662(6)	8020(4)	116(3)	
O34	4046(7)	4145(7)	9469(3)	110(3)	
O36	4595(6)	5824(6)	8656(4)	114(3)	
O38	-49(6)	2221(7)	7579(4)	115(2)	
O40	2130(6)	3098(7)	9217(3)	111(3)	
O41	5784(6)	2916(6)	8474(4)	106(3)	
O21	2926(5)	3904(5)	8266(3)	81.1(17)	
O11	2504(5)	4190(6)	7250(3)	94(2)	
O23	3654(7)	4679(8)	6419(4)	117(3)	
O3	4338(6)	3326(7)	6260(4)	117(3)	
O25	5707(6)	5475(6)	7896(4)	111(3)	
O13	1728(6)	573(7)	6717(4)	119(3)	
O27	5517(6)	3130(8)	6899(4)	122(3)	
O7	1744(7)	3342(9)	6086(4)	130(3)	
O29	5595(6)	5635(7)	9378(4)	115(3)	
O15	4205(7)	5699(7)	7220(5)	120(3)	
O31	2305(7)	2242(8)	5958(4)	127(3)	
O2	3477(7)	697(6)	7442(4)	113(3)	
O33	3871(8)	1048(9)	6122(4)	112(3)	
O17	-1072(9)	790(11)	8031(7)	174(5)	
C1	903(15)	240(13)	6920(8)	144(3)	
O9	346(8)	1024(8)	8653(5)	133(4)	
C4	2565(11)	884(11)	8782(7)	113(4)	
C6	-330(13)	2679(16)	7200(9)	148(2)	
C8	5584(12)	2801(10)	8965(7)	117(5)	
C10	3310(30)	510(30)	5782(14)	225(6)	
C12	1289(13)	3214(10)	9076(7)	118(5)	
C14	4034(12)	5488(14)	6673(8)	126(5)	
O37	2299(10)	150(11)	5786(4)	159(6)	
C16	1988(19)	5441(18)	8029(13)	205(4)	
C18	5300(20)	6149(18)	9069(13)	212(5)	
C20	6390(9)	5155(14)	7871(6)	124(5)	
C24	6351(12)	2604(13)	9400(8)	152(6)	
C26	1710(20)	2640(20)	5797(9)	205(5)	
C28	-541(19)	596(15)	8453(10)	158(7)	
C30	2437(17)	136(11)	9168(9)	179(9)	
C32	5183(12)	3206(13)	6416(8)	134(5)	
C34	3670(20)	253(16)	5228(9)	225(6)	
C36	7448(15)	5830(20)	7918(17)	192(7)	
C38	98(13)	-559(12)	6567(8)	144(3)	
C40	4818(14)	131(13)	7353(8)	149(6)	
C42	-1463(12)	2285(14)	6952(8)	148(2)	
C44	5899(19)	7102(16)	9198(10)	212(5)	
C46	8170(40)	5620(40)	8348(16)	192(7)	
C48	1011(17)	5233(18)	6240(9)	250	
C50	720(18)	3227(18)	9557(11)	200	
C52	1636(17)	6244(16)	7990(9)	205(4)	
C56	3212(18)	555(17)	4705(9)	225(6)	
C58	1270(20)	6398(19)	5452(11)	250	
C60	6790(20)	1914(16)	9181(11)	225(6)	
C62	902(17)	2286(17)	5278(8)	205(5)	
C64	-1248(18)	-143(13)	8739(9)	99(7)	
C68	191(18)	1323(16)	5275(8)	205(5)	
C70	-981(19)	-510(30)	6465(15)	144(3)	
C72	-1993(12)	2820(14)	7218(6)	148(2)	
C74	6823(18)	7496(16)	9497(11)	212(5)	
C78	5195(17)	7717(16)	8963(9)	212(5)	
C80	-1557(11)	2277(13)	6325(7)	148(2)	
C5	360(30)	-1441(19)	6653(15)	144(3)	
C41	4560(40)	6260(30)	6320(19)	163(12)	
C21	-1524(17)	-1031(14)	8376(9)	200	
C43	720(30)	4150(20)	9763(15)	200	
C45	3269(18)	-776(16)	5055(9)	225(6)	
C23	7730(40)	5860(30)	7336(16)	192(7)	
C47	2166(16)	7113(15)	7822(10)	205(4)	
C49	4080(30)	-806(17)	7384(16)	158(13)	
C25	3420(30)	180(30)	9533(13)	304(13)	
C51	1990(20)	-785(16)	8836(13)	304(13)	
C13	1381(17)	2443(16)	4764(8)	205(5)	
C53	4160(40)	7110(30)	6579(15)	220(16)	
C27	3890(40)	6100(30)	5746(16)	250	
C55	5930(20)	2456(16)	5731(12)	250	
C7	7147(18)	3473(13)	9649(11)	225(6)	
C57	440(40)	2390(30)	9850(19)	200	
C59	-750(30)	-250(30)	9291(12)	200	
C15	-964(19)	-800(20)	6757(15)	144(3)	
C61	-180(20)	-1427(19)	6870(15)	144(3)	
C63	7910(30)	5890(30)	8509(15)	192(7)	
C2	7505(14)	5670(30)	7892(14)	192(7)	
C65	5340(40)	7120(30)	6630(20)	163(12)	
C33	5040(40)	6070(40)	5820(20)	163(12)	
C67	4260(30)	6315(19)	6324(13)	159(12)	
C17	240(20)	5624(16)	6047(9)	250	
C35	670(20)	6565(17)	5894(11)	250	
C71	7680(40)	6540(30)	7624(18)	192(7)	
C9	6897(17)	4045(15)	6106(12)	250	
C73	5880(15)	3321(13)	5945(9)	167(7)	
O39	1684(9)	5352(8)	6687(5)	144(4)	
O5	757(11)	4459(12)	5898(7)	209(7)	
C37	5150(40)	-110(40)	7915(13)	253(12)	
C75	5630(20)	380(20)	6983(11)	253(12)	
C77	0(30)	3710(30)	9440(20)	200	
C79	-340(20)	2540(20)	9460(20)	200	
C81	-2210(30)	-30(30)	8840(20)	200	
C82	-594(17)	-1285(16)	8350(10)	200	
C83	2189(18)	3347(15)	4787(8)	205(5)	
C84	-1830(11)	3067(13)	7831(6)	148(2)	
C85	5607(17)	7921(15)	8419(9)	212(5)	
C86	3674(18)	-1082(17)	5580(8)	225(6)	
C87	-170(40)	2460(40)	10320(20)	250(30)	
C88	110(40)	4420(30)	9310(20)	250	
C89	1231(14)	6138(14)	8534(10)	205(4)	
C90	2108(15)	6711(14)	8953(8)	205(4)	
C91	3991(18)	-12(19)	9081(12)	240	
C92	6790(20)	4193(17)	9900(11)	225(6)	
C93	-1160(20)	20(20)	6545(15)	144(3)	
C94	770(20)	-1650(20)	7016(15)	144(3)	
C11	-398(17)	5641(18)	6497(11)	250	
C29	6260(20)	-200(20)	7146(12)	250	
C22	4448(11)	937(12)	7455(6)	115(4)	
C3	6650(20)	4801(16)	5852(12)	250	


Table 0: Anisotropic Displacement Parameters (×104) for hw07_150k_new. The anisotropic displacement factor exponent takes the form: -2p2[h2a*2 × U11+ ... +2hka* × b* × U12]

Atom	U11	U22	U33	U23	U13	U12	
Zr1	58.7(5)	107.4(7)	90.9(6)	-9.9(5)	2.1(4)	36.2(4)	
Zr4	49.2(5)	128.8(8)	97.9(6)	-0.5(5)	6.1(4)	41.9(5)	
Zr6	49.0(5)	115.1(7)	102.0(7)	-8.3(5)	8.3(4)	36.9(4)	
Zr3	62.1(5)	106.5(7)	81.3(6)	-1.6(5)	5.7(4)	26.6(4)	
Zr2	61.1(5)	136.7(9)	81.9(6)	3.1(5)	4.5(4)	44.1(5)	
Zr5	59.6(5)	105.8(7)	99.8(7)	5.2(5)	6.6(4)	32.2(5)	
O1	54(4)	118(5)	83(4)	-1(4)	11(3)	35(3)	
O4	54(4)	108(5)	97(5)	10(4)	5(3)	28(3)	
O6	55(4)	118(5)	80(4)	-6(4)	2(3)	39(3)	
O8	60(4)	139(6)	82(4)	-10(4)	6(3)	51(4)	
O10	75(5)	133(7)	101(5)	8(5)	-13(4)	40(5)	
O12	95(6)	104(6)	114(6)	9(5)	14(5)	38(4)	
O14	60(4)	112(5)	84(4)	-1(4)	8(3)	44(4)	
O16	63(5)	147(8)	125(7)	-19(6)	31(5)	35(5)	
O18	57(4)	131(7)	141(7)	12(6)	16(4)	30(5)	
O20	80(5)	147(6)	143(7)	-9(6)	12(5)	70(4)	
O22	65(4)	164(7)	132(7)	-11(5)	-4(4)	60(4)	
O24	102(6)	112(6)	100(6)	11(5)	2(5)	28(5)	
O26	60(4)	115(5)	83(4)	-7(4)	10(3)	33(4)	
O28	73(5)	136(7)	104(6)	-24(5)	12(4)	19(4)	
O30	67(5)	141(8)	144(7)	-21(6)	-7(5)	61(5)	
O32	92(5)	103(6)	156(7)	-5(5)	-3(5)	48(4)	
O34	84(6)	135(7)	93(5)	-10(5)	6(4)	21(5)	
O36	80(5)	126(7)	123(6)	-3(5)	-6(5)	29(5)	
O38	52(4)	142(7)	143(7)	-25(5)	8(4)	32(4)	
O40	70(5)	149(7)	98(5)	-15(5)	21(4)	23(5)	
O41	65(4)	151(7)	103(6)	2(5)	-14(4)	50(5)	
O21	62(4)	101(5)	88(4)	3(3)	14(3)	39(3)	
O11	67(4)	130(6)	95(5)	7(4)	7(4)	51(4)	
O23	93(6)	155(9)	114(7)	31(6)	16(5)	53(6)	
O3	74(5)	177(9)	101(6)	6(5)	28(4)	43(5)	
O25	61(5)	138(7)	126(7)	16(5)	3(4)	26(4)	
O13	72(5)	158(8)	119(6)	-26(5)	8(5)	39(5)	
O27	71(5)	203(10)	107(6)	12(6)	27(5)	63(6)	
O7	89(6)	198(10)	105(6)	19(6)	-22(5)	63(6)	
O29	77(5)	139(7)	106(6)	-14(5)	13(4)	14(5)	
O15	94(6)	131(7)	130(8)	27(6)	-2(5)	37(5)	
O31	102(7)	185(10)	95(6)	-14(6)	-7(5)	63(6)	
O2	92(6)	127(7)	129(7)	-16(5)	9(5)	60(5)	
O33	77(6)	175(10)	102(7)	-17(7)	18(5)	72(7)	
O17	94(8)	211(14)	211(14)	55(11)	35(9)	33(8)	
C1	133(5)	145(5)	147(5)	1(4)	13(4)	47(4)	
O9	85(6)	172(10)	135(8)	17(7)	34(6)	27(6)	
C4	100(10)	133(12)	106(10)	3(9)	5(8)	45(8)	
C6	86(4)	201(6)	164(5)	-4(5)	10(4)	67(4)	
C8	94(10)	125(10)	127(12)	-13(9)	-39(9)	52(8)	
C10	160(9)	319(13)	172(9)	-73(10)	20(8)	73(10)	
C12	98(11)	127(11)	120(11)	-15(9)	43(9)	26(8)	
C14	99(10)	153(14)	139(14)	50(12)	10(10)	55(10)	
O37	100(9)	214(15)	144(11)	-78(10)	6(8)	51(9)	
C16	136(6)	166(7)	321(12)	-13(9)	27(7)	73(5)	
C18	169(10)	174(9)	239(12)	32(9)	-26(9)	4(7)	
C20	48(6)	192(17)	126(11)	38(11)	13(6)	28(9)	
C24	108(11)	188(17)	156(14)	-19(12)	-39(10)	73(11)	
C26	203(12)	258(14)	123(6)	-17(8)	-35(7)	64(9)	
C28	144(18)	183(19)	163(18)	38(15)	75(15)	57(16)	
C30	230(20)	88(10)	184(18)	23(11)	-27(16)	25(12)	
C32	92(10)	207(17)	128(12)	16(11)	50(9)	74(11)	
C34	160(9)	319(13)	172(9)	-73(10)	20(8)	73(10)	
C38	133(5)	145(5)	147(5)	1(4)	13(4)	47(4)	
C42	86(4)	201(6)	164(5)	-4(5)	10(4)	67(4)	
C44	169(10)	174(9)	239(12)	32(9)	-26(9)	4(7)	
C52	136(6)	166(7)	321(12)	-13(9)	27(7)	73(5)	
C56	160(9)	319(13)	172(9)	-73(10)	20(8)	73(10)	
C62	203(12)	258(14)	123(6)	-17(8)	-35(7)	64(9)	
C64	90(10)	103(10)	100(10)	11(8)	29(8)	22(7)	
C68	203(12)	258(14)	123(6)	-17(8)	-35(7)	64(9)	
C70	133(5)	145(5)	147(5)	1(4)	13(4)	47(4)	
C72	86(4)	201(6)	164(5)	-4(5)	10(4)	67(4)	
C74	169(10)	174(9)	239(12)	32(9)	-26(9)	4(7)	
C78	169(10)	174(9)	239(12)	32(9)	-26(9)	4(7)	
C80	86(4)	201(6)	164(5)	-4(5)	10(4)	67(4)	
C5	133(5)	145(5)	147(5)	1(4)	13(4)	47(4)	
C45	160(9)	319(13)	172(9)	-73(10)	20(8)	73(10)	
C47	136(6)	166(7)	321(12)	-13(9)	27(7)	73(5)	
C13	203(12)	258(14)	123(6)	-17(8)	-35(7)	64(9)	
C15	133(5)	145(5)	147(5)	1(4)	13(4)	47(4)	
C61	133(5)	145(5)	147(5)	1(4)	13(4)	47(4)	
O39	130(8)	148(9)	170(10)	22(7)	4(7)	74(7)	
O5	177(13)	244(16)	228(15)	42(12)	-42(11)	122(12)	
C83	203(12)	258(14)	123(6)	-17(8)	-35(7)	64(9)	
C84	86(4)	201(6)	164(5)	-4(5)	10(4)	67(4)	
C85	169(10)	174(9)	239(12)	32(9)	-26(9)	4(7)	
C86	160(9)	319(13)	172(9)	-73(10)	20(8)	73(10)	
C89	136(6)	166(7)	321(12)	-13(9)	27(7)	73(5)	
C90	136(6)	166(7)	321(12)	-13(9)	27(7)	73(5)	
C93	133(5)	145(5)	147(5)	1(4)	13(4)	47(4)	
C94	133(5)	145(5)	147(5)	1(4)	13(4)	47(4)	
C22	75(8)	146(12)	136(11)	-9(9)	4(8)	63(8)	


Table 0: Bond Lengths in Å for hw07_150k_new.


Atom	Atom	Length/Å	
Zr1	Zr4	3.4827(14)	
Zr1	Zr3	3.4695(13)	
Zr1	Zr2	3.5008(16)	
Zr1	O6	2.096(7)	
Zr1	O8	2.191(8)	
Zr1	O12	2.211(9)	
Zr1	O14	2.045(7)	
Zr1	O26	2.197(7)	
Zr1	O28	2.271(8)	
Zr1	O13	2.286(9)	
Zr1	O2	2.211(8)	
Zr1	C1	2.634(19)	
Zr4	Zr3	3.5370(14)	
Zr4	Zr2	3.4718(13)	
Zr4	Zr5	3.5520(14)	
Zr4	O1	2.042(7)	
Zr4	O4	2.375(8)	
Zr4	O8	2.394(7)	
Zr4	O14	2.049(7)	
Zr4	O18	2.156(10)	
Zr4	O30	2.188(10)	
Zr4	O41	2.186(8)	
Zr4	O27	2.165(9)	
Zr6	Zr3	3.4701(13)	
Zr6	Zr2	3.5064(14)	
Zr6	Zr5	3.4825(13)	
Zr6	O6	2.091(7)	
Zr6	O16	2.211(9)	
Zr6	O20	2.215(9)	
Zr6	O22	2.288(8)	
Zr6	O26	2.202(8)	
Zr6	O38	2.286(8)	
Zr6	O21	2.063(7)	
Zr6	O11	2.168(8)	
Zr6	C6	2.654(18)	
Zr3	Zr5	3.5759(15)	
Zr3	O4	2.275(7)	
Zr3	O10	2.248(8)	
Zr3	O14	2.052(7)	
Zr3	O24	2.148(9)	
Zr3	O26	2.343(7)	
Zr3	O34	2.240(8)	
Zr3	O40	2.205(9)	
Zr3	O21	2.068(7)	
Zr2	Zr5	3.4899(14)	
Zr2	O1	2.039(7)	
Zr2	O6	2.094(7)	
Zr2	O8	2.188(8)	
Zr2	O11	2.204(8)	
Zr2	O23	2.213(11)	
Zr2	O3	2.195(8)	
Zr2	O7	2.280(8)	
Zr2	O31	2.283(9)	
Zr2	C26	2.66(2)	
Zr5	O1	2.062(7)	
Zr5	O4	2.264(7)	
Zr5	O32	2.207(8)	
Zr5	O36	2.115(9)	
Zr5	O21	2.060(7)	
Zr5	O11	2.377(7)	
Zr5	O25	2.300(8)	
Zr5	O15	2.164(10)	
O10	C8	1.257(18)	
O12	C4	1.274(15)	
O16	C12	1.260(18)	
O18	C20	1.29(2)	
O20	C16	1.25(3)	
O22	C6	1.19(2)	
O24	C4	1.281(16)	
O28	C1	1.22(2)	
O30	C22	1.219(17)	
O32	C16	1.28(2)	
O36	C18	1.27(3)	
O38	C6	1.30(2)	
O40	C12	1.272(19)	
O41	C8	1.258(18)	
O23	C14	1.28(2)	
O3	C32	1.287(17)	
O25	C20	1.228(18)	
O13	C1	1.27(2)	
O27	C32	1.234(18)	
O7	C26	1.24(3)	
O29	C18	1.27(3)	
O15	C14	1.31(2)	
O31	C26	1.25(3)	
O2	C22	1.291(15)	
O33	C10	1.15(3)	
O17	C28	1.31(2)	
C1	C38	1.50(2)	
O9	C28	1.23(3)	
C4	C30	1.52(2)	
C6	C42	1.54(2)	
C8	C24	1.540(19)	
C10	O37	1.35(3)	
C10	C34	1.55(3)	
C12	C50	1.48(3)	
C14	C41	1.56(3)	
C14	C67	1.56(2)	
C16	C52	1.50(3)	
C18	C44	1.44(3)	
C20	C36	1.504(9)	
C20	C2	1.504(9)	
C24	C60	1.500(16)	
C24	C7	1.478(9)	
C26	C62	1.52(3)	
C28	C64	1.52(3)	
C30	C25	1.54(4)	
C30	C51	1.493(10)	
C32	C73	1.56(2)	
C34	C56	1.53(2)	
C34	C45	1.53(2)	
C36	C46	1.502(9)	
C36	C23	1.498(9)	
C38	C70	1.54(2)	
C38	C5	1.56(2)	
C38	C15	1.55(2)	
C38	C61	1.54(2)	
C40	C49	1.501(10)	
C40	C37	1.496(10)	
C40	C75	1.500(10)	
C40	C22	1.53(2)	
C42	C72	1.45(2)	
C42	C80	1.50(2)	
C44	C74	1.34(3)	
C44	C78	1.68(3)	
C48	C17	1.456(10)	
C48	O39	1.312(17)	
C48	O5	1.332(18)	
C50	C43	1.495(7)	
C50	C57	1.484(18)	
C50	C77	1.470(18)	
C50	C79	1.500(10)	
C52	C47	1.43(3)	
C52	C89	1.486(10)	
C58	C35	1.494(10)	
C62	C68	1.505(19)	
C62	C13	1.473(14)	
C64	C21	1.494(5)	
C64	C59	1.477(14)	
C64	C81	1.471(18)	
C72	C84	1.471(9)	
C78	C85	1.503(10)	
C41	C65	1.500(9)	
C41	C33	1.500(9)	
C21	C82	1.497(10)	
C43	C88	1.496(7)	
C45	C86	1.503(10)	
C25	C91	1.492(10)	
C13	C83	1.484(10)	
C53	C67	1.40(3)	
C27	C67	1.41(3)	
C55	C73	1.442(17)	
C7	C92	1.497(10)	
C57	C87	1.499(10)	
C15	C93	1.506(10)	
C61	C94	1.502(10)	
C63	C2	1.502(9)	
C2	C71	1.494(15)	
C17	C35	1.482(10)	
C17	C11	1.493(10)	
C9	C73	1.492(9)	
C9	C3	1.493(10)	
C75	C29	1.503(10)	
C89	C90	1.486(10)	


Table 0: Bond Angles in ° for hw07_150k_new.


Atom	Atom	Atom	Angle/°	
Zr4	Zr1	Zr2	59.62(3)	
Zr3	Zr1	Zr4	61.16(3)	
Zr3	Zr1	Zr2	90.41(3)	
O6	Zr1	Zr4	85.73(19)	
O6	Zr1	Zr3	85.71(18)	
O6	Zr1	Zr2	33.32(19)	
O6	Zr1	O8	70.0(3)	
O6	Zr1	O12	143.8(3)	
O6	Zr1	O26	69.8(3)	
O6	Zr1	O28	90.7(3)	
O6	Zr1	O13	88.7(3)	
O6	Zr1	O2	142.5(3)	
O6	Zr1	C1	90.5(5)	
O8	Zr1	Zr4	42.79(18)	
O8	Zr1	Zr3	99.98(19)	
O8	Zr1	Zr2	36.89(19)	
O8	Zr1	O12	141.6(3)	
O8	Zr1	O26	125.2(3)	
O8	Zr1	O28	134.4(3)	
O8	Zr1	O13	81.4(3)	
O8	Zr1	O2	73.6(3)	
O8	Zr1	C1	109.1(5)	
O12	Zr1	Zr4	108.2(2)	
O12	Zr1	Zr3	73.4(2)	
O12	Zr1	Zr2	163.5(2)	
O12	Zr1	O28	75.5(3)	
O12	Zr1	O13	109.6(4)	
O12	Zr1	O2	73.5(4)	
O12	Zr1	C1	91.7(5)	
O14	Zr1	Zr4	31.76(19)	
O14	Zr1	Zr3	32.18(18)	
O14	Zr1	Zr2	81.4(2)	
O14	Zr1	O6	94.8(3)	
O14	Zr1	O8	74.3(3)	
O14	Zr1	O12	83.0(3)	
O14	Zr1	O26	73.6(2)	
O14	Zr1	O28	150.4(3)	
O14	Zr1	O13	152.5(3)	
O14	Zr1	O2	83.9(3)	
O14	Zr1	C1	174.4(5)	
O26	Zr1	Zr4	98.81(19)	
O26	Zr1	Zr3	41.73(17)	
O26	Zr1	Zr2	95.1(2)	
O26	Zr1	O12	75.0(3)	
O26	Zr1	O28	81.1(3)	
O26	Zr1	O13	132.5(3)	
O26	Zr1	O2	143.1(3)	
O26	Zr1	C1	106.9(5)	
O28	Zr1	Zr4	176.2(3)	
O28	Zr1	Zr3	119.9(2)	
O28	Zr1	Zr2	116.6(3)	
O28	Zr1	O13	56.5(3)	
O28	Zr1	C1	27.6(5)	
O13	Zr1	Zr4	122.0(2)	
O13	Zr1	Zr3	173.3(2)	
O13	Zr1	Zr2	86.9(3)	
O13	Zr1	C1	28.9(5)	
O2	Zr1	Zr4	73.6(3)	
O2	Zr1	Zr3	109.7(2)	
O2	Zr1	Zr2	110.4(3)	
O2	Zr1	O28	108.7(3)	
O2	Zr1	O13	77.0(3)	
O2	Zr1	C1	92.8(5)	
C1	Zr1	Zr4	150.9(5)	
C1	Zr1	Zr3	147.4(5)	
C1	Zr1	Zr2	103.9(4)	
Zr1	Zr4	Zr3	59.23(3)	
Zr1	Zr4	Zr5	90.10(3)	
Zr3	Zr4	Zr5	60.59(3)	
Zr2	Zr4	Zr1	60.45(3)	
Zr2	Zr4	Zr3	89.77(3)	
Zr2	Zr4	Zr5	59.57(3)	
O1	Zr4	Zr1	81.5(2)	
O1	Zr4	Zr3	80.68(19)	
O1	Zr4	Zr2	31.68(19)	
O1	Zr4	Zr5	30.22(19)	
O1	Zr4	O4	68.9(3)	
O1	Zr4	O8	69.9(3)	
O1	Zr4	O14	89.5(3)	
O1	Zr4	O18	84.9(3)	
O1	Zr4	O30	142.4(3)	
O1	Zr4	O41	140.3(3)	
O1	Zr4	O27	85.3(3)	
O4	Zr4	Zr1	95.40(16)	
O4	Zr4	Zr3	39.45(17)	
O4	Zr4	Zr2	95.19(16)	
O4	Zr4	Zr5	38.89(17)	
O4	Zr4	O8	121.2(2)	
O8	Zr4	Zr1	38.46(18)	
O8	Zr4	Zr3	94.18(17)	
O8	Zr4	Zr2	38.56(19)	
O8	Zr4	Zr5	94.62(18)	
O14	Zr4	Zr1	31.67(19)	
O14	Zr4	Zr3	30.43(19)	
O14	Zr4	Zr2	82.14(18)	
O14	Zr4	Zr5	81.7(2)	
O14	Zr4	O4	69.7(3)	
O14	Zr4	O8	69.9(3)	
O14	Zr4	O18	142.3(3)	
O14	Zr4	O30	83.7(3)	
O14	Zr4	O41	83.5(3)	
O14	Zr4	O27	142.0(3)	
O18	Zr4	Zr1	164.9(3)	
O18	Zr4	Zr3	112.1(3)	
O18	Zr4	Zr2	109.5(3)	
O18	Zr4	Zr5	74.8(3)	
O18	Zr4	O4	73.5(3)	
O18	Zr4	O8	140.1(3)	
O18	Zr4	O30	121.6(4)	
O18	Zr4	O41	77.6(4)	
O18	Zr4	O27	74.7(4)	
O30	Zr4	Zr1	73.5(2)	
O30	Zr4	Zr3	108.5(3)	
O30	Zr4	Zr2	110.7(2)	
O30	Zr4	Zr5	163.5(2)	
O30	Zr4	O4	139.9(3)	
O30	Zr4	O8	73.1(3)	
O41	Zr4	Zr1	109.2(3)	
O41	Zr4	Zr3	73.7(2)	
O41	Zr4	Zr2	163.5(2)	
O41	Zr4	Zr5	110.2(2)	
O41	Zr4	O4	72.1(3)	
O41	Zr4	O8	140.6(3)	
O41	Zr4	O30	75.7(3)	
O27	Zr4	Zr1	110.5(3)	
O27	Zr4	Zr3	163.6(2)	
O27	Zr4	Zr2	73.9(2)	
O27	Zr4	Zr5	109.4(3)	
O27	Zr4	O4	140.3(4)	
O27	Zr4	O8	73.0(3)	
O27	Zr4	O30	77.9(4)	
O27	Zr4	O41	122.7(3)	
Zr3	Zr6	Zr2	90.30(3)	
Zr3	Zr6	Zr5	61.90(3)	
Zr5	Zr6	Zr2	59.91(3)	
O6	Zr6	Zr3	85.76(17)	
O6	Zr6	Zr2	33.1(2)	
O6	Zr6	Zr5	86.25(19)	
O6	Zr6	O16	142.5(3)	
O6	Zr6	O20	143.4(3)	
O6	Zr6	O22	89.2(3)	
O6	Zr6	O26	69.8(3)	
O6	Zr6	O38	88.6(3)	
O6	Zr6	O11	70.0(3)	
O6	Zr6	C6	90.9(5)	
O16	Zr6	Zr3	73.8(3)	
O16	Zr6	Zr2	164.0(2)	
O16	Zr6	Zr5	109.8(2)	
O16	Zr6	O20	74.1(4)	
O16	Zr6	O22	108.4(3)	
O16	Zr6	O38	75.8(3)	
O16	Zr6	C6	91.6(5)	
O20	Zr6	Zr3	111.0(2)	
O20	Zr6	Zr2	111.9(3)	
O20	Zr6	Zr5	74.9(2)	
O20	Zr6	O22	75.2(3)	
O20	Zr6	O38	108.1(3)	
O20	Zr6	C6	89.2(5)	
O22	Zr6	Zr3	173.8(3)	
O22	Zr6	Zr2	87.5(2)	
O22	Zr6	Zr5	121.5(3)	
O22	Zr6	C6	26.5(5)	
O26	Zr6	Zr3	41.75(18)	
O26	Zr6	Zr2	94.80(18)	
O26	Zr6	Zr5	99.64(18)	
O26	Zr6	O16	74.1(3)	
O26	Zr6	O20	143.4(3)	
O26	Zr6	O22	132.7(3)	
O26	Zr6	O38	81.0(3)	
O26	Zr6	C6	109.2(6)	
O38	Zr6	Zr3	120.3(3)	
O38	Zr6	Zr2	114.4(2)	
O38	Zr6	Zr5	174.3(2)	
O38	Zr6	O22	55.8(4)	
O38	Zr6	C6	29.4(5)	
O21	Zr6	Zr3	32.9(2)	
O21	Zr6	Zr2	82.39(19)	
O21	Zr6	Zr5	32.3(2)	
O21	Zr6	O6	96.2(2)	
O21	Zr6	O16	83.5(3)	
O21	Zr6	O20	84.5(3)	
O21	Zr6	O22	152.0(4)	
O21	Zr6	O26	74.5(3)	
O21	Zr6	O38	151.4(3)	
O21	Zr6	O11	74.3(3)	
O21	Zr6	C6	172.9(5)	
O11	Zr6	Zr3	99.84(18)	
O11	Zr6	Zr2	37.0(2)	
O11	Zr6	Zr5	42.24(19)	
O11	Zr6	O16	143.4(3)	
O11	Zr6	O20	75.1(3)	
O11	Zr6	O22	81.9(3)	
O11	Zr6	O26	125.0(3)	
O11	Zr6	O38	133.1(3)	
O11	Zr6	C6	107.2(6)	
C6	Zr6	Zr3	149.7(5)	
C6	Zr6	Zr2	103.1(4)	
C6	Zr6	Zr5	148.0(5)	
Zr1	Zr3	Zr4	59.60(3)	
Zr1	Zr3	Zr6	60.85(3)	
Zr1	Zr3	Zr5	89.92(3)	
Zr4	Zr3	Zr5	59.91(3)	
Zr6	Zr3	Zr4	89.72(3)	
Zr6	Zr3	Zr5	59.22(3)	
O4	Zr3	Zr1	97.68(19)	
O4	Zr3	Zr4	41.54(19)	
O4	Zr3	Zr6	94.95(17)	
O4	Zr3	Zr5	37.91(17)	
O4	Zr3	O26	122.4(3)	
O10	Zr3	Zr1	105.9(2)	
O10	Zr3	Zr4	72.2(2)	
O10	Zr3	Zr6	161.7(2)	
O10	Zr3	Zr5	111.3(2)	
O10	Zr3	O4	73.5(3)	
O10	Zr3	O26	137.5(3)	
O14	Zr3	Zr1	32.06(18)	
O14	Zr3	Zr4	30.4(2)	
O14	Zr3	Zr6	82.44(18)	
O14	Zr3	Zr5	81.1(2)	
O14	Zr3	O4	71.8(3)	
O14	Zr3	O10	80.5(3)	
O14	Zr3	O24	85.0(3)	
O14	Zr3	O26	70.4(2)	
O14	Zr3	O34	143.7(3)	
O14	Zr3	O40	143.7(3)	
O14	Zr3	O21	91.3(3)	
O24	Zr3	Zr1	74.6(2)	
O24	Zr3	Zr4	109.6(3)	
O24	Zr3	Zr6	112.1(2)	
O24	Zr3	Zr5	164.5(2)	
O24	Zr3	O4	141.7(3)	
O24	Zr3	O10	73.0(3)	
O24	Zr3	O26	74.3(3)	
O24	Zr3	O34	112.4(3)	
O24	Zr3	O40	79.0(4)	
O26	Zr3	Zr1	38.62(16)	
O26	Zr3	Zr4	94.49(17)	
O26	Zr3	Zr6	38.75(19)	
O26	Zr3	Zr5	94.35(19)	
O34	Zr3	Zr1	172.8(2)	
O34	Zr3	Zr4	114.8(2)	
O34	Zr3	Zr6	116.3(3)	
O34	Zr3	Zr5	83.1(3)	
O34	Zr3	O4	75.7(3)	
O34	Zr3	O10	75.2(4)	
O34	Zr3	O26	143.5(3)	
O40	Zr3	Zr1	111.7(2)	
O40	Zr3	Zr4	163.6(3)	
O40	Zr3	Zr6	74.0(2)	
O40	Zr3	Zr5	108.5(3)	
O40	Zr3	O4	136.4(3)	
O40	Zr3	O10	124.2(3)	
O40	Zr3	O26	74.0(3)	
O40	Zr3	O34	72.5(3)	
O21	Zr3	Zr1	83.79(19)	
O21	Zr3	Zr4	81.57(18)	
O21	Zr3	Zr6	32.82(19)	
O21	Zr3	Zr5	29.89(19)	
O21	Zr3	O4	67.8(3)	
O21	Zr3	O10	141.0(3)	
O21	Zr3	O24	144.6(3)	
O21	Zr3	O26	71.4(3)	
O21	Zr3	O34	91.0(3)	
O21	Zr3	O40	83.7(3)	
Zr4	Zr2	Zr1	59.93(3)	
Zr4	Zr2	Zr5	61.36(3)	
Zr5	Zr2	Zr1	90.83(3)	
O1	Zr2	Zr1	81.1(2)	
O1	Zr2	Zr4	31.7(2)	
O1	Zr2	Zr5	31.88(19)	
O1	Zr2	O6	94.2(3)	
O1	Zr2	O8	74.4(3)	
O1	Zr2	O11	73.6(3)	
O1	Zr2	O23	84.2(3)	
O1	Zr2	O3	84.4(3)	
O1	Zr2	O7	152.0(4)	
O1	Zr2	O31	151.9(3)	
O1	Zr2	C26	176.5(7)	
O6	Zr2	Zr1	33.36(18)	
O6	Zr2	Zr4	86.05(17)	
O6	Zr2	Zr5	86.02(18)	
O6	Zr2	O8	70.1(3)	
O6	Zr2	O11	69.2(3)	
O6	Zr2	O23	143.6(3)	
O6	Zr2	O3	143.2(3)	
O6	Zr2	O7	89.1(3)	
O6	Zr2	O31	88.7(3)	
O6	Zr2	C26	89.3(7)	
O8	Zr2	Zr1	36.96(19)	
O8	Zr2	Zr4	43.00(19)	
O8	Zr2	Zr5	100.37(18)	
O8	Zr2	O11	125.2(3)	
O8	Zr2	O23	142.1(3)	
O8	Zr2	O3	74.1(3)	
O8	Zr2	O7	132.2(4)	
O8	Zr2	O31	80.4(3)	
O8	Zr2	C26	106.9(8)	
O11	Zr2	Zr1	94.7(2)	
O11	Zr2	Zr4	99.21(18)	
O11	Zr2	Zr5	42.25(18)	
O11	Zr2	O23	75.6(4)	
O11	Zr2	O7	81.7(4)	
O11	Zr2	O31	132.7(3)	
O11	Zr2	C26	107.8(8)	
O23	Zr2	Zr1	164.2(3)	
O23	Zr2	Zr4	108.7(2)	
O23	Zr2	Zr5	73.6(3)	
O23	Zr2	O7	76.9(4)	
O23	Zr2	O31	109.5(4)	
O23	Zr2	C26	93.0(7)	
O3	Zr2	Zr1	111.0(3)	
O3	Zr2	Zr4	73.6(2)	
O3	Zr2	Zr5	109.1(2)	
O3	Zr2	O11	143.1(3)	
O3	Zr2	O23	73.0(4)	
O3	Zr2	O7	109.0(4)	
O3	Zr2	O31	76.9(4)	
O3	Zr2	C26	92.8(7)	
O7	Zr2	Zr1	114.5(3)	
O7	Zr2	Zr4	174.4(3)	
O7	Zr2	Zr5	121.2(3)	
O7	Zr2	O31	55.8(4)	
O7	Zr2	C26	27.8(7)	
O31	Zr2	Zr1	86.3(3)	
O31	Zr2	Zr4	121.1(3)	
O31	Zr2	Zr5	174.0(3)	
O31	Zr2	C26	27.9(7)	
C26	Zr2	Zr1	101.9(7)	
C26	Zr2	Zr4	148.9(8)	
C26	Zr2	Zr5	148.9(8)	
Zr4	Zr5	Zr3	59.50(3)	
Zr6	Zr5	Zr4	89.28(3)	
Zr6	Zr5	Zr3	58.88(3)	
Zr6	Zr5	Zr2	60.38(3)	
Zr2	Zr5	Zr4	59.07(3)	
Zr2	Zr5	Zr3	88.85(3)	
O1	Zr5	Zr4	29.89(19)	
O1	Zr5	Zr6	81.03(19)	
O1	Zr5	Zr3	79.4(2)	
O1	Zr5	Zr2	31.48(18)	
O1	Zr5	O4	70.9(3)	
O1	Zr5	O32	141.1(3)	
O1	Zr5	O36	146.2(3)	
O1	Zr5	O11	69.6(3)	
O1	Zr5	O25	79.3(3)	
O1	Zr5	O15	85.9(4)	
O4	Zr5	Zr4	41.20(19)	
O4	Zr5	Zr6	94.84(18)	
O4	Zr5	Zr3	38.14(17)	
O4	Zr5	Zr2	96.84(19)	
O4	Zr5	O11	121.3(3)	
O4	Zr5	O25	73.0(3)	
O32	Zr5	Zr4	162.1(2)	
O32	Zr5	Zr6	72.8(2)	
O32	Zr5	Zr3	109.2(3)	
O32	Zr5	Zr2	109.7(2)	
O32	Zr5	O4	138.3(3)	
O32	Zr5	O11	72.1(3)	
O32	Zr5	O25	127.3(4)	
O36	Zr5	Zr4	118.7(3)	
O36	Zr5	Zr6	120.9(3)	
O36	Zr5	Zr3	90.3(3)	
O36	Zr5	Zr2	177.7(3)	
O36	Zr5	O4	81.2(3)	
O36	Zr5	O32	72.6(3)	
O36	Zr5	O11	143.7(3)	
O36	Zr5	O25	74.6(4)	
O36	Zr5	O15	106.1(4)	
O21	Zr5	Zr4	81.28(18)	
O21	Zr5	Zr6	32.39(18)	
O21	Zr5	Zr3	30.03(18)	
O21	Zr5	Zr2	82.87(19)	
O21	Zr5	O1	89.6(3)	
O21	Zr5	O4	68.2(2)	
O21	Zr5	O32	83.5(3)	
O21	Zr5	O36	97.5(3)	
O21	Zr5	O11	70.0(3)	
O21	Zr5	O25	141.1(3)	
O21	Zr5	O15	143.8(3)	
O11	Zr5	Zr4	93.72(19)	
O11	Zr5	Zr6	37.81(19)	
O11	Zr5	Zr3	93.0(2)	
O11	Zr5	Zr2	38.58(18)	
O25	Zr5	Zr4	70.6(2)	
O25	Zr5	Zr6	159.4(3)	
O25	Zr5	Zr3	111.1(2)	
O25	Zr5	Zr2	103.7(2)	
O25	Zr5	O11	136.2(3)	
O15	Zr5	Zr4	109.6(3)	
O15	Zr5	Zr6	111.6(3)	
O15	Zr5	Zr3	163.5(3)	
O15	Zr5	Zr2	74.7(3)	
O15	Zr5	O4	141.7(3)	
O15	Zr5	O32	77.9(4)	
O15	Zr5	O11	74.8(3)	
O15	Zr5	O25	73.0(3)	
Zr4	O1	Zr5	119.9(3)	
Zr2	O1	Zr4	116.6(4)	
Zr2	O1	Zr5	116.6(3)	
Zr3	O4	Zr4	99.0(3)	
Zr5	O4	Zr4	99.9(3)	
Zr5	O4	Zr3	104.0(3)	
Zr6	O6	Zr1	114.1(3)	
Zr6	O6	Zr2	113.8(3)	
Zr2	O6	Zr1	113.3(3)	
Zr1	O8	Zr4	98.8(3)	
Zr2	O8	Zr1	106.1(3)	
Zr2	O8	Zr4	98.4(3)	
C8	O10	Zr3	133.6(9)	
C4	O12	Zr1	132.9(9)	
Zr1	O14	Zr4	116.6(3)	
Zr1	O14	Zr3	115.8(3)	
Zr4	O14	Zr3	119.2(3)	
C12	O16	Zr6	133.9(9)	
C20	O18	Zr4	134.3(8)	
C16	O20	Zr6	131.9(12)	
C6	O22	Zr6	94.3(12)	
C4	O24	Zr3	133.8(9)	
Zr1	O26	Zr6	106.0(3)	
Zr1	O26	Zr3	99.6(3)	
Zr6	O26	Zr3	99.5(3)	
C1	O28	Zr1	93.0(11)	
C22	O30	Zr4	134.9(8)	
C16	O32	Zr5	134.6(13)	
C18	O36	Zr5	147.8(16)	
C6	O38	Zr6	91.1(11)	
C12	O40	Zr3	133.6(9)	
C8	O41	Zr4	134.2(8)	
Zr6	O21	Zr3	114.3(3)	
Zr5	O21	Zr6	115.3(3)	
Zr5	O21	Zr3	120.1(3)	
Zr6	O11	Zr2	106.6(4)	
Zr6	O11	Zr5	100.0(3)	
Zr2	O11	Zr5	99.2(3)	
C14	O23	Zr2	133.2(10)	
C32	O3	Zr2	131.8(9)	
C20	O25	Zr5	135.5(10)	
C1	O13	Zr1	90.9(10)	
C32	O27	Zr4	134.1(8)	
C26	O7	Zr2	93.2(14)	
C14	O15	Zr5	133.1(11)	
C26	O31	Zr2	92.9(14)	
C22	O2	Zr1	131.9(9)	
O28	C1	Zr1	59.4(9)	
O28	C1	O13	119.6(16)	
O28	C1	C38	124.3(18)	
O13	C1	Zr1	60.2(9)	
O13	C1	C38	116.0(17)	
C38	C1	Zr1	172.4(14)	
O12	C4	O24	125.0(14)	
O12	C4	C30	120.1(15)	
O24	C4	C30	114.9(14)	
O22	C6	Zr6	59.3(9)	
O22	C6	O38	118.3(15)	
O22	C6	C42	127(2)	
O38	C6	Zr6	59.4(8)	
O38	C6	C42	114.6(19)	
C42	C6	Zr6	171.8(15)	
O10	C8	O41	125.9(11)	
O10	C8	C24	114.6(16)	
O41	C8	C24	119.4(15)	
O33	C10	O37	125(3)	
O33	C10	C34	120(3)	
O37	C10	C34	115(2)	
O16	C12	O40	124.7(12)	
O16	C12	C50	122.6(17)	
O40	C12	C50	112.7(17)	
O23	C14	O15	125.3(15)	
O23	C14	C41	117(2)	
O23	C14	C67	119(2)	
O15	C14	C41	116(2)	
O15	C14	C67	115(2)	
O20	C16	O32	126(2)	
O20	C16	C52	120(2)	
O32	C16	C52	112(2)	
O36	C18	C44	124(2)	
O29	C18	O36	122(2)	
O29	C18	C44	114(2)	
O18	C20	C36	119(2)	
O18	C20	C2	108(2)	
O25	C20	O18	124.4(12)	
O25	C20	C36	116(2)	
O25	C20	C2	127(2)	
C60	C24	C8	114.5(17)	
C7	C24	C8	109.5(18)	
C7	C24	C60	112.4(16)	
O7	C26	Zr2	59.0(10)	
O7	C26	O31	118.0(19)	
O7	C26	C62	118(3)	
O31	C26	Zr2	59.1(10)	
O31	C26	C62	124(3)	
C62	C26	Zr2	177(2)	
O17	C28	C64	109(2)	
O9	C28	O17	126.8(19)	
O9	C28	C64	123(2)	
C4	C30	C25	114(2)	
C51	C30	C4	111(2)	
C51	C30	C25	108(2)	
O3	C32	C73	113.2(16)	
O27	C32	O3	126.4(12)	
O27	C32	C73	119.7(14)	
C56	C34	C10	114(2)	
C45	C34	C10	112(2)	
C45	C34	C56	99.0(16)	
C46	C36	C20	112(3)	
C23	C36	C20	107(3)	
C23	C36	C46	115(2)	
C1	C38	C70	116.7(19)	
C1	C38	C5	108.9(19)	
C1	C38	C15	114(2)	
C1	C38	C61	112.5(19)	
C15	C38	C5	102(2)	
C61	C38	C70	97(2)	
C49	C40	C22	117(2)	
C37	C40	C75	114(2)	
C37	C40	C22	108(3)	
C75	C40	C49	127(2)	
C75	C40	C22	109.2(17)	
C72	C42	C6	108.6(15)	
C72	C42	C80	113.5(15)	
C80	C42	C6	108.8(15)	
C18	C44	C78	109(2)	
C74	C44	C18	129(2)	
C74	C44	C78	122(2)	
O39	C48	C17	137.1(19)	
O39	C48	O5	118(2)	
O5	C48	C17	103.5(15)	
C12	C50	C43	115(3)	
C12	C50	C57	117(3)	
C12	C50	C79	114(3)	
C43	C50	C79	110(2)	
C77	C50	C12	111(3)	
C77	C50	C57	120(3)	
C47	C52	C16	128(2)	
C47	C52	C89	122.8(19)	
C89	C52	C16	93(2)	
C68	C62	C26	114(2)	
C13	C62	C26	110(2)	
C13	C62	C68	114(2)	
C21	C64	C28	108.3(19)	
C59	C64	C28	112(3)	
C59	C64	C21	107(2)	
C81	C64	C28	117(3)	
C81	C64	C21	106(3)	
C42	C72	C84	121.9(15)	
C85	C78	C44	98.7(16)	
C65	C41	C14	118(4)	
C33	C41	C14	121(4)	
C33	C41	C65	103(2)	
C64	C21	C82	109.3(19)	
C50	C43	C88	106.5(8)	
C86	C45	C34	99.2(17)	
C91	C25	C30	99(2)	
C62	C13	C83	115(2)	
C24	C7	C92	116(2)	
C50	C57	C87	113(4)	
C93	C15	C38	94.4(16)	
C94	C61	C38	108(2)	
C63	C2	C20	105(2)	
C71	C2	C20	111(3)	
C71	C2	C63	110(2)	
C53	C67	C14	116(3)	
C53	C67	C27	115(3)	
C27	C67	C14	116(3)	
C48	C17	C35	114(3)	
C48	C17	C11	109.4(10)	
C35	C17	C11	107.9(9)	
C17	C35	C58	99(2)	
C73	C9	C3	97.5(16)	
C55	C73	C32	111.2(19)	
C55	C73	C9	113.3(19)	
C9	C73	C32	114(2)	
C40	C75	C29	103(2)	
C52	C89	C90	103.5(14)	
O30	C22	O2	126.1(13)	
O30	C22	C40	120.4(13)	
O2	C22	C40	113.5(15)	


Table 0: Torsion Angles in ° for hw07_150k_new.


Atom	Atom	Atom	Atom	Angle/°	
Zr1	O12	C4	O24	3(2)	
Zr1	O12	C4	C30	-176.9(12)	
Zr1	O28	C1	O13	-3.1(18)	
Zr1	O28	C1	C38	172.1(17)	
Zr1	O13	C1	O28	3.1(18)	
Zr1	O13	C1	C38	-172.5(14)	
Zr1	O2	C22	O30	2(2)	
Zr1	O2	C22	C40	-178.6(10)	
Zr4	O18	C20	O25	3(2)	
Zr4	O18	C20	C36	-177.3(19)	
Zr4	O18	C20	C2	-175.8(15)	
Zr4	O30	C22	O2	-2(3)	
Zr4	O30	C22	C40	178.6(11)	
Zr4	O41	C8	O10	-5(2)	
Zr4	O41	C8	C24	172.7(10)	
Zr4	O27	C32	O3	4(3)	
Zr4	O27	C32	C73	174.1(11)	
Zr6	O16	C12	O40	-4(2)	
Zr6	O16	C12	C50	178.9(14)	
Zr6	O20	C16	O32	1(5)	
Zr6	O20	C16	C52	163.5(15)	
Zr6	O22	C6	O38	-7.6(18)	
Zr6	O22	C6	C42	173.9(18)	
Zr6	O38	C6	O22	7.6(18)	
Zr6	O38	C6	C42	-173.7(14)	
Zr3	O10	C8	O41	-3(2)	
Zr3	O10	C8	C24	179.7(10)	
Zr3	O24	C4	O12	-8(2)	
Zr3	O24	C4	C30	172.8(12)	
Zr3	O40	C12	O16	4(2)	
Zr3	O40	C12	C50	-178.7(13)	
Zr2	O23	C14	O15	3(2)	
Zr2	O23	C14	C41	168(3)	
Zr2	O23	C14	C67	-173.3(17)	
Zr2	O3	C32	O27	-7(3)	
Zr2	O3	C32	C73	-177.4(10)	
Zr2	O7	C26	O31	2(3)	
Zr2	O7	C26	C62	180(2)	
Zr2	O31	C26	O7	-2(3)	
Zr2	O31	C26	C62	-180(2)	
Zr5	O32	C16	O20	-4(5)	
Zr5	O32	C16	C52	-167.9(12)	
Zr5	O36	C18	O29	-50(4)	
Zr5	O36	C18	C44	124(3)	
Zr5	O25	C20	O18	5(2)	
Zr5	O25	C20	C36	-174.6(18)	
Zr5	O25	C20	C2	-176.3(17)	
Zr5	O15	C14	O23	1(2)	
Zr5	O15	C14	C41	-164(3)	
Zr5	O15	C14	C67	177.2(17)	
O10	C8	C24	C60	132.9(18)	
O10	C8	C24	C7	-99.9(19)	
O12	C4	C30	C25	119.4(19)	
O12	C4	C30	C51	-3(3)	
O16	C12	C50	C43	-76(3)	
O16	C12	C50	C57	117(3)	
O16	C12	C50	C77	-25(3)	
O16	C12	C50	C79	53(3)	
O18	C20	C36	C46	-50(4)	
O18	C20	C36	C23	77(3)	
O18	C20	C2	C63	-96(3)	
O18	C20	C2	C71	146(3)	
O20	C16	C52	C47	-150(3)	
O20	C16	C52	C89	74(3)	
O22	C6	C42	C72	-80(3)	
O22	C6	C42	C80	44(3)	
O24	C4	C30	C25	-61(2)	
O24	C4	C30	C51	176.3(18)	
O28	C1	C38	C70	54(3)	
O28	C1	C38	C5	-95(2)	
O28	C1	C38	C15	18(3)	
O28	C1	C38	C61	-56(3)	
O32	C16	C52	C47	15(4)	
O32	C16	C52	C89	-121(2)	
O36	C18	C44	C74	-158(3)	
O36	C18	C44	C78	28(4)	
O38	C6	C42	C72	101(2)	
O38	C6	C42	C80	-134.7(17)	
O40	C12	C50	C43	107(2)	
O40	C12	C50	C57	-60(3)	
O40	C12	C50	C77	157(2)	
O40	C12	C50	C79	-125(3)	
O41	C8	C24	C60	-45(2)	
O41	C8	C24	C7	83(2)	
O23	C14	C41	C65	-156(4)	
O23	C14	C41	C33	-29(6)	
O23	C14	C67	C53	148(3)	
O23	C14	C67	C27	9(4)	
O3	C32	C73	C55	-109(2)	
O3	C32	C73	C9	121.4(19)	
O25	C20	C36	C46	130(3)	
O25	C20	C36	C23	-103(3)	
O25	C20	C2	C63	86(3)	
O25	C20	C2	C71	-33(4)	
O13	C1	C38	C70	-131(2)	
O13	C1	C38	C5	80(2)	
O13	C1	C38	C15	-166.7(17)	
O13	C1	C38	C61	119(2)	
O27	C32	C73	C55	80(3)	
O27	C32	C73	C9	-50(3)	
O7	C26	C62	C68	-123(3)	
O7	C26	C62	C13	108(3)	
O29	C18	C44	C74	17(5)	
O29	C18	C44	C78	-157(2)	
O15	C14	C41	C65	11(6)	
O15	C14	C41	C33	138(4)	
O15	C14	C67	C53	-29(4)	
O15	C14	C67	C27	-167(3)	
O31	C26	C62	C68	55(4)	
O31	C26	C62	C13	-74(3)	
O33	C10	C34	C56	-112(4)	
O33	C10	C34	C45	137(4)	
O17	C28	C64	C21	75(2)	
O17	C28	C64	C59	-167(2)	
O17	C28	C64	C81	-45(3)	
C1	C38	C15	C93	73(3)	
C1	C38	C61	C94	-58(3)	
O9	C28	C64	C21	-115(2)	
O9	C28	C64	C59	3(3)	
O9	C28	C64	C81	125(3)	
C4	C30	C25	C91	-66(3)	
C6	C42	C72	C84	-46(3)	
C8	C24	C7	C92	54(3)	
C10	C34	C45	C86	-56(3)	
C12	C50	C43	C88	68(4)	
C12	C50	C57	C87	-178(4)	
O37	C10	C34	C56	60(4)	
O37	C10	C34	C45	-51(4)	
C16	C52	C89	C90	90(2)	
C18	C44	C78	C85	-99(2)	
C26	C62	C13	C83	-42(3)	
C28	C64	C21	C82	65(3)	
C48	C17	C35	C58	58(3)	
C56	C34	C45	C86	-177.3(19)	
C60	C24	C7	C92	-178(2)	
C68	C62	C13	C83	-172(2)	
C70	C38	C61	C94	179(3)	
C74	C44	C78	C85	87(3)	
C80	C42	C72	C84	-167.5(17)	
C5	C38	C15	C93	-170(2)	
C47	C52	C89	C90	-50(3)	
C49	C40	C75	C29	-57(3)	
C49	C40	C22	O30	166(2)	
C49	C40	C22	O2	-13(3)	
C51	C30	C25	C91	58(3)	
C59	C64	C21	C82	-55(3)	
O39	C48	C17	C35	71(3)	
O39	C48	C17	C11	-50(5)	
O5	C48	C17	C35	-125(2)	
O5	C48	C17	C11	114(2)	
C37	C40	C75	C29	32(4)	
C37	C40	C22	O30	83(3)	
C37	C40	C22	O2	-96(3)	
C75	C40	C22	O30	-41(2)	
C75	C40	C22	O2	139.5(18)	
C77	C50	C57	C87	-39(6)	
C79	C50	C43	C88	-63(5)	
C81	C64	C21	C82	-169(3)	
C11	C17	C35	C58	180(2)	
C22	C40	C75	C29	153(2)	
C3	C9	C73	C32	-95(2)	
C3	C9	C73	C55	136(2)	


Table 0: Hydrogen Fractional Atomic Coordinates (×104) and Equivalent Isotropic Displacement Parameters (Å2×103) for hw07_150k_new. Ueq is defined as 1/3 of the trace of the orthogonalised Uij.

Atom	x	y	z	Ueq	
H4	5314.73	4386.26	8714.18	130	
H8	3697.81	1804.95	6688.73	136	
H26	1304.58	1782.32	8350.44	129	
H34A	4526.73	4648.74	9472.6	165	
H34B	3886.05	4155.22	9798.2	165	
H11	2218.08	4613.05	7054.25	141	
H17	-710	1253.5	7917.73	262	
H24	5972.82	2343.91	9710.84	182	
H30	1949.14	196.03	9426.89	215	
H34	4429.86	493.63	5266.34	270	
H36	7424.12	6448.96	8046.39	230	
H38	324.93	-682.67	6199.79	172	
H38A	57.97	-457.14	6160.81	172	
H40	5225	222.97	7734.18	179	
H40A	4253.73	-361.22	7106.92	179	
H42	-1745.68	1641.87	7041.93	178	
H44	5612.19	7084.85	9560.16	254	
H50	1297.42	3683.92	9815.98	240	
H52	965.88	6036.41	7740.68	246	
H56A	2467.59	310.34	4646	337	
H56B	3441.98	1221.08	4712.15	337	
H56C	3468.44	294.21	4396.95	337	
H58A	1485.24	5864.39	5407.94	375	
H58B	674	6312.49	5174.04	375	
H58C	1824.17	6945.7	5395.05	375	
H60A	6259.84	1331.7	9074.06	337	
H60B	7078.45	2135.71	8854.69	337	
H60C	7303.94	1848.4	9452.62	337	
H62	471.02	2681.02	5287.22	246	
H64	-1874.59	2.58	8788.09	119	
H64A	-875.01	-188.98	9107.86	119	
H68A	-113.52	1279.74	5613.88	307	
H68B	-331.42	1116.94	4955.12	307	
H68C	611.48	949.73	5273.78	307	
H70A	-1101.42	-308.8	6829.24	216	
H70B	-704.02	13.1	6269.64	216	
H70C	-1609.33	-905.72	6253.05	216	
H72A	-1855.46	3403.99	7053.69	178	
H72B	-2720.3	2445.98	7116.21	178	
H74A	6903.5	7240.46	9852.38	318	
H74B	7278.75	7367.22	9250.28	318	
H74C	6991.61	8157.94	9573.34	318	
H78A	5395.77	8341.61	9160.61	254	
H78B	4451.68	7412.36	8940.1	254	
H80A	-2104.77	2501.2	6193.87	222	
H80B	-1712.6	1651.67	6148.59	222	
H80C	-916.07	2671.37	6222.41	222	
H5A	-91.17	-1949.43	6396.64	216	
H5B	1048.22	-1365.91	6612.23	216	
H5C	247.83	-1553.33	7032.22	216	
H41	4014.86	6371.9	6061.32	195	
H21A	-1634.72	-880.68	7986.82	240	
H21B	-2126.68	-1536.62	8445.95	240	
H43A	1383.51	4635.88	9881.97	240	
H43B	339.15	4035.1	10089.71	240	
H45A	3476.3	-932.75	4692.18	270	
H45B	2519.79	-1029.8	5026.68	270	
H47A	2869.76	7324.04	7981.46	308	
H47B	2104.05	6968.53	7422.42	308	
H47C	1883.14	7581.36	7900.04	308	
H49A	3594.51	-965.88	7642.42	237	
H49B	3738.98	-788.68	7019.35	237	
H49C	4409.39	-1252.57	7359.41	237	
H25A	3726.02	820.38	9713.97	365	
H25B	3333.93	-239.44	9826.72	365	
H51A	1318.75	-858.71	8634.27	456	
H51B	2428.86	-875.98	8567.02	456	
H51C	1917.19	-1236.27	9105.68	456	
H13A	1754.94	2007.83	4786.34	246	
H13B	879.2	2265.82	4417.46	246	
H55A	6170.75	2187.52	6050.02	375	
H55B	5270.47	2037.84	5545.4	375	
H55C	6422.48	2558.78	5461.57	375	
H7A	7654.16	3710.47	9393.5	270	
H7B	7473.46	3287.54	9982.39	270	
H57A	28.23	1877.88	9564.28	240	
H57B	1056.65	2285.73	10012.28	240	
H59A	-785.01	254.87	9532.24	300	
H59B	-1143.39	-811.44	9411.78	300	
H59C	-54.69	-214.43	9308.02	300	
H15A	-1033.04	-251.02	6631.46	216	
H15B	-963.77	-911.5	7157.07	172	
H15C	-1478.58	-1326.39	6517.57	172	
H61A	-651.66	-1923.2	6589.75	172	
H61B	-505.45	-1391.04	7211.42	172	
H2	7840.52	5283.86	7704.37	230	
H67	5007.54	6514.05	6363.07	190	
H17A	-191.56	5345.77	5678.41	300	
H35A	1224.29	6688.23	6209.54	300	
H35B	379.65	7063.4	5910.6	300	
H9A	7459.16	3883.42	5969.22	300	
H9B	7076.09	4259.05	6514.01	300	
H73	5531.22	3529.95	5625.4	201	
H5	655.05	4051.29	6104.73	313	
H37A	5853.42	-104.38	7968.82	379	
H37B	5046.28	253.24	8237.78	379	
H37C	4681.37	-735.44	7885.25	379	
H75A	6078.97	1017.3	7108.25	303	
H75B	5399.58	310.32	6574.01	303	
H77A	376.82	4311.76	9343.81	300	
H77B	-536.96	3374.32	9132.71	300	
H77C	-312.53	3781.69	9781.86	300	
H79A	-690.27	2444.52	9086.86	300	
H79B	-33.47	2085.24	9516.7	300	
H79C	-809.97	2511.42	9726.76	300	
H81A	-2136.14	535.73	9069.5	300	
H81B	-2611.3	-60.15	8472.66	300	
H81C	-2563.2	-554.97	9029.4	300	
H37	2137.3	331.1	6097	700(500)	
H82A	-370.95	-1245.46	8754.27	300	
H82B	-52.42	-868.77	8176.68	300	
H82C	-750.75	-1911.45	8176.68	300	
H83A	2701.72	3528.08	5122.97	307	
H83B	2520.42	3333.72	4450.82	307	
H83C	1831.43	3787.75	4764.37	307	
H84A	-1129.48	3474.75	7954.05	222	
H84B	-2300.46	3375.31	7929.93	222	
H84C	-1956.05	2510.68	8018.27	222	
H85A	5369.91	8322.43	8188.55	318	
H85B	6345.32	8189.04	8525.97	318	
H85C	5436.44	7321.71	8202.62	318	
H86A	4021.52	-1483.57	5439.08	337	
H86B	4171.58	-543.99	5809.34	337	
H86C	3170.34	-1411.23	5809.34	337	
H87A	-783.05	2566.52	10153.78	374	
H87B	-365.44	1890.77	10493.73	374	
H87C	234.78	2967.92	10597.62	374	
H88A	159.32	4209.46	8922.05	375	
H88B	-609.82	4215.84	9362.49	375	
H88C	380.72	5093.3	9362.49	375	
H89A	898.77	5498.65	8606.9	246	
H89B	756.59	6478.35	8568.66	246	
H90A	1891.19	6740.07	9324.81	308	
H90B	2632.05	6425.36	8965.74	308	
H90C	2385.28	7329.21	8846.74	308	
H91A	3611.02	-618.55	8879.58	360	
H91B	4087.78	445.26	8814.13	360	
H91C	4654.84	-2.56	9258.42	360	
H92A	6464.13	4375	9569	337	
H92B	6285.21	3956.31	10151.9	337	
H92C	7344.23	4723.31	10098.28	337	
H93A	-634.78	327.56	6322.52	216	
H93B	-1832.8	-76.69	6336.19	216	
H93C	-1073.25	394.75	6903.35	216	
H94A	641.28	-2227.35	7174.04	216	
H94B	1085.35	-1663.59	6675.44	216	
H94C	1222.94	-1148.22	7293.5	216	
H11A	116.56	5748.29	6827.61	375	
H11B	-712.71	6113.19	6516.97	375	
H11C	-919.8	5039.42	6488.49	375	
H29A	6348.47	-510.4	6796.96	375	
H29B	6924.11	132.9	7366.1	375	
H29C	5856.55	-658.62	7366.1	375	
H3A	6571.76	4605.03	5448.74	375	
H3B	6002.07	4843.44	5945.36	375	
H3C	7181.15	5399.5	5945.36	375	


Table 0: Atomic Occupancies for all atoms that are not fully occupied in hw07_150k_new.


Atom	Occupancy	
O33	0.8	
C10	0.8	
O37	0.8	
C36	0.5	
H36	0.5	
H38	0.5	
H38A	0.5	
H40	0.5	
H40A	0.5	
C46	0.5	
H50	0.5	
C64	0.5	
H64	0.5	
H64A	0.5	
C70	0.5	
H70A	0.5	
H70B	0.5	
H70C	0.5	
C5	0.5	
H5A	0.5	
H5B	0.5	
H5C	0.5	
C41	0.35	
H41	0.35	
H21A	0.5	
H21B	0.5	
C43	0.5	
H43A	0.5	
H43B	0.5	
C23	0.5	
C49	0.5	
H49A	0.5	
H49B	0.5	
H49C	0.5	
C53	0.65	
C27	0.65	
C57	0.5	
H57A	0.5	
H57B	0.5	
C59	0.5	
H59A	0.5	
H59B	0.5	
H59C	0.5	
C15	0.5	
H15A	0.5	
H15B	0.5	
H15C	0.5	
C61	0.5	
H61A	0.5	
H61B	0.5	
C63	0.5	
C2	0.5	
H2	0.5	
C65	0.35	
C33	0.35	
C67	0.65	
H67	0.65	
C71	0.5	
C37	0.5	
H37A	0.5	
H37B	0.5	
H37C	0.5	
C77	0.5	
H77A	0.5	
H77B	0.5	
H77C	0.5	
C79	0.5	
H79A	0.5	
H79B	0.5	
H79C	0.5	
C81	0.5	
H81A	0.5	
H81B	0.5	
H81C	0.5	
H37	0.8	
C87	0.5	
H87A	0.5	
H87B	0.5	
H87C	0.5	
C88	0.5	
H88A	0.5	
H88B	0.5	
H88C	0.5	
C93	0.5	
H93A	0.5	
H93B	0.5	
H93C	0.5	
C94	0.5	
H94A	0.5	
H94B	0.5	
H94C	0.5	


Citations
O.V. Dolomanov and L.J. Bourhis and R.J. Gildea and J.A.K. Howard and H. Puschmann, Olex2: A complete structure solution, refinement and analysis program, J. Appl. Cryst., (2009), 42, 339-341.
STOE & Cie GmbH, X-Area, software package for collecting single-crystal or multi-domain crystal data on STOE area-detector diffractometers, for image  processing, for the correction and scaling of reflection intensities and  for outlier rejection, version 1.88, Darmstadt 2019
Sheldrick, G.M., Crystal structure refinement with ShelXL, Acta Cryst., (2015), C71, 3-8.
Sheldrick, G.M., ShelXT-Integrated space-group and crystal-structure determination, Acta Cryst., (2015), A71, 3-8.
X-Area Integrate 1.77.0.0 (STOE, 2019) X-Area LANA 1.77.1.0 (STOE, 2019) X-Area X-Red32 1.65.0.0 (STOE, 2018)
X-Area Pilatus3_SV 1.31.186.0 (STOE, 2022)
X-Area Recipe 1.37.0.0 (STOE, 2021)
