# Supplementary material for: Mechanism‐Guided Precision Hydrolysis of Early Transition Metals to Access (Mixed‐Metal) Oxo Clusters
Source: Angew Chem Int Ed Engl. 2026 Feb 24;65(15):e25769. doi: 10.1002/anie.202525769 (PMC13053926; doi:10.1002/anie.202525769)

checkCIF/PLATON report

Structure factors have been supplied for datablock(s) hw07_150k_new

THIS REPORT IS FOR GUIDANCE ONLY. IF USED AS PART OF A REVIEW PROCEDURE FOR PUBLICATION, IT SHOULD NOT REPLACE THE EXPERTISE OF AN EXPERIENCED CRYSTALLOGRAPHIC REFEREE.

No syntax errors found. CIF dictionary Interpreting this report

Datablock: hw07_150k_new

Bond precision: C-C = 0.0334 A Wavelength=1.34143

Cell:	a=14.1011(3)	b=15.6314(3)	c=24.0684(5)
	alpha=94.094(2)	beta=96.378(2)	gamma=109.376(2)
Temperature:	150 K		

	Calculated	Reported
Volume	4940.44(19)	4940.44(19)
Space group	P -1	P -1
Hall group	-P 1	-P 1
Moiety formula	C58 H98 O33 Zr6, C4.50 H9 O2, C5 H10 O2, C4.80 H9.80 O1.60	C58 H98 O33 Zr6, C4.8 H9.8 O1.6, C4.5 H9 O2, C5 H10 O2
Sum formula	C72.30 H126.80 O38.60 Zr6	C72.30 H126.80 O38.60 Zr6
Mr	2161.06	2161.05
Dx,g cm-3	1.453	1.453
Z	2	2
Mu (mm-1)	3.794	3.794
F000	2218.8	2219.0
F000’	2218.86	
h,k,lmax	17,19,29	17,19,29
Nref	19474	19060
Tmin,Tmax	0.492,0.634	0.000,0.016
Tmin’	0.446	

Correction method= # Reported T Limits: Tmin=0.000 Tmax=0.016
AbsCorr = MULTI-SCAN

Data completeness= 0.979   Theta(max)= 55.883

R(reflections)= 0.1244(12576)   wR2(reflections)= 0.3107(19060)

S = 1.119   Npar= 785

The following ALERTS were generated. Each ALERT has the format
test-name_ALERT_alert-type_alert-level.
Click on the hyperlinks for more details of the test.

Alert level A

```
PLAT201_ALERT_2_A Isotropic non-H Atoms in Main Residue(s) .......         17 Report
                  C3      C7      C9      C25     C27     C29     C40     C50
                  C51     C53     C55     C60     C67     C73     C75     C91
                  C92
```

Author Response: due to the large amount of disorder and thermal motion, these atoms had to be refined isotropically

```
PLAT412_ALERT_2_A Short Intra XH3 .. XHn     H51B      ..H91A      .       1.66 Ang.
                                                       x,y,z  =      1_555 Check
```

Author Response: H atoms were geomtrically placed. In a structure with a lot of disorder alert like this one are not unexpected

```
PLAT413_ALERT_2_A Short Inter XH3 .. XHn     H7B       ..H90A      .       1.81 Ang.
                                                  1-x,1-y,2-z  =      2_667 Check
```

Author Response: H atoms were geomtrically placed. In a structure with a lot of disorder alert like this one are not unexpected

---

Alert level B

```
PLAT230_ALERT_2_B Hirshfeld Test Diff for    O31       --C26       .        8.4 s.u.
```

Author Response: all these alerts are caused by either atoms with high thermal motion connected to some that are strongly held in place or viceversa

```
PLAT241_ALERT_2_B High   'MainMol' Ueq as Compared to Neighbors of       C16 Check
```

Author Response: all these alerts are caused by either atoms with high thermal motion connected to some that are strongly held in place or viceversa

```
PLAT241_ALERT_2_B High   'MainMol' Ueq as Compared to Neighbors of       C18 Check
```

Author Response: all these alerts are caused by either atoms with high thermal motion connected to some that are strongly held in place or viceversa

```
PLAT241_ALERT_2_B High   'MainMol' Ueq as Compared to Neighbors of       C25 Check
```

Author Response: all these alerts are caused by either atoms with high thermal motion connected to some that are strongly held in place or viceversa

```
PLAT241_ALERT_2_B High   'MainMol' Ueq as Compared to Neighbors of       C26 Check
```

Author Response: all these alerts are caused by either atoms with high thermal motion connected to some that are strongly held in place or viceversa

```
PLAT241_ALERT_2_B High   'MainMol' Ueq as Compared to Neighbors of       C75 Check
```

Author Response: all these alerts are caused by either atoms with high thermal motion connected to some that are strongly held in place or viceversa

```
PLAT242_ALERT_2_B Low    'MainMol' Ueq as Compared to Neighbors of       C30 Check
```

Author Response: all these alerts are caused by either atoms with high thermal motion connected to some that are strongly held in place or viceversa

```
PLAT342_ALERT_3_B Low Bond Precision on  C-C Bonds ..............    0.03341 Ang.
```

Author Response: the weak diffraction and the huge amount of disorder are most likely the cause of this alert

```
PLAT910_ALERT_3_B Missing FCF Reflection(s) Below Theta(Min)[Deg]=       3.93 Note
                1  0  0,  -1  1  0,   0  1  0,   0 -1  1,   1 -1  1,  -1  0  1,
                0  0  1,   1  0  1,  -1  1  1,   0  1  1,   0  0  2,
```

Author Response: these are likely either hidden by the beam stop or outliers

---

Alert level C

```
PLAT042_ALERT_1_C Calc. and Reported MoietyFormula Strings  Differ     Please Check
              Calc: C58 H98 O33 Zr6, C4.50 H9 O2, C5 H10 O2, C4.80
                     H9.80 O1.60
              Rep.: C58 H98 O33 Zr6, C4.8 H9.8 O1.6, C4.5 H9 O2, C
                    5 H10 O2
PLAT082_ALERT_2_C High R1 Value .................................       0.12 Report
PLAT084_ALERT_3_C High wR2 Value (i.e. > 0.25) ...................       0.31 Report
PLAT202_ALERT_3_C Isotropic non-H Atoms in Anion/Solvent .........          7 Check
              C21     C82     C11     C17     C35     C48     C58
PLAT230_ALERT_2_C Hirshfeld Test Diff for    O13      --C1       .        6.0 s.u.
```

Author Response: all these alerts are caused by either atoms with high thermal motion connected to some that are strongly held in place or viceversa

```
PLAT230_ALERT_2_C Hirshfeld Test Diff for    O18      --C20      .        5.4 s.u.
```

Author Response: all these alerts are caused by either atoms with high thermal motion connected to some that are strongly held in place or viceversa

```
PLAT230_ALERT_2_C Hirshfeld Test Diff for    O28      --C1       .        6.8 s.u.
```

Author Response: all these alerts are caused by either atoms with high thermal motion connected to some that are strongly held in place or viceversa

```
PLAT230_ALERT_2_C Hirshfeld Test Diff for    O36      --C18      .        5.9 s.u.
```

Author Response: all these alerts are caused by either atoms with high thermal motion connected to some that are strongly held in place or viceversa

```
PLAT234_ALERT_4_C Large Hirshfeld Difference O7       --C26      .       0.19 Ang.
PLAT234_ALERT_4_C Large Hirshfeld Difference O10      --C8       .       0.18 Ang.
PLAT234_ALERT_4_C Large Hirshfeld Difference O29      --C18      .       0.19 Ang.
PLAT234_ALERT_4_C Large Hirshfeld Difference O32      --C16      .       0.19 Ang.
PLAT234_ALERT_4_C Large Hirshfeld Difference O40      --C12      .       0.17 Ang.
PLAT234_ALERT_4_C Large Hirshfeld Difference O41      --C8       .       0.21 Ang.
PLAT234_ALERT_4_C Large Hirshfeld Difference O33      --C10      .       0.20 Ang.
PLAT234_ALERT_4_C Large Hirshfeld Difference O37      --C10      .       0.24 Ang.
PLAT241_ALERT_2_C High   'MainMol' Ueq as Compared to Neighbors of         C1 Check
```

Author Response: all these alerts are caused by either atoms with high thermal motion connected to some that are strongly held in place or viceversa

```
PLAT241_ALERT_2_C High   'MainMol' Ueq as Compared to Neighbors of        C6 Check
```

Author Response: all these alerts are caused by either atoms with high thermal motion connected to some that are strongly held in place or viceversa

```
PLAT241_ALERT_2_C High   'MainMol' Ueq as Compared to Neighbors of        C7 Check
```

Author Response: all these alerts are caused by either atoms with high thermal motion connected to some that are strongly held in place or viceversa

```
PLAT241_ALERT_2_C High   'MainMol' Ueq as Compared to Neighbors of        C9 Check
```

Author Response: all these alerts are caused by either atoms with high thermal motion connected to some that are strongly held in place or viceversa

```
PLAT241_ALERT_2_C High   'MainMol' Ueq as Compared to Neighbors of       C50 Check
```

Author Response: all these alerts are caused by either atoms with high thermal motion connected to some that are strongly held in place or viceversa

```
PLAT242_ALERT_2_C Low    'MainMol' Ueq as Compared to Neighbors of        O7 Check
```

Author Response: all these alerts are caused by either atoms with high thermal motion connected to some that are strongly held in place or viceversa

```
PLAT242_ALERT_2_C Low    'MainMol' Ueq as Compared to Neighbors of       O20 Check
```

Author Response: all these alerts are caused by either atoms with high thermal motion connected to some that are strongly held in place or viceversa

```
PLAT242_ALERT_2_C Low    'MainMol' Ueq as Compared to Neighbors of       O31 Check
```

Author Response: all these alerts are caused by either atoms with high thermal motion connected to some that are strongly held in place or viceversa

```
PLAT242_ALERT_2_C Low    'MainMol' Ueq as Compared to Neighbors of        O32 Check
```

Author Response: all these alerts are caused by either atoms with high thermal motion connected to some that are strongly held in place or viceversa

```
PLAT242_ALERT_2_C Low    'MainMol' Ueq as Compared to Neighbors of        O36 Check
```

Author Response: all these alerts are caused by either atoms with high thermal motion connected to some that are strongly held in place or viceversa

```
PLAT242_ALERT_2_C Low    'MainMol' Ueq as Compared to Neighbors of         C4 Check
```

Author Response: all these alerts are caused by either atoms with high thermal motion connected to some that are strongly held in place or viceversa

```
PLAT242_ALERT_2_C Low    'MainMol' Ueq as Compared to Neighbors of        C12 Check
```

Author Response: all these alerts are caused by either atoms with high thermal motion connected to some that are strongly held in place or viceversa

```
PLAT242_ALERT_2_C Low    'MainMol' Ueq as Compared to Neighbors of        C20 Check
```

Author Response: all these alerts are caused by either atoms with high thermal motion connected to some that are strongly held in place or viceversa

```
PLAT242_ALERT_2_C Low    'MainMol' Ueq as Compared to Neighbors of        C24 Check
```

Author Response: all these alerts are caused by either atoms with high thermal motion connected to some that are strongly held in place or viceversa

```
PLAT242_ALERT_2_C Low    'MainMol' Ueq as Compared to Neighbors of        C40 Check
```

Author Response: all these alerts are caused by either atoms with high thermal motion connected to some that are strongly held in place or viceversa

```
PLAT242_ALERT_2_C Low    'MainMol' Ueq as Compared to Neighbors of       C73 Check
```

Author Response: all these alerts are caused by either atoms with high thermal motion connected to some that are strongly held in place or viceversa

```
PLAT243_ALERT_4_C High   'Solvent' Ueq as Compared to Neighbors of       C21 Check
PLAT243_ALERT_4_C High   'Solvent' Ueq as Compared to Neighbors of       C28 Check
PLAT243_ALERT_4_C High   'Solvent' Ueq as Compared to Neighbors of       C48 Check
PLAT250_ALERT_2_C Large U3/U1 Ratio for <U(i,j)> Tensor(Resd    4)       3.1 Note
PLAT260_ALERT_2_C Large Average Ueq of Residue Including      Zr1      0.135 Check
PLAT260_ALERT_2_C Large Average Ueq of Residue Including       O9      0.141 Check
PLAT260_ALERT_2_C Large Average Ueq of Residue Including       O5      0.176 Check
PLAT260_ALERT_2_C Large Average Ueq of Residue Including      O33      0.199 Check
PLAT329_ALERT_4_C Carbon Atom Hybridisation Unclear for .........       C21 Check
PLAT360_ALERT_2_C Short  C(sp3)-C(sp3) Bond  C47      - C52      .       1.43 Ang.
PLAT412_ALERT_2_C Short Intra XH3 .. XHn     H44      ..H74A     .       1.81 Ang.
                                                      x,y,z  =     1_555 Check
```

Author Response: H atoms were geomtrically placed. In a structure with a lot of disorder alert like this one are not unexpected

```
PLAT413_ALERT_2_C Short Inter XH3 .. XHn     H51A     ..H82B     .       2.11 Ang.
                                                      x,y,z  =     1_555 Check
```

Author Response: H atoms were geomtrically placed. In a structure with a lot of disorder alert like this one are not unexpected

```
PLAT906_ALERT_3_C Large K Value in the Analysis of Variance ......      7.850 Check
PLAT906_ALERT_3_C Large K Value in the Analysis of Variance ......      2.309 Check
PLAT911_ALERT_3_C Missing FCF Refl Between Thmin & STh/L=    0.600         92 Report
              -5  1  0,   1  2  0,  -4  8  0,   2-17  1,   2 -9  1,   1 -8  1,
               0 -6  1,   5 -1  1,   6  0  1,  -4  1  1,  -6  2  1, -16  5  1,
              -1 15  1,   2 -8  2,   1 -7  2,   0 -5  2,  -1 -4  2,  -1 -3  2,
              -1 -1  2,  -1  0  2,  -4  1  2,  -1  2  2,  -1 15  2,   2 -7  3,
               1  1  3,  -3  2  3,  -1 15  3,   2 -7  4,   5 -2  4,  -3  2  4,
                  (   62 More Missing: see the .ckf listing file)
PLAT913_ALERT_3_C Missing # of Very Strong Reflections in FCF ....          6 Note
              -1  1  0,   0 -1  1,  -1  0  1,   1  0  1,   0  1  1,   0  0  2,
PLAT918_ALERT_3_C Reflection(s) with I(obs) much Smaller I(calc) .          4 Check
               3  2  0,   3  3  0,   5  4  2,   3  6  4,
PLAT971_ALERT_2_C Check Calcd Resid. Dens.  1.07Ang From Zr6           1.82 eA-3
PLAT971_ALERT_2_C Check Calcd Resid. Dens.  1.19Ang From Zr1           1.69 eA-3
PLAT971_ALERT_2_C Check Calcd Resid. Dens.  1.17Ang From Zr5           1.68 eA-3
PLAT971_ALERT_2_C Check Calcd Resid. Dens.  1.04Ang From Zr2           1.64 eA-3
PLAT971_ALERT_2_C Check Calcd Resid. Dens.  0.98Ang From Zr3           1.62 eA-3
PLAT971_ALERT_2_C Check Calcd Resid. Dens.  1.13Ang From Zr3           1.61 eA-3
```

```
PLAT971_ALERT_2_C Check Calcd Resid. Dens.  1.03Ang From Zr3           1.56 eA-3
PLAT971_ALERT_2_C Check Calcd Resid. Dens.  1.09Ang From Zr4           1.51 eA-3
PLAT971_ALERT_2_C Check Calcd Resid. Dens.  1.10Ang From Zr4           1.51 eA-3
PLAT973_ALERT_2_C Check Calcd Positive Resid. Density on      Zr6      1.19 eA-3
PLAT977_ALERT_2_C Check Negative Difference Density on H3A      .     -0.40 eA-3
PLAT977_ALERT_2_C Check Negative Difference Density on H3C      .     -0.51 eA-3
PLAT977_ALERT_2_C Check Negative Difference Density on H9A      .     -0.41 eA-3
PLAT977_ALERT_2_C Check Negative Difference Density on H11      .     -0.36 eA-3
PLAT977_ALERT_2_C Check Negative Difference Density on H29C     .     -0.32 eA-3
PLAT977_ALERT_2_C Check Negative Difference Density on H49C     .     -0.42 eA-3
PLAT977_ALERT_2_C Check Negative Difference Density on H51C     .     -0.31 eA-3
PLAT977_ALERT_2_C Check Negative Difference Density on H85A     .     -0.37 eA-3
PLAT977_ALERT_2_C Check Negative Difference Density on H90A     .     -0.48 eA-3
PLAT977_ALERT_2_C Check Negative Difference Density on H90B     .     -0.41 eA-3
PLAT977_ALERT_2_C Check Negative Difference Density on H92A     .     -0.36 eA-3
```

Alert level G

```
ABSMU01_ALERT_1_G  Calculation of _exptl_absorpt_correction_mu
                not performed for this radiation type.
PLAT002_ALERT_2_G Number of Distance or Angle Restraints on AtSite        79 Note
PLAT003_ALERT_2_G Number of Uiso or U(i,j)  Restrained non-H-Atoms        41 Report
PLAT007_ALERT_5_G Number of Unrefined Donor-H Atoms .............          9 Report
              H4      H8      H11     H26     H34A    H34B    H17     H5
              H37
PLAT068_ALERT_1_G Reported F000 Differs from Calcd (or Missing)...     Please Check
PLAT083_ALERT_2_G SHELXL Second Parameter in WGHT  Unusually Large      25.00 Why ?
PLAT093_ALERT_1_G No s.u.'s on H-positions, Refinement Reported as      mixed Check
PLAT154_ALERT_1_G The s.u.'s on the Cell Angles are Equal ..(Note)      0.002 Degree
PLAT171_ALERT_4_G The CIF-Embedded .res File Contains EADP Records        17 Report
PLAT172_ALERT_4_G The CIF-Embedded .res File Contains DFIX Records        43 Report
PLAT173_ALERT_4_G The CIF-Embedded .res File Contains DANG Records        24 Report
PLAT176_ALERT_4_G The CIF-Embedded .res File Contains SADI Records        11 Report
PLAT178_ALERT_4_G The CIF-Embedded .res File Contains SIMU Records         3 Report
PLAT186_ALERT_4_G The CIF-Embedded .res File Contains ISOR Records         3 Report
PLAT187_ALERT_4_G The CIF-Embedded .res File Contains RIGU Records         3 Report
PLAT299_ALERT_4_G Atom Site Occupancy Constrained at ............        0.5 Check
              C2      C5      C15     C23     C36     C37     C43     C46
              C49     C57     C61     C63     C70     C71     C77     C79
              C87     C88     C93     C94     H2      H5A     H5B     H5C
              H15A    H15B    H15C    H36     H37A    H37B    H37C    H38
              H38A    H40     H40A    H43A    H43B    H49A    H49B    H49C
              H50     H57A    H57B    H61A    H61B    H70A    H70B    H70C
              H77A    H77B    H77C    H79A    H79B    H79C    H87A    H87B
              H87C    H88A    H88B    H88C    H93A    H93B    H93C    H94A
              H94B    H94C    C59     C64     C81     H21A    H21B    H59A
              H59B    H59C    H64     H64A    H81A    H81B    H81C
PLAT300_ALERT_4_G Atom Site Occupancy of C27        Constrained at       0.65 Check
PLAT300_ALERT_4_G Atom Site Occupancy of C53        Constrained at       0.65 Check
PLAT300_ALERT_4_G Atom Site Occupancy of C67        Constrained at       0.65 Check
PLAT300_ALERT_4_G Atom Site Occupancy of C33        Constrained at       0.35 Check
PLAT300_ALERT_4_G Atom Site Occupancy of C41        Constrained at       0.35 Check
PLAT300_ALERT_4_G Atom Site Occupancy of C65        Constrained at       0.35 Check
PLAT300_ALERT_4_G Atom Site Occupancy of H67        Constrained at       0.65 Check
PLAT300_ALERT_4_G Atom Site Occupancy of H41        Constrained at       0.35 Check
PLAT300_ALERT_4_G Atom Site Occupancy of O33        Constrained at        0.8 Check
PLAT300_ALERT_4_G Atom Site Occupancy of O37        Constrained at        0.8 Check
```

```
PLAT300_ALERT_4_G Atom Site Occupancy of C10       Constrained at        0.8 Check
PLAT300_ALERT_4_G Atom Site Occupancy of H37       Constrained at        0.8 Check
PLAT301_ALERT_3_G Main Residue  Disorder ..............(Resd    1)        13% Note
PLAT302_ALERT_4_G Anion/Solvent/Minor-Residue Disorder (Resd    2)        23% Note
PLAT302_ALERT_4_G Anion/Solvent/Minor-Residue Disorder (Resd    4)        38% Note
PLAT304_ALERT_4_G Non-Integer Number of Atoms in ..... (Resd    2)      15.50 Check
PLAT304_ALERT_4_G Non-Integer Number of Atoms in ..... (Resd    4)      16.20 Check
PLAT309_ALERT_2_G Single Bonded Oxygen (C-O > 1.3 Ang) ..........         O39 Check
PLAT315_ALERT_2_G Singly Bonded Carbon Detected (H-atoms Missing).        C23 Check
PLAT315_ALERT_2_G Singly Bonded Carbon Detected (H-atoms Missing).        C27 Check
PLAT315_ALERT_2_G Singly Bonded Carbon Detected (H-atoms Missing).        C46 Check
PLAT315_ALERT_2_G Singly Bonded Carbon Detected (H-atoms Missing).        C53 Check
PLAT315_ALERT_2_G Singly Bonded Carbon Detected (H-atoms Missing).        C63 Check
PLAT315_ALERT_2_G Singly Bonded Carbon Detected (H-atoms Missing).        C71 Check
PLAT315_ALERT_2_G Singly Bonded Carbon Detected (H-atoms Missing).        C33 Check
PLAT315_ALERT_2_G Singly Bonded Carbon Detected (H-atoms Missing).        C65 Check
PLAT343_ALERT_2_G Unusual         Angle Range in Main Residue for         C44 Check
PLAT343_ALERT_2_G Unusual sp3     Angle Range in Main Residue for         C52 Check
PLAT344_ALERT_2_G Unusual sp3      Angle Range in Solvent/Ion for         C35 Check
PLAT367_ALERT_2_G Long?  C(sp?)-C(sp?) Bond  C44      - C78      .       1.68 Ang.
PLAT412_ALERT_2_G Short Intra XH3 .. XHn     H29C     ..H37A     .       1.64 Ang.
                                                      x,y,z  =      1_555 Check
```

Author Response: H atoms were geomtrically placed. In a structure with a lot of disorder alert like this one are not unexpected

```
PLAT412_ALERT_2_G Short Intra XH3 .. XHn     H29C     ..H40      .       2.07 Ang.
                                                      x,y,z  =      1_555 Check
```

Author Response: H atoms were geomtrically placed. In a structure with a lot of disorder alert like this one are not unexpected

```
PLAT412_ALERT_2_G Short Intra XH3 .. XHn     H29C     ..H49C     .       1.94 Ang.
                                                      x,y,z  =      1_555 Check
```

Author Response: H atoms were geomtrically placed. In a structure with a lot of disorder alert like this one are not unexpected

```
PLAT412_ALERT_2_G Short Intra XH3 .. XHn     H37B     ..H91B     .       2.11 Ang.
                                                      x,y,z  =      1_555 Check
```

Author Response: H atoms were geomtrically placed. In a structure with a lot of disorder alert like this one are not unexpected

```
PLAT412_ALERT_2_G Short Intra XH3 .. XHn     H88C     ..H89A     .       2.10 Ang.
                                                      x,y,z  =      1_555 Check
```

Author Response: H atoms were geomtrically placed. In a structure with a lot of disorder alert like this one are not unexpected

```
PLAT412_ALERT_2_G Short Intra XH3 .. XHn     H59C     ..H82A     .       1.91 Ang.
                                                  x,y,z  =      1_555 Check
```

Author Response: H atoms were geomtrically placed. In a structure with a lot of disorder alert like this one are not unexpected

```
PLAT413_ALERT_2_G Short Inter XH3 .. XHn     H5A      ..H13B     .       2.08 Ang.
                                              -x,-y,1-z  =      2_556 Check
```

Author Response: H atoms were geomtrically placed. In a structure with a lot of disorder alert like this one are not unexpected

```
PLAT413_ALERT_2_G Short Inter XH3 .. XHn     H37C     ..H85A     .       2.14 Ang.
                                               x,-1+y,z  =      1_545 Check
```

Author Response: H atoms were geomtrically placed. In a structure with a lot of disorder alert like this one are not unexpected

```
PLAT413_ALERT_2_G Short Inter XH3 .. XHn     H43A     ..H92C     .       1.73 Ang.
                                             1-x,1-y,2-z  =      2_667 Check
```

Author Response: H atoms were geomtrically placed. In a structure with a lot of disorder alert like this one are not unexpected

```
PLAT413_ALERT_2_G Short Inter XH3 .. XHn     H21B     ..H85B     .       2.09 Ang.
                                             -1+x,-1+y,z  =      1_445 Check
```

Author Response: H atoms were geomtrically placed. In a structure with a lot of disorder alert like this one are not unexpected

```
PLAT721_ALERT_1_G Bond    Calc     0.95000, Rep     0.96050 Dev...       0.01 Ang.
             C79      -H79A           1_555   1_555 ........     #  267 Check
PLAT721_ALERT_1_G Bond    Calc     0.97000, Rep     0.95950 Dev...       0.01 Ang.
             C79      -H79C           1_555   1_555 ........     #  269 Check
PLAT722_ALERT_1_G Angle   Calc      109.00, Rep      110.20 Dev...       1.20 Degree
             C50     -C77     -H77C       1_555  1_555  1_555     #  812 Check
PLAT722_ALERT_1_G Angle   Calc      110.00, Rep      111.20 Dev...       1.20 Degree
             C57     -C87     -H87B       1_555  1_555  1_555     #  859 Check
PLAT722_ALERT_1_G Angle   Calc      111.00, Rep      109.40 Dev...       1.60 Degree
             C43     -C88     -H88B       1_555  1_555  1_555     #  865 Check
PLAT779_ALERT_4_G Suspect or Irrelevant (Bond) Angle(s) in CIF ...       4.60 Deg.
             C93  -C15  -H15A    1_555   1_555   1_555 ......     #  752 Check
PLAT779_ALERT_4_G Suspect or Irrelevant (Bond) Angle(s) in CIF ...       8.00 Deg.
             C15  -C93  -H15A    1_555   1_555   1_555 ......     #  894 Check
PLAT793_ALERT_4_G Model has Chirality at C17         (Centro SpGr)          S Verify
PLAT793_ALERT_4_G Model has Chirality at C24         (Centro SpGr)          S Verify
PLAT793_ALERT_4_G Model has Chirality at C30         (Centro SpGr)          S Verify
PLAT793_ALERT_4_G Model has Chirality at C34         (Centro SpGr)          R Verify
PLAT793_ALERT_4_G Model has Chirality at C42         (Centro SpGr)          R Verify
```

```
PLAT793_ALERT_4_G Model has Chirality at C52         (Centro SpGr)          R Verify
PLAT793_ALERT_4_G Model has Chirality at C62         (Centro SpGr)          R Verify
PLAT793_ALERT_4_G Model has Chirality at C73         (Centro SpGr)          R Verify
PLAT794_ALERT_5_G Tentative Bond Valency for Zr1       (IV)      .       4.24 Info
PLAT794_ALERT_5_G Tentative Bond Valency for Zr2       (IV)      .       4.25 Info
PLAT794_ALERT_5_G Tentative Bond Valency for Zr3       (IV)      .       3.99 Info
PLAT794_ALERT_5_G Tentative Bond Valency for Zr4       (IV)      .       4.10 Info
PLAT794_ALERT_5_G Tentative Bond Valency for Zr5       (IV)      .       4.06 Info
PLAT794_ALERT_5_G Tentative Bond Valency for Zr6       (IV)      .       4.20 Info
PLAT860_ALERT_3_G Number of Least-Squares Restraints ............         285 Note
PLAT883_ALERT_1_G Absent Datum for _atom_sites_solution_primary ..     Please Do !
PLAT912_ALERT_4_G Missing # of FCF Reflections Above STh/L=  0.600        311 Note
PLAT933_ALERT_2_G Number of HKL-OMIT Records in Embedded .res File         10 Note
               -6  2  1,  -4  8  0,  -3  2  3,  -3  2  4,  -1 -2  5,  -1 -1  2,
               -1  2  2,   1  1  3,   1  2  0,   5 -2  4,
PLAT941_ALERT_3_G Average HKL Measurement Multiplicity ..........         4.0 Low
PLAT969_ALERT_5_G The 'Henn et al.' R-Factor-gap value ..........       7.278 Note
              Predicted wR2: Based on SigI**2  4.27 or SHELX Weight 27.76
PLAT978_ALERT_2_G Number C-C Bonds with Positive Residual Density.          0 Info
```

```
   3 ALERT level A = Most likely a serious problem - resolve or explain
   9 ALERT level B = A potentially serious problem, consider carefully
  70 ALERT level C = Check. Ensure it is not caused by an omission or oversight
  84 ALERT level G = General information/check it is not something unexpected

  11 ALERT type 1 CIF construction/syntax error, inconsistent or missing data
  88 ALERT type 2 Indicator that the structure model may be wrong or deficient
  12 ALERT type 3 Indicator that the structure quality may be low
  47 ALERT type 4 Improvement, methodology, query or suggestion
   8 ALERT type 5 Informative message, check
```

It is advisable to attempt to resolve as many as possible of the alerts in all categories. Often the minor alerts point to easily fixed oversights, errors and omissions in your CIF or refinement strategy, so attention to these fine details can be worthwhile. It is up to the individual to critically assess their own results and, if necessary, seek expert advice.

PLATON version of 26/09/2025; check.def file version of 20/09/2025

Datablock hw07_150k_new - ellipsoid plot

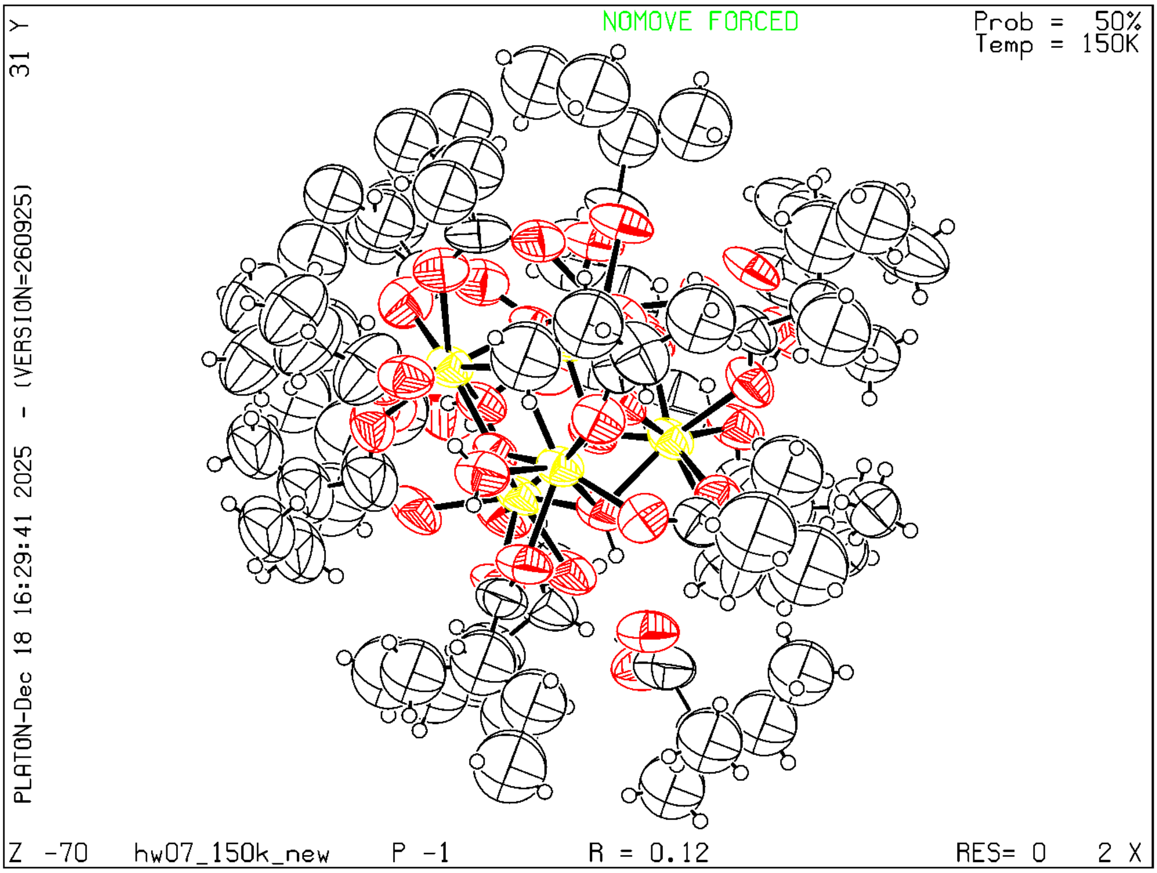

Supplement: Supplementary file 2 — Supporting File 2: anie71298–sup–0002–Data.zip. [file ANIE-65-e25769-s002.zip › CCDC_2453142/hw07_150k_new_cifreport.pdf]
