# Supplementary material for: Mechanism‐Guided Precision Hydrolysis of Early Transition Metals to Access (Mixed‐Metal) Oxo Clusters
Source: Angew Chem Int Ed Engl. 2026 Feb 24;65(15):e25769. doi: 10.1002/anie.202525769 (PMC13053926; doi:10.1002/anie.202525769)
Supplement: Supplementary file 2 — Supporting File 2: anie71298–sup–0002–Data.zip. [file ANIE-65-e25769-s002.zip › CCDC_2453143/mjp169_150k_new.rtf]

;;;;;;;fLine0fBehindDocument1shapeType1pibmjp169_150k_new

Submitted by: 	Muhammed Jibin Parammal
		University of Basel
Solved by: 	Alessandro Prescimone
Sample ID: 	MJP169
R1=8.81%


Crystal Data and Experimental


Experimental. Single colourless plate-shaped crystals of mjp169_150k_new were used as supplied. A suitable crystal with dimensions 0.13 × 0.10 × 0.06 mm was selected and mounted on a STOE STADIVARI Cu diffractometer. The crystal was kept at a steady T = 150 K during data collection. The structure was solved with the ShelXT 2018/2 (Sheldrick, 2018) solution program using iterative methods and by using Olex2 1.5 (Dolomanov et al., 2009) as the graphical interface. The model was refined with ShelXL 2019/3 (Sheldrick, 2015) using full matrix least squares minimisation on |F|2.
Crystal Data. C74H136Hf6O39, Mr = 2720.76, triclinic, P-1 (No. 2), a = 14.0863(5) Å, b = 15.6140(6) Å, c = 23.9826(8) Å, a = 94.310(3)°, b = 96.311(3)°, g = 109.356(3)°, V = 4911.4(3) Å3, T = 150 K, Z = 2, Z' = 1, m(Cu Ka) = 11.997, 73934 reflections measured, 18232 unique (Rint = 0.0674) which were used in all calculations. The final wR2 was 0.2521 (all data) and R1 was 0.0881 (I≥2s(I)).
Compound 	mjp169_150k_new 	
 	 	
Formula 	C74H136Hf6O39 	
Dcalc./ g cm-3 	1.840 	
m/mm-1 	11.997 	
Formula Weight 	2720.76 	
Colour 	colourless 	
Shape 	plate-shaped 	
Size/mm 	0.13×0.10×0.06 	
T/K 	150 	
Crystal System 	triclinic 	
Space Group 	P-1 	
a/Å 	14.0863(5) 	
b/Å 	15.6140(6) 	
c/Å 	23.9826(8) 	
a/° 	94.310(3) 	
b/° 	96.311(3) 	
g/° 	109.356(3) 	
V/Å3 	4911.4(3) 	
Z 	2 	
Z' 	1 	
Wavelength/Å 	1.54186 	
Radiation type 	Cu Ka 	
Qmin/° 	4.655 	
Qmax/° 	70.479 	
Index range h 	-16 ≥ h ≥ 17 	
Index range k 	-19 ≥ k ≥ 17 	
Index range l 	-19 ≥ l ≥ 28 	
Measured Refl's. 	73934 	
Indep't Refl's 	18232 	
Refl's I≥2s(I) 	10640 	
Rint 	0.0674 	
Parameters 	703 	
Restraints 	364 	
Largest Peak/eÅ3 	1.665 	
Deepest Hole/eÅ3 	-1.363 	
GooF 	1.052 	
R1 (I≥2s(I) / all) 	0.0881 / 0.1240 	
wR2 (I≥2s(I) / all) 	0.2227 / 0.2521 	

Structure Quality Indicators
Reflections:		
Refinement:		
A colourless plate-shaped crystal with dimensions 0.13 × 0.10 × 0.06 mm was mounted. Data were collected using a STOE STADIVARI Cu diffractometer equipped with an Oxford Cryosystems low-temperature device operating at T = 150 K.
Data were measured using rotation method, w scans with Cu Ka radiation. The diffraction pattern was indexed and the total number of runs and images was based on the strategy calculation from the program X-Area Pilatus3_SV 1.31.170.0 (STOE, 2020) . The maximum resolution achieved was Q = 70.479° (0.82 Å).
The unit cell was refined using X-Area Pilatus3_SV 1.31.170.0 (STOE, 2020) on 40509 reflections, 55% of the observed reflections.
Data reduction, scaling and absorption corrections were performed using X-Area Pilatus3_SV 1.31.170.0 (STOE, 2020). The final completeness is 98.80 % out to 70.479° in Q. A multi-scan absorption correction was performed using STOE. The absorption coefficient m of this material is 11.997 mm-1 at this wavelength (l = 1.54186Å) and the minimum and maximum transmissions are 0.003 and 0.036.
The structure was solved in the space group P-1 (# 2) by ShelXT 2018/2 (Sheldrick, 2018) using iterative methods. It was refined by full matrix least squares minimisation on |F|2 using version 2019/3 of ShelXL 2019/3 (Sheldrick, 2015). All non-hydrogen atoms were refined anisotropically.
Hydrogen atom positions were calculated geometrically and refined using the riding model.
_refine_special_details: crystal was weakly diffracting and with a lot of disorder.One atom on one of the ligands was not found in the diffrerence map.H atoms on this ligand were therefore omitted as some C atoms couldeither be a CH2 or a CH3.several atoms had to be refined isotropicallya lot of SADI, EADP, DFIX and D&nbsp;&Aring; had to used
_exptl_absorpt_process_details: STOE X-Red32, absorption correction by Gaussian integration, analogous toP. Coppens in: F. R. Ahmed (Editor), "Crystallographic Computing", Munksgaard,Copenhagen (1970), 255 - 270. Afterwards scaling of reflection intensities wasperformed within STOE LANA. J. Koziskova, F. Hahn, J. Richter, J. Kozisek, ActaChimica Slovaca, vol. 9, no. 2, 2016, pp. 136 - 140.Finally a spherical absorption correction was done within STOE LANA.
There is a single formula unit in the asymmetric unit, which is represented by the reported sum formula. In other words: Z is 2 and Z' is 1. The moiety formula is C59 H106 Hf6 O33, 3(C5 H10 O2).
Data Plots: Diffraction Data
 	 	
 	 	
Data Plots: Refinement and Data
 	 	
Reflection Statistics

Total reflections (after filtering) 	73934 	Unique reflections 	18232 	
Completeness 	0.97 	Mean I/s 	9.9 	
hklmax collected 	(17, 17, 28) 	hklmin collected 	(-16, -19, -19) 	
hklmax used 	(17, 18, 28) 	hklmin used 	(-17, -19, 0) 	
Lim dmax collected 	100.0 	Lim dmin collected 	0.77 	
dmax used 	9.5 	dmin used 	0.82 	
Friedel pairs 	3492 	Friedel pairs merged 	1 	
Inconsistent equivalents 	276 	Rint 	0.0674 	
Rsigma 	0.0476 	Intensity transformed 	0 	
Omitted reflections 	0 	Omitted by user (OMIT hkl) 	11 	
Multiplicity 	(4570, 4964, 3495, 2875, 2066, 1464, 1002, 687, 367, 146, 71, 18, 6) 	Maximum multiplicity 	14 	
Removed systematic absences 	0 	Filtered off (Shel/OMIT) 	0 	


Table 0: Fractional Atomic Coordinates (×104) and Equivalent Isotropic Displacement Parameters (Å2×103) for mjp169_150k_new. Ueq is defined as 1/3 of the trace of the orthogonalised Uij.

Atom	x	y	z	Ueq	
Hf1	2469.7(6)	1514.9(5)	7536.5(3)	96.1(2)	
Hf4	4919.7(6)	3071.9(6)	7693.2(3)	100.7(3)	
Hf6	1541.0(6)	3309.2(5)	7773.0(3)	97.7(2)	
Hf3	3378.1(6)	3038.2(5)	8750.9(3)	94.9(2)	
Hf2	3056.9(6)	3315.7(6)	6720.3(3)	103.3(3)	
Hf5	3981.5(6)	4885.1(5)	7924.3(3)	98.8(2)	
O1	4181(7)	3895(7)	7389(4)	93(3)	
O4	4695(7)	4067(6)	8444(4)	91(2)	
O6	2036(7)	2519(7)	7198(4)	99(3)	
O8	3540(8)	2211(7)	6985(4)	105(3)	
O10	4805(11)	2819(8)	9120(5)	124(4)	
O12	2337(10)	689(7)	8236(5)	115(3)	
O14	3686(7)	2405(7)	8056(4)	92(2)	
O16	957(9)	3308(8)	8577(6)	124(4)	
O18	6231(9)	4265(10)	7813(5)	115(3)	
O20	1389(11)	4649(11)	7955(5)	134(4)	
O22	323(10)	3317(11)	7080(5)	134(4)	
O24	2876(10)	1691(8)	9026(5)	123(4)	
O26	1866(7)	2195(7)	8167(4)	96(3)	
O28	825(10)	568(8)	7398(5)	127(4)	
O30	5065(9)	1764(10)	7541(5)	121(4)	
O32	2912(14)	5623(9)	8029(6)	155(5)	
O34	4042(9)	4142(7)	9471(4)	122(4)	
O36	4600(10)	5810(8)	8681(5)	126(4)	
O38	-32(9)	2232(9)	7587(6)	131(3)	
O40	2154(10)	3112(8)	9207(4)	112(3)	
O41	5751(9)	2929(9)	8454(5)	118(3)	
O21	2888(7)	3871(6)	8263(4)	93(2)	
O11	2523(8)	4194(7)	7246(4)	102(3)	
O23	3647(10)	4671(9)	6431(5)	128(4)	
O3	4315(11)	3311(10)	6261(5)	129(4)	
O25	5708(11)	5465(9)	7914(5)	131(4)	
O13	1697(11)	566(9)	6725(5)	134(4)	
O27	5501(10)	3138(11)	6897(6)	139(5)	
O7	1774(10)	3374(11)	6104(5)	139(4)	
O29	5578(10)	5654(9)	9380(5)	129(4)	
O15	4215(10)	5696(9)	7220(5)	129(4)	
O31	2286(11)	2229(11)	5963(5)	135(4)	
O2	3488(12)	716(9)	7439(6)	134(4)	
O33	6124(11)	-1082(11)	3856(6)	154(6)	
O17	-1020(13)	786(12)	8066(10)	201(8)	
C1	880(30)	290(30)	6908(18)	219(6)	
O9	329(12)	1015(11)	8651(5)	150(5)	
C4	2580(30)	860(20)	8742(15)	245(9)	
C6	-350(20)	2620(30)	7239(13)	181(4)	
C8	5620(30)	2840(20)	8930(14)	200(5)	
C10	6630(40)	-550(40)	4220(20)	275(9)	
C12	1289(18)	3228(14)	9068(9)	125(6)	
C14	4034(17)	5473(15)	6684(11)	138(6)	
O37	7630(20)	-156(15)	4247(7)	225(10)	
C16	2010(30)	5450(30)	8060(20)	247(6)	
C18	5280(16)	6126(13)	9063(9)	121(6)	
C20	6379(15)	5090(20)	7849(9)	133(7)	
C22	4420(18)	951(17)	7453(10)	134(7)	
C24	6339(17)	2588(16)	9396(10)	200(5)	
C26	1740(30)	2630(30)	5801(12)	216(7)	
C28	-540(30)	480(30)	8467(16)	211(14)	
C30	2540(20)	94(18)	9088(11)	245(9)	
C32	5162(18)	3259(19)	6399(10)	147(8)	
C34	6300(30)	-190(20)	4777(18)	275(9)	
C38	-10(30)	-480(20)	6519(15)	219(6)	
C40	4760(30)	100(30)	7207(13)	300	
C42	-1439(19)	2325(16)	6903(10)	181(4)	
C44	5770(20)	7157(18)	9297(9)	200	
C46	8090(40)	5470(30)	8642(16)	300	
C48	880(30)	5130(20)	6301(14)	300	
C50	670(20)	3340(20)	9562(13)	200	
C52	1460(20)	6133(18)	8174(14)	247(6)	
C54	320(30)	6100(30)	5671(17)	300	
C56	6790(30)	-390(20)	5303(16)	275(9)	
C60	6740(20)	1885(18)	9160(11)	200(5)	
C62	960(20)	2340(20)	5266(10)	216(7)	
C64	-1090(30)	-128(18)	8843(13)	247(16)	
C68	260(30)	1410(20)	5278(10)	216(7)	
C70	-1010(20)	-330(20)	6559(14)	219(6)	
C72	-1980(20)	2800(20)	7256(10)	181(4)	
C74	6910(20)	7460(20)	9446(12)	200	
C78	5230(20)	7730(20)	8997(10)	200	
C80	-1658.95	1346.22	6629.71	181(4)	
C5	0(30)	-1320(20)	6795(14)	219(6)	
C41	5410(30)	6660(20)	6353(19)	280	
C21	-1540(20)	-920(20)	8391(11)	200	
C43	270(50)	4110(30)	9500(30)	200	
C11	1660(20)	6390(20)	8777(12)	247(6)	
C45	6680(30)	830(20)	4761(14)	275(9)	
C47	1630(20)	7288(19)	7968(14)	247(6)	
C3	6460(30)	4694(19)	5996(17)	300	
C49	4370(30)	-640(30)	7571(18)	300	
C25	3600(20)	80(20)	9268(13)	245(9)	
C51	1990(40)	-800(20)	8730(20)	245(9)	
C13	1470(20)	2440(20)	4763(9)	216(7)	
C27	4280(30)	6300(30)	6317(18)	280	
C55	6020(30)	2170(20)	5746(17)	300	
C7	7156(19)	3463(16)	9652(12)	200(5)	
C57	500(40)	2440(30)	9782(18)	200	
C29	1200(30)	6120(30)	5394(16)	300	
C59	-1280(40)	-450(30)	9398(13)	200	
C63	7980(30)	6090(20)	8226(15)	300	
C2	7370(20)	5650(20)	7672(13)	300	
C67	4100(30)	7060(20)	6674(19)	280	
C69	630(30)	5950(20)	6263(14)	300	
C71	7510(40)	6320(20)	7247(13)	300	
C9	6710(20)	3810(20)	6010(20)	300	
C73	5580(20)	3080(20)	5830(20)	300	
O39	1693(14)	5335(11)	6688(7)	169(6)	
O5	720(19)	4434(17)	5903(10)	234(9)	
C37	4960(30)	-220(30)	8175(14)	300	
C75	5870(30)	410(30)	7142(15)	300	
C77	-270(30)	3490(40)	9310(20)	200	
C79	60(50)	2550(30)	9651(14)	200	
C81	-1970(30)	130(30)	8960(20)	200	
C76	2060(40)	180(40)	9600(20)	245(9)	
C82	-600(20)	-1230(20)	8425(12)	200	
C83	-120(20)	2420(20)	10249(11)	200	
C84	6810(20)	4050(20)	9984(12)	200(5)	
C85	5560(20)	7750(20)	8423(9)	200	
C128	2650(20)	7395(19)	7794(13)	247(6)	
C86	-1834(19)	3117(19)	7853(10)	181(4)	
C87	2190(20)	3340(20)	4779(10)	216(7)	
C88	3580(30)	-420(20)	9780(13)	245(9)	
C17	6420(30)	-1430(20)	5272(15)	275(9)	
C15	950(30)	-1560(20)	6772(14)	219(6)	
C19	-420(30)	5620(30)	6415(18)	310(20)	


Table 0: Anisotropic Displacement Parameters (×104) for mjp169_150k_new. The anisotropic displacement factor exponent takes the form: -2p2[h2a*2 × U11+ ... +2hka* × b* × U12]

Atom	U11	U22	U33	U23	U13	U12	
Hf1	96.0(5)	93.1(5)	90.1(4)	-18.7(4)	0.9(4)	30.4(4)	
Hf4	85.7(5)	117.6(6)	96.0(5)	-9.7(4)	6.0(4)	38.1(4)	
Hf6	85.2(4)	102.0(5)	100.1(5)	-16.9(4)	8.3(4)	32.3(4)	
Hf3	99.0(5)	91.6(5)	79.2(4)	-10.3(3)	4.3(3)	19.9(4)	
Hf2	100.7(5)	126.7(6)	80.0(4)	-2.1(4)	4.3(4)	41.6(5)	
Hf5	97.0(5)	91.4(5)	99.1(5)	-2.9(4)	5.9(4)	26.4(4)	
O1	80(6)	114(7)	84(5)	10(5)	14(4)	30(5)	
O4	72(5)	92(6)	91(6)	-10(5)	5(4)	9(4)	
O6	88(6)	110(7)	92(6)	-16(5)	0(5)	37(5)	
O8	107(7)	115(7)	94(6)	-14(5)	21(5)	44(6)	
O10	136(10)	101(7)	106(7)	-9(6)	-34(7)	24(7)	
O12	135(9)	100(7)	98(7)	2(6)	2(6)	32(6)	
O14	80(5)	101(6)	91(6)	-3(5)	4(4)	33(5)	
O16	98(8)	124(9)	134(10)	-29(7)	35(7)	21(6)	
O18	90(7)	126(9)	121(8)	-5(7)	11(6)	32(7)	
O20	133(8)	180(9)	122(8)	1(8)	11(7)	103(7)	
O22	119(8)	170(10)	120(8)	-14(7)	-17(6)	73(7)	
O24	149(10)	98(7)	94(7)	14(6)	1(6)	11(7)	
O26	89(6)	95(6)	92(6)	-8(5)	14(5)	19(5)	
O28	130(9)	105(8)	112(8)	-33(6)	9(7)	9(7)	
O30	104(8)	141(10)	128(9)	-9(8)	9(7)	64(8)	
O32	202(10)	99(8)	158(11)	-32(7)	-4(10)	64(8)	
O34	136(9)	103(7)	86(6)	-30(5)	-3(6)	3(6)	
O36	134(10)	96(7)	117(8)	-16(6)	-6(7)	14(7)	
O38	103(8)	120(8)	152(9)	-36(6)	31(6)	22(6)	
O40	134(10)	104(7)	82(6)	-12(5)	12(6)	24(7)	
O41	95(7)	133(9)	116(8)	-18(7)	-3(6)	38(6)	
O21	83(6)	96(6)	91(6)	-16(5)	17(4)	23(5)	
O11	108(7)	101(7)	99(6)	8(5)	9(5)	42(6)	
O23	134(10)	129(9)	113(8)	15(7)	15(7)	35(8)	
O3	129(10)	168(12)	101(8)	1(7)	30(7)	66(9)	
O25	123(10)	126(9)	115(9)	10(7)	4(7)	10(8)	
O13	158(11)	126(9)	93(7)	-59(6)	-31(7)	48(8)	
O27	111(9)	197(14)	110(9)	-7(9)	33(7)	51(9)	
O7	115(9)	195(13)	94(7)	4(8)	-14(6)	49(9)	
O29	133(10)	122(9)	93(7)	-15(7)	-1(7)	5(8)	
O15	135(10)	128(9)	114(9)	23(7)	6(7)	35(8)	
O31	135(10)	179(12)	76(6)	-11(7)	0(6)	46(9)	
O2	143(12)	120(9)	130(9)	-23(7)	3(8)	49(8)	
O33	130(10)	197(14)	131(10)	-57(10)	0(8)	74(10)	
O17	128(12)	145(13)	280(20)	15(14)	46(14)	-26(10)	
C1	222(9)	209(9)	212(9)	5(7)	27(8)	62(7)	
O9	136(11)	167(12)	114(9)	-9(8)	33(8)	9(9)	
C4	300(20)	178(13)	237(19)	68(12)	-27(16)	61(15)	
C6	151(6)	204(7)	190(7)	-7(6)	26(5)	70(5)	
C8	183(11)	207(12)	212(12)	-16(9)	-53(9)	103(9)	
C12	133(16)	135(15)	104(13)	-13(11)	29(12)	45(13)	
O37	290(30)	230(20)	149(13)	-94(13)	10(15)	105(19)	
C16	220(10)	221(10)	285(13)	-64(11)	13(12)	83(9)	
C18	136(15)	98(12)	109(13)	-18(10)	-12(11)	30(11)	
C20	93(13)	190(20)	115(14)	34(15)	36(10)	40(15)	
C22	104(14)	134(16)	167(18)	-32(14)	-7(13)	61(13)	
C24	183(11)	207(12)	212(12)	-16(9)	-53(9)	103(9)	
C26	250(20)	263(19)	105(7)	9(11)	-25(9)	64(14)	
C30	300(20)	178(13)	237(19)	68(12)	-27(16)	61(15)	
C32	111(15)	200(20)	111(15)	-13(14)	37(12)	37(15)	
C38	222(9)	209(9)	212(9)	5(7)	27(8)	62(7)	
C42	151(6)	204(7)	190(7)	-7(6)	26(5)	70(5)	
C52	220(10)	221(10)	285(13)	-64(11)	13(12)	83(9)	
C60	183(11)	207(12)	212(12)	-16(9)	-53(9)	103(9)	
C62	250(20)	263(19)	105(7)	9(11)	-25(9)	64(14)	
C68	250(20)	263(19)	105(7)	9(11)	-25(9)	64(14)	
C70	222(9)	209(9)	212(9)	5(7)	27(8)	62(7)	
C72	151(6)	204(7)	190(7)	-7(6)	26(5)	70(5)	
C80	151(6)	204(7)	190(7)	-7(6)	26(5)	70(5)	
C5	222(9)	209(9)	212(9)	5(7)	27(8)	62(7)	
C11	220(10)	221(10)	285(13)	-64(11)	13(12)	83(9)	
C47	220(10)	221(10)	285(13)	-64(11)	13(12)	83(9)	
C25	300(20)	178(13)	237(19)	68(12)	-27(16)	61(15)	
C51	300(20)	178(13)	237(19)	68(12)	-27(16)	61(15)	
C13	250(20)	263(19)	105(7)	9(11)	-25(9)	64(14)	
C7	183(11)	207(12)	212(12)	-16(9)	-53(9)	103(9)	
O39	179(15)	154(12)	170(13)	13(10)	-5(11)	62(11)	
C76	300(20)	178(13)	237(19)	68(12)	-27(16)	61(15)	
C84	183(11)	207(12)	212(12)	-16(9)	-53(9)	103(9)	
C128	220(10)	221(10)	285(13)	-64(11)	13(12)	83(9)	
C86	151(6)	204(7)	190(7)	-7(6)	26(5)	70(5)	
C87	250(20)	263(19)	105(7)	9(11)	-25(9)	64(14)	
C88	300(20)	178(13)	237(19)	68(12)	-27(16)	61(15)	
C15	222(9)	209(9)	212(9)	5(7)	27(8)	62(7)	


Table 0: Bond Lengths in Å for mjp169_150k_new.


Atom	Atom	Length/Å	
Hf1	Hf4	3.4608(11)	
Hf1	Hf3	3.4495(9)	
Hf1	Hf2	3.4732(12)	
Hf1	O6	2.051(10)	
Hf1	O8	2.185(11)	
Hf1	O12	2.177(11)	
Hf1	O14	2.027(9)	
Hf1	O26	2.186(10)	
Hf1	O28	2.269(12)	
Hf1	O13	2.281(9)	
Hf1	O2	2.210(14)	
Hf1	C1	2.63(4)	
Hf4	Hf3	3.5083(11)	
Hf4	Hf2	3.4530(10)	
Hf4	Hf5	3.5319(11)	
Hf4	O1	2.031(10)	
Hf4	O4	2.405(9)	
Hf4	O8	2.381(10)	
Hf4	O14	2.032(10)	
Hf4	O18	2.118(13)	
Hf4	O30	2.128(13)	
Hf4	O41	2.125(12)	
Hf4	O27	2.154(13)	
Hf6	Hf3	3.4520(11)	
Hf6	Hf2	3.4796(11)	
Hf6	Hf5	3.4624(11)	
Hf6	O6	2.106(9)	
Hf6	O16	2.178(13)	
Hf6	O20	2.190(14)	
Hf6	O22	2.258(11)	
Hf6	O26	2.193(10)	
Hf6	O38	2.266(12)	
Hf6	O21	2.001(9)	
Hf6	O11	2.191(11)	
Hf6	C6	2.65(3)	
Hf3	Hf5	3.5458(11)	
Hf3	O4	2.250(9)	
Hf3	O10	2.247(13)	
Hf3	O14	2.035(9)	
Hf3	O24	2.164(11)	
Hf3	O26	2.332(9)	
Hf3	O34	2.225(9)	
Hf3	O40	2.170(13)	
Hf3	O21	2.045(10)	
Hf2	Hf5	3.4688(11)	
Hf2	O1	2.042(9)	
Hf2	O6	2.078(11)	
Hf2	O8	2.165(11)	
Hf2	O11	2.162(10)	
Hf2	O23	2.199(13)	
Hf2	O3	2.189(13)	
Hf2	O7	2.236(11)	
Hf2	O31	2.288(12)	
Hf2	C26	2.62(3)	
Hf5	O1	2.046(10)	
Hf5	O4	2.238(10)	
Hf5	O32	2.202(16)	
Hf5	O36	2.136(11)	
Hf5	O21	2.096(10)	
Hf5	O11	2.359(10)	
Hf5	O25	2.301(15)	
Hf5	O15	2.175(12)	
O10	C8	1.27(4)	
O12	C4	1.21(3)	
O16	C12	1.25(2)	
O18	C20	1.23(3)	
O20	C16	1.26(4)	
O22	C6	1.30(4)	
O24	C4	1.33(4)	
O28	C1	1.24(4)	
O30	C22	1.28(2)	
O32	C16	1.22(4)	
O36	C18	1.19(2)	
O38	C6	1.20(3)	
O40	C12	1.30(2)	
O41	C8	1.19(4)	
O23	C14	1.26(2)	
O3	C32	1.23(3)	
O25	C20	1.28(3)	
O13	C1	1.23(4)	
O27	C32	1.29(3)	
O7	C26	1.31(4)	
O29	C18	1.23(2)	
O15	C14	1.28(2)	
O31	C26	1.19(4)	
O2	C22	1.24(2)	
O33	C10	1.15(6)	
O17	C28	1.32(4)	
C1	C38	1.57(5)	
O9	C28	1.24(4)	
C4	C30	1.501(18)	
C6	C42	1.55(3)	
C8	C24	1.59(3)	
C10	O37	1.33(5)	
C10	C34	1.58(4)	
C12	C50	1.58(3)	
C14	C27	1.58(5)	
C16	C52	1.54(3)	
C18	C44	1.56(3)	
C20	C2	1.501(10)	
C22	C40	1.64(4)	
C24	C60	1.498(16)	
C24	C7	1.498(5)	
C26	C62	1.53(4)	
C28	C64	1.446(8)	
C30	C25	1.511(18)	
C30	C51	1.498(9)	
C30	C76	1.497(19)	
C32	C73	1.59(5)	
C34	C56	1.49(2)	
C34	C45	1.50(2)	
C38	C70	1.509(16)	
C38	C5	1.517(18)	
C40	C49	1.497(16)	
C40	C75	1.51(2)	
C42	C72	1.504(10)	
C42	C80	1.53(2)	
C44	C74	1.502(10)	
C44	C78	1.519(10)	
C46	C63	1.48(3)	
C48	C69	1.436(10)	
C48	O39	1.33(2)	
C48	O5	1.340(10)	
C50	C43	1.508(10)	
C50	C57	1.484(19)	
C50	C77	1.496(9)	
C50	C79	1.29(5)	
C52	C11	1.44(2)	
C52	C47	1.85(3)	
C54	C29	1.465(5)	
C54	C69	1.497(7)	
C56	C17	1.51(2)	
C62	C68	1.46(2)	
C62	C13	1.466(16)	
C64	C21	1.494(5)	
C64	C59	1.479(13)	
C64	C81	1.476(17)	
C72	C86	1.45(2)	
C78	C85	1.499(10)	
C5	C15	1.52(2)	
C41	C27	1.49(3)	
C21	C82	1.56(3)	
C47	C128	1.499(5)	
C3	C9	1.54(3)	
C49	C37	1.57(3)	
C25	C88	1.51(2)	
C13	C87	1.43(2)	
C27	C67	1.52(3)	
C55	C73	1.725(12)	
C7	C84	1.41(3)	
C57	C83	1.497(10)	
C63	C2	1.486(8)	
C2	C71	1.498(13)	
C69	C19	1.496(10)	
C9	C73	1.604(8)	
C79	C83	1.502(10)	


Table 0: Bond Angles in ° for mjp169_150k_new.


Atom	Atom	Atom	Angle/°	
Hf4	Hf1	Hf2	59.73(2)	
Hf3	Hf1	Hf4	61.02(2)	
Hf3	Hf1	Hf2	90.26(2)	
O6	Hf1	Hf4	85.7(3)	
O6	Hf1	Hf3	85.9(3)	
O6	Hf1	Hf2	33.0(3)	
O6	Hf1	O8	69.6(4)	
O6	Hf1	O12	143.9(4)	
O6	Hf1	O26	70.1(4)	
O6	Hf1	O28	90.1(5)	
O6	Hf1	O13	88.8(4)	
O6	Hf1	O2	142.1(5)	
O6	Hf1	C1	88.5(9)	
O8	Hf1	Hf4	42.9(3)	
O8	Hf1	Hf3	99.8(3)	
O8	Hf1	Hf2	36.8(3)	
O8	Hf1	O26	125.1(4)	
O8	Hf1	O28	134.7(4)	
O8	Hf1	O13	82.7(5)	
O8	Hf1	O2	73.6(5)	
O8	Hf1	C1	108.6(10)	
O12	Hf1	Hf4	108.8(3)	
O12	Hf1	Hf3	73.9(3)	
O12	Hf1	Hf2	164.0(3)	
O12	Hf1	O8	142.2(4)	
O12	Hf1	O26	75.0(4)	
O12	Hf1	O28	75.1(5)	
O12	Hf1	O13	108.2(5)	
O12	Hf1	O2	73.7(5)	
O12	Hf1	C1	92.5(10)	
O14	Hf1	Hf4	31.5(3)	
O14	Hf1	Hf3	31.9(3)	
O14	Hf1	Hf2	80.9(3)	
O14	Hf1	O6	94.3(4)	
O14	Hf1	O8	74.1(4)	
O14	Hf1	O12	84.2(4)	
O14	Hf1	O26	73.4(4)	
O14	Hf1	O28	149.9(4)	
O14	Hf1	O13	153.8(5)	
O14	Hf1	O2	84.0(4)	
O14	Hf1	C1	176.7(9)	
O26	Hf1	Hf4	98.8(3)	
O26	Hf1	Hf3	41.8(2)	
O26	Hf1	Hf2	95.0(3)	
O26	Hf1	O28	80.2(4)	
O26	Hf1	O13	131.5(5)	
O26	Hf1	O2	142.9(4)	
O26	Hf1	C1	106.0(10)	
O28	Hf1	Hf4	175.8(4)	
O28	Hf1	Hf3	119.3(3)	
O28	Hf1	Hf2	116.1(4)	
O28	Hf1	O13	55.9(5)	
O28	Hf1	C1	28.1(9)	
O13	Hf1	Hf4	123.3(4)	
O13	Hf1	Hf3	172.8(4)	
O13	Hf1	Hf2	87.8(4)	
O13	Hf1	C1	27.8(9)	
O2	Hf1	Hf4	73.2(4)	
O2	Hf1	Hf3	109.4(3)	
O2	Hf1	Hf2	110.3(4)	
O2	Hf1	O28	110.0(5)	
O2	Hf1	O13	77.8(5)	
O2	Hf1	C1	94.9(10)	
C1	Hf1	Hf4	150.8(9)	
C1	Hf1	Hf3	146.9(10)	
C1	Hf1	Hf2	102.4(9)	
Hf1	Hf4	Hf3	59.33(2)	
Hf1	Hf4	Hf5	90.09(2)	
Hf3	Hf4	Hf5	60.48(2)	
Hf2	Hf4	Hf1	60.31(2)	
Hf2	Hf4	Hf3	89.62(3)	
Hf2	Hf4	Hf5	59.54(2)	
O1	Hf4	Hf1	82.2(3)	
O1	Hf4	Hf3	81.0(3)	
O1	Hf4	Hf2	32.1(3)	
O1	Hf4	Hf5	30.1(3)	
O1	Hf4	O4	68.7(3)	
O1	Hf4	O8	70.2(4)	
O1	Hf4	O14	89.5(4)	
O1	Hf4	O18	85.0(5)	
O1	Hf4	O30	143.2(4)	
O1	Hf4	O41	139.9(4)	
O1	Hf4	O27	84.8(5)	
O4	Hf4	Hf1	95.5(2)	
O4	Hf4	Hf3	39.5(2)	
O4	Hf4	Hf2	95.0(2)	
O4	Hf4	Hf5	38.8(2)	
O8	Hf4	Hf1	38.6(3)	
O8	Hf4	Hf3	94.4(2)	
O8	Hf4	Hf2	38.3(3)	
O8	Hf4	Hf5	94.5(2)	
O8	Hf4	O4	121.2(3)	
O14	Hf4	Hf1	31.5(2)	
O14	Hf4	Hf3	30.4(3)	
O14	Hf4	Hf2	81.4(2)	
O14	Hf4	Hf5	81.2(3)	
O14	Hf4	O4	69.7(3)	
O14	Hf4	O8	69.8(4)	
O14	Hf4	O18	142.4(4)	
O14	Hf4	O30	85.2(5)	
O14	Hf4	O41	84.0(4)	
O14	Hf4	O27	141.9(5)	
O18	Hf4	Hf1	165.7(4)	
O18	Hf4	Hf3	112.2(3)	
O18	Hf4	Hf2	110.4(4)	
O18	Hf4	Hf5	75.6(4)	
O18	Hf4	O4	73.7(4)	
O18	Hf4	O8	140.4(4)	
O18	Hf4	O30	119.9(5)	
O18	Hf4	O41	76.8(5)	
O18	Hf4	O27	74.6(5)	
O30	Hf4	Hf1	74.5(4)	
O30	Hf4	Hf3	109.5(4)	
O30	Hf4	Hf2	111.2(3)	
O30	Hf4	Hf5	164.6(4)	
O30	Hf4	O4	140.5(4)	
O30	Hf4	O8	73.9(4)	
O30	Hf4	O27	77.6(6)	
O41	Hf4	Hf1	109.3(4)	
O41	Hf4	Hf3	73.5(3)	
O41	Hf4	Hf2	163.2(3)	
O41	Hf4	Hf5	109.9(3)	
O41	Hf4	O4	71.9(4)	
O41	Hf4	O8	141.0(4)	
O41	Hf4	O30	75.7(5)	
O41	Hf4	O27	122.9(5)	
O27	Hf4	Hf1	110.6(4)	
O27	Hf4	Hf3	163.5(4)	
O27	Hf4	Hf2	73.9(4)	
O27	Hf4	Hf5	109.2(4)	
O27	Hf4	O4	139.9(5)	
O27	Hf4	O8	72.9(4)	
Hf3	Hf6	Hf2	90.11(2)	
Hf3	Hf6	Hf5	61.70(2)	
Hf5	Hf6	Hf2	59.96(2)	
O6	Hf6	Hf3	85.0(3)	
O6	Hf6	Hf2	33.5(3)	
O6	Hf6	Hf5	86.2(3)	
O6	Hf6	O16	142.9(5)	
O6	Hf6	O20	143.6(5)	
O6	Hf6	O22	88.9(4)	
O6	Hf6	O26	69.0(4)	
O6	Hf6	O38	88.6(4)	
O6	Hf6	O11	70.0(4)	
O6	Hf6	C6	91.5(7)	
O16	Hf6	Hf3	74.5(4)	
O16	Hf6	Hf2	164.5(4)	
O16	Hf6	Hf5	109.5(3)	
O16	Hf6	O20	73.4(5)	
O16	Hf6	O22	108.8(5)	
O16	Hf6	O26	75.3(5)	
O16	Hf6	O38	76.2(5)	
O16	Hf6	O11	142.9(4)	
O16	Hf6	C6	89.7(8)	
O20	Hf6	Hf3	110.6(3)	
O20	Hf6	Hf2	111.4(4)	
O20	Hf6	Hf5	74.2(4)	
O20	Hf6	O22	76.4(5)	
O20	Hf6	O26	143.5(4)	
O20	Hf6	O38	108.8(5)	
O20	Hf6	O11	75.0(5)	
O20	Hf6	C6	91.2(8)	
O22	Hf6	Hf3	172.9(3)	
O22	Hf6	Hf2	86.7(4)	
O22	Hf6	Hf5	121.5(4)	
O22	Hf6	O38	55.8(6)	
O22	Hf6	C6	29.2(8)	
O26	Hf6	Hf3	41.8(2)	
O26	Hf6	Hf2	94.7(3)	
O26	Hf6	Hf5	99.5(3)	
O26	Hf6	O22	132.2(5)	
O26	Hf6	O38	80.9(5)	
O26	Hf6	C6	106.9(9)	
O38	Hf6	Hf3	120.4(4)	
O38	Hf6	Hf2	114.3(3)	
O38	Hf6	Hf5	174.3(3)	
O38	Hf6	C6	26.9(8)	
O21	Hf6	Hf3	31.8(3)	
O21	Hf6	Hf2	82.7(3)	
O21	Hf6	Hf5	33.1(3)	
O21	Hf6	O6	95.6(4)	
O21	Hf6	O16	83.1(4)	
O21	Hf6	O20	84.6(4)	
O21	Hf6	O22	153.2(5)	
O21	Hf6	O26	73.5(4)	
O21	Hf6	O38	150.5(5)	
O21	Hf6	O11	75.1(4)	
O21	Hf6	C6	172.4(7)	
O11	Hf6	Hf3	99.5(3)	
O11	Hf6	Hf2	36.6(3)	
O11	Hf6	Hf5	42.3(3)	
O11	Hf6	O22	81.7(5)	
O11	Hf6	O26	124.5(4)	
O11	Hf6	O38	133.1(5)	
O11	Hf6	C6	109.9(8)	
C6	Hf6	Hf3	147.2(8)	
C6	Hf6	Hf2	104.7(7)	
C6	Hf6	Hf5	150.7(8)	
Hf1	Hf3	Hf4	59.65(2)	
Hf1	Hf3	Hf6	60.91(2)	
Hf1	Hf3	Hf5	90.05(2)	
Hf4	Hf3	Hf5	60.09(2)	
Hf6	Hf3	Hf4	89.90(2)	
Hf6	Hf3	Hf5	59.29(2)	
O4	Hf3	Hf1	98.8(2)	
O4	Hf3	Hf4	42.8(2)	
O4	Hf3	Hf6	95.2(2)	
O4	Hf3	Hf5	37.7(2)	
O4	Hf3	O26	123.3(3)	
O10	Hf3	Hf1	105.3(3)	
O10	Hf3	Hf4	70.8(4)	
O10	Hf3	Hf6	160.5(4)	
O10	Hf3	Hf5	110.1(3)	
O10	Hf3	O4	72.5(4)	
O10	Hf3	O26	137.5(4)	
O14	Hf3	Hf1	31.8(3)	
O14	Hf3	Hf4	30.4(3)	
O14	Hf3	Hf6	82.0(2)	
O14	Hf3	Hf5	80.8(3)	
O14	Hf3	O4	72.9(4)	
O14	Hf3	O10	80.0(4)	
O14	Hf3	O24	85.2(4)	
O14	Hf3	O26	70.2(3)	
O14	Hf3	O34	144.4(4)	
O14	Hf3	O40	143.3(4)	
O14	Hf3	O21	90.6(4)	
O24	Hf3	Hf1	74.0(3)	
O24	Hf3	Hf4	109.6(4)	
O24	Hf3	Hf6	111.2(3)	
O24	Hf3	Hf5	164.0(3)	
O24	Hf3	O4	143.0(5)	
O24	Hf3	O10	74.7(5)	
O24	Hf3	O26	73.3(4)	
O24	Hf3	O34	112.4(4)	
O24	Hf3	O40	79.0(5)	
O26	Hf3	Hf1	38.7(2)	
O26	Hf3	Hf4	94.6(2)	
O26	Hf3	Hf6	38.8(3)	
O26	Hf3	Hf5	94.5(3)	
O34	Hf3	Hf1	173.4(3)	
O34	Hf3	Hf4	115.4(3)	
O34	Hf3	Hf6	116.4(3)	
O34	Hf3	Hf5	83.5(3)	
O34	Hf3	O4	75.2(4)	
O34	Hf3	O10	75.7(5)	
O34	Hf3	O26	143.1(4)	
O40	Hf3	Hf1	111.5(3)	
O40	Hf3	Hf4	163.2(3)	
O40	Hf3	Hf6	73.4(3)	
O40	Hf3	Hf5	108.0(3)	
O40	Hf3	O4	135.1(4)	
O40	Hf3	O10	126.0(5)	
O40	Hf3	O26	73.6(4)	
O40	Hf3	O34	72.1(4)	
O21	Hf3	Hf1	82.7(2)	
O21	Hf3	Hf4	82.4(3)	
O21	Hf3	Hf6	31.0(3)	
O21	Hf3	Hf5	31.5(3)	
O21	Hf3	O4	69.2(4)	
O21	Hf3	O10	141.6(4)	
O21	Hf3	O24	141.9(4)	
O21	Hf3	O26	69.7(4)	
O21	Hf3	O34	92.5(4)	
O21	Hf3	O40	82.3(4)	
Hf4	Hf2	Hf1	59.96(2)	
Hf4	Hf2	Hf5	61.36(2)	
Hf5	Hf2	Hf1	90.94(2)	
O1	Hf2	Hf1	81.7(3)	
O1	Hf2	Hf4	31.9(3)	
O1	Hf2	Hf5	32.0(3)	
O1	Hf2	O6	94.8(4)	
O1	Hf2	O8	74.6(4)	
O1	Hf2	O11	73.7(4)	
O1	Hf2	O23	83.3(4)	
O1	Hf2	O3	84.1(4)	
O1	Hf2	O7	150.9(5)	
O1	Hf2	O31	151.7(5)	
O1	Hf2	C26	174.7(9)	
O6	Hf2	Hf1	32.5(3)	
O6	Hf2	Hf4	85.5(3)	
O6	Hf2	Hf5	86.5(3)	
O6	Hf2	O8	69.5(4)	
O6	Hf2	O11	71.1(4)	
O6	Hf2	O23	144.8(5)	
O6	Hf2	O3	141.8(5)	
O6	Hf2	O7	90.8(5)	
O6	Hf2	O31	88.1(4)	
O6	Hf2	C26	90.3(9)	
O8	Hf2	Hf1	37.2(3)	
O8	Hf2	Hf4	43.0(3)	
O8	Hf2	Hf5	100.5(3)	
O8	Hf2	O23	141.5(5)	
O8	Hf2	O3	73.4(5)	
O8	Hf2	O7	133.6(5)	
O8	Hf2	O31	80.1(5)	
O8	Hf2	C26	106.1(11)	
O11	Hf2	Hf1	95.7(3)	
O11	Hf2	Hf4	99.4(3)	
O11	Hf2	Hf5	42.0(3)	
O11	Hf2	O8	126.2(4)	
O11	Hf2	O23	74.7(5)	
O11	Hf2	O3	142.7(5)	
O11	Hf2	O7	81.2(5)	
O11	Hf2	O31	133.1(5)	
O11	Hf2	C26	109.3(11)	
O23	Hf2	Hf1	164.1(3)	
O23	Hf2	Hf4	108.3(3)	
O23	Hf2	Hf5	73.4(3)	
O23	Hf2	O7	75.9(5)	
O23	Hf2	O31	110.0(5)	
O23	Hf2	C26	93.2(9)	
O3	Hf2	Hf1	110.5(4)	
O3	Hf2	Hf4	73.5(3)	
O3	Hf2	Hf5	109.4(4)	
O3	Hf2	O23	73.2(5)	
O3	Hf2	O7	108.5(5)	
O3	Hf2	O31	76.6(5)	
O3	Hf2	C26	91.1(10)	
O7	Hf2	Hf1	115.9(4)	
O7	Hf2	Hf4	175.8(4)	
O7	Hf2	Hf5	120.4(4)	
O7	Hf2	O31	56.9(6)	
O7	Hf2	C26	29.9(10)	
O31	Hf2	Hf1	85.9(4)	
O31	Hf2	Hf4	120.9(4)	
O31	Hf2	Hf5	173.9(4)	
O31	Hf2	C26	27.0(10)	
C26	Hf2	Hf1	102.1(9)	
C26	Hf2	Hf4	147.9(10)	
C26	Hf2	Hf5	150.2(11)	
Hf4	Hf5	Hf3	59.43(2)	
Hf6	Hf5	Hf4	89.34(3)	
Hf6	Hf5	Hf3	59.00(2)	
Hf6	Hf5	Hf2	60.27(2)	
Hf2	Hf5	Hf4	59.10(2)	
Hf2	Hf5	Hf3	88.75(2)	
O1	Hf5	Hf4	29.9(3)	
O1	Hf5	Hf6	81.7(3)	
O1	Hf5	Hf3	79.8(3)	
O1	Hf5	Hf2	31.9(3)	
O1	Hf5	O4	72.0(3)	
O1	Hf5	O32	140.7(5)	
O1	Hf5	O36	145.2(5)	
O1	Hf5	O21	89.6(4)	
O1	Hf5	O11	69.5(4)	
O1	Hf5	O25	78.9(5)	
O1	Hf5	O15	85.1(5)	
O4	Hf5	Hf4	42.3(2)	
O4	Hf5	Hf6	95.1(2)	
O4	Hf5	Hf3	37.9(2)	
O4	Hf5	Hf2	97.8(2)	
O4	Hf5	O11	122.3(3)	
O4	Hf5	O25	72.3(4)	
O32	Hf5	Hf4	160.6(4)	
O32	Hf5	Hf6	71.3(4)	
O32	Hf5	Hf3	108.1(5)	
O32	Hf5	Hf2	108.8(4)	
O32	Hf5	O4	136.9(5)	
O32	Hf5	O11	71.6(4)	
O32	Hf5	O25	129.0(6)	
O36	Hf5	Hf4	117.8(4)	
O36	Hf5	Hf6	120.5(4)	
O36	Hf5	Hf3	89.3(3)	
O36	Hf5	Hf2	176.9(4)	
O36	Hf5	O4	79.2(4)	
O36	Hf5	O32	74.2(5)	
O36	Hf5	O11	144.8(5)	
O36	Hf5	O25	74.0(5)	
O36	Hf5	O15	107.1(5)	
O21	Hf5	Hf4	81.1(3)	
O21	Hf5	Hf6	31.5(3)	
O21	Hf5	Hf3	30.7(3)	
O21	Hf5	Hf2	81.8(2)	
O21	Hf5	O4	68.6(3)	
O21	Hf5	O32	82.1(5)	
O21	Hf5	O36	97.8(4)	
O21	Hf5	O11	69.8(4)	
O21	Hf5	O25	140.9(4)	
O21	Hf5	O15	143.4(4)	
O11	Hf5	Hf4	93.4(2)	
O11	Hf5	Hf6	38.7(3)	
O11	Hf5	Hf3	93.6(3)	
O11	Hf5	Hf2	37.8(2)	
O25	Hf5	Hf4	70.3(4)	
O25	Hf5	Hf6	159.4(4)	
O25	Hf5	Hf3	110.2(3)	
O25	Hf5	Hf2	104.4(3)	
O25	Hf5	O11	136.1(4)	
O15	Hf5	Hf4	109.0(4)	
O15	Hf5	Hf6	112.0(3)	
O15	Hf5	Hf3	163.4(4)	
O15	Hf5	Hf2	74.7(4)	
O15	Hf5	O4	141.6(4)	
O15	Hf5	O32	79.4(6)	
O15	Hf5	O11	74.4(4)	
O15	Hf5	O25	73.3(5)	
Hf4	O1	Hf2	115.9(5)	
Hf4	O1	Hf5	120.0(4)	
Hf2	O1	Hf5	116.1(4)	
Hf3	O4	Hf4	97.8(3)	
Hf5	O4	Hf4	99.0(3)	
Hf5	O4	Hf3	104.4(4)	
Hf1	O6	Hf6	114.6(5)	
Hf1	O6	Hf2	114.5(4)	
Hf2	O6	Hf6	112.5(5)	
Hf1	O8	Hf4	98.5(4)	
Hf2	O8	Hf1	106.0(4)	
Hf2	O8	Hf4	98.7(4)	
C8	O10	Hf3	133.7(16)	
C4	O12	Hf1	134.1(16)	
Hf1	O14	Hf4	117.0(4)	
Hf1	O14	Hf3	116.2(4)	
Hf4	O14	Hf3	119.2(4)	
C12	O16	Hf6	133.6(13)	
C20	O18	Hf4	134.5(13)	
C16	O20	Hf6	134(2)	
C6	O22	Hf6	92.6(15)	
C4	O24	Hf3	131.7(15)	
Hf1	O26	Hf6	106.0(4)	
Hf1	O26	Hf3	99.5(4)	
Hf6	O26	Hf3	99.4(4)	
C1	O28	Hf1	92(2)	
C22	O30	Hf4	133.0(11)	
C16	O32	Hf5	139(2)	
C18	O36	Hf5	148.1(14)	
C6	O38	Hf6	95(2)	
C12	O40	Hf3	134.5(12)	
C8	O41	Hf4	137.4(19)	
Hf6	O21	Hf3	117.1(5)	
Hf6	O21	Hf5	115.4(5)	
Hf3	O21	Hf5	117.8(4)	
Hf6	O11	Hf5	99.1(4)	
Hf2	O11	Hf6	106.2(4)	
Hf2	O11	Hf5	100.1(4)	
C14	O23	Hf2	133.5(14)	
C32	O3	Hf2	133.9(14)	
C20	O25	Hf5	133.2(14)	
C1	O13	Hf1	92(2)	
C32	O27	Hf4	133.7(13)	
C26	O7	Hf2	91.7(18)	
C14	O15	Hf5	132.2(13)	
C26	O31	Hf2	92(2)	
C22	O2	Hf1	131.9(14)	
O28	C1	Hf1	59.6(19)	
O28	C1	C38	124(4)	
O13	C1	Hf1	60.1(18)	
O13	C1	O28	120(4)	
O13	C1	C38	116(3)	
C38	C1	Hf1	175(3)	
O12	C4	O24	125(2)	
O12	C4	C30	119(3)	
O24	C4	C30	116(2)	
O22	C6	Hf6	58.2(13)	
O22	C6	C42	116(2)	
O38	C6	Hf6	58.3(15)	
O38	C6	O22	116(3)	
O38	C6	C42	128(3)	
C42	C6	Hf6	174(2)	
O10	C8	C24	110(3)	
O41	C8	O10	124(2)	
O41	C8	C24	126(3)	
O33	C10	O37	124(4)	
O33	C10	C34	128(4)	
O37	C10	C34	108(4)	
O16	C12	O40	123.9(18)	
O16	C12	C50	119(2)	
O40	C12	C50	117(2)	
O23	C14	O15	126(2)	
O23	C14	C27	118(2)	
O15	C14	C27	115(2)	
O20	C16	C52	111(3)	
O32	C16	O20	122(4)	
O32	C16	C52	128(4)	
O36	C18	O29	123.0(18)	
O36	C18	C44	125.8(19)	
O29	C18	C44	110.4(16)	
O18	C20	O25	125.6(19)	
O18	C20	C2	114(3)	
O25	C20	C2	119(3)	
O30	C22	C40	120(2)	
O2	C22	O30	127.4(18)	
O2	C22	C40	111(2)	
C60	C24	C8	112(2)	
C60	C24	C7	113.3(18)	
C7	C24	C8	107(2)	
O7	C26	Hf2	58.4(13)	
O7	C26	C62	115(3)	
O31	C26	Hf2	60.7(15)	
O31	C26	O7	119(3)	
O31	C26	C62	126(4)	
C62	C26	Hf2	173(3)	
O17	C28	C64	122(3)	
O9	C28	O17	113(3)	
O9	C28	C64	118(3)	
C4	C30	C25	110.6(19)	
C51	C30	C4	109.6(18)	
C51	C30	C25	105.4(16)	
C76	C30	C4	111(2)	
C76	C30	C25	108.8(19)	
O3	C32	O27	124.8(19)	
O3	C32	C73	107(2)	
O27	C32	C73	126(3)	
C56	C34	C10	113(3)	
C56	C34	C45	109(3)	
C45	C34	C10	102(3)	
C70	C38	C1	110(3)	
C70	C38	C5	109(3)	
C5	C38	C1	102(3)	
C49	C40	C22	105(3)	
C49	C40	C75	118(3)	
C75	C40	C22	112(3)	
C72	C42	C6	104(2)	
C72	C42	C80	136(2)	
C80	C42	C6	105.5(18)	
C74	C44	C18	113(2)	
C74	C44	C78	123(2)	
C78	C44	C18	111(2)	
O39	C48	C69	108(3)	
O39	C48	O5	117(3)	
O5	C48	C69	129(3)	
C43	C50	C12	111(3)	
C57	C50	C12	100(2)	
C57	C50	C77	116(2)	
C77	C50	C12	108(3)	
C79	C50	C12	111(3)	
C79	C50	C43	119(4)	
C16	C52	C47	133(3)	
C11	C52	C16	106(3)	
C11	C52	C47	96.9(17)	
C29	C54	C69	103(2)	
C34	C56	C17	106(3)	
C68	C62	C26	110(3)	
C68	C62	C13	113(3)	
C13	C62	C26	110(3)	
C28	C64	C21	94(3)	
C28	C64	C59	155(3)	
C28	C64	C81	108(2)	
C59	C64	C21	109.2(18)	
C81	C64	C21	103(2)	
C86	C72	C42	133(2)	
C85	C78	C44	104.3(9)	
C15	C5	C38	115(3)	
C64	C21	C82	96(2)	
C128	C47	C52	92.8(13)	
C40	C49	C37	104(2)	
C88	C25	C30	108.7(19)	
C87	C13	C62	112(2)	
C41	C27	C14	106(3)	
C41	C27	C67	101(3)	
C67	C27	C14	105(3)	
C84	C7	C24	114(2)	
C50	C57	C83	106(2)	
C46	C63	C2	115(3)	
C63	C2	C20	102(2)	
C63	C2	C71	110(2)	
C71	C2	C20	127(4)	
C48	C69	C54	114(2)	
C48	C69	C19	99(3)	
C19	C69	C54	96(3)	
C3	C9	C73	99.1(12)	
C32	C73	C55	119(3)	
C32	C73	C9	96(3)	
C9	C73	C55	93.1(18)	
C50	C79	C83	118(4)	


Table 0: Torsion Angles in ° for mjp169_150k_new.


Atom	Atom	Atom	Atom	Angle/°	
Hf1	O12	C4	O24	11(6)	
Hf1	O12	C4	C30	-168.6(15)	
Hf1	O28	C1	O13	3(4)	
Hf1	O28	C1	C38	176(3)	
Hf1	O13	C1	O28	-3(4)	
Hf1	O13	C1	C38	-176(3)	
Hf1	O2	C22	O30	1(4)	
Hf1	O2	C22	C40	-166.4(15)	
Hf4	O18	C20	O25	13(3)	
Hf4	O18	C20	C2	-153.5(17)	
Hf4	O30	C22	O2	-1(4)	
Hf4	O30	C22	C40	166.0(15)	
Hf4	O41	C8	O10	-1(5)	
Hf4	O41	C8	C24	169.6(16)	
Hf4	O27	C32	O3	-5(4)	
Hf4	O27	C32	C73	-166.1(17)	
Hf6	O16	C12	O40	0(3)	
Hf6	O16	C12	C50	175.5(15)	
Hf6	O20	C16	O32	5(7)	
Hf6	O20	C16	C52	-178.1(16)	
Hf6	O22	C6	O38	-10(3)	
Hf6	O22	C6	C42	177(2)	
Hf6	O38	C6	O22	10(3)	
Hf6	O38	C6	C42	-178(3)	
Hf3	O10	C8	O41	-6(5)	
Hf3	O10	C8	C24	-178.0(11)	
Hf3	O24	C4	O12	-15(5)	
Hf3	O24	C4	C30	164.8(15)	
Hf3	O40	C12	O16	2(3)	
Hf3	O40	C12	C50	-174.0(15)	
Hf2	O23	C14	O15	2(4)	
Hf2	O23	C14	C27	-174.3(16)	
Hf2	O3	C32	O27	4(4)	
Hf2	O3	C32	C73	168.0(15)	
Hf2	O7	C26	O31	4(4)	
Hf2	O7	C26	C62	-178(3)	
Hf2	O31	C26	O7	-4(4)	
Hf2	O31	C26	C62	178(3)	
Hf5	O32	C16	O20	-9(7)	
Hf5	O32	C16	C52	175(2)	
Hf5	O36	C18	O29	-56(4)	
Hf5	O36	C18	C44	136(2)	
Hf5	O25	C20	O18	-6(3)	
Hf5	O25	C20	C2	159.6(16)	
Hf5	O15	C14	O23	0(3)	
Hf5	O15	C14	C27	177.2(16)	
O10	C8	C24	C60	132(3)	
O10	C8	C24	C7	-103(3)	
O12	C4	C30	C25	104(3)	
O12	C4	C30	C51	-12(4)	
O12	C4	C30	C76	-135(4)	
O16	C12	C50	C43	-41(4)	
O16	C12	C50	C57	123(3)	
O16	C12	C50	C77	2(4)	
O16	C12	C50	C79	94(4)	
O18	C20	C2	C63	-106(3)	
O18	C20	C2	C71	127(3)	
O20	C16	C52	C11	104(3)	
O20	C16	C52	C47	-140(3)	
O22	C6	C42	C72	-96(3)	
O22	C6	C42	C80	118(2)	
O24	C4	C30	C25	-76(3)	
O24	C4	C30	C51	168(4)	
O24	C4	C30	C76	45(4)	
O28	C1	C38	C70	44(5)	
O28	C1	C38	C5	-72(4)	
O30	C22	C40	C49	133(3)	
O30	C22	C40	C75	3(3)	
O32	C16	C52	C11	-80(5)	
O32	C16	C52	C47	36(7)	
O36	C18	C44	C74	-140(2)	
O36	C18	C44	C78	3(3)	
O38	C6	C42	C72	93(3)	
O38	C6	C42	C80	-53(4)	
O40	C12	C50	C43	135(3)	
O40	C12	C50	C57	-61(3)	
O40	C12	C50	C77	178(3)	
O40	C12	C50	C79	-90(4)	
O41	C8	C24	C60	-40(4)	
O41	C8	C24	C7	85(4)	
O23	C14	C27	C41	-107(3)	
O23	C14	C27	C67	147(3)	
O3	C32	C73	C55	-128(3)	
O3	C32	C73	C9	135(2)	
O25	C20	C2	C63	87(3)	
O25	C20	C2	C71	-40(4)	
O13	C1	C38	C70	-143(3)	
O13	C1	C38	C5	101(4)	
O27	C32	C73	C55	36(4)	
O27	C32	C73	C9	-61(3)	
O7	C26	C62	C68	-123(4)	
O7	C26	C62	C13	112(4)	
O29	C18	C44	C74	51(3)	
O29	C18	C44	C78	-166.2(18)	
O15	C14	C27	C41	75(3)	
O15	C14	C27	C67	-30(3)	
O31	C26	C62	C68	55(5)	
O31	C26	C62	C13	-70(5)	
O2	C22	C40	C49	-58(3)	
O2	C22	C40	C75	172(2)	
O33	C10	C34	C56	118(4)	
O33	C10	C34	C45	-126(5)	
O17	C28	C64	C21	70(4)	
O17	C28	C64	C59	-133(8)	
O17	C28	C64	C81	-35(5)	
C1	C38	C5	C15	-65(4)	
O9	C28	C64	C21	-141(3)	
O9	C28	C64	C59	16(10)	
O9	C28	C64	C81	114(4)	
C4	C30	C25	C88	158(3)	
C6	C42	C72	C86	-26(4)	
C8	C24	C7	C84	69(3)	
C10	C34	C56	C17	-64(4)	
C12	C50	C57	C83	-178(3)	
C12	C50	C79	C83	146(4)	
O37	C10	C34	C56	-60(4)	
O37	C10	C34	C45	57(4)	
C16	C52	C47	C128	-15(4)	
C18	C44	C78	C85	-73(3)	
C22	C40	C49	C37	-63(4)	
C26	C62	C13	C87	-53(4)	
C28	C64	C21	C82	82(3)	
C46	C63	C2	C20	56(5)	
C46	C63	C2	C71	-167(4)	
C60	C24	C7	C84	-167(2)	
C68	C62	C13	C87	-177(3)	
C70	C38	C5	C15	178(3)	
C74	C44	C78	C85	66(3)	
C80	C42	C72	C86	104(4)	
C43	C50	C79	C83	-84(6)	
C11	C52	C47	C128	105(2)	
C45	C34	C56	C17	-177(3)	
C3	C9	C73	C32	-70(4)	
C3	C9	C73	C55	170(3)	
C51	C30	C25	C88	-84(3)	
C29	C54	C69	C48	-58(5)	
C29	C54	C69	C19	-161(4)	
C59	C64	C21	C82	-87(3)	
O39	C48	C69	C54	139(3)	
O39	C48	C69	C19	-120(4)	
O5	C48	C69	C54	-11(7)	
O5	C48	C69	C19	89(5)	
C75	C40	C49	C37	63(5)	
C77	C50	C57	C83	-62(5)	
C81	C64	C21	C82	-168(3)	
C76	C30	C25	C88	36(4)	


Table 0: Hydrogen Fractional Atomic Coordinates (×104) and Equivalent Isotropic Displacement Parameters (Å2×103) for mjp169_150k_new. Ueq is defined as 1/3 of the trace of the orthogonalised Uij.

Atom	x	y	z	Ueq	
H4	5302.71	4410.2	8728.69	137	
H8	3690.02	1817.58	6682.67	157	
H26	1313.34	1790.24	8354.22	144	
H34A	4334.28	4623.51	9350.28	183	
H34B	4251.92	4175.1	9806.99	183	
H11	2240.72	4618.47	7048.83	153	
H17	-654.51	1253.45	7958.82	302	
H37	7876.51	-516.55	4113.57	337	
H24	5957.54	2355.14	9712.77	240	
H30	2221.94	189.16	9429.4	294	
H30A	2096.44	-497.04	8863.16	294	
H34	5540.12	-420.32	4760.65	329	
H38	147.05	-540.5	6124.28	263	
H40	4428.85	13.48	6805.05	360	
H42	-1356.43	2692.86	6575.8	217	
H44	5521.46	7211.56	9667.6	240	
H46A	7558.82	4912.72	8713.98	450	
H46B	8608.62	5308.02	8467	450	
H46C	8394.59	5860.25	9000.2	450	
H50	1075.34	3848.94	9859.85	240	
H50A	1180.97	3558.01	9910.61	240	
H52	809.84	5883.97	7907.7	296	
H54A	-38.36	5488.74	5451.89	360	
H54B	-105.73	6487.33	5619.28	360	
H56A	6591.54	-132.9	5639.79	329	
H56B	7542.17	-138.1	5319.96	329	
H60A	6189.72	1304.46	9033.28	300	
H60B	7109.7	2094.2	8843.77	300	
H60C	7212.95	1797.22	9465.68	300	
H62	512.22	2720.05	5244.22	260	
H64	-1715.25	8.32	8903.04	296	
H64A	-666.68	-215.94	9183.13	296	
H68A	707.64	1045.47	5259.76	324	
H68B	-67.81	1311.05	5619.11	324	
H68C	-263.04	1216.77	4943.66	324	
H70A	-1134.04	-372.87	6951.12	328	
H70B	-996.47	265.91	6453.04	328	
H70C	-1543.91	-816.78	6309.44	328	
H72A	-2031.96	3310.92	7051.52	217	
H72B	-2659.7	2316.5	7218.26	217	
H74A	7060.36	7153.12	9766.12	300	
H74B	7209.47	7299.99	9123.92	300	
H74C	7189.55	8127.42	9553.46	300	
H78A	5405.86	8360.87	9177.45	240	
H78B	4489.01	7406.69	8964.48	240	
H80A	-1168.21	1345.25	6370.11	272	
H80B	-1608.94	949.06	6920.83	272	
H80C	-2348.03	1120.11	6418.95	272	
H5A	-185.38	-1254.47	7178.42	263	
H5B	-545.56	-1833.11	6558.4	263	
H21A	-1820.32	-848.48	8007.22	240	
H21B	-2048.17	-1419.99	8541.53	240	
H43A	696.85	4696.14	9391.3	300	
H43B	-283.18	3804.8	9184.19	300	
H43C	-23.08	4225.3	9834.8	300	
H11A	1332.01	5839.88	8952.44	370	
H11B	2383.43	6636.57	8922.79	370	
H11C	1343.42	6849.47	8866.06	370	
H45A	7426.45	1034.01	4807.39	412	
H45B	6429.01	1062.88	4430.79	412	
H45C	6462.53	1048.72	5099.06	412	
H47A	1422.27	7772.07	8150.71	296	
H47B	1402.86	7200.68	7556.21	296	
H3A	6216.27	4784.09	5613.27	450	
H3B	5947.12	4693.05	6242.17	450	
H3C	7096.13	5191.64	6142.32	450	
H49A	4468.57	-1166.78	7362.66	360	
H49B	3628.63	-753.63	7556.38	360	
H25A	4092.19	705.22	9396.99	294	
H25B	3816.26	-189.17	8941.06	294	
H51A	1305.97	-796.94	8599.41	367	
H51B	2320.88	-893.75	8405.77	367	
H51C	1933.39	-1299.84	8965.42	367	
H13A	990.95	2239.22	4406.55	260	
H13B	1901.5	2052.4	4792.3	260	
H27	4017.53	6091.88	5907.47	336	
H55A	6582.35	2255.13	6045.45	450	
H55B	5464.26	1601.6	5770.89	450	
H55C	6247.58	2146.25	5376.98	450	
H7A	7390.66	3816.8	9336.66	240	
H7B	7730.42	3327.17	9854.98	240	
H57A	93.03	1833.6	9577.15	240	
H57B	1208.43	2463.85	9893.54	240	
H29A	1132.97	6053.75	4980.99	450	
H29B	1467.36	6765.81	5542.71	450	
H29C	1669.16	5815.69	5542.71	450	
H59A	-683.32	-575.76	9564.8	300	
H59B	-1385.62	34.13	9644.8	300	
H59C	-1881.37	-1002.11	9356.2	300	
H63A	7450.21	6258.75	8400.49	360	
H63B	8510.72	6658.09	8150.92	360	
H2	7788.95	5249.88	7618.25	360	
H69	1132.69	6474.76	6523.98	360	
H71A	7189.62	6773.47	7316.5	450	
H71B	8249.32	6625.54	7265.46	450	
H71C	7235.05	5976.27	6871.43	450	
H9A	6937.49	3689.72	6392	360	
H9B	7209.36	3781.66	5756.6	360	
H73	5230.16	3207.49	5475.97	360	
H5	575.59	3971.04	6063.21	351	
H37A	5676.54	-152.63	8170.54	450	
H37B	4916.67	336.1	8371.96	450	
H37C	4635.9	-744.54	8371.96	450	
H75A	6047.04	887.34	6892.77	450	
H75B	6238.16	646.8	7523.01	450	
H75C	6062.27	-108.86	6996.58	450	
H77A	-118.72	4138.64	9255.76	300	
H77B	-662.63	3108.72	8963.07	300	
H77C	-671.24	3353.4	9622.26	300	
H79A	-616.57	2561.2	9490.48	240	
H79B	169.31	2007.17	9476.21	240	
H81A	-1790.58	739.38	9160.45	300	
H81B	-2298.26	118.55	8571.12	300	
H81C	-2444.89	-318.84	9151.84	300	
H76A	2480.3	760.77	9825.89	367	
H76B	1372.76	198.53	9490.81	367	
H76C	2009.4	-321.68	9833.55	367	
H82A	-318.43	-1318.28	8800.71	300	
H82B	-777.11	-1802.89	8170.88	300	
H82C	-86.42	-743.95	8275.85	300	
H83A	-757.58	2523.53	10118.65	300	
H83B	-270.37	1909.35	10477.3	300	
H83C	351.78	2972.95	10477.3	300	
H83D	-566.05	1828.69	10336.2	300	
H83E	579.74	2523.65	10410.24	300	
H83F	-341.64	2908.03	10410.24	300	
H84A	6388.16	4336.53	9767.05	300	
H84B	6392.03	3639.83	10223.01	300	
H84C	7375.05	4525.73	10223.01	300	
H85A	4966.01	7581.3	8128.33	300	
H85B	6024.52	8359.62	8383.58	300	
H85C	5907.35	7304.47	8383.58	300	
H12A	2927.52	7401.54	7435.66	370	
H12B	2952.28	8002.64	8011.3	370	
H12C	2819.92	6944.35	8011.3	370	
H86A	-1801.24	3755.43	7869.15	272	
H86B	-2397.17	2770.16	8042.52	272	
H86C	-1193.94	3089.78	8042.52	272	
H87A	2243.11	3499.45	4392.36	324	
H87B	1884.45	3733.78	4977.74	324	
H87C	2870.58	3440.39	4977.74	324	
H88A	4256.87	-463.19	9895.54	367	
H88B	3366.11	-152.12	10102.45	367	
H88C	3095.49	-1037.13	9651.33	367	
H17A	6252.58	-1695.46	4875.89	412	
H17B	6964.22	-1605.73	5460.85	412	
H17C	5815.02	-1643.34	5460.85	412	
H15A	1102.89	-1695.99	6392.62	328	
H15B	1510.45	-1038.66	6976.61	328	
H15C	866.37	-2097.25	6976.61	328	
H19A	-331.47	5408.29	6786.09	461	
H19B	-704.58	6111.09	6447.79	461	
H19C	-886.95	5107.72	6147.81	461	


Table 0: Atomic Occupancies for all atoms that are not fully occupied in mjp169_150k_new.


Atom	Occupancy	
H30	0.5	
H30A	0.5	
H50	0.5	
H50A	0.5	
H64	0.5	
H64A	0.5	
C43	0.5	
H43A	0.5	
H43B	0.5	
H43C	0.5	
C51	0.5	
H51A	0.5	
H51B	0.5	
H51C	0.5	
C57	0.5	
H57A	0.5	
H57B	0.5	
C59	0.5	
H59A	0.5	
H59B	0.5	
H59C	0.5	
C77	0.5	
H77A	0.5	
H77B	0.5	
H77C	0.5	
C79	0.5	
H79A	0.5	
H79B	0.5	
C81	0.5	
H81A	0.5	
H81B	0.5	
H81C	0.5	
C76	0.5	
H76A	0.5	
H76B	0.5	
H76C	0.5	
H83A	0.5	
H83B	0.5	
H83C	0.5	
H83D	0.5	
H83E	0.5	
H83F	0.5	


Citations
O.V. Dolomanov and L.J. Bourhis and R.J. Gildea and J.A.K. Howard and H. Puschmann, Olex2: A complete structure solution, refinement and analysis program, J. Appl. Cryst., (2009), 42, 339-341.
STOE & Cie GmbH, X-Area, software package for collecting single-crystal or multi-domain crystal data on STOE area-detector diffractometers, for image  processing, for the correction and scaling of reflection intensities and  for outlier rejection, version 1.90, Darmstadt 2020
Sheldrick, G.M., Crystal structure refinement with ShelXL, Acta Cryst., (2015), C71, 3-8.
Sheldrick, G.M., ShelXT-Integrated space-group and crystal-structure determination, Acta Cryst., (2015), A71, 3-8.
X-Area Integrate 1.78.3.0
X-Area Pilatus3_SV 1.31.170.0 (STOE, 2020)
X-Area Recipe 1.36.0.0
