# Supplementary material for: Mechanism‐Guided Precision Hydrolysis of Early Transition Metals to Access (Mixed‐Metal) Oxo Clusters
Source: Angew Chem Int Ed Engl. 2026 Feb 24;65(15):e25769. doi: 10.1002/anie.202525769 (PMC13053926; doi:10.1002/anie.202525769)

checkCIF/PLATON report

Structure factors have been supplied for datablock(s) mjp169_150k_new

THIS REPORT IS FOR GUIDANCE ONLY. IF USED AS PART OF A REVIEW PROCEDURE FOR PUBLICATION, IT SHOULD NOT REPLACE THE EXPERTISE OF AN EXPERIENCED CRYSTALLOGRAPHIC REFEREE.

No syntax errors found. CIF dictionary Interpreting this report

Datablock: mjp169_150k_new

```
Bond precision:   C-C = 0.0455 A           Wavelength=1.54186

Cell:            a=14.0863(5)      b=15.6140(6)      c=23.9826(8)
                 alpha=94.310(3)   beta=96.311(3)    gamma=109.356(3)
Temperature:     150 K
```

	Calculated	Reported
Volume	4911.4(3)	4911.4(3)
Space group	P -1	P -1
Hall group	-P 1	-P 1
Moiety formula	C57 H101 Hf6 O33, 3(C5 H10 O2), C2 H5	C59 H106 Hf6 O33, 3(C5 H10 O2)
Sum formula	C74 H136 Hf6 O39	C74 H136 Hf6 O39
Mr	2720.77	2720.76
Dx,g cm-3	1.840	1.840
Z	2	2
Mu (mm-1)	11.996	11.997
F000	2648.0	2648.0
F000’	2580.45	
h,k,lmax	17,19,29	17,19,28
Nref	18808	18232
Tmin,Tmax	0.285,0.487	0.003,0.036
Tmin’	0.183	

```
Correction method= # Reported T Limits: Tmin=0.003 Tmax=0.036
AbsCorr = MULTI-SCAN
```

Data completeness= 0.969 Theta(max)= 70.479

R(reflections)= 0.0881(10640) wR2(reflections)= 0.2521(18232)

S = 1.052 Npar= 703

The following ALERTS were generated. Each ALERT has the format
test-name_ALERT_alert-type_alert-level.
Click on the hyperlinks for more details of the test.

Alert level A

PLAT201_ALERT_2_A Isotropic non-H Atoms in Main Residue(s) .... 22 Report

C2	C3	C9	C14	C27	C37	C40	C41
C44	C46	C49	C50	C55	C63	C67	C71
C73	C74	C75	C78	C83	C85		

Author Response: Due to the data quality and amount of disorder, several atoms had to be refiend isotropically.

Alert level B

PLAT230_ALERT_2_B Hirshfeld Test Diff for O28 --C1 . 9.2 s.u.

Author Response: These alerts are due to O atoms strongly bonded to a Hf atom or H-bonding and their neighouring C atom that is part of either a disordered or highly vibrating ligand.

PLAT230_ALERT_2_B Hirshfeld Test Diff for O41 --C8 . 9.3 s.u.

Author Response: These alerts are due to O atoms strongly bonded to a Hf atom or H-bonding and their neighouring C atom that is part of either a disordered or highly vibrating ligand.

PLAT234_ALERT_4_B Large Hirshfeld Difference O7 --C26 . 0.27 Ang.

Author Response: These alerts are due to O atoms strongly bonded to a Hf atom or H-bonding and their neighouring C atom that is part of either a disordered or highly vibrating ligand.

PLAT234_ALERT_4_B Large Hirshfeld Difference O18 --C20 . 0.26 Ang.

Author Response: These alerts are due to O atoms strongly bonded to a Hf atom or H-bonding and their neighouring C atom that is part of either a disordered or highly vibrating ligand.

PLAT241_ALERT_2_B High 'MainMol' Ueq as Compared to Neighbors of C1 Check

Author Response: These alerts are due to O atoms strongly bonded to a Hf atom or H-bonding and their neighouring C atom that is part of either a disordered or highly vibrating ligand.

PLAT241_ALERT_2_B High 'MainMol' Ueq as Compared to Neighbors of C2 Check

Author Response: These alerts are due to O atoms strongly bonded to a Hf atom or H-bonding and their neighouring C atom that is part of either a disordered or highly vibrating ligand.

PLAT241_ALERT_2_B High 'MainMol' Ueq as Compared to Neighbors of C4 Check

Author Response: These alerts are due to O atoms strongly bonded to a Hf atom or H-bonding and their neighouring C atom that is part of either a disordered or highly vibrating ligand.

PLAT241_ALERT_2_B High 'MainMol' Ueq as Compared to Neighbors of C8 Check

Author Response: These alerts are due to O atoms strongly bonded to a Hf atom or H-bonding and their neighouring C atom that is part of either a disordered or highly vibrating ligand.

PLAT241_ALERT_2_B High 'MainMol' Ueq as Compared to Neighbors of C16 Check

Author Response: These alerts are due to O atoms strongly bonded to a Hf atom or H-bonding and their neighouring C atom that is part of either a disordered or highly vibrating ligand.

PLAT241_ALERT_2_B High 'MainMol' Ueq as Compared to Neighbors of C26 Check

Author Response: These alerts are due to O atoms strongly bonded to a Hf atom or H-bonding and their neighouring C atom that is part of either a disordered or highly vibrating ligand.

PLAT241_ALERT_2_B High 'MainMol' Ueq as Compared to Neighbors of C40 Check

Author Response: These alerts are due to O atoms strongly bonded to a Hf atom or H-bonding and their neighouring C atom that is part of either a disordered or highly vibrating ligand.

```
PLAT241_ALERT_2_B High   'MainMol' Ueq as Compared to Neighbors of       C73 Check
```

Author Response: These alerts are due to O atoms strongly bonded to a Hf atom or H-bonding and their neighouring C atom that is part of either a disordered or highly vibrating ligand.

```
PLAT242_ALERT_2_B Low    'MainMol' Ueq as Compared to Neighbors of       O12 Check
```

Author Response: These alerts are due to O atoms strongly bonded to a Hf atom or H-bonding and their neighouring C atom that is part of either a disordered or highly vibrating ligand.

```
PLAT315_ALERT_2_B Singly Bonded Carbon Detected (H-atoms Missing).       C41 Check
```

Author Response: this atom is part of a ligand in which another C atom could not be located. As I am unsere whether this particular is a CH2 or a CH3 I chose to not show any H atoms

```
PLAT315_ALERT_2_B Singly Bonded Carbon Detected (H-atoms Missing).       C67 Check
```

Author Response: this atom is part of a ligand in which another C atom could not be located. As I am unsere whether this particular is a CH2 or a CH3 I chose to not show any H atoms

```
PLAT342_ALERT_3_B Low Bond Precision on  C-C Bonds ...............    0.04547 Ang.
```

Author Response: not unvexpected given the large amount of disorder in the structure.

```
PLAT413_ALERT_2_B Short Inter XH3 .. XHn     H45C     ..H55C     .       1.92 Ang.
                                                      x,y,z  =      1_555 Check
```

Author Response: H atoms were placed geometrically

```
PLAT910_ALERT_3_B Missing FCF Reflection(s) Below Theta(Min)[Deg]=       4.65 Note
               1  0  0,  -1  1  0,   0  1  0,   0 -1  1,   1 -1  1,  -1  0  1,
               0  0  1,   1  0  1,  -1  1  1,   0  1  1,   0 -1  2,   0  0  2,
```

Author Response: either outliers or blocked by the beam stop

Alert level C

```
PLAT042_ALERT_1_C Calc. and Reported MoietyFormula Strings  Differ     Please Check
              Calc: C57 H101 Hf6 O33, 3(C5 H10 O2), C2 H5
              Rep.: C59 H106 Hf6 O33, 3(C5 H10 O2)
PLAT202_ALERT_3_C Isotropic non-H Atoms in Anion/Solvent .........         15 Check
              C21     C28     C64     C82     O5      C19     C29     C48
              C54     C69     C10     C17     C34     C45     C56
PLAT230_ALERT_2_C Hirshfeld Test Diff for    O12      --C4       .        7.0 s.u.
```

Author Response: These alerts are due to O atoms strongly bonded to a Hf atom or H-bonding and their neighouring C atom that is part of either a disordered or highly vibrating ligand.

```
PLAT230_ALERT_2_C Hirshfeld Test Diff for    O24      --C4       .        6.5 s.u.
```

Author Response: These alerts are due to O atoms strongly bonded to a Hf atom or H-bonding and their neighouring C atom that is part of either a disordered or highly vibrating ligand.

```
PLAT230_ALERT_2_C Hirshfeld Test Diff for    O31      --C26      .        5.2 s.u.
```

Author Response: These alerts are due to O atoms strongly bonded to a Hf atom or H-bonding and their neighouring C atom that is part of either a disordered or highly vibrating ligand.

```
PLAT234_ALERT_4_C Large Hirshfeld Difference Hf1      --O2       .       0.17 Ang.
```

Author Response: These alerts are due to O atoms strongly bonded to a Hf atom or H-bonding and their neighouring C atom that is part of either a disordered or highly vibrating ligand.

```
PLAT234_ALERT_4_C Large Hirshfeld Difference Hf5      --O25      .       0.16 Ang.
```

Author Response: These alerts are due to O atoms strongly bonded to a Hf atom or H-bonding and their neighouring C atom that is part of either a disordered or highly vibrating ligand.

```
PLAT234_ALERT_4_C Large Hirshfeld Difference Hf5      --O32      .       0.19 Ang.
```

Author Response: These alerts are due to O atoms strongly bonded to a Hf atom or H-bonding and their neighouring C atom that is part of either a disordered or highly vibrating ligand.

```
PLAT234_ALERT_4_C Large Hirshfeld Difference Hf6      --O20      .       0.18 Ang.
```

Author Response: These alerts are due to O atoms strongly bonded to a Hf atom or H-bonding and their neighouring C atom that is part of either a disordered or highly vibrating ligand.

```
PLAT234_ALERT_4_C Large Hirshfeld Difference O2       --C22      .       0.20 Ang.
```

Author Response: These alerts are due to O atoms strongly bonded to a Hf atom or H-bonding and their neighouring C atom that is part of either a disordered or highly vibrating ligand.

```
PLAT234_ALERT_4_C Large Hirshfeld Difference O10      --C8       .       0.23 Ang.
```

Author Response: These alerts are due to O atoms strongly bonded to a Hf atom or H-bonding and their neighouring C atom that is part of either a disordered or highly vibrating ligand.

```
PLAT234_ALERT_4_C Large Hirshfeld Difference O13      --C1       .       0.17 Ang.
```

Author Response: These alerts are due to O atoms strongly bonded to a Hf atom or H-bonding and their neighouring C atom that is part of either a disordered or highly vibrating ligand.

```
PLAT234_ALERT_4_C Large Hirshfeld Difference O16      --C12      .       0.20 Ang.
```

Author Response: These alerts are due to O atoms strongly bonded to a Hf atom or H-bonding and their neighouring C atom that is part of either a disordered or highly vibrating ligand.

```
PLAT234_ALERT_4_C Large Hirshfeld Difference O25      --C20      .       0.20 Ang.
```

Author Response: These alerts are due to O atoms strongly bonded to a Hf atom or H-bonding and their neighouring C atom that is part of either a disordered or highly vibrating ligand.

```
PLAT234_ALERT_4_C Large Hirshfeld Difference O29      --C18      .       0.18 Ang.
```

Author Response: These alerts are due to O atoms strongly bonded to a Hf atom or H-bonding and their neighouring C atom that is part of either a disordered or highly vibrating ligand.

PLAT241_ALERT_2_C High 'MainMol' Ueq as Compared to Neighbors of O2 Check

Author Response: These alerts are due to O atoms strongly bonded to a Hf atom or H-bonding and their neighouring C atom that is part of either a disordered or highly vibrating ligand.

PLAT241_ALERT_2_C High 'MainMol' Ueq as Compared to Neighbors of O25 Check

Author Response: These alerts are due to O atoms strongly bonded to a Hf atom or H-bonding and their neighouring C atom that is part of either a disordered or highly vibrating ligand.

PLAT241_ALERT_2_C High 'MainMol' Ueq as Compared to Neighbors of O27 Check

Author Response: These alerts are due to O atoms strongly bonded to a Hf atom or H-bonding and their neighouring C atom that is part of either a disordered or highly vibrating ligand.

PLAT241_ALERT_2_C High 'MainMol' Ueq as Compared to Neighbors of O36 Check

Author Response: These alerts are due to O atoms strongly bonded to a Hf atom or H-bonding and their neighouring C atom that is part of either a disordered or highly vibrating ligand.

PLAT241_ALERT_2_C High 'MainMol' Ueq as Compared to Neighbors of C6 Check

Author Response: These alerts are due to O atoms strongly bonded to a Hf atom or H-bonding and their neighouring C atom that is part of either a disordered or highly vibrating ligand.

PLAT241_ALERT_2_C High 'MainMol' Ueq as Compared to Neighbors of C27 Check

Author Response: These alerts are due to O atoms strongly bonded to a Hf atom or H-bonding and their neighouring C atom that is part of either a disordered or highly vibrating ligand.

PLAT241_ALERT_2_C High 'MainMol' Ueq as Compared to Neighbors of C44 Check

Author Response: These alerts are due to O atoms strongly bonded to a Hf atom or H-bonding and their neighouring C atom that is part of either a disordered or highly vibrating ligand.

PLAT242_ALERT_2_C Low 'MainMol' Ueq as Compared to Neighbors of Hf1 Check

Author Response: These alerts are due to O atoms strongly bonded to a Hf atom or H-bonding and their neighouring C atom that is part of either a disordered or highly vibrating ligand.

PLAT242_ALERT_2_C Low 'MainMol' Ueq as Compared to Neighbors of Hf5 Check

Author Response: These alerts are due to O atoms strongly bonded to a Hf atom or H-bonding and their neighouring C atom that is part of either a disordered or highly vibrating ligand.

PLAT242_ALERT_2_C Low 'MainMol' Ueq as Compared to Neighbors of Hf6 Check

Author Response: These alerts are due to O atoms strongly bonded to a Hf atom or H-bonding and their neighouring C atom that is part of either a disordered or highly vibrating ligand.

PLAT242_ALERT_2_C Low 'MainMol' Ueq as Compared to Neighbors of O7 Check

Author Response: These alerts are due to O atoms strongly bonded to a Hf atom or H-bonding and their neighouring C atom that is part of either a disordered or highly vibrating ligand.

PLAT242_ALERT_2_C Low 'MainMol' Ueq as Compared to Neighbors of O10 Check

Author Response: These alerts are due to O atoms strongly bonded to a Hf atom or H-bonding and their neighouring C atom that is part of either a disordered or highly vibrating ligand.

PLAT242_ALERT_2_C Low 'MainMol' Ueq as Compared to Neighbors of O13 Check

Author Response: These alerts are due to O atoms strongly bonded to a Hf atom or H-bonding and their neighouring C atom that is part of either a disordered or highly vibrating ligand.

PLAT242_ALERT_2_C Low 'MainMol' Ueq as Compared to Neighbors of O20 Check

Author Response: These alerts are due to O atoms strongly bonded to a Hf atom or H-bonding and their neighouring C atom that is part of either a disordered or highly vibrating ligand.

PLAT242_ALERT_2_C Low 'MainMol' Ueq as Compared to Neighbors of O24 Check

Author Response: These alerts are due to O atoms strongly bonded to a Hf atom or H-bonding and their neighouring C atom that is part of either a disordered or highly vibrating ligand.

PLAT242_ALERT_2_C Low 'MainMol' Ueq as Compared to Neighbors of O28 Check

Author Response: These alerts are due to O atoms strongly bonded to a Hf atom or H-bonding and their neighouring C atom that is part of either a disordered or highly vibrating ligand.

PLAT242_ALERT_2_C Low 'MainMol' Ueq as Compared to Neighbors of O31 Check

Author Response: These alerts are due to O atoms strongly bonded to a Hf atom or H-bonding and their neighouring C atom that is part of either a disordered or highly vibrating ligand.

PLAT242_ALERT_2_C Low 'MainMol' Ueq as Compared to Neighbors of O32 Check

Author Response: These alerts are due to O atoms strongly bonded to a Hf atom or H-bonding and their neighouring C atom that is part of either a disordered or highly vibrating ligand.

PLAT242_ALERT_2_C Low 'MainMol' Ueq as Compared to Neighbors of O41 Check

Author Response: These alerts are due to O atoms strongly bonded to a Hf atom or H-bonding and their neighouring C atom that is part of either a disordered or highly vibrating ligand.

PLAT242_ALERT_2_C Low 'MainMol' Ueq as Compared to Neighbors of C12 Check

Author Response: These alerts are due to O atoms strongly bonded to a Hf atom or H-bonding and their neighouring C atom that is part of either a disordered or highly vibrating ligand.

PLAT242_ALERT_2_C Low 'MainMol' Ueq as Compared to Neighbors of C14 Check

Author Response: These alerts are due to O atoms strongly bonded to a Hf atom or H-bonding and their neighouring C atom that is part of either a disordered or highly vibrating ligand.

```
PLAT242_ALERT_2_C Low    'MainMol' Ueq as Compared to Neighbors of       C18 Check
```

Author Response: These alerts are due to O atoms strongly bonded to a Hf atom or H-bonding and their neighouring C atom that is part of either a disordered or highly vibrating ligand.

```
PLAT242_ALERT_2_C Low    'MainMol' Ueq as Compared to Neighbors of       C20 Check
```

Author Response: These alerts are due to O atoms strongly bonded to a Hf atom or H-bonding and their neighouring C atom that is part of either a disordered or highly vibrating ligand.

```
PLAT242_ALERT_2_C Low    'MainMol' Ueq as Compared to Neighbors of       C22 Check
```

Author Response: These alerts are due to O atoms strongly bonded to a Hf atom or H-bonding and their neighouring C atom that is part of either a disordered or highly vibrating ligand.

```
PLAT242_ALERT_2_C Low    'MainMol' Ueq as Compared to Neighbors of       C32 Check
```

Author Response: These alerts are due to O atoms strongly bonded to a Hf atom or H-bonding and their neighouring C atom that is part of either a disordered or highly vibrating ligand.

```
PLAT243_ALERT_4_C High   'Solvent' Ueq as Compared to Neighbors of       C64 Check
PLAT243_ALERT_4_C High   'Solvent' Ueq as Compared to Neighbors of       C48 Check
PLAT243_ALERT_4_C High   'Solvent' Ueq as Compared to Neighbors of       C10 Check
PLAT244_ALERT_4_C Low    'Solvent' Ueq as Compared to Neighbors of       C21 Check
PLAT260_ALERT_2_C Large Average Ueq of Residue Including      Hf1      0.158 Check
PLAT260_ALERT_2_C Large Average Ueq of Residue Including       O9      0.176 Check
PLAT260_ALERT_2_C Large Average Ueq of Residue Including       O5      0.169 Check
PLAT260_ALERT_2_C Large Average Ueq of Residue Including      O33      0.190 Check
PLAT260_ALERT_2_C Large Average Ueq of Residue Including      C47      0.247 Check
PLAT329_ALERT_4_C Carbon Atom Hybridisation Unclear for .........       C47 Check
PLAT360_ALERT_2_C Short  C(sp3)-C(sp3) Bond  C7       - C84      .       1.41 Ang.
PLAT360_ALERT_2_C Short  C(sp3)-C(sp3) Bond  C13      - C87      .       1.43 Ang.
PLAT361_ALERT_2_C Long   C(sp3)-C(sp3) Bond  C55      - C73      .       1.74 Ang.
PLAT363_ALERT_2_C Long   C(sp3)-C(sp2) Bond  C22      - C40      .       1.65 Ang.
PLAT410_ALERT_2_C Short Intra H...H Contact  H42      ..H72A     .       1.95 Ang.
                                                      x,y,z  =      1_555 Check
PLAT412_ALERT_2_C Short Intra XH3 .. XHn     H70B     ..H80A     .       1.80 Ang.
                                                      x,y,z  =      1_555 Check
PLAT413_ALERT_2_C Short Inter XH3 .. XHn     H13B     ..H17B     .       2.07 Ang.
                                                1-x,-y,1-z  =      2_656 Check
```

Author Response: H atoms were placed geometrically

```
PLAT413_ALERT_2_C Short Inter XH3 .. XHn     H15C     ..H47B     .       2.06 Ang.
                                              x,-1+y,z  =      1_545 Check
```

Author Response: H atoms were placed geometrically

```
PLAT413_ALERT_2_C Short Inter XH3 .. XHn     H88A     ..H88A     .       2.09 Ang.
                                            1-x,-y,2-z  =      2_657 Check
```

Author Response: H atoms were placed geometrically

```
PLAT413_ALERT_2_C Short Inter XH3 .. XHn     H34      ..H55B     .       2.13 Ang.
                                            1-x,-y,1-z  =      2_656 Check
```

Author Response: H atoms were placed geometrically

```
PLAT601_ALERT_2_C Unit-Cell Contains Solvent Accessible VOIDS .LE.         34 Ang**3
PLAT906_ALERT_3_C Large K Value in the Analysis of Variance ......      8.071 Check
PLAT911_ALERT_3_C Missing FCF Refl Between Thmin & STh/L=    0.600        197 Report
                2  0  0,  -2  1  0,   1  1  0,   0  2  0,  -1  3  0,   0  3  0,
                9  7  0,   1 -3  1,  -1 -1  1,   2 -1  1,   2  0  1,  -2  1  1,
                1  1  1,   0  2  1,   1  2  1,  -2  3  1,  -1  3  1,   0  3  1,
                1  3  1,  -4  4  1,   9  7  1,  -1 -1  2,   2 -1  2,   1  0  2,
               -1  1  2,   0  1  2,   1  1  2,   0  2  2,   0  3  2,   0 -1  3,
                   (  167 More Missing: see the .ckf listing file)
PLAT913_ALERT_3_C Missing # of Very Strong Reflections in FCF ....          6 Note
               -1  1  0,   0 -1  1,  -1  0  1,   1  0  1,   0  1  1,   0  0  2,
PLAT971_ALERT_2_C Check Calcd Resid. Dens.  0.92Ang From Hf6            1.81 eA-3
PLAT971_ALERT_2_C Check Calcd Resid. Dens.  0.98Ang From Hf3            1.69 eA-3
PLAT973_ALERT_2_C Check Calcd Positive Resid. Density on       Hf6      1.16 eA-3
PLAT973_ALERT_2_C Check Calcd Positive Resid. Density on       Hf3      1.09 eA-3
PLAT973_ALERT_2_C Check Calcd Positive Resid. Density on       Hf4      1.06 eA-3
PLAT973_ALERT_2_C Check Calcd Positive Resid. Density on       Hf1      1.05 eA-3
PLAT977_ALERT_2_C Check Negative Difference Density on H3B       .     -0.31 eA-3
PLAT977_ALERT_2_C Check Negative Difference Density on H5B       .     -0.38 eA-3
PLAT977_ALERT_2_C Check Negative Difference Density on H29A      .     -0.51 eA-3
PLAT977_ALERT_2_C Check Negative Difference Density on H37A      .     -0.50 eA-3
PLAT977_ALERT_2_C Check Negative Difference Density on H37C      .     -0.35 eA-3
PLAT977_ALERT_2_C Check Negative Difference Density on H47B      .     -0.48 eA-3
PLAT977_ALERT_2_C Check Negative Difference Density on H55B      .     -0.36 eA-3
PLAT977_ALERT_2_C Check Negative Difference Density on H80A      .     -0.32 eA-3
PLAT977_ALERT_2_C Check Negative Difference Density on H83B      .     -0.43 eA-3
PLAT977_ALERT_2_C Check Negative Difference Density on H83D      .     -0.36 eA-3
PLAT977_ALERT_2_C Check Negative Difference Density on H88A      .     -0.31 eA-3
PLAT977_ALERT_2_C Check Negative Difference Density on H88B      .     -0.43 eA-3
```

Alert level G

```
PLAT002_ALERT_2_G Number of Distance or Angle Restraints on AtSite         83 Note
PLAT003_ALERT_2_G Number of Uiso or U(i,j)  Restrained non-H-Atoms         55 Report
PLAT007_ALERT_5_G Number of Unrefined Donor-H Atoms .............           9 Report
              H4      H8      H11     H26     H34A    H34B    H17     H5
              H37
PLAT083_ALERT_2_G SHELXL Second Parameter in WGHT  Unusually Large      27.00 Why ?
```

```
PLAT154_ALERT_1_G The s.u.'s on the Cell Angles are Equal ..(Note)      0.003 Degree
PLAT171_ALERT_4_G The CIF-Embedded .res File Contains EADP Records         22 Report
PLAT172_ALERT_4_G The CIF-Embedded .res File Contains DFIX Records         48 Report
PLAT173_ALERT_4_G The CIF-Embedded .res File Contains DANG Records         61 Report
PLAT176_ALERT_4_G The CIF-Embedded .res File Contains SADI Records         12 Report
PLAT178_ALERT_4_G The CIF-Embedded .res File Contains SIMU Records          4 Report
PLAT186_ALERT_4_G The CIF-Embedded .res File Contains ISOR Records          4 Report
PLAT187_ALERT_4_G The CIF-Embedded .res File Contains RIGU Records          4 Report
PLAT191_ALERT_3_G A Non-default SADI Restraint Value has been used     0.0400 Report
PLAT299_ALERT_4_G Atom Site Occupancy Constrained at ............         0.5 Check
              C43     C51     C57     C76     C77     C79     H30     H30A
              H43A    H43B    H43C    H50     H50A    H51A    H51B    H51C
              H57A    H57B    H76A    H76B    H76C    H77A    H77B    H77C
              H79A    H79B    H83A    H83B    H83C    H83D    H83E    H83F
              C59     C81     H59A    H59B    H59C    H64     H64A    H81A
              H81B    H81C
PLAT301_ALERT_3_G Main Residue  Disorder .............(Resd    1)          3% Note
PLAT302_ALERT_4_G Anion/Solvent/Minor-Residue Disorder (Resd    2)         14% Note
PLAT309_ALERT_2_G Single Bonded Oxygen (C-O > 1.3 Ang) ..........         O39 Check
PLAT343_ALERT_2_G Unusual sp3      Angle Range in Main Residue for         C2 Check
PLAT343_ALERT_2_G Unusual sp3      Angle Range in Main Residue for        C27 Check
PLAT343_ALERT_2_G Unusual sp?      Angle Range in Main Residue for        C41 Check
PLAT343_ALERT_2_G Unusual sp3      Angle Range in Main Residue for        C42 Check
PLAT343_ALERT_2_G Unusual sp3      Angle Range in Main Residue for        C46 Check
PLAT343_ALERT_2_G Unusual sp?      Angle Range in Main Residue for        C52 Check
PLAT343_ALERT_2_G Unusual sp3      Angle Range in Main Residue for        C63 Check
PLAT343_ALERT_2_G Unusual sp?      Angle Range in Main Residue for        C67 Check
PLAT343_ALERT_2_G Unusual sp3      Angle Range in Main Residue for        C72 Check
PLAT344_ALERT_2_G Unusual sp3       Angle Range in Solvent/Ion for        C54 Check
PLAT344_ALERT_2_G Unusual           Angle Range in Solvent/Ion for        C47 Check
PLAT344_ALERT_2_G Unusual sp3       Angle Range in Solvent/Ion for       C128 Check
PLAT367_ALERT_2_G Long?  C(sp?)-C(sp?) Bond  C16      - C52      .       1.54 Ang.
PLAT367_ALERT_2_G Long?  C(sp?)-C(sp?) Bond  C27      - C67      .       1.52 Ang.
PLAT412_ALERT_2_G Short Intra XH3 .. XHn     H11A     ..H43A     .       2.13 Ang.
                                                      x,y,z  =      1_555 Check
PLAT412_ALERT_2_G Short Intra XH3 .. XHn     H51C     ..H88C     .       2.10 Ang.
                                                      x,y,z  =      1_555 Check
PLAT412_ALERT_2_G Short Intra XH3 .. XHn     H76C     ..H88B     .       1.87 Ang.
                                                      x,y,z  =      1_555 Check
PLAT412_ALERT_2_G Short Intra XH3 .. XHn     H21B     ..H59C     .       1.98 Ang.
                                                      x,y,z  =      1_555 Check
PLAT412_ALERT_2_G Short Intra XH3 .. XHn     H64A     ..H82A     .       2.11 Ang.
                                                      x,y,z  =      1_555 Check
PLAT413_ALERT_2_G Short Inter XH3 .. XHn     H51A     ..H82C     .       2.06 Ang.
                                                      x,y,z  =      1_555 Check
```

Author Response: H atoms were placed geometrically

```
PLAT413_ALERT_2_G Short Inter XH3 .. XHn     H74C     ..H76A     .       2.09 Ang.
                                                1-x,1-y,2-z  =      2_667 Check
```

Author Response: H atoms were placed geometrically

```
PLAT413_ALERT_2_G Short Inter XH3 .. XHn     H59C     ..H74C     .       1.68 Ang.
                                              -1+x,-1+y,z  =     1_445 Check
```

Author Response: H atoms were placed geometrically

```
PLAT413_ALERT_2_G Short Inter XH3 .. XHn     H47A     ..H51B     .       2.05 Ang.
                                                  x,1+y,z  =     1_565 Check
```

Author Response: H atoms were placed geometrically

```
PLAT432_ALERT_2_G Short Inter X...Y Contact  O32      ..C128     .       3.00 Ang.
                                                    x,y,z  =     1_555 Check
PLAT432_ALERT_2_G Short Inter X...Y Contact  C11      ..C47      .       2.48 Ang.
                                                    x,y,z  =     1_555 Check
PLAT432_ALERT_2_G Short Inter X...Y Contact  C11      ..C128     .       3.13 Ang.
                                                    x,y,z  =     1_555 Check
PLAT432_ALERT_2_G Short Inter X...Y Contact  C16      ..C128     .       3.01 Ang.
                                                    x,y,z  =     1_555 Check
PLAT432_ALERT_2_G Short Inter X...Y Contact  C16      ..C47      .       3.11 Ang.
                                                    x,y,z  =     1_555 Check
PLAT432_ALERT_2_G Short Inter X...Y Contact  C47      ..C52      .       1.85 Ang.
                                                    x,y,z  =     1_555 Check
PLAT432_ALERT_2_G Short Inter X...Y Contact  C52      ..C128     .       2.44 Ang.
                                                    x,y,z  =     1_555 Check
PLAT721_ALERT_1_G Bond    Calc     0.97000, Rep     0.98010 Dev...       0.01 Ang.
             C74      -H74B           1_555   1_555 .......     #  180 Check
PLAT721_ALERT_1_G Bond    Calc     0.98000, Rep     0.99010 Dev...       0.01 Ang.
             C78      -H78A           1_555   1_555 .......     #  182 Check
PLAT721_ALERT_1_G Bond    Calc     0.99000, Rep     0.97990 Dev...       0.01 Ang.
             C81      -H81B           1_555   1_555 .......     #  269 Check
PLAT722_ALERT_1_G Angle   Calc      112.00, Rep      110.60 Dev...       1.40 Degree
             C34     -C56     -H56A       1_555  1_555  1_555     #  592 Check
PLAT722_ALERT_1_G Angle   Calc      108.00, Rep      109.50 Dev...       1.50 Degree
             H43A    -C43     -H43B       1_555  1_555  1_555     #  672 Check
PLAT722_ALERT_1_G Angle   Calc      107.00, Rep      108.20 Dev...       1.20 Degree
             C34     -C45     -H45A       1_555  1_555  1_555     #  681 Check
PLAT722_ALERT_1_G Angle   Calc      115.00, Rep      113.80 Dev...       1.20 Degree
             C88     -C25     -H25B       1_555  1_555  1_555     #  710 Check
PLAT722_ALERT_1_G Angle   Calc      115.00, Rep      113.60 Dev...       1.40 Degree
             C25     -C88     -H88B       1_555  1_555  1_555     #  881 Check
PLAT764_ALERT_4_G Overcomplete CIF Bond List Detected (Rep/Expd) .       1.11 Ratio
PLAT773_ALERT_2_G Check long C-C Bond in CIF: C52      --C47             1.85 Ang.
PLAT773_ALERT_2_G Check long C-C Bond in CIF: C55      --C73             1.73 Ang.
PLAT793_ALERT_4_G Model has Chirality at C2         (Centro SpGr)           S Verify
PLAT793_ALERT_4_G Model has Chirality at C24        (Centro SpGr)           S Verify
PLAT793_ALERT_4_G Model has Chirality at C34        (Centro SpGr)           R Verify
PLAT793_ALERT_4_G Model has Chirality at C38        (Centro SpGr)           R Verify
PLAT793_ALERT_4_G Model has Chirality at C40        (Centro SpGr)           S Verify
PLAT793_ALERT_4_G Model has Chirality at C42        (Centro SpGr)           S Verify
PLAT793_ALERT_4_G Model has Chirality at C44        (Centro SpGr)           S Verify
PLAT793_ALERT_4_G Model has Chirality at C62        (Centro SpGr)           R Verify
PLAT793_ALERT_4_G Model has Chirality at C69        (Centro SpGr)           R Verify
PLAT793_ALERT_4_G Model has Chirality at C73        (Centro SpGr)           R Verify
PLAT794_ALERT_5_G Tentative Bond Valency for Hf1       (IV)      .       4.18 Info
```

```
PLAT794_ALERT_5_G Tentative Bond Valency for Hf2       (IV)      .       4.19 Info
PLAT794_ALERT_5_G Tentative Bond Valency for Hf3       (IV)      .       4.09 Info
PLAT794_ALERT_5_G Tentative Bond Valency for Hf4       (IV)      .       4.33 Info
PLAT794_ALERT_5_G Tentative Bond Valency for Hf5       (IV)      .       3.98 Info
PLAT794_ALERT_5_G Tentative Bond Valency for Hf6       (IV)      .       4.18 Info
PLAT860_ALERT_3_G Number of Least-Squares Restraints ............         364 Note
PLAT883_ALERT_1_G Absent Datum for _atom_sites_solution_primary ..     Please Do !
PLAT912_ALERT_4_G Missing # of FCF Reflections Above STh/L=  0.600        345 Note
PLAT933_ALERT_2_G Number of HKL-OMIT Records in Embedded .res File          7 Note
               -2  3  1,  -1 -1  1,  -1 -1  2,   0 -1  3,   1 -1  5,   1  1  0,
                1  2  3,
PLAT941_ALERT_3_G Average HKL Measurement Multiplicity ..........         4.1 Low
PLAT969_ALERT_5_G The 'Henn et al.' R-Factor-gap value ..........       5.117 Note
              Predicted wR2: Based on SigI**2  4.93 or SHELX Weight 23.97
PLAT978_ALERT_2_G Number C-C Bonds with Positive Residual Density.          0 Info
```

```
   1 ALERT level A = Most likely a serious problem - resolve or explain
  18 ALERT level B = A potentially serious problem, consider carefully
  82 ALERT level C = Check. Ensure it is not caused by an omission or oversight
  81 ALERT level G = General information/check it is not something unexpected

  11 ALERT type 1 CIF construction/syntax error, inconsistent or missing data
 115 ALERT type 2 Indicator that the structure model may be wrong or deficient
  10 ALERT type 3 Indicator that the structure quality may be low
  38 ALERT type 4 Improvement, methodology, query or suggestion
   8 ALERT type 5 Informative message, check
```

It is advisable to attempt to resolve as many as possible of the alerts in all categories. Often the minor alerts point to easily fixed oversights, errors and omissions in your CIF or refinement strategy, so attention to these fine details can be worthwhile. It is up to the individual to critically assess their own results and, if necessary, seek expert advice.

PLATON version of 26/09/2025; check.def file version of 20/09/2025

Datablock mjp169_150k_new - ellipsoid plot

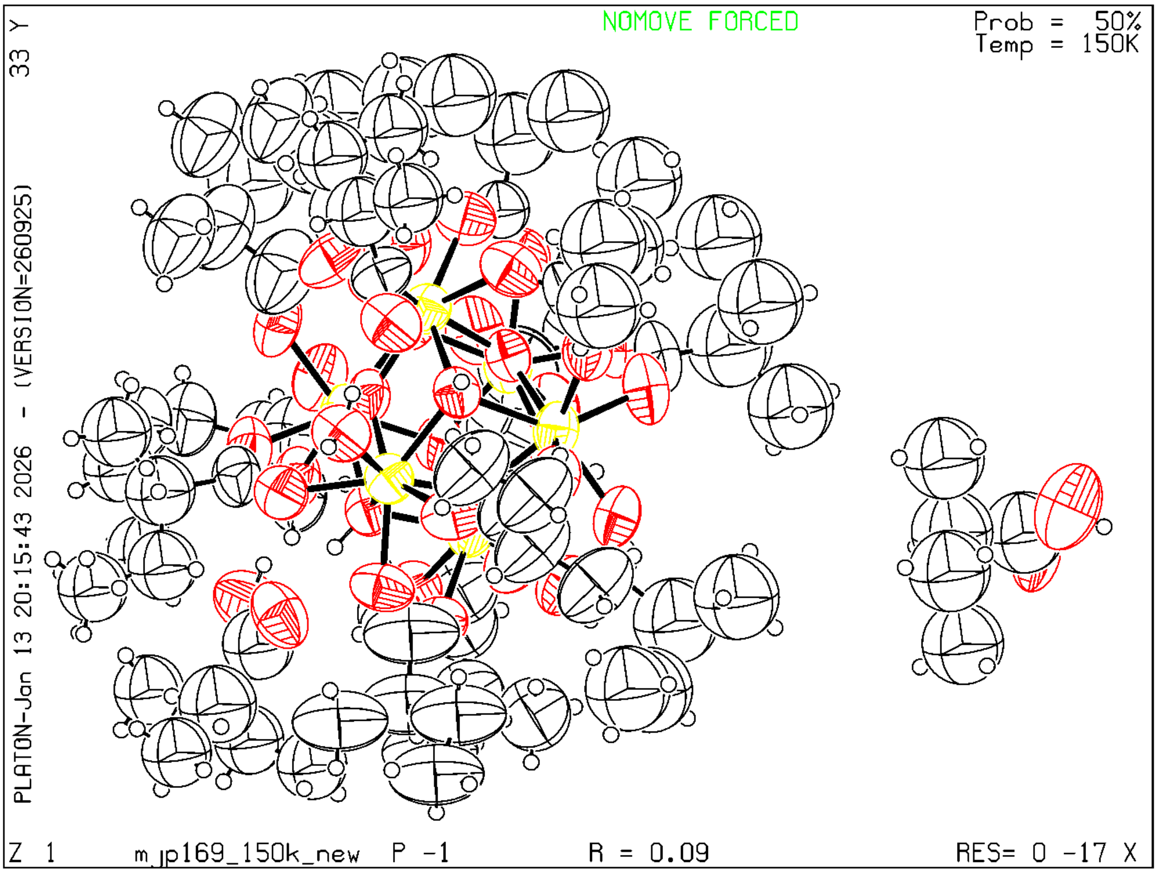

Supplement: Supplementary file 2 — Supporting File 2: anie71298–sup–0002–Data.zip. [file ANIE-65-e25769-s002.zip › CCDC_2453143/mjp169_150k_new_cifreport.pdf]
