# Supplementary material for: Mechanism‐Guided Precision Hydrolysis of Early Transition Metals to Access (Mixed‐Metal) Oxo Clusters
Source: Angew Chem Int Ed Engl. 2026 Feb 24;65(15):e25769. doi: 10.1002/anie.202525769 (PMC13053926; doi:10.1002/anie.202525769)
Supplement: Supplementary file 2 — Supporting File 2: anie71298–sup–0002–Data.zip. [file ANIE-65-e25769-s002.zip › CCDC_2495248/mjp184_150k_new.rtf]

;;;;;;;fLine0fBehindDocument1shapeType1pibmjp184_150k_new

	
		
	
	
R1=10.64%


Crystal Data and Experimental


Experimental. Single colourless block-shaped crystals of mjp184_150k_new were used as supplied. A suitable crystal with dimensions 0.30 × 0.23 × 0.20 mm was selected and mounted on a STOE STADIVARI Cu diffractometer. The crystal was kept at a steady T = 150 K during data collection. The structure was solved with the ShelXT 2018/2 (Sheldrick, 2018) solution program using iterative methods and by using Olex2 1.5 (Dolomanov et al., 2009) as the graphical interface. The model was refined with ShelXL 2019/3 (Sheldrick, 2015) using full matrix least squares minimisation on |F|2.
Crystal Data. C74H136O39Zr6, Mr = 2197.14, triclinic, P-1 (No. 2), a = 14.2648(5) Å, b = 15.8095(5) Å, c = 24.2737(7) Å, a = 94.600(2)°, b = 96.227(3)°, g = 109.628(2)°, V = 5085.8(3) Å3, T = 150 K, Z = 2, Z' = 1, m(Cu Ka) = 5.521, 78162 reflections measured, 20361 unique (Rint = 0.0918) which were used in all calculations. The final wR2 was 0.2863 (all data) and R1 was 0.1064 (I≥2s(I)).
Compound 	mjp184_150k_new 	
 	 	
Formula 	C74H136O39Zr6 	
Dcalc./ g cm-3 	1.435 	
m/mm-1 	5.521 	
Formula Weight 	2197.14 	
Colour 	colourless 	
Shape 	block-shaped 	
Size/mm 	0.30×0.23×0.20 	
T/K 	150 	
Crystal System 	triclinic 	
Space Group 	P-1 	
a/Å 	14.2648(5) 	
b/Å 	15.8095(5) 	
c/Å 	24.2737(7) 	
a/° 	94.600(2) 	
b/° 	96.227(3) 	
g/° 	109.628(2) 	
V/Å3 	5085.8(3) 	
Z 	2 	
Z' 	1 	
Wavelength/Å 	1.54186 	
Radiation type 	Cu Ka 	
Qmin/° 	5.036 	
Qmax/° 	75.230 	
Index range h 	-9 ≥ h ≥ 17 	
Index range k 	-19 ≥ k ≥ 18 	
Index range l 	-30 ≥ l ≥ 27 	
Measured Refl's. 	78162 	
Indep't Refl's 	20361 	
Refl's I≥2s(I) 	10458 	
Rint 	0.0918 	
Parameters 	775 	
Restraints 	998 	
Largest Peak/eÅ3 	1.278 	
Deepest Hole/eÅ3 	-0.890 	
GooF 	1.021 	
R1 (I≥2s(I) / all) 	0.1064 / 0.1594 	
wR2 (I≥2s(I) / all) 	0.2498 / 0.2863 	

Structure Quality Indicators
Reflections:		
Refinement:		
A colourless block-shaped crystal with dimensions 0.30 × 0.23 × 0.20 mm was mounted. Data were collected using a STOE STADIVARI Cu diffractometer equipped with an Oxford Cryosystems low-temperature device operating at T = 150 K.
Data were measured using rotation method, w scans with Cu Ka radiation. The diffraction pattern was indexed and the total number of runs and images was based on the strategy calculation from the program X-Area Pilatus3_SV 1.31.170.0 (STOE, 2020) . The maximum resolution achieved was Q = 75.230° (0.80 Å).
The unit cell was refined using X-Area Pilatus3_SV 1.31.170.0 (STOE, 2020) on 38701 reflections, 50% of the observed reflections.
Data reduction, scaling and absorption corrections were performed using X-Area Pilatus3_SV 1.31.170.0 (STOE, 2020). The final completeness is 98.50 % out to 75.230° in Q. A multi-scan absorption correction was performed using STOE. The absorption coefficient m of this material is 5.521 mm-1 at this wavelength (l = 1.54186Å) and the minimum and maximum transmissions are 0.007 and 0.041.
The structure was solved in the space group P-1 (# 2) by ShelXT 2018/2 (Sheldrick, 2018) using iterative methods. It was refined by full matrix least squares minimisation on |F|2 using version 2019/3 of ShelXL 2019/3 (Sheldrick, 2015). All non-hydrogen atoms were refined anisotropically.
Hydrogen atom positions were calculated geometrically and refined using the riding model.
_refine_special_details: There is a lot of disorder and the asymmetric shape of the carboxylates does not help:A lot of DFIX, D&nbsp;&Aring;, EADP, SIMU and RIGU had to be used to refine those ligands.2 ligands had to be refined isotropically 1 disordered ligand is missing the extra terminal C that could not be located in the map
_exptl_absorpt_process_details: STOE X-Red32, absorption correction by Gaussian integration, analogous toP. Coppens in: F. R. Ahmed (Editor), "Crystallographic Computing", Munksgaard,Copenhagen (1970), 255 - 270. Afterwards scaling of reflection intensities wasperformed within STOE LANA. J. Koziskova, F. Hahn, J. Richter, J. Kozisek, ActaChimica Slovaca, vol. 9, no. 2, 2016, pp. 136 - 140.Finally a spherical absorption correction was done within STOE LANA.
There is a single formula unit in the asymmetric unit, which is represented by the reported sum formula. In other words: Z is 2 and Z' is 1. The moiety formula is C59 H106 O33 Zr6, 3(C5 H10 O2).
Data Plots: Diffraction Data
 	 	
 	 	
Data Plots: Refinement and Data
 	 	
Reflection Statistics

Total reflections (after filtering) 	78162 	Unique reflections 	20361 	
Completeness 	0.97 	Mean I/s 	8.93 	
hklmax collected 	(17, 18, 27) 	hklmin collected 	(-9, -19, -30) 	
hklmax used 	(17, 19, 30) 	hklmin used 	(-17, -19, 0) 	
Lim dmax collected 	100.0 	Lim dmin collected 	0.77 	
dmax used 	8.78 	dmin used 	0.8 	
Friedel pairs 	3341 	Friedel pairs merged 	1 	
Inconsistent equivalents 	479 	Rint 	0.0918 	
Rsigma 	0.0642 	Intensity transformed 	0 	
Omitted reflections 	0 	Omitted by user (OMIT hkl) 	15 	
Multiplicity 	(4728, 6336, 4113, 2979, 1909, 1316, 943, 636, 386, 208, 96, 43, 15, 4, 1) 	Maximum multiplicity 	15 	
Removed systematic absences 	0 	Filtered off (Shel/OMIT) 	0 	


Table 0: Fractional Atomic Coordinates (×104) and Equivalent Isotropic Displacement Parameters (Å2×103) for mjp184_150k_new. Ueq is defined as 1/3 of the trace of the orthogonalised Uij.

Atom	x	y	z	Ueq	
Zr1	6626.7(7)	6967.7(7)	1253.3(4)	94.7(3)	
Zr2	7546.8(7)	8480.4(7)	2468.7(4)	96.8(3)	
Zr3	8453.9(7)	6695.1(7)	2217.8(4)	99.0(3)	
Zr4	6950.5(8)	6677.7(8)	3273.9(4)	102.2(3)	
Zr5	6017.9(8)	5124.2(7)	2068.8(4)	98.2(3)	
Zr6	5103.5(7)	6930.3(8)	2311.6(4)	101.4(3)	
O1	7974(5)	7456(5)	2798(3)	94.4(19)	
O2	5255(7)	7201(6)	881(3)	121(3)	
O3	5315(5)	5950(5)	1568(3)	96.4(19)	
O4	6483(5)	7776(5)	3020(3)	104(2)	
O5	8585(7)	5361(8)	2024(4)	132(3)	
O6	7120(7)	8306(7)	992(3)	121(3)	
O7	4255(6)	7088(6)	1548(4)	124(3)	
O8	8140(5)	7785(5)	1842(3)	99(2)	
O9	5405(7)	4214(6)	1322(4)	116(2)	
O10	9666(8)	6678(8)	2889(4)	137(3)	
O11	7063(9)	4382(6)	1956(4)	134(3)	
O12	6366(7)	5307(8)	3539(4)	132(3)	
O13	5851(5)	6128(5)	2611(3)	92.7(18)	
O14	3773(6)	5699(8)	2186(4)	129(3)	
O15	7489(5)	5810(5)	2743(3)	100(2)	
O16	5949(6)	5886(6)	537(3)	113(2)	
O17	6345(5)	7613(5)	1949(3)	96(2)	
O18	7649(7)	9286(6)	1769(4)	119(3)	
O19	7083(5)	6129(5)	1728(3)	95.7(19)	
O20	4982(7)	8255(7)	2468(4)	128(3)	
O21	9162(7)	9425(6)	2606(4)	127(3)	
O22	9018(6)	6716(6)	1412(4)	123(3)	
O23	8238(7)	6633(8)	3891(4)	136(3)	
O24	4332(7)	4545(6)	2093(4)	126(3)	
O25	7830(7)	6911(6)	789(3)	117(3)	
O26	8272(7)	9414(6)	3272(3)	120(3)	
O27	10013(6)	7750(6)	2395(4)	122(3)	
O28	6540(8)	9280(6)	2564(4)	127(3)	
O29	5810(7)	4331(6)	2752(4)	126(3)	
O30	4478(7)	4314(7)	628(4)	148(4)	
O31	7711(8)	7726(7)	4032(4)	137(3)	
O32	5677(8)	6649(7)	3726(3)	133(3)	
O33	4526(7)	6874(8)	3122(4)	135(3)	
C1	4730(20)	3828(19)	938(14)	247(6)	
C2	4441(16)	7232(16)	1038(10)	183(4)	
C3	8010(30)	4570(20)	1925(15)	356(9)	
C4	7500(30)	9140(20)	1217(14)	256(6)	
O34	6195(9)	8968(9)	3889(5)	164(4)	
O35	-338(10)	8979(9)	1374(5)	164(4)	
C5	9070(20)	9723(19)	3077(15)	256(6)	
C6	3625(18)	4850(20)	2136(14)	238(5)	
C7	5980(30)	4480(20)	3292(16)	281(7)	
C8	8631(16)	6811(12)	912(8)	151(6)	
O36	8275(11)	4675(9)	3305(6)	175(4)	
C9	6760(30)	9350(20)	4319(12)	324(8)	
C10	8290(20)	7350(20)	4192(10)	244(6)	
C11	5550(20)	9054(19)	2540(12)	208(5)	
O37	9260(20)	5551(18)	4035(12)	393(19)	
C12	7503(19)	9819(13)	859(10)	256(6)	
C13	4822(14)	6736(14)	3595(7)	147(5)	
O39	997(11)	9169(12)	1884(9)	255(9)	
C14	4192(19)	2918(16)	844(11)	247(6)	
C15	470(30)	9450(30)	1525(19)	334(9)	
C17	5215(16)	9799(16)	2653(11)	208(5)	
C18	9930(17)	10559(16)	3421(11)	256(6)	
C19	8310(20)	3803(16)	2045(9)	356(9)	
C20	2592(17)	4220(20)	1991(10)	238(5)	
C21	11416(14)	7704(17)	3009(9)	210(4)	
C22	8940(30)	4780(20)	3715(18)	270	
C23	4752(19)	2315(16)	975(10)	247(6)	
C24	1220(20)	10220(20)	1309(14)	334(9)	
C25	9058(18)	7600(20)	4704(8)	244(6)	
C27	3666(13)	7382(13)	665(8)	183(4)	
C29	4103(17)	6620(20)	4030(9)	212(9)	
C30	1810(30)	4170(70)	1510(20)	238(5)	
C31	5735(19)	3752(14)	3623(11)	281(7)	
C32	11496(13)	7731(18)	3624(9)	210(4)	
C33	11914(13)	7103(16)	2762(7)	210(4)	
C34	9356.94	6710.15	379.71	280	
C36	3184(17)	2508(15)	501(11)	247(6)	
C37	10306(16)	7310(20)	2773(11)	210(4)	
C38	7697(19)	2829(17)	2061(13)	356(9)	
C39	9770(20)	5996(15)	549(13)	280	
C28	3080(30)	5910(40)	3890(30)	260(20)	
C41	6000(20)	10726(18)	2740(20)	208(5)	
C42	9109(16)	3818(19)	1691(11)	356(9)	
C43	2310(40)	3352(17)	2241(13)	238(5)	
C44	3590(30)	8260(20)	907(19)	183(4)	
C45	8528(18)	7613(17)	5198(8)	244(6)	
C46	10922(17)	10426(16)	3411(12)	256(6)	
C47	6493(19)	9828(16)	605(11)	256(6)	
C48	10098(17)	11383(16)	3127(11)	256(6)	
C49	9750(19)	8605(19)	4711(8)	244(6)	
C51	4104(17)	9533(18)	2626(16)	208(5)	
C52	5922(19)	2945(19)	3353(12)	281(7)	
C53	3160(30)	6840(15)	112(10)	183(4)	
C54	9350(20)	7513(15)	102(13)	280	
C56	1320(50)	11100(30)	1630(20)	334(9)	
C57	3820(40)	7450(30)	4088(19)	239(15)	
C59	1800(30)	9690(30)	1050(20)	334(9)	
C60	850(50)	10230(50)	709(18)	334(9)	
C61	890(50)	10990(40)	1180(30)	334(9)	
C62	4623(19)	3335(16)	3627(13)	281(7)	
C63	3050(20)	7933(18)	851(18)	183(4)	
C64	2840(20)	6477(13)	509(14)	183(4)	
C66	5390(30)	10390(30)	2191(15)	208(5)	
C65	4450(30)	9680(30)	3046(16)	208(5)	
C67	4660(30)	7020(50)	4605(12)	280(20)	
C68	3690(40)	5630(20)	4090(30)	260(20)	
C69	2350(30)	4550(20)	2534(14)	238(5)	
C70	2060(40)	4260(70)	1430(20)	238(5)	
C72	6750(20)	9450(20)	5305(12)	324(8)	
C73	6900(30)	10780(20)	4826(12)	324(8)	
C71	6421(18)	9780(20)	4778(11)	324(8)	
O40	7723(14)	9832(14)	4258(6)	274(9)	
C16	9408(17)	4110(20)	3805(11)	270	
C26	10420(20)	4350(20)	3624(11)	270	
C80	9577(19)	3750(20)	4339(11)	270	
C35	8435(15)	10554(17)	1134(12)	256(6)	
C50	11870(13)	7004(17)	2151(7)	210(4)	
C55	9149(17)	11595(16)	3034(12)	256(6)	
C58	4230(20)	2425(14)	3833(12)	281(7)	
C74	4540(19)	2190(15)	1562(9)	247(6)	
C75	10220(20)	7690(20)	-224(13)	280	
C122	8060(19)	11115(15)	1525(10)	256(6)	
C86	2080(50)	11240(30)	2141(18)	334(9)	
C119	2890(30)	10200(40)	1280(20)	334(9)	
C76	3155(14)	5889(14)	103(8)	183(4)	
C77	7896(19)	6649(16)	5197(8)	244(6)	
C94	6500(20)	11178(19)	4347(12)	324(8)	
C78	8670(20)	3984(19)	1136(10)	356(9)	
C79	8677(19)	3710(20)	4617(12)	270	
C40	2585(17)	3831(19)	2828(11)	238(5)	
C81	3680(15)	10130(14)	2954(10)	208(5)	


Table 0: Anisotropic Displacement Parameters (×104) for mjp184_150k_new. The anisotropic displacement factor exponent takes the form: -2p2[h2a*2 × U11+ ... +2hka* × b* × U12]

Atom	U11	U22	U33	U23	U13	U12	
Zr1	99.5(6)	102.9(7)	69.2(5)	-8.3(4)	-2.3(4)	27.9(5)	
Zr2	97.8(6)	101.6(7)	81.7(6)	-16.5(5)	-5.0(4)	34.5(5)	
Zr3	88.6(6)	113.0(7)	89.0(6)	-16.0(5)	-0.8(5)	37.4(5)	
Zr4	102.5(7)	128.6(8)	69.7(5)	-5.6(5)	-3.4(4)	41.6(6)	
Zr5	99.5(6)	101.1(7)	83.7(6)	-5.0(5)	-3.5(5)	30.3(5)	
Zr6	88.8(6)	126.0(8)	85.8(6)	-8.3(5)	-0.1(5)	41.5(5)	
O1	98(4)	112(5)	75(4)	-9(3)	0(3)	47(4)	
O2	128(6)	130(6)	84(5)	-8(4)	-34(4)	37(5)	
O3	90(4)	110(5)	76(4)	-9(4)	-4(3)	26(4)	
O4	101(5)	127(6)	78(4)	-18(4)	-1(4)	42(4)	
O5	127(7)	176(9)	115(6)	-10(6)	5(5)	89(7)	
O6	124(6)	125(7)	97(5)	6(5)	-5(4)	31(5)	
O7	92(5)	142(7)	130(7)	-1(6)	-14(5)	47(5)	
O8	94(4)	105(5)	85(4)	-17(4)	5(3)	28(4)	
O9	123(6)	101(5)	112(6)	-8(4)	-2(5)	34(5)	
O10	110(6)	188(10)	121(7)	-6(6)	-6(5)	73(7)	
O11	184(9)	104(6)	110(6)	-13(5)	-14(6)	61(6)	
O12	138(7)	166(9)	102(6)	43(6)	16(5)	61(7)	
O13	92(4)	108(5)	76(4)	0(3)	2(3)	37(4)	
O14	79(5)	186(9)	103(6)	4(6)	3(4)	28(5)	
O15	97(5)	107(5)	90(4)	7(4)	1(4)	32(4)	
O16	114(5)	126(6)	77(4)	-13(4)	-3(4)	24(5)	
O17	92(4)	111(5)	79(4)	-17(4)	-7(3)	39(4)	
O18	125(6)	106(6)	113(6)	3(5)	1(5)	29(5)	
O19	100(5)	98(5)	80(4)	-3(3)	5(3)	29(4)	
O20	122(7)	144(8)	125(7)	-13(6)	2(5)	67(6)	
O21	117(6)	125(7)	114(6)	-34(5)	-4(5)	28(5)	
O22	111(6)	138(7)	110(7)	-15(5)	15(5)	38(5)	
O23	124(6)	186(9)	98(6)	-2(6)	-27(5)	70(6)	
O24	110(6)	125(7)	113(6)	9(5)	1(5)	9(5)	
O25	109(6)	140(7)	84(5)	-15(4)	12(5)	29(5)	
O26	132(7)	110(6)	92(5)	-24(4)	-12(5)	25(5)	
O27	84(5)	137(7)	126(7)	-14(5)	-5(4)	25(5)	
O28	153(8)	118(6)	109(6)	-23(5)	8(5)	57(6)	
O29	126(6)	120(6)	118(6)	26(5)	-1(5)	28(5)	
O30	118(6)	145(8)	125(7)	-65(6)	9(5)	-7(6)	
O31	150(8)	158(8)	83(5)	-24(5)	-15(5)	47(6)	
O32	137(7)	180(9)	82(5)	1(5)	19(5)	61(7)	
O33	106(6)	192(10)	107(7)	-8(6)	18(5)	57(6)	
C1	274(12)	156(8)	239(12)	29(9)	-64(10)	10(8)	
C2	160(7)	205(9)	174(8)	7(7)	-33(6)	72(7)	
C3	339(16)	346(16)	391(17)	-162(15)	-112(14)	226(13)	
C4	304(14)	199(11)	238(13)	62(9)	17(11)	51(10)	
O34	163(8)	223(12)	139(8)	-1(7)	30(6)	110(8)	
O35	173(10)	172(10)	132(8)	8(7)	38(7)	38(8)	
C5	218(11)	199(10)	263(12)	-71(9)	-77(10)	13(10)	
C6	157(8)	266(12)	258(12)	45(10)	15(9)	31(9)	
C7	305(13)	236(13)	278(13)	124(11)	30(11)	44(11)	
C8	165(14)	151(13)	122(12)	-26(10)	69(12)	29(12)	
O36	191(11)	167(10)	173(11)	14(8)	12(9)	78(9)	
C9	287(11)	393(16)	208(12)	-115(12)	-12(10)	57(13)	
C10	257(12)	291(14)	125(6)	-43(9)	-53(7)	59(10)	
C11	198(9)	190(9)	261(11)	-12(9)	19(9)	113(7)	
O37	420(30)	320(30)	420(40)	30(20)	-210(30)	200(30)	
C12	304(14)	199(11)	238(13)	62(9)	17(11)	51(10)	
C13	134(11)	221(17)	93(9)	1(10)	18(8)	73(11)	
O39	155(12)	235(18)	310(20)	10(16)	76(13)	-21(11)	
C14	274(12)	156(8)	239(12)	29(9)	-64(10)	10(8)	
C15	330(15)	327(16)	259(14)	116(13)	36(13)	-12(14)	
C17	198(9)	190(9)	261(11)	-12(9)	19(9)	113(7)	
C18	218(11)	199(10)	263(12)	-71(9)	-77(10)	13(10)	
C19	339(16)	346(16)	391(17)	-162(15)	-112(14)	226(13)	
C20	157(8)	266(12)	258(12)	45(10)	15(9)	31(9)	
C21	121(6)	320(12)	178(8)	-45(9)	-22(6)	91(7)	
C23	274(12)	156(8)	239(12)	29(9)	-64(10)	10(8)	
C24	330(15)	327(16)	259(14)	116(13)	36(13)	-12(14)	
C25	257(12)	291(14)	125(6)	-43(9)	-53(7)	59(10)	
C27	160(7)	205(9)	174(8)	7(7)	-33(6)	72(7)	
C29	174(15)	310(20)	157(13)	-2(15)	79(11)	75(14)	
C30	157(8)	266(12)	258(12)	45(10)	15(9)	31(9)	
C31	305(13)	236(13)	278(13)	124(11)	30(11)	44(11)	
C32	121(6)	320(12)	178(8)	-45(9)	-22(6)	91(7)	
C33	121(6)	320(12)	178(8)	-45(9)	-22(6)	91(7)	
C36	274(12)	156(8)	239(12)	29(9)	-64(10)	10(8)	
C37	121(6)	320(12)	178(8)	-45(9)	-22(6)	91(7)	
C38	339(16)	346(16)	391(17)	-162(15)	-112(14)	226(13)	
C28	160(20)	370(30)	210(40)	30(40)	50(30)	60(20)	
C41	198(9)	190(9)	261(11)	-12(9)	19(9)	113(7)	
C42	339(16)	346(16)	391(17)	-162(15)	-112(14)	226(13)	
C43	157(8)	266(12)	258(12)	45(10)	15(9)	31(9)	
C44	160(7)	205(9)	174(8)	7(7)	-33(6)	72(7)	
C45	257(12)	291(14)	125(6)	-43(9)	-53(7)	59(10)	
C46	218(11)	199(10)	263(12)	-71(9)	-77(10)	13(10)	
C47	304(14)	199(11)	238(13)	62(9)	17(11)	51(10)	
C48	218(11)	199(10)	263(12)	-71(9)	-77(10)	13(10)	
C49	257(12)	291(14)	125(6)	-43(9)	-53(7)	59(10)	
C51	198(9)	190(9)	261(11)	-12(9)	19(9)	113(7)	
C52	305(13)	236(13)	278(13)	124(11)	30(11)	44(11)	
C53	160(7)	205(9)	174(8)	7(7)	-33(6)	72(7)	
C56	330(15)	327(16)	259(14)	116(13)	36(13)	-12(14)	
C57	240(30)	360(30)	160(30)	10(30)	110(30)	140(20)	
C59	330(15)	327(16)	259(14)	116(13)	36(13)	-12(14)	
C60	330(15)	327(16)	259(14)	116(13)	36(13)	-12(14)	
C61	330(15)	327(16)	259(14)	116(13)	36(13)	-12(14)	
C62	305(13)	236(13)	278(13)	124(11)	30(11)	44(11)	
C63	160(7)	205(9)	174(8)	7(7)	-33(6)	72(7)	
C64	160(7)	205(9)	174(8)	7(7)	-33(6)	72(7)	
C66	198(9)	190(9)	261(11)	-12(9)	19(9)	113(7)	
C65	198(9)	190(9)	261(11)	-12(9)	19(9)	113(7)	
C67	240(30)	410(40)	149(17)	-30(30)	102(19)	40(30)	
C68	180(30)	330(30)	240(40)	30(30)	130(30)	10(30)	
C69	157(8)	266(12)	258(12)	45(10)	15(9)	31(9)	
C70	157(8)	266(12)	258(12)	45(10)	15(9)	31(9)	
C72	287(11)	393(16)	208(12)	-115(12)	-12(10)	57(13)	
C73	287(11)	393(16)	208(12)	-115(12)	-12(10)	57(13)	
C71	287(11)	393(16)	208(12)	-115(12)	-12(10)	57(13)	
O40	260(12)	340(20)	177(12)	-149(14)	-8(11)	94(13)	
C35	304(14)	199(11)	238(13)	62(9)	17(11)	51(10)	
C50	121(6)	320(12)	178(8)	-45(9)	-22(6)	91(7)	
C55	218(11)	199(10)	263(12)	-71(9)	-77(10)	13(10)	
C58	305(13)	236(13)	278(13)	124(11)	30(11)	44(11)	
C74	274(12)	156(8)	239(12)	29(9)	-64(10)	10(8)	
C122	304(14)	199(11)	238(13)	62(9)	17(11)	51(10)	
C86	330(15)	327(16)	259(14)	116(13)	36(13)	-12(14)	
C119	330(15)	327(16)	259(14)	116(13)	36(13)	-12(14)	
C76	160(7)	205(9)	174(8)	7(7)	-33(6)	72(7)	
C77	257(12)	291(14)	125(6)	-43(9)	-53(7)	59(10)	
C94	287(11)	393(16)	208(12)	-115(12)	-12(10)	57(13)	
C78	339(16)	346(16)	391(17)	-162(15)	-112(14)	226(13)	
C40	157(8)	266(12)	258(12)	45(10)	15(9)	31(9)	
C81	198(9)	190(9)	261(11)	-12(9)	19(9)	113(7)	


Table 0: Bond Lengths in Å for mjp184_150k_new.


Atom	Atom	Length/Å	
Zr1	Zr2	3.4736(12)	
Zr1	Zr3	3.4720(14)	
Zr1	Zr5	3.5729(15)	
Zr1	Zr6	3.5317(14)	
Zr1	O2	2.222(9)	
Zr1	O3	2.272(7)	
Zr1	O6	2.165(10)	
Zr1	O8	2.351(7)	
Zr1	O16	2.221(7)	
Zr1	O17	2.050(7)	
Zr1	O19	2.043(7)	
Zr1	O25	2.175(9)	
Zr2	Zr4	3.5059(16)	
Zr2	Zr6	3.4816(15)	
Zr2	O1	2.097(7)	
Zr2	O4	2.209(8)	
Zr2	O8	2.208(7)	
Zr2	O17	2.021(6)	
Zr2	O18	2.192(9)	
Zr2	O21	2.256(9)	
Zr2	O26	2.265(7)	
Zr2	O28	2.227(9)	
Zr2	C5	2.60(2)	
Zr3	Zr4	3.5121(15)	
Zr3	Zr5	3.4870(14)	
Zr3	O1	2.095(6)	
Zr3	O5	2.198(10)	
Zr3	O8	2.166(8)	
Zr3	O10	2.253(9)	
Zr3	O15	2.202(8)	
Zr3	O19	2.046(7)	
Zr3	O22	2.193(10)	
Zr3	O27	2.258(8)	
Zr3	C37	2.65(2)	
Zr4	Zr5	3.4880(13)	
Zr4	Zr6	3.4754(14)	
Zr4	O1	2.090(8)	
Zr4	O4	2.168(8)	
Zr4	O12	2.222(11)	
Zr4	O13	2.029(6)	
Zr4	O15	2.185(8)	
Zr4	O23	2.264(8)	
Zr4	O31	2.275(8)	
Zr4	O32	2.212(10)	
Zr4	C10	2.66(2)	
Zr5	Zr6	3.5498(15)	
Zr5	O3	2.249(7)	
Zr5	O9	2.124(8)	
Zr5	O11	2.210(10)	
Zr5	O13	2.071(7)	
Zr5	O15	2.387(7)	
Zr5	O19	2.092(7)	
Zr5	O24	2.277(9)	
Zr5	O29	2.144(9)	
Zr6	O3	2.394(7)	
Zr6	O4	2.397(7)	
Zr6	O7	2.181(9)	
Zr6	O13	2.041(7)	
Zr6	O14	2.187(10)	
Zr6	O17	2.068(8)	
Zr6	O20	2.167(10)	
Zr6	O33	2.210(10)	
O2	C2	1.28(2)	
O5	C3	1.23(4)	
O6	C4	1.29(3)	
O7	C2	1.31(3)	
O9	C1	1.22(3)	
O10	C37	1.18(3)	
O11	C3	1.29(4)	
O12	C7	1.30(4)	
O14	C6	1.28(3)	
O18	C4	1.32(3)	
O20	C11	1.24(3)	
O21	C5	1.24(4)	
O22	C8	1.32(2)	
O23	C10	1.27(3)	
O24	C6	1.26(3)	
O25	C8	1.21(2)	
O26	C5	1.25(4)	
O27	C37	1.31(3)	
O28	C11	1.32(3)	
O29	C7	1.29(4)	
O30	C1	1.22(3)	
O31	C10	1.22(3)	
O32	C13	1.282(17)	
O33	C13	1.241(18)	
C1	C14	1.37(3)	
C2	C27	1.447(9)	
C3	C19	1.461(10)	
C4	C12	1.437(10)	
O34	C9	1.22(3)	
O35	C15	1.14(4)	
C5	C18	1.56(3)	
C6	C20	1.458(10)	
C7	C31	1.424(10)	
C8	C34	1.771(14)	
O36	C22	1.25(4)	
C9	C71	1.462(10)	
C9	O40	1.36(3)	
C10	C25	1.49(3)	
C11	C17	1.43(3)	
O37	C22	1.30(4)	
C12	C47	1.508(10)	
C12	C35	1.487(10)	
C13	C29	1.53(2)	
O39	C15	1.29(4)	
C14	C23	1.47(3)	
C14	C36	1.487(10)	
C15	C24	1.50(5)	
C17	C41	1.500(10)	
C17	C51	1.490(10)	
C17	C66	1.502(10)	
C17	C65	1.501(10)	
C18	C46	1.501(10)	
C18	C48	1.496(10)	
C19	C38	1.498(5)	
C19	C42	1.499(4)	
C20	C30	1.498(10)	
C20	C43	1.495(10)	
C20	C69	1.495(10)	
C20	C70	1.503(10)	
C21	C32	1.48(3)	
C21	C33	1.492(10)	
C21	C37	1.52(3)	
C22	C16	1.451(9)	
C23	C74	1.503(10)	
C24	C56	1.502(10)	
C24	C59	1.502(7)	
C24	C60	1.502(10)	
C24	C61	1.501(10)	
C25	C45	1.489(10)	
C25	C49	1.56(3)	
C27	C44	1.504(10)	
C27	C53	1.498(7)	
C27	C63	1.503(10)	
C27	C64	1.501(7)	
C29	C28	1.498(10)	
C29	C57	1.494(10)	
C29	C67	1.501(10)	
C29	C68	1.508(10)	
C31	C52	1.504(10)	
C31	C62	1.501(10)	
C33	C50	1.470(10)	
C34	C39	1.503(10)	
C34	C54	1.487(10)	
C42	C78	1.504(10)	
C43	C40	1.507(10)	
C45	C77	1.488(10)	
C48	C55	1.499(10)	
C51	C81	1.503(10)	
C53	C76	1.498(10)	
C54	C75	1.502(10)	
C56	C86	1.499(10)	
C59	C119	1.498(10)	
C62	C58	1.507(10)	
C64	C76	1.501(10)	
C65	C81	1.505(10)	
C69	C40	1.504(10)	
C72	C71	1.504(10)	
C73	C71	1.496(10)	
C73	C94	1.509(10)	
C16	C26	1.492(7)	
C16	C80	1.485(9)	
C80	C79	1.499(10)	
C35	C122	1.506(10)	


Table 0: Bond Angles in ° for mjp184_150k_new.


Atom	Atom	Atom	Angle/°	
Zr2	Zr1	Zr5	89.91(3)	
Zr2	Zr1	Zr6	59.60(3)	
Zr3	Zr1	Zr2	60.82(3)	
Zr3	Zr1	Zr5	59.32(3)	
Zr3	Zr1	Zr6	89.81(3)	
Zr6	Zr1	Zr5	59.95(3)	
O2	Zr1	Zr2	105.8(2)	
O2	Zr1	Zr3	161.9(2)	
O2	Zr1	Zr5	111.5(3)	
O2	Zr1	Zr6	72.3(2)	
O2	Zr1	O3	74.0(3)	
O2	Zr1	O8	138.1(3)	
O3	Zr1	Zr2	98.10(16)	
O3	Zr1	Zr3	94.86(18)	
O3	Zr1	Zr5	37.55(18)	
O3	Zr1	Zr6	42.13(17)	
O3	Zr1	O8	122.0(3)	
O6	Zr1	Zr2	73.8(2)	
O6	Zr1	Zr3	111.3(2)	
O6	Zr1	Zr5	163.7(2)	
O6	Zr1	Zr6	109.2(3)	
O6	Zr1	O2	73.2(3)	
O6	Zr1	O3	142.4(3)	
O6	Zr1	O8	74.4(3)	
O6	Zr1	O16	112.4(3)	
O6	Zr1	O25	78.7(4)	
O8	Zr1	Zr2	38.86(17)	
O8	Zr1	Zr3	37.84(19)	
O8	Zr1	Zr5	93.6(2)	
O8	Zr1	Zr6	94.38(18)	
O16	Zr1	Zr2	173.5(2)	
O16	Zr1	Zr3	117.0(3)	
O16	Zr1	Zr5	83.9(3)	
O16	Zr1	Zr6	115.1(2)	
O16	Zr1	O2	74.8(3)	
O16	Zr1	O3	75.7(3)	
O16	Zr1	O8	143.3(3)	
O17	Zr1	Zr2	31.20(19)	
O17	Zr1	Zr3	81.86(19)	
O17	Zr1	Zr5	81.3(2)	
O17	Zr1	Zr6	31.1(2)	
O17	Zr1	O2	81.2(3)	
O17	Zr1	O3	73.0(3)	
O17	Zr1	O6	84.1(3)	
O17	Zr1	O8	69.7(3)	
O17	Zr1	O16	144.7(3)	
O17	Zr1	O25	142.7(3)	
O19	Zr1	Zr2	82.98(19)	
O19	Zr1	Zr3	31.91(19)	
O19	Zr1	Zr5	30.6(2)	
O19	Zr1	Zr6	81.7(2)	
O19	Zr1	O2	142.1(3)	
O19	Zr1	O3	68.2(3)	
O19	Zr1	O6	143.1(3)	
O19	Zr1	O8	69.6(3)	
O19	Zr1	O16	92.6(3)	
O19	Zr1	O17	90.7(3)	
O19	Zr1	O25	83.9(3)	
O25	Zr1	Zr2	111.6(2)	
O25	Zr1	Zr3	74.2(2)	
O25	Zr1	Zr5	109.0(3)	
O25	Zr1	Zr6	164.0(2)	
O25	Zr1	O2	123.7(3)	
O25	Zr1	O3	136.3(3)	
O25	Zr1	O8	73.9(3)	
O25	Zr1	O16	72.5(3)	
Zr1	Zr2	Zr4	90.32(3)	
Zr1	Zr2	Zr6	61.03(3)	
Zr6	Zr2	Zr4	59.65(3)	
O1	Zr2	Zr1	85.50(18)	
O1	Zr2	Zr4	33.1(2)	
O1	Zr2	Zr6	85.4(2)	
O1	Zr2	O4	69.3(3)	
O1	Zr2	O8	68.9(3)	
O1	Zr2	O18	143.9(3)	
O1	Zr2	O21	90.9(3)	
O1	Zr2	O26	90.0(3)	
O1	Zr2	O28	142.8(3)	
O1	Zr2	C5	91.2(8)	
O4	Zr2	Zr1	99.80(18)	
O4	Zr2	Zr4	36.4(2)	
O4	Zr2	Zr6	42.97(17)	
O4	Zr2	O21	134.6(3)	
O4	Zr2	O26	81.7(3)	
O4	Zr2	O28	74.5(3)	
O4	Zr2	C5	108.8(9)	
O8	Zr2	Zr1	41.93(18)	
O8	Zr2	Zr4	94.2(2)	
O8	Zr2	Zr6	98.51(19)	
O8	Zr2	O4	124.1(3)	
O8	Zr2	O21	80.9(3)	
O8	Zr2	O26	133.0(3)	
O8	Zr2	O28	143.0(3)	
O8	Zr2	C5	107.5(9)	
O17	Zr2	Zr1	31.7(2)	
O17	Zr2	Zr4	81.5(2)	
O17	Zr2	Zr6	32.1(2)	
O17	Zr2	O1	94.4(3)	
O17	Zr2	O4	74.7(3)	
O17	Zr2	O8	73.2(3)	
O17	Zr2	O18	82.3(3)	
O17	Zr2	O21	149.6(3)	
O17	Zr2	O26	152.6(3)	
O17	Zr2	O28	83.6(3)	
O17	Zr2	C5	174.2(8)	
O18	Zr2	Zr1	72.9(2)	
O18	Zr2	Zr4	162.9(2)	
O18	Zr2	Zr6	107.6(2)	
O18	Zr2	O4	141.7(3)	
O18	Zr2	O8	75.8(3)	
O18	Zr2	O21	75.9(3)	
O18	Zr2	O26	109.3(3)	
O18	Zr2	O28	72.8(4)	
O18	Zr2	C5	92.2(9)	
O21	Zr2	Zr1	119.5(2)	
O21	Zr2	Zr4	116.6(3)	
O21	Zr2	Zr6	176.3(3)	
O21	Zr2	O26	57.1(3)	
O21	Zr2	C5	28.4(9)	
O26	Zr2	Zr1	174.4(3)	
O26	Zr2	Zr4	87.7(3)	
O26	Zr2	Zr6	122.0(3)	
O26	Zr2	C5	28.7(9)	
O28	Zr2	Zr1	108.9(2)	
O28	Zr2	Zr4	110.7(3)	
O28	Zr2	Zr6	73.3(3)	
O28	Zr2	O21	109.4(4)	
O28	Zr2	O26	76.8(3)	
O28	Zr2	C5	92.9(8)	
C5	Zr2	Zr1	147.9(9)	
C5	Zr2	Zr4	104.1(8)	
C5	Zr2	Zr6	150.6(9)	
Zr1	Zr3	Zr4	90.24(3)	
Zr1	Zr3	Zr5	61.78(3)	
Zr5	Zr3	Zr4	59.78(3)	
O1	Zr3	Zr1	85.57(18)	
O1	Zr3	Zr4	32.9(2)	
O1	Zr3	Zr5	85.8(2)	
O1	Zr3	O5	142.8(4)	
O1	Zr3	O8	69.8(3)	
O1	Zr3	O10	89.1(3)	
O1	Zr3	O15	69.4(3)	
O1	Zr3	O22	142.9(3)	
O1	Zr3	O27	89.6(3)	
O1	Zr3	C37	91.3(6)	
O5	Zr3	Zr1	110.2(2)	
O5	Zr3	Zr4	111.2(3)	
O5	Zr3	Zr5	73.9(3)	
O5	Zr3	O10	76.3(4)	
O5	Zr3	O15	74.7(3)	
O5	Zr3	O27	108.7(4)	
O5	Zr3	C37	90.1(7)	
O8	Zr3	Zr1	41.77(18)	
O8	Zr3	Zr4	94.77(19)	
O8	Zr3	Zr5	99.51(19)	
O8	Zr3	O5	143.2(3)	
O8	Zr3	O10	132.4(4)	
O8	Zr3	O15	124.9(3)	
O8	Zr3	O22	74.4(3)	
O8	Zr3	O27	81.2(3)	
O8	Zr3	C37	109.4(8)	
O10	Zr3	Zr1	173.5(3)	
O10	Zr3	Zr4	87.3(3)	
O10	Zr3	Zr5	121.6(3)	
O10	Zr3	O27	55.7(4)	
O10	Zr3	C37	26.4(8)	
O15	Zr3	Zr1	99.96(19)	
O15	Zr3	Zr4	36.6(2)	
O15	Zr3	Zr5	42.58(18)	
O15	Zr3	O10	81.6(4)	
O15	Zr3	O27	133.1(3)	
O15	Zr3	C37	106.9(8)	
O19	Zr3	Zr1	31.9(2)	
O19	Zr3	Zr4	82.3(2)	
O19	Zr3	Zr5	33.0(2)	
O19	Zr3	O1	95.4(3)	
O19	Zr3	O5	84.4(3)	
O19	Zr3	O8	73.4(3)	
O19	Zr3	O10	153.1(4)	
O19	Zr3	O15	75.3(3)	
O19	Zr3	O22	83.1(3)	
O19	Zr3	O27	150.5(3)	
O19	Zr3	C37	173.3(6)	
O22	Zr3	Zr1	73.7(2)	
O22	Zr3	Zr4	163.9(2)	
O22	Zr3	Zr5	109.5(2)	
O22	Zr3	O5	74.1(4)	
O22	Zr3	O10	108.9(4)	
O22	Zr3	O15	143.5(3)	
O22	Zr3	O27	75.7(3)	
O22	Zr3	C37	91.8(7)	
O27	Zr3	Zr1	120.4(3)	
O27	Zr3	Zr4	115.0(2)	
O27	Zr3	Zr5	174.7(2)	
O27	Zr3	C37	29.5(8)	
C37	Zr3	Zr1	149.9(8)	
C37	Zr3	Zr4	103.2(6)	
C37	Zr3	Zr5	148.0(8)	
Zr5	Zr4	Zr2	90.79(3)	
Zr6	Zr4	Zr2	59.83(3)	
Zr6	Zr4	Zr5	61.30(3)	
O1	Zr4	Zr2	33.20(18)	
O1	Zr4	Zr5	85.85(18)	
O1	Zr4	Zr6	85.70(18)	
O1	Zr4	O4	70.2(3)	
O1	Zr4	O12	142.7(3)	
O1	Zr4	O15	69.8(3)	
O1	Zr4	O23	90.3(3)	
O1	Zr4	O31	89.4(3)	
O1	Zr4	O32	143.5(3)	
O1	Zr4	C10	90.2(7)	
O4	Zr4	Zr2	37.2(2)	
O4	Zr4	Zr5	100.40(18)	
O4	Zr4	Zr6	42.94(18)	
O4	Zr4	O12	142.3(3)	
O4	Zr4	O15	126.0(3)	
O4	Zr4	O23	132.8(3)	
O4	Zr4	O31	80.8(3)	
O4	Zr4	O32	74.2(3)	
O4	Zr4	C10	106.6(8)	
O12	Zr4	Zr2	163.1(2)	
O12	Zr4	Zr5	72.4(3)	
O12	Zr4	Zr6	108.4(2)	
O12	Zr4	O23	76.5(4)	
O12	Zr4	O31	110.1(4)	
O12	Zr4	C10	93.6(8)	
O13	Zr4	Zr2	80.8(2)	
O13	Zr4	Zr5	32.08(19)	
O13	Zr4	Zr6	31.5(2)	
O13	Zr4	O1	93.9(3)	
O13	Zr4	O4	74.1(3)	
O13	Zr4	O12	83.6(3)	
O13	Zr4	O15	74.1(3)	
O13	Zr4	O23	152.1(3)	
O13	Zr4	O31	151.8(3)	
O13	Zr4	O32	83.5(3)	
O13	Zr4	C10	175.9(8)	
O15	Zr4	Zr2	95.4(2)	
O15	Zr4	Zr5	42.49(18)	
O15	Zr4	Zr6	99.62(19)	
O15	Zr4	O12	73.8(3)	
O15	Zr4	O23	81.5(3)	
O15	Zr4	O31	132.6(3)	
O15	Zr4	O32	141.9(3)	
O15	Zr4	C10	108.2(8)	
O23	Zr4	Zr2	115.4(3)	
O23	Zr4	Zr5	121.2(3)	
O23	Zr4	Zr6	175.1(3)	
O23	Zr4	O31	55.7(4)	
O23	Zr4	C10	28.5(8)	
O31	Zr4	Zr2	86.8(3)	
O31	Zr4	Zr5	174.3(3)	
O31	Zr4	Zr6	121.4(3)	
O31	Zr4	C10	27.2(8)	
O32	Zr4	Zr2	111.3(3)	
O32	Zr4	Zr5	108.2(3)	
O32	Zr4	Zr6	73.2(2)	
O32	Zr4	O12	73.4(4)	
O32	Zr4	O23	108.8(4)	
O32	Zr4	O31	77.4(4)	
O32	Zr4	C10	92.7(8)	
C10	Zr4	Zr2	102.2(8)	
C10	Zr4	Zr5	149.5(8)	
C10	Zr4	Zr6	148.5(8)	
Zr3	Zr5	Zr1	58.90(3)	
Zr3	Zr5	Zr4	60.47(3)	
Zr3	Zr5	Zr6	89.28(3)	
Zr4	Zr5	Zr1	88.99(3)	
Zr4	Zr5	Zr6	59.18(3)	
Zr6	Zr5	Zr1	59.45(3)	
O3	Zr5	Zr1	38.00(19)	
O3	Zr5	Zr3	94.89(18)	
O3	Zr5	Zr4	97.42(17)	
O3	Zr5	Zr6	41.68(17)	
O3	Zr5	O15	122.0(3)	
O3	Zr5	O24	73.1(3)	
O9	Zr5	Zr1	89.3(2)	
O9	Zr5	Zr3	120.3(3)	
O9	Zr5	Zr4	177.2(2)	
O9	Zr5	Zr6	118.0(2)	
O9	Zr5	O3	79.9(3)	
O9	Zr5	O11	73.7(3)	
O9	Zr5	O15	144.2(3)	
O9	Zr5	O24	74.5(3)	
O9	Zr5	O29	107.2(4)	
O11	Zr5	Zr1	108.3(3)	
O11	Zr5	Zr3	71.7(3)	
O11	Zr5	Zr4	109.0(2)	
O11	Zr5	Zr6	160.9(3)	
O11	Zr5	O3	137.5(3)	
O11	Zr5	O15	71.5(3)	
O11	Zr5	O24	128.2(4)	
O13	Zr5	Zr1	79.5(2)	
O13	Zr5	Zr3	80.9(2)	
O13	Zr5	Zr4	31.36(18)	
O13	Zr5	Zr6	30.06(18)	
O13	Zr5	O3	71.5(3)	
O13	Zr5	O9	146.0(3)	
O13	Zr5	O11	140.3(3)	
O13	Zr5	O15	69.1(3)	
O13	Zr5	O19	88.8(3)	
O13	Zr5	O24	79.8(3)	
O13	Zr5	O29	85.8(3)	
O15	Zr5	Zr1	93.63(19)	
O15	Zr5	Zr3	38.60(18)	
O15	Zr5	Zr4	38.18(18)	
O15	Zr5	Zr6	93.69(19)	
O19	Zr5	Zr1	29.8(2)	
O19	Zr5	Zr3	32.17(19)	
O19	Zr5	Zr4	82.36(18)	
O19	Zr5	Zr6	80.7(2)	
O19	Zr5	O3	67.8(3)	
O19	Zr5	O9	97.2(3)	
O19	Zr5	O11	83.0(3)	
O19	Zr5	O15	70.5(3)	
O19	Zr5	O24	141.0(3)	
O19	Zr5	O29	143.5(3)	
O24	Zr5	Zr1	111.1(2)	
O24	Zr5	Zr3	159.7(3)	
O24	Zr5	Zr4	104.1(2)	
O24	Zr5	Zr6	70.8(3)	
O24	Zr5	O15	135.8(3)	
O29	Zr5	Zr1	163.4(3)	
O29	Zr5	Zr3	111.5(2)	
O29	Zr5	Zr4	74.5(3)	
O29	Zr5	Zr6	109.5(3)	
O29	Zr5	O3	142.0(3)	
O29	Zr5	O11	78.6(4)	
O29	Zr5	O15	73.9(3)	
O29	Zr5	O24	73.1(3)	
Zr1	Zr6	Zr5	60.60(3)	
Zr2	Zr6	Zr1	59.37(3)	
Zr2	Zr6	Zr5	90.16(3)	
Zr4	Zr6	Zr1	89.86(3)	
Zr4	Zr6	Zr2	60.52(3)	
Zr4	Zr6	Zr5	59.53(3)	
O3	Zr6	Zr1	39.52(18)	
O3	Zr6	Zr2	95.49(17)	
O3	Zr6	Zr4	94.95(17)	
O3	Zr6	Zr5	38.64(17)	
O3	Zr6	O4	121.0(2)	
O4	Zr6	Zr1	94.53(18)	
O4	Zr6	Zr2	38.91(19)	
O4	Zr6	Zr4	38.0(2)	
O4	Zr6	Zr5	94.20(19)	
O7	Zr6	Zr1	73.8(2)	
O7	Zr6	Zr2	109.2(3)	
O7	Zr6	Zr4	163.6(3)	
O7	Zr6	Zr5	110.4(2)	
O7	Zr6	O3	72.5(3)	
O7	Zr6	O4	141.2(3)	
O7	Zr6	O14	77.4(4)	
O7	Zr6	O33	122.6(4)	
O13	Zr6	Zr1	80.9(2)	
O13	Zr6	Zr2	81.3(2)	
O13	Zr6	Zr4	31.27(19)	
O13	Zr6	Zr5	30.55(18)	
O13	Zr6	O3	69.0(2)	
O13	Zr6	O4	69.0(3)	
O13	Zr6	O7	140.9(3)	
O13	Zr6	O14	85.1(3)	
O13	Zr6	O17	89.2(3)	
O13	Zr6	O20	142.5(3)	
O13	Zr6	O33	85.1(3)	
O14	Zr6	Zr1	111.7(2)	
O14	Zr6	Zr2	164.8(3)	
O14	Zr6	Zr4	109.6(3)	
O14	Zr6	Zr5	74.7(3)	
O14	Zr6	O3	73.1(3)	
O14	Zr6	O4	139.8(3)	
O14	Zr6	O33	75.3(4)	
O17	Zr6	Zr1	30.81(18)	
O17	Zr6	Zr2	31.24(17)	
O17	Zr6	Zr4	81.73(19)	
O17	Zr6	Zr5	81.7(2)	
O17	Zr6	O3	70.1(3)	
O17	Zr6	O4	69.9(3)	
O17	Zr6	O7	84.1(3)	
O17	Zr6	O14	142.3(3)	
O17	Zr6	O20	84.1(3)	
O17	Zr6	O33	141.3(3)	
O20	Zr6	Zr1	108.8(3)	
O20	Zr6	Zr2	73.9(3)	
O20	Zr6	Zr4	111.3(2)	
O20	Zr6	Zr5	164.1(3)	
O20	Zr6	O3	140.2(3)	
O20	Zr6	O4	74.1(3)	
O20	Zr6	O7	75.1(3)	
O20	Zr6	O14	121.3(4)	
O20	Zr6	O33	77.7(4)	
O33	Zr6	Zr1	163.6(2)	
O33	Zr6	Zr2	110.2(3)	
O33	Zr6	Zr4	73.7(2)	
O33	Zr6	Zr5	109.5(3)	
O33	Zr6	O3	140.3(4)	
O33	Zr6	O4	72.4(3)	
Zr3	O1	Zr2	114.0(3)	
Zr4	O1	Zr2	113.7(3)	
Zr4	O1	Zr3	114.1(3)	
C2	O2	Zr1	137.2(11)	
Zr1	O3	Zr6	98.3(3)	
Zr5	O3	Zr1	104.4(3)	
Zr5	O3	Zr6	99.7(3)	
Zr2	O4	Zr6	98.1(3)	
Zr4	O4	Zr2	106.5(3)	
Zr4	O4	Zr6	99.0(3)	
C3	O5	Zr3	136.4(16)	
C4	O6	Zr1	138.5(14)	
C2	O7	Zr6	135.6(10)	
Zr2	O8	Zr1	99.2(3)	
Zr3	O8	Zr1	100.4(3)	
Zr3	O8	Zr2	107.0(3)	
C1	O9	Zr5	152.0(19)	
C37	O10	Zr3	96.0(15)	
C3	O11	Zr5	137.6(14)	
C7	O12	Zr4	136.2(15)	
Zr4	O13	Zr5	116.6(3)	
Zr4	O13	Zr6	117.3(3)	
Zr6	O13	Zr5	119.4(3)	
C6	O14	Zr6	134.8(14)	
Zr3	O15	Zr5	98.8(3)	
Zr4	O15	Zr3	106.4(3)	
Zr4	O15	Zr5	99.3(3)	
Zr1	O17	Zr6	118.1(3)	
Zr2	O17	Zr1	117.1(3)	
Zr2	O17	Zr6	116.7(3)	
C4	O18	Zr2	137.5(14)	
Zr1	O19	Zr3	116.2(3)	
Zr1	O19	Zr5	119.5(3)	
Zr3	O19	Zr5	114.8(3)	
C11	O20	Zr6	137.4(13)	
C5	O21	Zr2	91.3(15)	
C8	O22	Zr3	131.5(10)	
C10	O23	Zr4	93.5(14)	
C6	O24	Zr5	137.1(14)	
C8	O25	Zr1	133.9(10)	
C5	O26	Zr2	90.7(14)	
C37	O27	Zr3	92.2(13)	
C11	O28	Zr2	133.2(13)	
C7	O29	Zr5	137.1(15)	
C10	O31	Zr4	94.4(14)	
C13	O32	Zr4	134.7(9)	
C13	O33	Zr6	134.9(10)	
O9	C1	O30	116(2)	
O9	C1	C14	126(3)	
O30	C1	C14	118(3)	
O2	C2	O7	120.6(13)	
O2	C2	C27	122(2)	
O7	C2	C27	117(2)	
O5	C3	O11	120(2)	
O5	C3	C19	123(3)	
O11	C3	C19	110(3)	
O6	C4	O18	116.9(19)	
O6	C4	C12	117(3)	
O18	C4	C12	124(3)	
O21	C5	Zr2	60.2(10)	
O21	C5	O26	120.8(19)	
O21	C5	C18	121(3)	
O26	C5	Zr2	60.6(10)	
O26	C5	C18	118(3)	
C18	C5	Zr2	173(2)	
O14	C6	C20	118(3)	
O24	C6	O14	122.1(19)	
O24	C6	C20	118(3)	
O12	C7	C31	119(3)	
O29	C7	O12	119.7(18)	
O29	C7	C31	121(3)	
O22	C8	C34	114.0(16)	
O25	C8	O22	126.7(13)	
O25	C8	C34	119.3(16)	
O34	C9	C71	122(3)	
O34	C9	O40	116(3)	
O40	C9	C71	112(2)	
O23	C10	Zr4	58.0(9)	
O23	C10	C25	114(3)	
O31	C10	Zr4	58.4(10)	
O31	C10	O23	116.4(18)	
O31	C10	C25	129(3)	
C25	C10	Zr4	172(3)	
O20	C11	O28	122.1(19)	
O20	C11	C17	123(2)	
O28	C11	C17	115(2)	
C4	C12	C47	117(3)	
C4	C12	C35	99(2)	
C35	C12	C47	132.2(17)	
O32	C13	C29	119.0(17)	
O33	C13	O32	123.2(14)	
O33	C13	C29	117.8(17)	
C1	C14	C23	117(2)	
C1	C14	C36	124(2)	
C23	C14	C36	117(2)	
O35	C15	O39	117(4)	
O35	C15	C24	136(4)	
O39	C15	C24	105(3)	
C11	C17	C41	117(2)	
C11	C17	C51	114(2)	
C11	C17	C66	110(3)	
C11	C17	C65	117(2)	
C41	C17	C65	114(3)	
C51	C17	C66	100.4(9)	
C46	C18	C5	109(2)	
C48	C18	C5	111(2)	
C48	C18	C46	101(2)	
C3	C19	C38	131(3)	
C3	C19	C42	105.8(18)	
C38	C19	C42	107.1(7)	
C6	C20	C30	130(4)	
C6	C20	C43	118(3)	
C6	C20	C69	90(3)	
C6	C20	C70	116(3)	
C43	C20	C30	110(4)	
C69	C20	C70	124(5)	
C32	C21	C33	111.8(19)	
C32	C21	C37	108(2)	
C33	C21	C37	107.4(17)	
O36	C22	O37	118(3)	
O36	C22	C16	122(3)	
O37	C22	C16	120(4)	
C14	C23	C74	99(2)	
C15	C24	C56	110(4)	
C15	C24	C59	98(3)	
C15	C24	C60	108(4)	
C15	C24	C61	116(4)	
C60	C24	C56	108(5)	
C61	C24	C59	138(5)	
C10	C25	C49	108(2)	
C45	C25	C10	108(2)	
C45	C25	C49	104(2)	
C2	C27	C44	106(2)	
C2	C27	C53	126.2(19)	
C2	C27	C63	123(2)	
C2	C27	C64	106(2)	
C53	C27	C44	128(2)	
C64	C27	C63	99.5(8)	
C28	C29	C13	118(3)	
C57	C29	C13	108(2)	
C57	C29	C28	100(4)	
C67	C29	C13	112(2)	
C67	C29	C68	102(5)	
C68	C29	C13	107(2)	
C7	C31	C52	111(3)	
C7	C31	C62	112(2)	
C62	C31	C52	99.1(8)	
C50	C33	C21	117.2(18)	
C39	C34	C8	103.3(14)	
C54	C34	C8	99.0(13)	
C54	C34	C39	155.2(16)	
O10	C37	Zr3	57.7(10)	
O10	C37	O27	115.5(18)	
O10	C37	C21	129(3)	
O27	C37	Zr3	58.3(9)	
O27	C37	C21	115(3)	
C21	C37	Zr3	172(2)	
C19	C42	C78	102.3(7)	
C20	C43	C40	92.6(17)	
C77	C45	C25	104(2)	
C18	C48	C55	110(2)	
C17	C51	C81	119(2)	
C27	C53	C76	110.6(17)	
C34	C54	C75	104.0(19)	
C86	C56	C24	106.6(10)	
C119	C59	C24	105.8(9)	
C31	C62	C58	117.9(19)	
C27	C64	C76	110.3(17)	
C17	C65	C81	118(2)	
C20	C69	C40	92.8(17)	
C71	C73	C94	113(2)	
C9	C71	C72	107(3)	
C9	C71	C73	110(2)	
C73	C71	C72	109(2)	
C22	C16	C26	114(3)	
C22	C16	C80	126(3)	
C80	C16	C26	101.4(8)	
C16	C80	C79	104.8(18)	
C12	C35	C122	103.8(8)	


Table 0: Torsion Angles in ° for mjp184_150k_new.


Atom	Atom	Atom	Atom	Angle/°	
Zr1	O2	C2	O7	-1(3)	
Zr1	O2	C2	C27	-178.2(12)	
Zr1	O6	C4	O18	-2(5)	
Zr1	O6	C4	C12	-166.7(15)	
Zr1	O25	C8	O22	-1(3)	
Zr1	O25	C8	C34	174.7(6)	
Zr2	O18	C4	O6	7(5)	
Zr2	O18	C4	C12	171.0(18)	
Zr2	O21	C5	O26	2(3)	
Zr2	O21	C5	C18	-172(3)	
Zr2	O26	C5	O21	-2(3)	
Zr2	O26	C5	C18	172(2)	
Zr2	O28	C11	O20	2(4)	
Zr2	O28	C11	C17	177.3(14)	
Zr3	O5	C3	O11	-4(5)	
Zr3	O5	C3	C19	-152.5(17)	
Zr3	O10	C37	O27	8(2)	
Zr3	O10	C37	C21	-174(2)	
Zr3	O22	C8	O25	0(3)	
Zr3	O22	C8	C34	-176.3(6)	
Zr3	O27	C37	O10	-8(2)	
Zr3	O27	C37	C21	174.1(17)	
Zr4	O12	C7	O29	-4(5)	
Zr4	O12	C7	C31	176.7(17)	
Zr4	O23	C10	O31	-1(3)	
Zr4	O23	C10	C25	177(2)	
Zr4	O31	C10	O23	1(3)	
Zr4	O31	C10	C25	-177(3)	
Zr4	O32	C13	O33	0(3)	
Zr4	O32	C13	C29	176.8(14)	
Zr5	O9	C1	O30	63(5)	
Zr5	O9	C1	C14	-114(4)	
Zr5	O11	C3	O5	8(5)	
Zr5	O11	C3	C19	160.0(13)	
Zr5	O24	C6	O14	0(5)	
Zr5	O24	C6	C20	164.6(14)	
Zr5	O29	C7	O12	1(5)	
Zr5	O29	C7	C31	-179.7(18)	
Zr6	O7	C2	O2	9(3)	
Zr6	O7	C2	C27	-174.1(11)	
Zr6	O14	C6	O24	-8(5)	
Zr6	O14	C6	C20	-173.0(15)	
Zr6	O20	C11	O28	-4(4)	
Zr6	O20	C11	C17	-178.2(15)	
Zr6	O33	C13	O32	6(3)	
Zr6	O33	C13	C29	-171.4(14)	
O2	C2	C27	C44	-117(3)	
O2	C2	C27	C53	55(4)	
O2	C2	C27	C63	-143(2)	
O2	C2	C27	C64	104(2)	
O5	C3	C19	C38	167(3)	
O5	C3	C19	C42	-62(4)	
O6	C4	C12	C47	64(4)	
O6	C4	C12	C35	-149(3)	
O7	C2	C27	C44	66(3)	
O7	C2	C27	C53	-122(3)	
O7	C2	C27	C63	40(3)	
O7	C2	C27	C64	-73(2)	
O9	C1	C14	C23	-36(6)	
O9	C1	C14	C36	159(3)	
O11	C3	C19	C38	16(4)	
O11	C3	C19	C42	147(2)	
O12	C7	C31	C52	-145(3)	
O12	C7	C31	C62	105(3)	
O14	C6	C20	C30	54(7)	
O14	C6	C20	C43	-145(3)	
O14	C6	C20	C69	-64(3)	
O14	C6	C20	C70	64(6)	
O18	C4	C12	C47	-100(3)	
O18	C4	C12	C35	47(4)	
O20	C11	C17	C41	179(3)	
O20	C11	C17	C51	-5(4)	
O20	C11	C17	C66	-117(3)	
O20	C11	C17	C65	41(4)	
O21	C5	C18	C46	-48(4)	
O21	C5	C18	C48	62(4)	
O22	C8	C34	C39	35.8(17)	
O22	C8	C34	C54	-133.3(17)	
O23	C10	C25	C45	-120(3)	
O23	C10	C25	C49	128(3)	
O24	C6	C20	C30	-111(6)	
O24	C6	C20	C43	50(4)	
O24	C6	C20	C69	130(3)	
O24	C6	C20	C70	-102(6)	
O25	C8	C34	C39	-140.7(18)	
O25	C8	C34	C54	50(2)	
O26	C5	C18	C46	138(3)	
O26	C5	C18	C48	-112(3)	
O28	C11	C17	C41	5(4)	
O28	C11	C17	C51	-180(2)	
O28	C11	C17	C66	68(3)	
O28	C11	C17	C65	-134(3)	
O29	C7	C31	C52	36(4)	
O29	C7	C31	C62	-74(4)	
O30	C1	C14	C23	148(3)	
O30	C1	C14	C36	-17(6)	
O31	C10	C25	C45	58(4)	
O31	C10	C25	C49	-54(4)	
O32	C13	C29	C28	-122(4)	
O32	C13	C29	C57	126(3)	
O32	C13	C29	C67	38(5)	
O32	C13	C29	C68	-72(4)	
O33	C13	C29	C28	55(5)	
O33	C13	C29	C57	-57(3)	
O33	C13	C29	C67	-145(4)	
O33	C13	C29	C68	105(4)	
C1	C14	C23	C74	94(3)	
C2	C27	C53	C76	28(5)	
C2	C27	C64	C76	-74(3)	
C3	C19	C42	C78	-45(3)	
C4	C12	C35	C122	-95(3)	
O34	C9	C71	C72	131(4)	
O34	C9	C71	C73	-110(4)	
O35	C15	C24	C56	105(7)	
O35	C15	C24	C59	-98(6)	
O35	C15	C24	C60	-13(8)	
O35	C15	C24	C61	56(8)	
C5	C18	C48	C55	58(3)	
C6	C20	C43	C40	59(4)	
C6	C20	C69	C40	-92(3)	
C7	C31	C62	C58	165(3)	
C8	C34	C54	C75	161(2)	
O36	C22	C16	C26	100(4)	
O36	C22	C16	C80	-134(4)	
C10	C25	C45	C77	67(3)	
C11	C17	C51	C81	155(3)	
C11	C17	C65	C81	-147(3)	
O37	C22	C16	C26	-73(5)	
O37	C22	C16	C80	53(6)	
O39	C15	C24	C56	-95(4)	
O39	C15	C24	C59	62(5)	
O39	C15	C24	C60	147(5)	
O39	C15	C24	C61	-144(5)	
C15	C24	C56	C86	86(5)	
C15	C24	C59	C119	-129(4)	
C22	C16	C80	C79	36(5)	
C30	C20	C43	C40	-136(5)	
C32	C21	C33	C50	179(2)	
C32	C21	C37	O10	-44(3)	
C32	C21	C37	O27	134(2)	
C33	C21	C37	O10	76(3)	
C33	C21	C37	O27	-106(2)	
C36	C14	C23	C74	-101(3)	
C37	C21	C33	C50	61(3)	
C38	C19	C42	C78	98(2)	
C39	C34	C54	C75	7(6)	
C41	C17	C65	C81	73(4)	
C44	C27	C53	C76	-162(3)	
C46	C18	C48	C55	174.2(18)	
C47	C12	C35	C122	45(4)	
C49	C25	C45	C77	-178(2)	
C52	C31	C62	C58	47(3)	
C60	C24	C56	C86	-156(5)	
C61	C24	C59	C119	87(7)	
C63	C27	C64	C76	157(3)	
C66	C17	C51	C81	-88(3)	
C70	C20	C69	C40	146(5)	
O40	C9	C71	C72	-86(4)	
O40	C9	C71	C73	33(4)	
C26	C16	C80	C79	167(3)	
C94	C73	C71	C9	70(4)	
C94	C73	C71	C72	-172(3)	


Table 0: Hydrogen Fractional Atomic Coordinates (×104) and Equivalent Isotropic Displacement Parameters (Å2×103) for mjp184_150k_new. Ueq is defined as 1/3 of the trace of the orthogonalised Uij.

Atom	x	y	z	Ueq	
H3	4708.47	5611.15	1288.1	145	
H4	6338.65	8163.77	3321.77	156	
H8	8689.06	8184.45	1657.95	148	
H15	7768.81	5390.01	2933.09	150	
H16A	5328.47	5624.44	410.45	170	
H16B	6252.08	5660.49	316.87	170	
H37	9338.22	5955.57	3834.12	590	
H12	7821.97	9717.58	534.12	307	
H39	608.47	8834.02	2077.16	382	
H14	3871.53	2985.73	1174.28	297	
H17	4814.77	9794.72	2287.31	250	
H17A	5564.55	10159.24	3020.18	250	
H18	9789.12	10557.55	3807.2	308	
H19	8678.36	4007.92	2432.71	428	
H20A	2650.53	3607.73	1976.14	286	
H20	2326.3	4474.26	2305.3	286	
H21	11655.87	8325.47	2915.74	252	
H23A	4504.14	1728.47	731.37	297	
H23B	5476.5	2620.61	959.44	297	
H24	1888.06	10132.27	1301.5	401	
H24A	1813.41	10523.38	1606.22	401	
H25	9450.53	7179.64	4723.29	293	
H27	3071.2	7049.2	843.44	219	
H27A	3968.89	7659	340.78	219	
H29	4438.74	6546.06	4396.99	255	
H29A	3555	6866	3924.61	255	
H30A	1704.16	4747.09	1553.55	358	
H30B	2046.43	4085.55	1152.86	358	
H30C	1175.89	3673.69	1522.09	358	
H31	6107.33	3946.37	4011.13	338	
H32A	12202.34	8037.37	3786.17	315	
H32B	11089	8067.46	3766.19	315	
H32C	11259.56	7115.49	3727.42	315	
H33A	12636.65	7381.66	2913.95	252	
H33B	11636.47	6500.25	2889.15	252	
H34	8772.76	6276.33	117.86	336	
H36A	2725.14	2807.36	617.66	371	
H36B	3314.46	2629.16	121.46	371	
H36C	2877.51	1853.65	506.33	371	
H38A	7246.94	2784.68	2343.89	534	
H38B	7298.1	2567.94	1694.7	534	
H38C	8150.32	2497.57	2156.3	534	
H39A	10215.29	6215.66	906.33	420	
H39B	9259.4	5410.26	574.23	420	
H39C	10167.76	5927.32	253.85	420	
H41A	6487.35	10762.55	3062.83	312	
H41B	6346.66	10820.33	2405.45	312	
H41C	5702.77	11194.15	2797.13	312	
H42A	9533.1	3434.78	1686.94	428	
H42B	9542.46	4459.52	1799.92	428	
H43A	2853.78	3126.24	2151.82	286	
H43B	1649.77	2877.26	2104.19	286	
H44A	3992.16	8675.61	674.06	274	
H44B	2891.91	8239.47	841.79	274	
H44C	3863.93	8465.92	1301.17	274	
H45A	9012.95	7859.97	5545.12	293	
H45B	8091.16	7982.01	5149.71	293	
H46A	10886.35	9881.54	3587.61	385	
H46B	11064.96	10348.54	3028.55	385	
H46C	11457.34	10950.26	3625.19	385	
H47A	6296.71	9267.65	347.04	384	
H47B	5983.12	9779.71	852.03	384	
H47C	6550.92	10338.84	391.53	384	
H48A	10268.92	11353.2	2743.31	308	
H48B	10624.28	11897.31	3365.99	308	
H49A	10076.87	8681.72	4373.86	366	
H49B	9320.96	8974.88	4721.71	366	
H49C	10264.4	8795.52	5042.05	366	
H51A	3872.64	9518.84	2224.43	250	
H51B	3834.11	8915.59	2734.36	250	
H52A	6659.32	3161.29	3409.46	422	
H52B	5666.07	2419.03	3552.84	422	
H52C	5671.64	2771.75	2952.96	422	
H53A	2518.32	6892.7	-50.77	219	
H53B	3674.43	7067.66	-134.86	219	
H54A	9429.46	8039.99	376.03	336	
H54B	8714.41	7364.05	-156.53	336	
H56A	710.79	11091.63	1798.82	401	
H56B	1558.01	11643.29	1441.6	401	
H59A	1681.2	9679.61	645	401	
H59B	1561.09	9059.42	1149.19	401	
H60A	777.85	9672.41	475.25	501	
H60B	198.89	10319.88	683.39	501	
H60C	1343.64	10745.59	579.61	501	
H61A	223.72	10718.41	953.19	501	
H61B	814.12	11252.36	1546.64	501	
H61C	1321.61	11464.61	993.27	501	
H62A	4303.66	3751.43	3781.2	338	
H62B	4430.27	3211.41	3215.96	338	
H63A	2693.18	7609.13	1135.39	274	
H63B	3516.66	8530.6	1022.6	274	
H63C	2569.98	8004.44	555.93	274	
H64A	2569.96	6212.92	838.96	219	
H64B	2312.16	6596.67	265.74	219	
H66A	6116.72	10619.8	2188.55	312	
H66B	5056.86	9997.57	1839.33	312	
H66C	5140.59	10892.38	2229.91	312	
H65A	4747.53	9817.8	3444.7	250	
H65B	4050.9	9024.96	2960.51	250	
H69A	1609.17	4371.91	2495.79	286	
H69B	2662.87	5177.02	2728.53	286	
H70A	2075	4882.3	1401.43	358	
H70B	2465.26	4105.17	1166.33	358	
H70C	1363.18	3842.96	1342.02	358	
H72A	7481.04	9708.19	5410.29	486	
H72B	6422.01	9659.2	5594.25	486	
H72C	6534.43	8787.14	5266.06	486	
H73A	7544.63	10994.69	5080.05	389	
H73B	6389.85	10954.44	5010.29	389	
H71	5671.55	9595.32	4712.72	389	
H40	7894.56	9406.43	4153.67	410	
H16	9066.26	3483.43	3604.61	324	
H26A	10618.92	4978.85	3792.22	405	
H26B	10899.06	4079.47	3786.16	405	
H26C	10426.19	4328	3220.07	405	
H80A	10160.48	4221.67	4572.48	324	
H80B	9708.45	3174.31	4298.18	324	
H35A	8771.82	10911.66	849.26	307	
H35B	8948.09	10482.75	1417.32	307	
H50A	11293.57	7137.28	1973.25	315	
H50B	12489.2	7401.38	2036.38	315	
H50C	11785.98	6371.24	2036.38	315	
H55A	8841.88	11542.64	2645.83	385	
H55B	8707.67	11128.46	3224.64	385	
H55C	9246.12	12194.62	3224.64	385	
H58A	3913.88	1946.6	3519.14	422	
H58B	4750.95	2273.05	4061.47	422	
H58C	3725.95	2478	4061.47	422	
H74A	4775.07	2789.1	1781.09	371	
H74B	3812.54	1905.92	1555.31	371	
H74C	4883.41	1811.61	1732.19	371	
H75A	9864.01	7516.51	-607.02	420	
H75B	10580.28	8348.37	-164.2	420	
H75C	10699.62	7377.36	-164.2	420	
H12A	7598.8	11350.25	1311.33	384	
H12B	7708.71	10742.31	1793.67	384	
H12C	8635.81	11621.99	1724.59	384	
H86A	2254.99	11771.31	2419.51	501	
H86B	1832.1	10688.42	2321.41	501	
H86C	2670.82	11243.52	1970.83	501	
H11A	3196.9	9900.2	1019.19	501	
H11B	3221.29	10855.71	1306.8	501	
H11C	2965.07	9999.2	1646.86	501	
H76A	3787.93	5830.23	262.16	274	
H76B	2643.49	5657.01	345.41	274	
H76C	2928.51	5541.96	-269.1	274	
H76D	3424.27	6142.8	-225.84	274	
H76E	3679.47	5762.85	341.59	274	
H76F	2582.9	5326.82	-16.19	274	
H77A	8334.07	6288.06	5240.18	366	
H77B	7526.54	6616.28	5517.05	366	
H77C	7418.95	6410.89	4852.35	366	
H94A	6228.98	10740.66	4008.85	486	
H94B	6154.48	11619.02	4350.59	486	
H94C	7221.34	11492.25	4350.59	486	
H78A	9044.68	3947.21	825.04	534	
H78B	8095.55	3420.46	1114.05	534	
H78C	8427.15	4493.19	1114.05	534	
H79A	8220.79	3357.59	4282.33	405	
H79B	8891.9	3295.75	4839.94	405	
H79C	8326.89	4015.61	4839.94	405	
H40D	2395.49	3768.32	3203.21	358	
H40E	2299.22	3242.33	2596.66	358	
H40F	3319.96	4047.26	2854.57	358	
H40A	2495.28	4420.03	2855	358	
H40B	2078.14	3409.37	3012.21	358	
H40C	3258.61	3902	3012.21	358	
H81A	3156.29	9928.3	3192.97	312	
H81B	4043.85	10781.25	3051.41	312	
H81C	3368.57	10008.46	2561.79	312	
H81D	3877.23	10148.52	3355.14	312	
H81E	3915.34	10745.69	2850.35	312	
H81F	2945.33	9870.57	2864.03	312	


Table 0: Atomic Occupancies for all atoms that are not fully occupied in mjp184_150k_new.


Atom	Occupancy	
H17	0.5	
H17A	0.5	
H20A	0.5	
H20	0.5	
H24	0.5	
H24A	0.5	
H27	0.5	
H27A	0.5	
H29	0.5	
H29A	0.5	
C30	0.5	
H30A	0.5	
H30B	0.5	
H30C	0.5	
C28	0.5	
C41	0.5	
H41A	0.5	
H41B	0.5	
H41C	0.5	
C43	0.5	
H43A	0.5	
H43B	0.5	
C44	0.5	
H44A	0.5	
H44B	0.5	
H44C	0.5	
C51	0.5	
H51A	0.5	
H51B	0.5	
C53	0.5	
H53A	0.5	
H53B	0.5	
C56	0.5	
H56A	0.5	
H56B	0.5	
C57	0.5	
C59	0.5	
H59A	0.5	
H59B	0.5	
C60	0.5	
H60A	0.5	
H60B	0.5	
H60C	0.5	
C61	0.5	
H61A	0.5	
H61B	0.5	
H61C	0.5	
C63	0.5	
H63A	0.5	
H63B	0.5	
H63C	0.5	
C64	0.5	
H64A	0.5	
H64B	0.5	
C66	0.5	
H66A	0.5	
H66B	0.5	
H66C	0.5	
C65	0.5	
H65A	0.5	
H65B	0.5	
C67	0.5	
C68	0.5	
C69	0.5	
H69A	0.5	
H69B	0.5	
C70	0.5	
H70A	0.5	
H70B	0.5	
H70C	0.5	
C86	0.5	
H86A	0.5	
H86B	0.5	
H86C	0.5	
C119	0.5	
H11A	0.5	
H11B	0.5	
H11C	0.5	
H76A	0.5	
H76B	0.5	
H76C	0.5	
H76D	0.5	
H76E	0.5	
H76F	0.5	
H40D	0.5	
H40E	0.5	
H40F	0.5	
H40A	0.5	
H40B	0.5	
H40C	0.5	
H81A	0.5	
H81B	0.5	
H81C	0.5	
H81D	0.5	
H81E	0.5	
H81F	0.5	


Citations
O.V. Dolomanov and L.J. Bourhis and R.J. Gildea and J.A.K. Howard and H. Puschmann, Olex2: A complete structure solution, refinement and analysis program, J. Appl. Cryst., (2009), 42, 339-341.
Sheldrick, G.M., Crystal structure refinement with ShelXL, Acta Cryst., (2015), C71, 3-8.
Sheldrick, G.M., ShelXT-Integrated space-group and crystal-structure determination, Acta Cryst., (2015), A71, 3-8.
X-Area Integrate 1.78.3.0
X-Area Pilatus3_SV 1.31.170.0 (STOE, 2020)
X-Area Recipe 1.36.0.0
