# Supplementary material for: Mechanism‐Guided Precision Hydrolysis of Early Transition Metals to Access (Mixed‐Metal) Oxo Clusters
Source: Angew Chem Int Ed Engl. 2026 Feb 24;65(15):e25769. doi: 10.1002/anie.202525769 (PMC13053926; doi:10.1002/anie.202525769)

# checkCIF/PLATON report

Structure factors have been supplied for datablock(s) mjp184_150k_new

THIS REPORT IS FOR GUIDANCE ONLY. IF USED AS PART OF A REVIEW PROCEDURE FOR PUBLICATION, IT SHOULD NOT REPLACE THE EXPERTISE OF AN EXPERIENCED CRYSTALLOGRAPHIC REFEREE.

No syntax errors found. CIF dictionary Interpreting this report

## Datablock: mjp184_150k_new

---

```
Bond precision:   C-C = 0.0368 A          Wavelength=1.54186

Cell:           a=14.2648(5)      b=15.8095(5)      c=24.2737(7)
                alpha=94.600(2)   beta=96.227(3)    gamma=109.628(2)
Temperature:    150 K
```

| | Calculated | Reported |
|---|---|---|
| Volume | 5085.8(3) | 5085.8(3) |
| Space group | P -1 | P -1 |
| Hall group | -P 1 | -P 1 |
| Moiety formula | C55 H97 O33 Zr6, 3(C5 H10 O2), C4 H9 | C59 H106 O33 Zr6, 3(C5 H10 O2) |
| Sum formula | C74 H136 O39 Zr6 | C74 H136 O39 Zr6 |
| Mr | 2197.15 | 2197.14 |
| Dx,g cm-3 | 1.435 | 1.435 |
| Z | 2 | 2 |
| Mu (mm-1) | 5.521 | 5.521 |
| F000 | 2264.0 | 2264.0 |
| F000’ | 2267.93 | |
| h,k,lmax | 17,19,30 | 17,19,30 |
| Nref | 21011 | 20361 |
| Tmin,Tmax | 0.267,0.331 | 0.007,0.041 |
| Tmin’ | 0.153 | |

```
Correction method= # Reported T Limits: Tmin=0.007 Tmax=0.041
AbsCorr = MULTI-SCAN
```

Data completeness= 0.969          Theta(max)= 75.230

R(reflections)= 0.1064( 10458)          wR2(reflections)= 0.2863( 20361)

S = 1.021          Npar= 775

---

The following ALERTS were generated. Each ALERT has the format
**test-name_ALERT_alert-type_alert-level**.
Click on the hyperlinks for more details of the test.

---

**Alert level B**

PLAT230_ALERT_2_B Hirshfeld Test Diff for O5 --C3 . 11.2 s.u.

**Author Response: These alerts are due to O atoms strongly bonded to a Zr atom or H-bonding and their neighouring C atom that is part of either a disordered or highly vibrating ligand.**

PLAT230_ALERT_2_B Hirshfeld Test Diff for O6 --C4 . 7.5 s.u.

**Author Response: These alerts are due to O atoms strongly bonded to a Zr atom or H-bonding and their neighouring C atom that is part of either a disordered or highly vibrating ligand.**

PLAT230_ALERT_2_B Hirshfeld Test Diff for O10 --C37 . 8.9 s.u.

**Author Response: These alerts are due to O atoms strongly bonded to a Zr atom or H-bonding and their neighouring C atom that is part of either a disordered or highly vibrating ligand.**

PLAT230_ALERT_2_B Hirshfeld Test Diff for O11 --C3 . 9.6 s.u.

**Author Response: These alerts are due to O atoms strongly bonded to a Zr atom or H-bonding and their neighouring C atom that is part of either a disordered or highly vibrating ligand.**

PLAT230_ALERT_2_B Hirshfeld Test Diff for O12 --C7 . 8.2 s.u.

**Author Response: These alerts are due to O atoms strongly bonded to a Zr atom or H-bonding and their neighouring C atom that is part of either a disordered or highly vibrating ligand.**

```
PLAT230_ALERT_2_B Hirshfeld Test Diff for    O18      --C4       .        9.2 s.u.
```

**Author Response: These alerts are due to O atoms strongly bonded to a Zr atom or H-bonding and their neighouring C atom that is part of either a disordered or highly vibrating ligand.**

```
PLAT230_ALERT_2_B Hirshfeld Test Diff for    O21      --C5       .       13.5 s.u.
```

**Author Response: These alerts are due to O atoms strongly bonded to a Zr atom or H-bonding and their neighouring C atom that is part of either a disordered or highly vibrating ligand.**

```
PLAT230_ALERT_2_B Hirshfeld Test Diff for    O24      --C6       .        7.3 s.u.
```

**Author Response: These alerts are due to O atoms strongly bonded to a Zr atom or H-bonding and their neighouring C atom that is part of either a disordered or highly vibrating ligand.**

```
PLAT230_ALERT_2_B Hirshfeld Test Diff for    O26      --C5       .       13.8 s.u.
```

**Author Response: These alerts are due to O atoms strongly bonded to a Zr atom or H-bonding and their neighouring C atom that is part of either a disordered or highly vibrating ligand.**

```
PLAT230_ALERT_2_B Hirshfeld Test Diff for    O27      --C37      .       10.3 s.u.
```

**Author Response: These alerts are due to O atoms strongly bonded to a Zr atom or H-bonding and their neighouring C atom that is part of either a disordered or highly vibrating ligand.**

```
PLAT230_ALERT_2_B Hirshfeld Test Diff for    O29      --C7       .       12.0 s.u.
```

**Author Response: These alerts are due to O atoms strongly bonded to a Zr atom or H-bonding and their neighouring C atom that is part of either a disordered or highly vibrating ligand.**

```
PLAT230_ALERT_2_B Hirshfeld Test Diff for    O31      --C10      .       10.8 s.u.
```

**Author Response: These alerts are due to O atoms strongly bonded to a Zr atom or H-bonding and their neighouring C atom that is part of either a disordered or highly vibrating ligand.**

PLAT241_ALERT_2_B High 'MainMol' Ueq as Compared to Neighbors of C1 Check

**Author Response: These alerts are due to O atoms strongly bonded to a Zr atom or H-bonding and their neighouring C atom that is part of either a disordered or highly vibrating ligand.**

PLAT241_ALERT_2_B High 'MainMol' Ueq as Compared to Neighbors of C3 Check

**Author Response: These alerts are due to O atoms strongly bonded to a Zr atom or H-bonding and their neighouring C atom that is part of either a disordered or highly vibrating ligand.**

PLAT241_ALERT_2_B High 'MainMol' Ueq as Compared to Neighbors of C4 Check

**Author Response: These alerts are due to O atoms strongly bonded to a Zr atom or H-bonding and their neighouring C atom that is part of either a disordered or highly vibrating ligand.**

PLAT241_ALERT_2_B High 'MainMol' Ueq as Compared to Neighbors of C5 Check

**Author Response: These alerts are due to O atoms strongly bonded to a Zr atom or H-bonding and their neighouring C atom that is part of either a disordered or highly vibrating ligand.**

PLAT241_ALERT_2_B High 'MainMol' Ueq as Compared to Neighbors of C6 Check

**Author Response: These alerts are due to O atoms strongly bonded to a Zr atom or H-bonding and their neighouring C atom that is part of either a disordered or highly vibrating ligand.**

PLAT241_ALERT_2_B High 'MainMol' Ueq as Compared to Neighbors of C7 Check

**Author Response: These alerts are due to O atoms strongly bonded to a Zr atom or H-bonding and their neighouring C atom that is part of either a disordered or highly vibrating ligand.**

PLAT241_ALERT_2_B High 'MainMol' Ueq as Compared to Neighbors of C10 Check

**Author Response: These alerts are due to O atoms strongly bonded to a Zr atom or H-bonding and their neighouring C atom that is part of either a disordered or highly vibrating ligand.**

```
PLAT241_ALERT_2_B High   'MainMol' Ueq as Compared to Neighbors of        C11 Check
```

**Author Response: These alerts are due to O atoms strongly bonded to a Zr atom or H-bonding and their neighouring C atom that is part of either a disordered or highly vibrating ligand.**

```
PLAT241_ALERT_2_B High   'MainMol' Ueq as Compared to Neighbors of        C37 Check
```

**Author Response: These alerts are due to O atoms strongly bonded to a Zr atom or H-bonding and their neighouring C atom that is part of either a disordered or highly vibrating ligand.**

```
PLAT242_ALERT_2_B Low    'MainMol' Ueq as Compared to Neighbors of         O5 Check
```

**Author Response: These alerts are due to O atoms strongly bonded to a Zr atom or H-bonding and their neighouring C atom that is part of either a disordered or highly vibrating ligand.**

```
PLAT242_ALERT_2_B Low    'MainMol' Ueq as Compared to Neighbors of         O6 Check
```

**Author Response: These alerts are due to O atoms strongly bonded to a Zr atom or H-bonding and their neighouring C atom that is part of either a disordered or highly vibrating ligand.**

```
PLAT242_ALERT_2_B Low    'MainMol' Ueq as Compared to Neighbors of         O9 Check
```

**Author Response: These alerts are due to O atoms strongly bonded to a Zr atom or H-bonding and their neighouring C atom that is part of either a disordered or highly vibrating ligand.**

```
PLAT242_ALERT_2_B Low    'MainMol' Ueq as Compared to Neighbors of        O11 Check
```

**Author Response: These alerts are due to O atoms strongly bonded to a Zr atom or H-bonding and their neighouring C atom that is part of either a disordered or highly vibrating ligand.**

```
PLAT242_ALERT_2_B Low    'MainMol' Ueq as Compared to Neighbors of        O12 Check
```

**Author Response: These alerts are due to O atoms strongly bonded to a Zr atom or H-bonding and their neighouring C atom that is part of either a disordered or highly vibrating ligand.**

```
PLAT242_ALERT_2_B Low    'MainMol' Ueq as Compared to Neighbors of        O18 Check
```

**Author Response: These alerts are due to O atoms strongly bonded to a Zr atom or H-bonding and their neighouring C atom that is part of either a disordered or highly vibrating ligand.**

```
PLAT242_ALERT_2_B Low    'MainMol' Ueq as Compared to Neighbors of        O26 Check
```

**Author Response: These alerts are due to O atoms strongly bonded to a Zr atom or H-bonding and their neighouring C atom that is part of either a disordered or highly vibrating ligand.**

```
PLAT242_ALERT_2_B Low    'MainMol' Ueq as Compared to Neighbors of        O29 Check
```

**Author Response: These alerts are due to O atoms strongly bonded to a Zr atom or H-bonding and their neighouring C atom that is part of either a disordered or highly vibrating ligand.**

```
PLAT260_ALERT_2_B Large Average Ueq of Residue Including       O35      0.309 Check
```

**Author Response: in this case the whole ligand is NOT bonded to the metal so it has the possibility to vibrate in its entirety.**

```
PLAT342_ALERT_3_B Low Bond Precision on  C-C Bonds ..............    0.03682 Ang.
```

**Author Response: not surprised given the huge amount of disorder**

```
PLAT910_ALERT_3_B Missing FCF Reflection(s) Below Theta(Min)[Deg]=       5.04 Note
                1  0  0,  -1  1  0,   0  1  0,   0 -1  1,   1 -1  1,  -1  0  1,
                0  0  1,   1  0  1,  -1  1  1,   0  1  1,   0 -1  2,  -1  0  2,
                0  0  2,
```

**Author Response: it is very likely they were either outliers or shadowed by the beam-stop.**

---

## Alert level C

```
PLAT042_ALERT_1_C Calc. and Reported MoietyFormula Strings  Differ     Please Check
              Calc: C55 H97 O33 Zr6, 3(C5 H10 O2), C4 H9
              Rep.: C59 H106 O33 Zr6, 3(C5 H10 O2)
PLAT082_ALERT_2_C High R1 Value ................................       0.11 Report
PLAT084_ALERT_3_C High wR2 Value (i.e. > 0.25) ..................       0.29 Report
PLAT202_ALERT_3_C Isotropic non-H Atoms in Anion/Solvent .........          9 Check
              C16     C22     C26     C79     C80     C34     C39     C54
              C75
PLAT230_ALERT_2_C Hirshfeld Test Diff for    O7       --C2       .        5.9 s.u.
```

**Author Response: These alerts are due to O atoms strongly bonded to a Zr atom or H-bonding and their neighouring C atom that is part of either a disordered or highly vibrating ligand.**

```
PLAT230_ALERT_2_C Hirshfeld Test Diff for    O9       --C1       .        5.2 s.u.
```

**Author Response: These alerts are due to O atoms strongly bonded to a Zr atom or H-bonding and their neighouring C atom that is part of either a disordered or highly vibrating ligand.**

```
PLAT230_ALERT_2_C Hirshfeld Test Diff for    O14      --C6       .        6.9 s.u.
```

**Author Response: These alerts are due to O atoms strongly bonded to a Zr atom or H-bonding and their neighouring C atom that is part of either a disordered or highly vibrating ligand.**

```
PLAT230_ALERT_2_C Hirshfeld Test Diff for    O20      --C11      .        5.4 s.u.
```

**Author Response: These alerts are due to O atoms strongly bonded to a Zr atom or H-bonding and their neighouring C atom that is part of either a disordered or highly vibrating ligand.**

```
PLAT230_ALERT_2_C Hirshfeld Test Diff for    O23      --C10      .        6.3 s.u.
```

**Author Response: These alerts are due to O atoms strongly bonded to a Zr atom or H-bonding and their neighouring C atom that is part of either a disordered or highly vibrating ligand.**

```
PLAT230_ALERT_2_C Hirshfeld Test Diff for    O25      --C8       .        6.0 s.u.
```

**Author Response: These alerts are due to O atoms strongly bonded to a Zr atom or H-bonding and their neighouring C atom that is part of either a disordered or highly vibrating ligand.**

```
PLAT230_ALERT_2_C Hirshfeld Test Diff for    O30      --C1       .        5.2 s.u.
```

**Author Response: These alerts are due to O atoms strongly bonded to a Zr atom or H-bonding and their neighouring C atom that is part of either a disordered or highly vibrating ligand.**

```
PLAT234_ALERT_4_C Large Hirshfeld Difference Zr3      --O5       .       0.17 Ang.
PLAT234_ALERT_4_C Large Hirshfeld Difference Zr5      --O11      .       0.18 Ang.
PLAT234_ALERT_4_C Large Hirshfeld Difference O2       --C2       .       0.19 Ang.
PLAT234_ALERT_4_C Large Hirshfeld Difference O22      --C8       .       0.25 Ang.
PLAT234_ALERT_4_C Large Hirshfeld Difference O28      --C11      .       0.19 Ang.
PLAT241_ALERT_2_C High   'MainMol' Ueq as Compared to Neighbors of         C2 Check
```

**Author Response: These alerts are due to O atoms strongly bonded to a Zr atom or H-bonding and their neighouring C atom that is part of either a disordered or highly vibrating ligand.**

```
PLAT241_ALERT_2_C High   'MainMol' Ueq as Compared to Neighbors of         C8 Check
```

**Author Response: These alerts are due to O atoms strongly bonded to a Zr atom or H-bonding and their neighouring C atom that is part of either a disordered or highly vibrating ligand.**

```
PLAT242_ALERT_2_C Low    'MainMol' Ueq as Compared to Neighbors of         O2 Check
```

**Author Response: These alerts are due to O atoms strongly bonded to a Zr atom or H-bonding and their neighouring C atom that is part of either a disordered or highly vibrating ligand.**

```
PLAT242_ALERT_2_C Low    'MainMol' Ueq as Compared to Neighbors of         O7 Check
```

**Author Response: These alerts are due to O atoms strongly bonded to a Zr atom or H-bonding and their neighouring C atom that is part of either a disordered or highly vibrating ligand.**

```
PLAT242_ALERT_2_C Low    'MainMol' Ueq as Compared to Neighbors of        O10 Check
```

**Author Response: These alerts are due to O atoms strongly bonded to a Zr atom or H-bonding and their neighouring C atom that is part of either a disordered or highly vibrating ligand.**

```
PLAT242_ALERT_2_C Low    'MainMol' Ueq as Compared to Neighbors of        O14 Check
```

**Author Response: These alerts are due to O atoms strongly bonded to a Zr atom or H-bonding and their neighouring C atom that is part of either a disordered or highly vibrating ligand.**

```
PLAT242_ALERT_2_C Low    'MainMol' Ueq as Compared to Neighbors of        O20 Check
```

**Author Response: These alerts are due to O atoms strongly bonded to a Zr atom or H-bonding and their neighouring C atom that is part of either a disordered or highly vibrating ligand.**

```
PLAT242_ALERT_2_C Low    'MainMol' Ueq as Compared to Neighbors of        O21 Check
```

**Author Response: These alerts are due to O atoms strongly bonded to a Zr atom or H-bonding and their neighouring C atom that is part of either a disordered or highly vibrating ligand.**

```
PLAT242_ALERT_2_C Low    'MainMol' Ueq as Compared to Neighbors of        O23 Check
```

**Author Response: These alerts are due to O atoms strongly bonded to a Zr atom or H-bonding and their neighouring C atom that is part of either a disordered or highly vibrating ligand.**

```
PLAT242_ALERT_2_C Low    'MainMol' Ueq as Compared to Neighbors of        O24 Check
```

**Author Response: These alerts are due to O atoms strongly bonded to a Zr atom or H-bonding and their neighouring C atom that is part of either a disordered or highly vibrating ligand.**

```
PLAT242_ALERT_2_C Low    'MainMol' Ueq as Compared to Neighbors of        O27 Check
```

**Author Response: These alerts are due to O atoms strongly bonded to a Zr atom or H-bonding and their neighouring C atom that is part of either a disordered or highly vibrating ligand.**

```
PLAT242_ALERT_2_C Low    'MainMol' Ueq as Compared to Neighbors of        O28 Check
```

**Author Response: These alerts are due to O atoms strongly bonded to a Zr atom or H-bonding and their neighouring C atom that is part of either a disordered or highly vibrating ligand.**

```
PLAT242_ALERT_2_C Low    'MainMol' Ueq as Compared to Neighbors of        O31 Check
```

**Author Response: These alerts are due to O atoms strongly bonded to a Zr atom or H-bonding and their neighouring C atom that is part of either a disordered or highly vibrating ligand.**

```
PLAT242_ALERT_2_C Low    'MainMol' Ueq as Compared to Neighbors of        C29 Check
```

**Author Response: These alerts are due to O atoms strongly bonded to a Zr atom or H-bonding and their neighouring C atom that is part of either a disordered or highly vibrating ligand.**

```
PLAT243_ALERT_4_C High   'Solvent' Ueq as Compared to Neighbors of        C15 Check
PLAT243_ALERT_4_C High   'Solvent' Ueq as Compared to Neighbors of         C9 Check
PLAT250_ALERT_2_C Large U3/U1 Ratio for <U(i,j)> Tensor(Resd    2)        2.8 Note
PLAT250_ALERT_2_C Large U3/U1 Ratio for <U(i,j)> Tensor(Resd    3)        3.6 Note
PLAT260_ALERT_2_C Large Average Ueq of Residue Including       Zr1      0.194 Check
```

**Author Response: in this case the whole ligand is NOT bonded to the metal so it has the possibility to vibrate in its entirety.**

```
PLAT260_ALERT_2_C Large Average Ueq of Residue Including       O34      0.295 Check
```

**Author Response: in this case the whole ligand is NOT bonded to the metal so it has the possibility to vibrate in its entirety.**

```
PLAT260_ALERT_2_C Large Average Ueq of Residue Including       O36      0.283 Check
```

**Author Response: in this case the whole ligand is NOT bonded to the metal so it has the possibility to vibrate in its entirety.**

```
PLAT362_ALERT_2_C Short  C(sp3)-C(sp2) Bond  C1       - C14      .       1.37 Ang.
PLAT410_ALERT_2_C Short Intra H...H Contact  H12      ..H35A     .       1.95 Ang.
                                                      x,y,z  =      1_555 Check
PLAT410_ALERT_2_C Short Intra H...H Contact  H34      ..H54B     .       1.92 Ang.
                                                      x,y,z  =      1_555 Check
PLAT412_ALERT_2_C Short Intra XH3 .. XHn     H52B     ..H58B     .       1.86 Ang.
                                                      x,y,z  =      1_555 Check
PLAT412_ALERT_2_C Short Intra XH3 .. XHn     H79B     ..H80B     .       1.89 Ang.
                                                      x,y,z  =      1_555 Check
PLAT413_ALERT_2_C Short Inter XH3 .. XHn     H12C     ..H38C     .       2.02 Ang.
                                                    x,1+y,z  =      1_565 Check
PLAT601_ALERT_2_C Unit-Cell Contains Solvent Accessible VOIDS .LE.         50 Ang**3
PLAT906_ALERT_3_C Large K Value in the Analysis of Variance ......      8.177 Check
PLAT906_ALERT_3_C Large K Value in the Analysis of Variance ......      2.386 Check
PLAT911_ALERT_3_C Missing FCF Refl Between Thmin & STh/L=    0.600        259 Report
                2  0  1,   1  1  1,   3  1  1,   2  2  1,   1 -1  2,   1  0  2,
                2  0  2,   3  0  2,  -3  3  2,   0  4  2,   0 -4  3,   1 -4  3,
               -1 -3  3,   0 -1  3,   1 -1  3,   3 -1  3,  -1  0  3,   5 12  3,
               -2 -2  4,  -1 -1  4,   0 -1  4,   0 -5  5,   1 -5  5,  -1 -3  5,
               -1 -2  5,   0 -1  5,   1  0  5,   1  1  5,   0  2  5,   0 -5  6,
                  (  229 More Missing: see the .ckf listing file)
PLAT913_ALERT_3_C Missing # of Very Strong Reflections in FCF ....          6 Note
               -1  1  0,   0 -1  1,  -1  0  1,   1  0  1,   0  1  1,   0  0  2,
PLAT918_ALERT_3_C Reflection(s) with I(obs) much Smaller I(calc) .          1 Check
                2 -3  5,
PLAT977_ALERT_2_C Check Negative Difference Density on H12A      .      -0.44 eA-3
PLAT977_ALERT_2_C Check Negative Difference Density on H33B      .      -0.36 eA-3
PLAT977_ALERT_2_C Check Negative Difference Density on H40A      .      -0.34 eA-3
```

```
PLAT977_ALERT_2_C Check Negative Difference Density on H58B      .      -0.31 eA-3
```

---

### Alert level G

```
PLAT002_ALERT_2_G Number of Distance or Angle Restraints on AtSite         82 Note
PLAT003_ALERT_2_G Number of Uiso or U(i,j)  Restrained non-H-Atoms         75 Report
PLAT007_ALERT_5_G Number of Unrefined Donor-H Atoms .............           9 Report
              H3      H4      H8      H15     H16A    H16B    H39     H40
              H37
PLAT083_ALERT_2_G SHELXL Second Parameter in WGHT  Unusually Large      25.00 Why ?
PLAT171_ALERT_4_G The CIF-Embedded .res File Contains EADP Records         14 Report
PLAT172_ALERT_4_G The CIF-Embedded .res File Contains DFIX Records         66 Report
PLAT173_ALERT_4_G The CIF-Embedded .res File Contains DANG Records         29 Report
PLAT176_ALERT_4_G The CIF-Embedded .res File Contains SADI Records          2 Report
PLAT178_ALERT_4_G The CIF-Embedded .res File Contains SIMU Records         20 Report
PLAT186_ALERT_4_G The CIF-Embedded .res File Contains ISOR Records         15 Report
PLAT187_ALERT_4_G The CIF-Embedded .res File Contains RIGU Records         20 Report
PLAT231_ALERT_4_G Hirshfeld Test (Solvent)   O35      --C15      .        5.9 s.u.
PLAT299_ALERT_4_G Atom Site Occupancy Constrained at ............         0.5 Check
              C28     C30     C41     C43     C44     C51     C53     C57
              C63     C64     C65     C66     C67     C68     C69     C70
              H17     H17A    H20     H20A    H27     H27A    H29     H29A
              H30A    H30B    H30C    H40A    H40B    H40C    H40D    H40E
              H40F    H41A    H41B    H41C    H43A    H43B    H44A    H44B
              H44C    H51A    H51B    H53A    H53B    H63A    H63B    H63C
              H64A    H64B    H65A    H65B    H66A    H66B    H66C    H69A
              H69B    H70A    H70B    H70C    H76A    H76B    H76C    H76D
              H76E    H76F    H81A    H81B    H81C    H81D    H81E    H81F
              C56     C59     C60     C61     C86     C119    H11A    H11B
              H11C    H24     H24A    H56A    H56B    H59A    H59B    H60A
              H60B    H60C    H61A    H61B    H61C    H86A    H86B    H86C
PLAT301_ALERT_3_G Main Residue  Disorder .............(Resd    1)          9% Note
PLAT302_ALERT_4_G Anion/Solvent/Minor-Residue Disorder (Resd    2)         43% Note
PLAT315_ALERT_2_G Singly Bonded Carbon Detected (H-atoms Missing).        C28 Check
PLAT315_ALERT_2_G Singly Bonded Carbon Detected (H-atoms Missing).        C57 Check
PLAT315_ALERT_2_G Singly Bonded Carbon Detected (H-atoms Missing).        C67 Check
PLAT315_ALERT_2_G Singly Bonded Carbon Detected (H-atoms Missing).        C68 Check
PLAT343_ALERT_2_G Unusual sp?      Angle Range in Main Residue for         C8 Check
PLAT343_ALERT_2_G Unusual sp3      Angle Range in Main Residue for        C12 Check
PLAT343_ALERT_2_G Unusual sp3      Angle Range in Main Residue for        C19 Check
PLAT343_ALERT_2_G Unusual sp3      Angle Range in Main Residue for        C35 Check
PLAT343_ALERT_2_G Unusual sp3      Angle Range in Main Residue for        C42 Check
PLAT344_ALERT_2_G Unusual sp3       Angle Range in Solvent/Ion for        C73 Check
PLAT344_ALERT_2_G Unusual sp3       Angle Range in Solvent/Ion for        C94 Check
PLAT344_ALERT_2_G Unusual sp3       Angle Range in Solvent/Ion for        C16 Check
PLAT344_ALERT_2_G Unusual sp3       Angle Range in Solvent/Ion for        C79 Check
PLAT344_ALERT_2_G Unusual sp?       Angle Range in Solvent/Ion for        C34 Check
PLAT413_ALERT_2_G Short Inter XH3 .. XHn     H46B     ..H86B     .       2.14 Ang.
                                                   1+x,y,z  =     1_655 Check
PLAT413_ALERT_2_G Short Inter XH3 .. XHn     H66C     ..H74C     .       2.06 Ang.
                                                   x,1+y,z  =     1_565 Check
PLAT413_ALERT_2_G Short Inter XH3 .. XHn     H74B     ..H86C     .       2.04 Ang.
                                                  x,-1+y,z  =     1_545 Check
PLAT413_ALERT_2_G Short Inter XH3 .. XHn     H11B     ..H74B     .       1.62 Ang.
                                                   x,1+y,z  =     1_565 Check
PLAT432_ALERT_2_G Short Inter X...Y Contact  O22      ..C34      .       2.60 Ang.
                                                     x,y,z  =     1_555 Check
```

```
PLAT432_ALERT_2_G Short Inter X...Y Contact  O22      ..C39      .       2.79 Ang.
                                                     x,y,z  =     1_555 Check
PLAT432_ALERT_2_G Short Inter X...Y Contact  O25      ..C34      .       2.59 Ang.
                                                     x,y,z  =     1_555 Check
PLAT432_ALERT_2_G Short Inter X...Y Contact  O25      ..C54      .       2.84 Ang.
                                                     x,y,z  =     1_555 Check
PLAT432_ALERT_2_G Short Inter X...Y Contact  C8       ..C34      .       1.77 Ang.
                                                     x,y,z  =     1_555 Check
PLAT432_ALERT_2_G Short Inter X...Y Contact  C8       ..C54      .       2.48 Ang.
                                                     x,y,z  =     1_555 Check
PLAT432_ALERT_2_G Short Inter X...Y Contact  C8       ..C39      .       2.57 Ang.
                                                     x,y,z  =     1_555 Check
PLAT432_ALERT_2_G Short Inter X...Y Contact  C38      ..C122     .       3.14 Ang.
                                                  x,-1+y,z  =     1_545 Check
PLAT432_ALERT_2_G Short Inter X...Y Contact  C74      ..C119     .       3.20 Ang.
                                                  x,-1+y,z  =     1_545 Check
PLAT721_ALERT_1_G Bond    Calc     0.97000, Rep     0.98010 Dev...       0.01 Ang.
              C41      -H41A           1_555   1_555 ........     #  191 Check
PLAT722_ALERT_1_G Angle   Calc      119.00, Rep      117.90 Dev...       1.10 Degree
              C24     -C61     -H61C       1_555  1_555  1_555     #  763 Check
PLAT773_ALERT_2_G Check long C-C Bond in CIF: C8       --C34             1.77 Ang.
PLAT793_ALERT_4_G Model has Chirality at C12        (Centro SpGr)          S Verify
PLAT793_ALERT_4_G Model has Chirality at C14        (Centro SpGr)          S Verify
PLAT793_ALERT_4_G Model has Chirality at C16        (Centro SpGr)          S Verify
PLAT793_ALERT_4_G Model has Chirality at C18        (Centro SpGr)          S Verify
PLAT793_ALERT_4_G Model has Chirality at C19        (Centro SpGr)          S Verify
PLAT793_ALERT_4_G Model has Chirality at C21        (Centro SpGr)          S Verify
PLAT793_ALERT_4_G Model has Chirality at C25        (Centro SpGr)          S Verify
PLAT793_ALERT_4_G Model has Chirality at C31        (Centro SpGr)          R Verify
PLAT793_ALERT_4_G Model has Chirality at C71        (Centro SpGr)          S Verify
PLAT794_ALERT_5_G Tentative Bond Valency for Zr1       (IV)      .      4.11 Info
PLAT794_ALERT_5_G Tentative Bond Valency for Zr2       (IV)      .      4.31 Info
PLAT794_ALERT_5_G Tentative Bond Valency for Zr3       (IV)      .      4.35 Info
PLAT794_ALERT_5_G Tentative Bond Valency for Zr4       (IV)      .      4.32 Info
PLAT794_ALERT_5_G Tentative Bond Valency for Zr5       (IV)      .      4.03 Info
PLAT794_ALERT_5_G Tentative Bond Valency for Zr6       (IV)      .      3.98 Info
PLAT802_ALERT_4_G CIF Input Record(s) with more than 80 Characters          2 Info
PLAT860_ALERT_3_G Number of Least-Squares Restraints ............         998 Note
PLAT883_ALERT_1_G Absent Datum for _atom_sites_solution_primary ..     Please Do !
PLAT912_ALERT_4_G Missing # of FCF Reflections Above STh/L=  0.600        377 Note
PLAT933_ALERT_2_G Number of HKL-OMIT Records in Embedded .res File         11 Note
               -1 -1  4,  -1  0  3,   0 -1  2,   0  0 11,   0  2  5,   1 -4  3,
                1  0  5,   1  1  1,   1  1  5,   2  2  1,   3 -1  3,
PLAT941_ALERT_3_G Average HKL Measurement Multiplicity ..........         3.8 Low
PLAT969_ALERT_5_G The 'Henn et al.' R-Factor-gap value ..........       5.860 Note
              Predicted wR2: Based on SigI**2  4.89 or SHELX Weight 28.04
PLAT978_ALERT_2_G Number C-C Bonds with Positive Residual Density.          0 Info
```

---

0 **ALERT level A** = Most likely a serious problem - resolve or explain
32 **ALERT level B** = A potentially serious problem, consider carefully
53 **ALERT level C** = Check. Ensure it is not caused by an omission or oversight
68 **ALERT level G** = General information/check it is not something unexpected

4 ALERT type 1 CIF construction/syntax error, inconsistent or missing data

```
101 ALERT type 2 Indicator that the structure model may be wrong or deficient
 12 ALERT type 3 Indicator that the structure quality may be low
 28 ALERT type 4 Improvement, methodology, query or suggestion
  8 ALERT type 5 Informative message, check
```

---

---

It is advisable to attempt to resolve as many as possible of the alerts in all categories. Often the minor alerts point to easily fixed oversights, errors and omissions in your CIF or refinement strategy, so attention to these fine details can be worthwhile. It is up to the individual to critically assess their own results and, if necessary, seek expert advice.

---

## PLATON version of 26/09/2025; check.def file version of 20/09/2025

**Datablock mjp184_150k_new** - ellipsoid plot

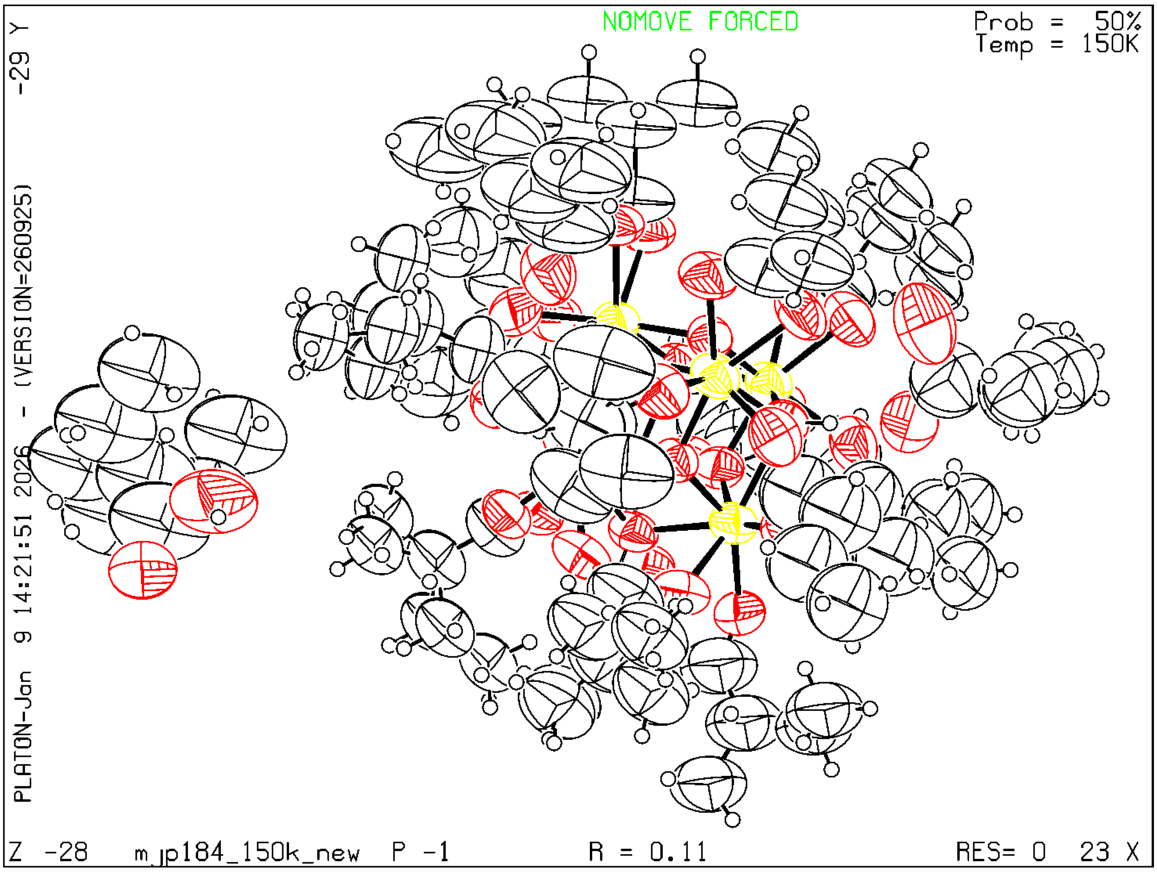

Supplement: Supplementary file 2 — Supporting File 2: anie71298–sup–0002–Data.zip. [file ANIE-65-e25769-s002.zip › CCDC_2495248/mjp184_150k_new_cifreport.pdf]
