# Supplementary material for: Mechanism‐Guided Precision Hydrolysis of Early Transition Metals to Access (Mixed‐Metal) Oxo Clusters
Source: Angew Chem Int Ed Engl. 2026 Feb 24;65(15):e25769. doi: 10.1002/anie.202525769 (PMC13053926; doi:10.1002/anie.202525769)

checkCIF/PLATON report

Structure factors have been supplied for datablock(s) mjp104-3_150k

THIS REPORT IS FOR GUIDANCE ONLY. IF USED AS PART OF A REVIEW PROCEDURE FOR PUBLICATION, IT SHOULD NOT REPLACE THE EXPERTISE OF AN EXPERIENCED CRYSTALLOGRAPHIC REFEREE.

No syntax errors found. CIF dictionary Interpreting this report

Datablock: mjp104-3_150k

Bond precision:	C-C = 0.0157 A		Wavelength=1.34143
Cell:	a=42.5266(5)	b=10.7503(2)	c=42.4305(6)
	alpha=90	beta=89.978(1)	gamma=90
Temperature:	150 K		

	Calculated	Reported
Volume	19398.1(5)	19398.1(5)
Space group	I 2/a	I 1 2/a 1
Hall group	-I 2ya	-I 2ya
Moiety formula	C81.96 H79.90 Nb8 O38, 0.8(C2 H3 N)	2(C40.98 H39.95 Nb4 O19), 0.8(C2 H3 N)
Sum formula	C83.56 H82.30 N0.80 Nb8 O38	C83.56 H82.30 N0.80 Nb8 O38
Mr	2449.01	2449.00
Dx,g cm-3	1.677	1.677
Z	8	8
Mu (mm-1)	5.494	5.494
F000	9770.1	9770.0
F000’	9770.30	
h,k,lmax		51,13,52
Nref		18778
Tmin,Tmax	0.343,0.372	0.067,0.245
Tmin’	0.260	

Correction method= # Reported T Limits: Tmin=0.067 Tmax=0.245
AbsCorr = MULTI-SCAN

Data completeness= Theta(max)= 55.763

R(reflections)= 0.0684(15471) wR2(reflections)= 0.1991(18778)

S = 1.061 Npar= 1241

The following ALERTS were generated. Each ALERT has the format
test-name_ALERT_alert-type_alert-level.
Click on the hyperlinks for more details of the test.

Alert level B

```
PLAT201_ALERT_2_B Isotropic non-H Atoms in Main Residue(s) .......          6 Report
              C101    C102    C103    C104    C105    C106
```

Author Response: these belong to very disordered aromatic rings that could be refined anisotropically

```
PLAT601_ALERT_2_B Unit Cell Contains Solvent Accessible VOIDS of .        174 Ang**3
```

Author Response: A solvent mask calculation was done: the void was found but no electron density was found in it. it was then removed from the refinement.

Alert level C

```
PLAT042_ALERT_1_C Calc. and Reported MoietyFormula Strings  Differ     Please Check
              Calc: C81.96 H79.90 Nb8 O38, 0.8(C2 H3 N)
              Rep.: 2(C40.98 H39.95 Nb4 O19), 0.8(C2 H3 N)
PLAT077_ALERT_4_C Unitcell Contains Non-integer Number of Atoms ..     Please Check
PLAT157_ALERT_4_C Non-standard Monoclinic Beta Angle less 90 Deg        89.98 Degree
PLAT220_ALERT_2_C NonSolvent   Resd 1  C   Ueq(max)/Ueq(min) Range        3.1 Ratio
PLAT220_ALERT_2_C NonSolvent   Resd 2  C   Ueq(max)/Ueq(min) Range        4.2 Ratio
PLAT220_ALERT_2_C NonSolvent   Resd 2  O   Ueq(max)/Ueq(min) Range        3.2 Ratio
PLAT241_ALERT_2_C High   'MainMol' Ueq as Compared to Neighbors of        C69 Check
PLAT241_ALERT_2_C High   'MainMol' Ueq as Compared to Neighbors of        C96 Check
PLAT241_ALERT_2_C High   'MainMol' Ueq as Compared to Neighbors of        C35 Check
PLAT241_ALERT_2_C High   'MainMol' Ueq as Compared to Neighbors of       C107 Check
PLAT242_ALERT_2_C Low    'MainMol' Ueq as Compared to Neighbors of         O2 Check
PLAT242_ALERT_2_C Low    'MainMol' Ueq as Compared to Neighbors of        O17 Check
PLAT242_ALERT_2_C Low    'MainMol' Ueq as Compared to Neighbors of        O19 Check
PLAT242_ALERT_2_C Low    'MainMol' Ueq as Compared to Neighbors of        C64 Check
PLAT242_ALERT_2_C Low    'MainMol' Ueq as Compared to Neighbors of        O33 Check
PLAT242_ALERT_2_C Low    'MainMol' Ueq as Compared to Neighbors of        O35 Check
PLAT242_ALERT_2_C Low    'MainMol' Ueq as Compared to Neighbors of        C74 Check
PLAT260_ALERT_2_C Large Average Ueq of Residue Including       Nb5      0.101 Check
PLAT260_ALERT_2_C Large Average Ueq of Residue Including        N2      0.105 Check
PLAT342_ALERT_3_C Low Bond Precision on  C-C Bonds ...............    0.01566 Ang.
PLAT360_ALERT_2_C Short  C(sp3)-C(sp3) Bond  C13      - C96      .       1.34 Ang.
PLAT369_ALERT_2_C Long   C(sp2)-C(sp2) Bond  C74      - C107     .       1.55 Ang.
PLAT906_ALERT_3_C Large K Value in the Analysis of Variance ......      3.495 Check
PLAT911_ALERT_3_C Missing FCF Refl Between Thmin & STh/L=    0.600          5 Report
              -13 12  1,  -3  1  2,   4  2  2,   4  0  4,   6  0  4,
PLAT918_ALERT_3_C Reflection(s) with I(obs) much Smaller I(calc) .          8 Check
PLAT977_ALERT_2_C Check Negative Difference Density on H29       .      -0.41 eA-3
PLAT977_ALERT_2_C Check Negative Difference Density on H47C      .      -0.33 eA-3
```

Alert level G

```
ABSMU01_ALERT_1_G  Calculation of _exptl_absorpt_correction_mu
                not performed for this radiation type.
PLAT002_ALERT_2_G Number of Distance or Angle Restraints on AtSite         34 Note
PLAT003_ALERT_2_G Number of Uiso or Uij Restrained non-H Atoms ...         68 Report
PLAT083_ALERT_2_G SHELXL Second Parameter in WGHT  Unusually Large     158.13 Why ?
PLAT128_ALERT_4_G Alternate Setting for Input Space Group  I2/a          I2/c Note
PLAT171_ALERT_4_G The CIF-Embedded .res File Contains EADP Records          4 Report
PLAT176_ALERT_4_G The CIF-Embedded .res File Contains SADI Records         16 Report
PLAT178_ALERT_4_G The CIF-Embedded .res File Contains SIMU Records         14 Report
PLAT187_ALERT_4_G The CIF-Embedded .res File Contains RIGU Records         14 Report
PLAT300_ALERT_4_G Atom Site Occupancy of C45        Constrained at        0.5 Check
PLAT300_ALERT_4_G Atom Site Occupancy of C73        Constrained at        0.5 Check
PLAT300_ALERT_4_G Atom Site Occupancy of C89        Constrained at        0.5 Check
PLAT300_ALERT_4_G Atom Site Occupancy of C91        Constrained at        0.5 Check
PLAT300_ALERT_4_G Atom Site Occupancy of C101       Constrained at       0.65 Check
PLAT300_ALERT_4_G Atom Site Occupancy of C102       Constrained at       0.65 Check
PLAT300_ALERT_4_G Atom Site Occupancy of C103       Constrained at       0.65 Check
PLAT300_ALERT_4_G Atom Site Occupancy of C104       Constrained at       0.65 Check
PLAT300_ALERT_4_G Atom Site Occupancy of C105       Constrained at       0.65 Check
PLAT300_ALERT_4_G Atom Site Occupancy of C106       Constrained at       0.65 Check
PLAT300_ALERT_4_G Atom Site Occupancy of C3         Constrained at       0.33 Check
PLAT300_ALERT_4_G Atom Site Occupancy of C15        Constrained at       0.35 Check
PLAT300_ALERT_4_G Atom Site Occupancy of C23        Constrained at       0.33 Check
PLAT300_ALERT_4_G Atom Site Occupancy of C29        Constrained at       0.35 Check
PLAT300_ALERT_4_G Atom Site Occupancy of C31        Constrained at       0.35 Check
PLAT300_ALERT_4_G Atom Site Occupancy of C47        Constrained at       0.33 Check
PLAT300_ALERT_4_G Atom Site Occupancy of C57        Constrained at       0.35 Check
PLAT300_ALERT_4_G Atom Site Occupancy of C59        Constrained at       0.35 Check
PLAT300_ALERT_4_G Atom Site Occupancy of C61        Constrained at       0.35 Check
PLAT300_ALERT_4_G Atom Site Occupancy of C75        Constrained at       0.33 Check
PLAT300_ALERT_4_G Atom Site Occupancy of C93        Constrained at       0.33 Check
PLAT300_ALERT_4_G Atom Site Occupancy of C95        Constrained at       0.33 Check
PLAT300_ALERT_4_G Atom Site Occupancy of H45A       Constrained at        0.5 Check
PLAT300_ALERT_4_G Atom Site Occupancy of H45B       Constrained at        0.5 Check
PLAT300_ALERT_4_G Atom Site Occupancy of H73A       Constrained at        0.5 Check
PLAT300_ALERT_4_G Atom Site Occupancy of H73B       Constrained at        0.5 Check
PLAT300_ALERT_4_G Atom Site Occupancy of H89A       Constrained at        0.5 Check
PLAT300_ALERT_4_G Atom Site Occupancy of H89B       Constrained at        0.5 Check
PLAT300_ALERT_4_G Atom Site Occupancy of H89C       Constrained at        0.5 Check
PLAT300_ALERT_4_G Atom Site Occupancy of H91A       Constrained at        0.5 Check
PLAT300_ALERT_4_G Atom Site Occupancy of H91B       Constrained at        0.5 Check
PLAT300_ALERT_4_G Atom Site Occupancy of H91C       Constrained at        0.5 Check
PLAT300_ALERT_4_G Atom Site Occupancy of H102       Constrained at       0.65 Check
PLAT300_ALERT_4_G Atom Site Occupancy of H103       Constrained at       0.65 Check
PLAT300_ALERT_4_G Atom Site Occupancy of H104       Constrained at       0.65 Check
PLAT300_ALERT_4_G Atom Site Occupancy of H105       Constrained at       0.65 Check
PLAT300_ALERT_4_G Atom Site Occupancy of H106       Constrained at       0.65 Check
PLAT300_ALERT_4_G Atom Site Occupancy of H3A        Constrained at       0.33 Check
PLAT300_ALERT_4_G Atom Site Occupancy of H3B        Constrained at       0.33 Check
PLAT300_ALERT_4_G Atom Site Occupancy of H15        Constrained at       0.35 Check
PLAT300_ALERT_4_G Atom Site Occupancy of H23A       Constrained at       0.33 Check
PLAT300_ALERT_4_G Atom Site Occupancy of H23B       Constrained at       0.33 Check
PLAT300_ALERT_4_G Atom Site Occupancy of H23C       Constrained at       0.33 Check
PLAT300_ALERT_4_G Atom Site Occupancy of H29        Constrained at       0.35 Check
PLAT300_ALERT_4_G Atom Site Occupancy of H31        Constrained at       0.35 Check
PLAT300_ALERT_4_G Atom Site Occupancy of H47A       Constrained at       0.33 Check
```

```
PLAT300_ALERT_4_G Atom Site Occupancy of H47B       Constrained at       0.33 Check
PLAT300_ALERT_4_G Atom Site Occupancy of H47C       Constrained at       0.33 Check
PLAT300_ALERT_4_G Atom Site Occupancy of H59        Constrained at       0.35 Check
PLAT300_ALERT_4_G Atom Site Occupancy of H61        Constrained at       0.35 Check
PLAT300_ALERT_4_G Atom Site Occupancy of H75A       Constrained at       0.33 Check
PLAT300_ALERT_4_G Atom Site Occupancy of H75B       Constrained at       0.33 Check
PLAT300_ALERT_4_G Atom Site Occupancy of H93A       Constrained at       0.33 Check
PLAT300_ALERT_4_G Atom Site Occupancy of H93B       Constrained at       0.33 Check
PLAT300_ALERT_4_G Atom Site Occupancy of H95A       Constrained at       0.33 Check
PLAT300_ALERT_4_G Atom Site Occupancy of H95B       Constrained at       0.33 Check
PLAT300_ALERT_4_G Atom Site Occupancy of H95C       Constrained at       0.33 Check
PLAT300_ALERT_4_G Atom Site Occupancy of C37        Constrained at        0.6 Check
PLAT300_ALERT_4_G Atom Site Occupancy of C79        Constrained at        0.6 Check
PLAT300_ALERT_4_G Atom Site Occupancy of C90        Constrained at        0.6 Check
PLAT300_ALERT_4_G Atom Site Occupancy of C100       Constrained at        0.6 Check
PLAT300_ALERT_4_G Atom Site Occupancy of C5         Constrained at        0.4 Check
PLAT300_ALERT_4_G Atom Site Occupancy of C11        Constrained at       0.33 Check
PLAT300_ALERT_4_G Atom Site Occupancy of C39        Constrained at        0.4 Check
PLAT300_ALERT_4_G Atom Site Occupancy of C41        Constrained at        0.4 Check
PLAT300_ALERT_4_G Atom Site Occupancy of C49        Constrained at       0.33 Check
PLAT300_ALERT_4_G Atom Site Occupancy of C77        Constrained at       0.33 Check
PLAT300_ALERT_4_G Atom Site Occupancy of C81        Constrained at        0.4 Check
PLAT300_ALERT_4_G Atom Site Occupancy of C87        Constrained at       0.33 Check
PLAT300_ALERT_4_G Atom Site Occupancy of C97        Constrained at       0.33 Check
PLAT300_ALERT_4_G Atom Site Occupancy of C99        Constrained at       0.33 Check
PLAT300_ALERT_4_G Atom Site Occupancy of H10A       Constrained at        0.6 Check
PLAT300_ALERT_4_G Atom Site Occupancy of H10B       Constrained at        0.6 Check
PLAT300_ALERT_4_G Atom Site Occupancy of H10C       Constrained at        0.6 Check
PLAT300_ALERT_4_G Atom Site Occupancy of H37A       Constrained at        0.6 Check
PLAT300_ALERT_4_G Atom Site Occupancy of H37B       Constrained at        0.6 Check
PLAT300_ALERT_4_G Atom Site Occupancy of H79A       Constrained at        0.6 Check
PLAT300_ALERT_4_G Atom Site Occupancy of H79B       Constrained at        0.6 Check
PLAT300_ALERT_4_G Atom Site Occupancy of H79C       Constrained at        0.6 Check
PLAT300_ALERT_4_G Atom Site Occupancy of H90A       Constrained at        0.6 Check
PLAT300_ALERT_4_G Atom Site Occupancy of H90B       Constrained at        0.6 Check
PLAT300_ALERT_4_G Atom Site Occupancy of H5A        Constrained at        0.4 Check
PLAT300_ALERT_4_G Atom Site Occupancy of H5B        Constrained at        0.4 Check
PLAT300_ALERT_4_G Atom Site Occupancy of H5C        Constrained at        0.4 Check
PLAT300_ALERT_4_G Atom Site Occupancy of H11A       Constrained at       0.33 Check
PLAT300_ALERT_4_G Atom Site Occupancy of H11B       Constrained at       0.33 Check
PLAT300_ALERT_4_G Atom Site Occupancy of H39A       Constrained at        0.4 Check
PLAT300_ALERT_4_G Atom Site Occupancy of H39B       Constrained at        0.4 Check
PLAT300_ALERT_4_G Atom Site Occupancy of H41A       Constrained at        0.4 Check
PLAT300_ALERT_4_G Atom Site Occupancy of H41B       Constrained at        0.4 Check
PLAT300_ALERT_4_G Atom Site Occupancy of H41C       Constrained at        0.4 Check
PLAT300_ALERT_4_G Atom Site Occupancy of H49A       Constrained at       0.33 Check
PLAT300_ALERT_4_G Atom Site Occupancy of H49B       Constrained at       0.33 Check
PLAT300_ALERT_4_G Atom Site Occupancy of H49C       Constrained at       0.33 Check
PLAT300_ALERT_4_G Atom Site Occupancy of H77A       Constrained at       0.33 Check
PLAT300_ALERT_4_G Atom Site Occupancy of H77B       Constrained at       0.33 Check
PLAT300_ALERT_4_G Atom Site Occupancy of H81A       Constrained at        0.4 Check
PLAT300_ALERT_4_G Atom Site Occupancy of H81B       Constrained at        0.4 Check
PLAT300_ALERT_4_G Atom Site Occupancy of H87A       Constrained at       0.33 Check
PLAT300_ALERT_4_G Atom Site Occupancy of H87B       Constrained at       0.33 Check
PLAT300_ALERT_4_G Atom Site Occupancy of H87C       Constrained at       0.33 Check
PLAT300_ALERT_4_G Atom Site Occupancy of H97A       Constrained at       0.33 Check
PLAT300_ALERT_4_G Atom Site Occupancy of H97B       Constrained at       0.33 Check
```

```
PLAT300_ALERT_4_G Atom Site Occupancy of H97C       Constrained at       0.33 Check
PLAT300_ALERT_4_G Atom Site Occupancy of H99A       Constrained at       0.33 Check
PLAT300_ALERT_4_G Atom Site Occupancy of H99B       Constrained at       0.33 Check
PLAT300_ALERT_4_G Atom Site Occupancy of N1         Constrained at        0.4 Check
PLAT300_ALERT_4_G Atom Site Occupancy of C83        Constrained at        0.4 Check
PLAT300_ALERT_4_G Atom Site Occupancy of C85        Constrained at        0.4 Check
PLAT300_ALERT_4_G Atom Site Occupancy of H83A       Constrained at        0.4 Check
PLAT300_ALERT_4_G Atom Site Occupancy of H83B       Constrained at        0.4 Check
PLAT300_ALERT_4_G Atom Site Occupancy of H83C       Constrained at        0.4 Check
PLAT300_ALERT_4_G Atom Site Occupancy of N2         Constrained at        0.4 Check
PLAT300_ALERT_4_G Atom Site Occupancy of C21        Constrained at        0.4 Check
PLAT300_ALERT_4_G Atom Site Occupancy of C43        Constrained at        0.4 Check
PLAT300_ALERT_4_G Atom Site Occupancy of H21A       Constrained at        0.4 Check
PLAT300_ALERT_4_G Atom Site Occupancy of H21B       Constrained at        0.4 Check
PLAT300_ALERT_4_G Atom Site Occupancy of H21C       Constrained at        0.4 Check
PLAT301_ALERT_3_G Main Residue  Disorder .............(Resd  1  )         16% Note
PLAT301_ALERT_3_G Main Residue  Disorder .............(Resd  2  )          9% Note
PLAT302_ALERT_4_G Anion/Solvent/Minor-Residue Disorder (Resd  3  )       100% Note
PLAT302_ALERT_4_G Anion/Solvent/Minor-Residue Disorder (Resd  4  )       100% Note
PLAT304_ALERT_4_G Non-Integer Number of Atoms in ..... (Resd  1  )     207.86 Check
PLAT304_ALERT_4_G Non-Integer Number of Atoms in ..... (Resd  2  )     207.86 Check
PLAT304_ALERT_4_G Non-Integer Number of Atoms in ..... (Resd  3  )       2.40 Check
PLAT304_ALERT_4_G Non-Integer Number of Atoms in ..... (Resd  4  )       2.40 Check
PLAT411_ALERT_2_G Short Inter H...H Contact  H102     ..H110      .       1.78 Ang.
                                               1-x,1-y,1-z  =      5_666 Check
PLAT411_ALERT_2_G Short Inter H...H Contact  H52      ..H73B      .       2.14 Ang.
                                              -1/2+x,1-y,z  =      6_565 Check
PLAT411_ALERT_2_G Short Inter H...H Contact  H108     ..H15       .       1.30 Ang.
                                             x,1/2-y,-1/2+z  =      8_554 Check
PLAT412_ALERT_2_G Short Intra XH3 .. XHn     H112     ..H49B      .       2.13 Ang.
                                                     x,y,z  =      1_555 Check
PLAT413_ALERT_2_G Short Inter XH3 .. XHn     H72      ..H97B      .       2.04 Ang.
                                               1-x,1-y,1-z  =      5_666 Check
PLAT413_ALERT_2_G Short Inter XH3 .. XHn     H96B     ..H95B      .       1.96 Ang.
                                         3/2-x,3/2-y,3/2-z  =      7_666 Check
PLAT413_ALERT_2_G Short Inter XH3 .. XHn     H25      ..H87C      .       1.68 Ang.
                                               1-x,2-y,1-z  =      5_676 Check
PLAT432_ALERT_2_G Short Inter X...Y Contact  C102     ..C110      .       2.88 Ang.
                                               1-x,1-y,1-z  =      5_666 Check
PLAT432_ALERT_2_G Short Inter X...Y Contact  C103     ..C110      .       3.08 Ang.
                                               1-x,1-y,1-z  =      5_666 Check
PLAT432_ALERT_2_G Short Inter X...Y Contact  C108     ..C15       .       2.75 Ang.
                                             x,1/2-y,-1/2+z  =      8_554 Check
PLAT432_ALERT_2_G Short Inter X...Y Contact  C109     ..C15       .       3.12 Ang.
                                             x,1/2-y,-1/2+z  =      8_554 Check
PLAT432_ALERT_2_G Short Inter X...Y Contact  N1       ..C97       .       2.57 Ang.
                                                     x,y,z  =      1_555 Check
PLAT432_ALERT_2_G Short Inter X...Y Contact  N2       ..C47       .       2.63 Ang.
                                               3/2-x,y,1-z  =      2_656 Check
PLAT432_ALERT_2_G Short Inter X...Y Contact  C21      ..C47       .       3.11 Ang.
                                               3/2-x,y,1-z  =      2_656 Check
PLAT432_ALERT_2_G Short Inter X...Y Contact  C43      ..C47       .       2.55 Ang.
                                               3/2-x,y,1-z  =      2_656 Check
PLAT432_ALERT_2_G Short Inter X...Y Contact  C83      ..C97       .       2.96 Ang.
                                                     x,y,z  =      1_555 Check
PLAT432_ALERT_2_G Short Inter X...Y Contact  C85      ..C97       .       2.46 Ang.
                                                     x,y,z  =      1_555 Check
```

```
PLAT722_ALERT_1_G Angle   Calc      109.00, Rep      107.90 Dev...       1.10 Degree
              C79     -C37     -H37A       1_555  1_555  1_555     #  389 Check
PLAT794_ALERT_5_G Tentative Bond Valency for Nb2       (V)       .       5.01 Info
PLAT860_ALERT_3_G Number of Least-Squares Restraints ............        762 Note
PLAT910_ALERT_3_G Missing # of FCF Reflection(s) Below Theta(Min).         3 Note
                2  0  0,   0  0  2,   2  0  2,
PLAT912_ALERT_4_G Missing # of FCF Reflections Above STh/L=  0.600        227 Note
PLAT933_ALERT_2_G Number of HKL-OMIT Records in Embedded .res File         6 Note
              -13 12  1,  -3  1  2,   2  0  2,   4  0  4,   4  2  2,   6  0  4,
PLAT978_ALERT_2_G Number C-C Bonds with Positive Residual Density.         0 Info
```

```
   0 ALERT level A = Most likely a serious problem - resolve or explain
   2 ALERT level B = A potentially serious problem, consider carefully
  27 ALERT level C = Check. Ensure it is not caused by an omission or oversight
 159 ALERT level G = General information/check it is not something unexpected

   3 ALERT type 1 CIF construction/syntax error, inconsistent or missing data
  44 ALERT type 2 Indicator that the structure model may be wrong or deficient
   8 ALERT type 3 Indicator that the structure quality may be low
 132 ALERT type 4 Improvement, methodology, query or suggestion
   1 ALERT type 5 Informative message, check
```

It is advisable to attempt to resolve as many as possible of the alerts in all categories. Often the minor alerts point to easily fixed oversights, errors and omissions in your CIF or refinement strategy, so attention to these fine details can be worthwhile. In order to resolve some of the more serious problems it may be necessary to carry out additional measurements or structure refinements. However, the purpose of your study may justify the reported deviations and the more serious of these should normally be commented upon in the discussion or experimental section of a paper or in the "special_details" fields of the CIF. checkCIF was carefully designed to identify outliers and unusual parameters, but every test has its limitations and alerts that are not important in a particular case may appear. Conversely, the absence of alerts does not guarantee there are no aspects of the results needing attention. It is up to the individual to critically assess their own results and, if necessary, seek expert advice.

Publication of your CIF in IUCr journals

A basic structural check has been run on your CIF. These basic checks will be run on all CIFs submitted for publication in IUCr journals (*Acta Crystallographica*, *Journal of Applied Crystallography*, *Journal of Synchrotron Radiation*); however, if you intend to submit to *Acta Crystallographica Section C* or *E* or *IUCrData*, you should make sure that full publication checks are run on the final version of your CIF prior to submission.

Publication of your CIF in other journals

Please refer to the *Notes for Authors* of the relevant journal for any special instructions relating to CIF submission.

PLATON version of 29/11/2023; check.def file version of 14/09/2023

Datablock mjp104-3_150k - ellipsoid plot

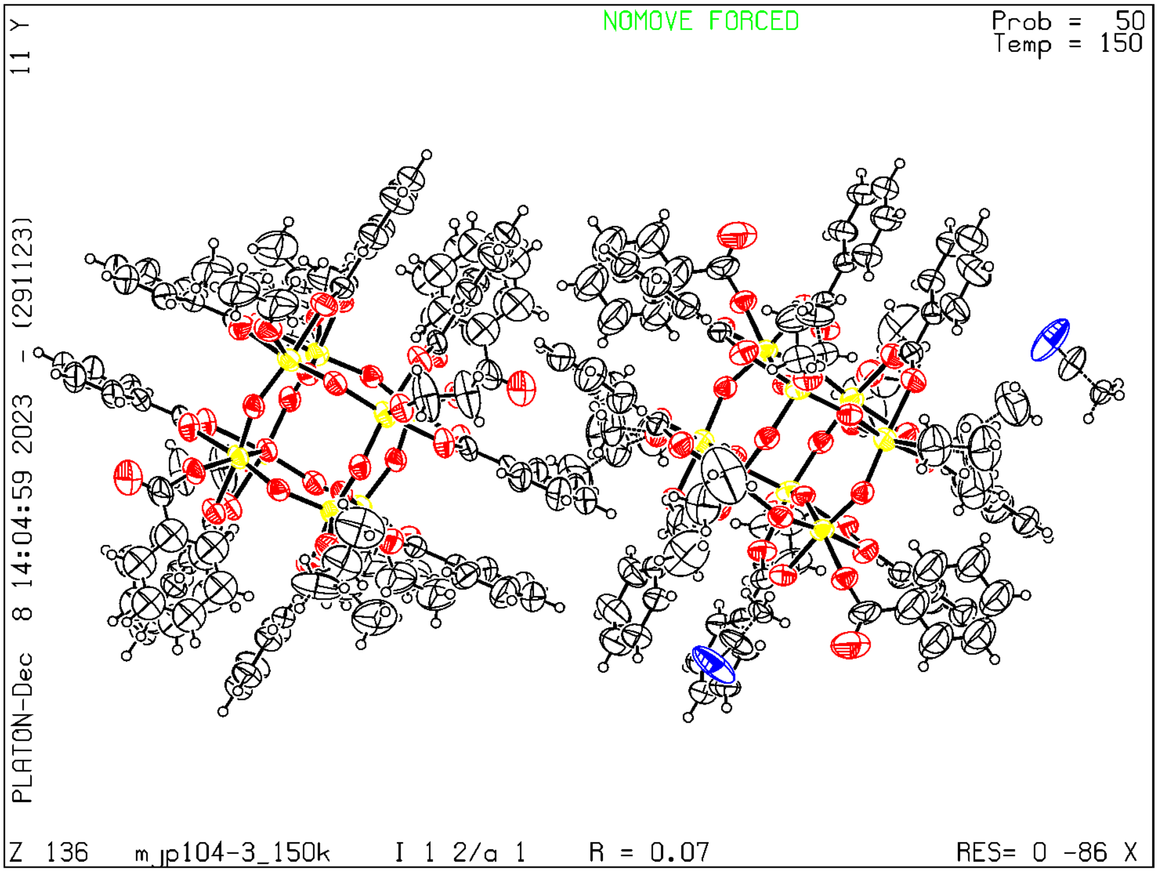

Supplement: Supplementary file 2 — Supporting File 2: anie71298–sup–0002–Data.zip. [file ANIE-65-e25769-s002.zip › CCDC_2495249/MJP104-3_150K_cifreport.pdf]
