# Supplementary material for: Mechanism‐Guided Precision Hydrolysis of Early Transition Metals to Access (Mixed‐Metal) Oxo Clusters
Source: Angew Chem Int Ed Engl. 2026 Feb 24;65(15):e25769. doi: 10.1002/anie.202525769 (PMC13053926; doi:10.1002/anie.202525769)
Supplement: Supplementary file 2 — Supporting File 2: anie71298–sup–0002–Data.zip. [file ANIE-65-e25769-s002.zip › CCDC_2495249/MJP104-3_150K_tables.html]

MJP104-3\_150K


# MJP104-3\_150K

b"\n \n \n "

Table 1 Crystal data and structure refinement for MJP104-3\_150K.

| Identification code | MJP104-3\_150K |
| Empirical formula | C83.56H82.3N0.8Nb8O38 |
| Formula weight | 2449.00 |
| Temperature/K | 150 |
| Crystal system | monoclinic |
| Space group | I2/a |
| a/Å | 42.5266(5) |
| b/Å | 10.7503(2) |
| c/Å | 42.4305(6) |
| α/° | 90 |
| β/° | 89.9780(10) |
| γ/° | 90 |
| Volume/Å3 | 19398.1(5) |
| Z | 8 |
| ρcalcg/cm3 | 1.677 |
| μ/mm‑1 | 5.494 |
| F(000) | 9770.0 |
| Crystal size/mm3 | 0.22 × 0.207 × 0.18 |
| Radiation | GaKα (λ = 1.34143) |
| 2Θ range for data collection/° | 5.12 to 111.526 |
| Index ranges | -51 ≤ h ≤ 51, -11 ≤ k ≤ 13, -52 ≤ l ≤ 39 |
| Reflections collected | 127791 |
| Independent reflections | 18778 [Rint = 0.0637, Rsigma = 0.0284] |
| Data/restraints/parameters | 18778/762/1241 |
| Goodness-of-fit on F2 | 1.061 |
| Final R indexes [I>=2σ (I)] | R1 = 0.0684, wR2 = 0.1864 |
| Final R indexes [all data] | R1 = 0.0807, wR2 = 0.1991 |
| Largest diff. peak/hole / e Å-3 | 0.89/-1.14 |

Table 2 Fractional Atomic Coordinates (×104) and Equivalent Isotropic Displacement Parameters (Å2×103) for MJP104-3\_150K. Ueq is defined as 1/3 of the trace of the orthogonalised UIJ tensor.

| Atom | *x* | *y* | *z* | U(eq) |
| --- | --- | --- | --- | --- |
| Nb1 | 6860.4(2) | 2098.5(6) | 7160.7(2) | 54.41(16) |
| Nb7 | 5338.9(2) | 5402.3(6) | 4360.8(2) | 54.63(16) |
| Nb4 | 7614.5(2) | 981.0(7) | 6876.2(2) | 61.63(17) |
| Nb3 | 7953.2(2) | 4010.4(6) | 7052.7(2) | 60.79(17) |
| Nb2 | 7191.3(2) | 5147.1(6) | 7332.1(2) | 59.63(17) |
| Nb6 | 5623.4(2) | 6518.8(7) | 5114.7(2) | 61.72(17) |
| Nb8 | 4552.6(2) | 6510.9(7) | 4546.8(2) | 61.05(17) |
| Nb5 | 4832.0(2) | 7646.4(6) | 5308.8(2) | 60.25(17) |
| O1 | 7124.5(10) | 3371(4) | 7301.5(11) | 52.8(10) |
| O4 | 5198.9(10) | 4135(4) | 4625.3(10) | 52.8(10) |
| O6 | 7609.1(11) | 5037(5) | 7172.5(11) | 59.9(12) |
| O8 | 7719.5(10) | 2500(5) | 7083.9(11) | 56.3(11) |
| O10 | 5268.4(11) | 7536(5) | 5201.8(11) | 59.1(12) |
| O12 | 6867.0(10) | 1157(5) | 7538.4(11) | 57.6(11) |
| O14 | 4582.3(10) | 4998(5) | 4780.6(11) | 56.6(11) |
| O16 | 4958.4(11) | 6352(5) | 4369.0(11) | 57.5(11) |
| O18 | 7171.8(11) | 1151(5) | 6947.9(11) | 59.2(12) |
| O20 | 5552.3(11) | 6347(5) | 4673.9(12) | 59.6(12) |
| O22 | 4673.2(11) | 7544(5) | 4890.8(11) | 60.4(12) |
| O24 | 7301.8(11) | 5037(5) | 7767.7(12) | 61.0(12) |
| O26 | 4256.2(12) | 5761(5) | 5700.8(12) | 65.8(13) |
| O28 | 5515.3(12) | 6305(5) | 3990.8(11) | 66.4(13) |
| O30 | 6489.8(11) | 1201(5) | 6984.8(13) | 66.9(13) |
| O32 | 8311.0(12) | 2834(6) | 6859.0(13) | 70.1(14) |
| O34 | 6490.1(11) | 3263(5) | 7353.9(13) | 64.5(13) |
| O36 | 6799.0(12) | 3257(5) | 6757.6(13) | 67.3(13) |
| O38 | 6700.2(11) | 5143(5) | 7446.6(14) | 69.8(14) |
| O19 | 7150.6(12) | 6916(5) | 7310.8(14) | 72.0(14) |
| O5 | 8106.2(12) | 970(6) | 6758.1(14) | 73.2(15) |
| O21 | 4854.2(13) | 5762(5) | 6009.5(11) | 66.0(13) |
| O11 | 5741.2(13) | 6522(6) | 5606.6(13) | 73.2(15) |
| O23 | 6030.3(12) | 5346(6) | 5071.5(13) | 70.6(14) |
| O3 | 4361.2(13) | 7633(5) | 5491.7(13) | 69.1(14) |
| O25 | 4811.3(13) | 9410(5) | 5350.2(13) | 72.8(14) |
| O13 | 4947.6(14) | 7648(5) | 5801.1(12) | 72.8(14) |
| O27 | 5922.6(12) | 3481(6) | 5280.2(13) | 70.7(14) |
| O7 | 7008.9(12) | 5134(5) | 6859.3(13) | 69.2(14) |
| O29 | 7572.5(13) | 2154(6) | 6468.5(12) | 70.8(14) |
| O15 | 7777.5(13) | 4024(6) | 6577.1(13) | 72.9(14) |
| O31 | 5639.4(13) | 4668(6) | 5811.3(12) | 72.2(15) |
| O2 | 8220.9(13) | 5339(6) | 6903.7(15) | 81.6(16) |
| O33 | 5931.7(14) | 7840(6) | 5091.7(13) | 80.4(16) |
| O17 | 7592.4(13) | -344(6) | 6568.8(14) | 79.9(16) |
| O35 | 4405.2(15) | 7841(7) | 4280.7(14) | 84.9(17) |
| C1 | 6471.0(15) | 4385(7) | 7436.2(17) | 58.0(17) |
| C4 | 4183.2(18) | 6875(8) | 5639.1(17) | 62.9(18) |
| C6 | 3871.7(17) | 7312(8) | 5750.5(16) | 61.4(18) |
| C8 | 6156.0(17) | 4860(8) | 7526.0(18) | 65(2) |
| C10 | 6747.2(15) | 4813(8) | 6374.5(18) | 60.1(17) |
| C12 | 6862.2(16) | 4375(8) | 6684.7(18) | 61.7(18) |
| C14 | 7632.1(17) | 3261(9) | 6401.6(17) | 65.1(19) |
| C16 | 4938.6(16) | 6884(7) | 6030.3(16) | 57.1(16) |
| C18 | 6098.4(17) | 4247(9) | 5131.8(18) | 65.1(19) |
| C20 | 8334.8(17) | 1726(9) | 6775.7(18) | 67(2) |
| C22 | 6408.3(18) | 3757(10) | 5019.3(19) | 75(2) |
| C24 | 5027.7(17) | 7365(9) | 6344.2(18) | 66(2) |
| C26 | 3681.1(19) | 6526(9) | 5920(2) | 76(2) |
| C28 | 5724.1(18) | 5765(9) | 5834.5(17) | 65.5(19) |
| C30 | 7517.9(18) | 3750(11) | 6092.1(18) | 77(2) |
| C32 | 6580.2(19) | 4038(9) | 6180(2) | 74(2) |
| C34 | 8657.3(18) | 1272(11) | 6680.0(18) | 78(2) |
| C36 | 5817.7(18) | 6239(11) | 6152.0(19) | 78(2) |
| C38 | 5906.4(18) | 4089(10) | 7565(2) | 79(2) |
| C40 | 3772(2) | 8513(9) | 5697(2) | 82(2) |
| C42 | 5074(2) | 8606(11) | 6389(2) | 87(3) |
| C44 | 6111(2) | 6124(10) | 7574(2) | 84(3) |
| C46 | 6801(2) | 6015(10) | 6279(2) | 84(3) |
| C48 | 3486(2) | 8907(11) | 5806(3) | 93(3) |
| C50 | 5063(2) | 6583(11) | 6595.1(19) | 83(3) |
| C52 | 3293(2) | 8108(12) | 5963(2) | 88(3) |
| C54 | 3391(2) | 6931(11) | 6026(2) | 87(3) |
| O9 | 6093(2) | 1436(13) | 6675(2) | 153(4) |
| C56 | 5566(2) | 5752(13) | 7684(2) | 99(3) |
| C58 | 5612(2) | 4500(11) | 7642(2) | 91(3) |
| C60 | 6693(2) | 6413(11) | 5990(2) | 93(3) |
| C62 | 6536(2) | 5612(11) | 5792(2) | 85(3) |
| O37 | 5825(2) | 6076(14) | 3587(2) | 170(5) |
| C64 | 6194(2) | 1275(12) | 6922(3) | 97(3) |
| C66 | 5181(2) | 8266(13) | 6935(2) | 97(3) |
| C68 | 5143(2) | 7011(12) | 6891(2) | 94(3) |
| C70 | 8900(2) | 2089(13) | 6656(2) | 100(3) |
| C72 | 6474(2) | 4433(11) | 5892(2) | 89(3) |
| C74 | 5579(2) | 6225(12) | 3691(2) | 101(4) |
| C76 | 7296(3) | 4591(19) | 5523(3) | 135(5) |
| C78 | 5843(2) | 5392(13) | 6402(2) | 100(3) |
| C80 | 8704(3) | 26(13) | 6627(3) | 106(3) |
| C82 | 6605(2) | 4513(14) | 4847(2) | 106(3) |
| C84 | 5143(3) | 9092(12) | 6689(3) | 101(3) |
| C86 | 5813(2) | 6605(12) | 7650(3) | 105(4) |
| C88 | 9243(3) | 429(17) | 6517(3) | 124(4) |
| C90 | 4814(5) | 10508(14) | 5529(4) | 82(5) |
| C92 | 7344(2) | 2983(15) | 5896(2) | 107(3) |
| C94 | 6497(2) | 2552(11) | 5086(3) | 99(3) |
| C96 | 7005(3) | 8005(11) | 7262(4) | 144(6) |
| C98 | 7580(3) | 4934(12) | 6005(2) | 101(3) |
| C100 | 4616(8) | 10580(40) | 5769(6) | 121(12) |
| C25 | 5872(3) | 7481(13) | 6204(3) | 109(3) |
| C51 | 5932(3) | 5850(17) | 6697(2) | 120(4) |
| C13 | 6766(3) | 8146(12) | 7059(4) | 138(6) |
| C53 | 6978(3) | 2901(19) | 4799(3) | 137(4) |
| C27 | 6893(3) | 4069(19) | 4740(3) | 132(4) |
| C55 | 5984(3) | 7131(18) | 6744(3) | 126(4) |
| C7 | 9193(2) | 1696(17) | 6566(3) | 124(4) |
| C57 | 5994(7) | 740(30) | 7212(6) | 120 |
| C29 | 5668(7) | 770(30) | 7195(5) | 120 |
| C59 | 5489(5) | 460(30) | 7458(6) | 120 |
| C15 | 5637(6) | 110(20) | 7736(4) | 120 |
| C61 | 5963(6) | 70(30) | 7752(5) | 120 |
| C31 | 6142(5) | 390(30) | 7490(7) | 120 |
| C69 | 9005(3) | -398(17) | 6547(3) | 136(4) |
| C35 | 5948(3) | 7902(17) | 6506(3) | 133(4) |
| C71 | 6786(3) | 2129(14) | 4970(4) | 126(4) |
| C9 | 7469(4) | 5349(15) | 5713(3) | 129(4) |
| C73 | 7713(7) | -580(30) | 6260(5) | 142(5) |
| C37 | 6241(4) | 8170(20) | 5204(5) | 117(6) |
| C75 | 8496(8) | 5640(30) | 6718(10) | 105(9) |
| C19 | 7238(3) | 3435(19) | 5610(3) | 130(4) |
| C77 | 4437(11) | 9200(20) | 4298(11) | 144(9) |
| C39 | 6054(10) | 9010(30) | 5208(10) | 139(9) |
| C79 | 6411(8) | 8950(40) | 5027(7) | 229(16) |
| C5 | 5828(11) | 9870(40) | 5269(16) | 240(20) |
| C81 | 4655(7) | 10510(20) | 5426(6) | 84(7) |
| C41 | 4498(9) | 10730(40) | 5687(8) | 90(11) |
| C83 | 3230(5) | 9678(17) | 3709(4) | 58(4) |
| C21 | 6212(4) | 7192(19) | 4273(5) | 62(4) |
| C85 | 3249(6) | 9808(18) | 4027(5) | 81(6) |
| N1 | 3252(9) | 9870(30) | 4277(5) | 153(12) |
| C43 | 6520(5) | 7305(19) | 4238(7) | 85(7) |
| N2 | 6771(5) | 7370(30) | 4235(10) | 167(13) |
| C87 | 4182(12) | 9510(40) | 4022(12) | 147(14) |
| C11 | 4125(9) | 8470(50) | 4165(11) | 129(10) |
| C89 | 7555(7) | -1820(30) | 6176(6) | 142(5) |
| C45 | 7716(7) | -1520(20) | 6467(7) | 142(5) |
| C91 | 7576(7) | -470(30) | 5951(6) | 142(5) |
| C23 | 8578(10) | 6930(30) | 6781(12) | 149(14) |
| C93 | 8371(9) | 5780(40) | 6615(8) | 112(10) |
| C47 | 8388(13) | 6210(50) | 6288(9) | 174(17) |
| C95 | 8048(12) | 7350(40) | 6881(15) | 180(20) |
| C3 | 8297(11) | 6540(30) | 6766(12) | 134(9) |
| C97 | 3748(9) | 8690(60) | 4102(15) | 200(20) |
| C49 | 4427(12) | 9230(50) | 3854(12) | 168(17) |
| C99 | 4203(9) | 8170(40) | 4018(8) | 112(9) |
| C101 | 5976(4) | 1003(16) | 7198(3) | 121(3) |
| C102 | 5662(4) | 1325(13) | 7159(3) | 121(3) |
| C103 | 5455(3) | 1238(13) | 7412(3) | 121(3) |
| C104 | 5563(3) | 830(13) | 7703(3) | 121(3) |
| C105 | 5877(3) | 508(14) | 7742(3) | 121(3) |
| C106 | 6084(3) | 594(17) | 7489(4) | 121(3) |
| C107 | 5296(2) | 6611(12) | 3483(2) | 158(3) |
| C108 | 5330(2) | 6384(11) | 3162(2) | 158(3) |
| C109 | 5074(3) | 6526(11) | 2961.7(15) | 158(3) |
| C110 | 4785(2) | 6895(11) | 3082(2) | 158(3) |
| C112 | 4751(2) | 7122(11) | 3402(2) | 158(3) |
| C111 | 5007(3) | 6980(11) | 3603.2(15) | 158(3) |

Table 3 Anisotropic Displacement Parameters (Å2×103) for MJP104-3\_150K. The Anisotropic displacement factor exponent takes the form: -2π2[h2a\*2U11+2hka\*b\*U12+…].

| Atom | U11 | U22 | U33 | U23 | U13 | U12 |
| --- | --- | --- | --- | --- | --- | --- |
| Nb1 | 40.8(3) | 61.2(3) | 61.3(3) | -9.4(3) | -8.2(2) | -1.8(2) |
| Nb7 | 52.7(3) | 61.2(3) | 49.9(3) | 1.6(2) | 7.1(2) | -9.6(3) |
| Nb4 | 46.0(3) | 72.4(4) | 66.5(4) | -8.1(3) | 1.1(3) | -1.0(3) |
| Nb3 | 51.8(3) | 72.1(4) | 58.5(3) | -5.3(3) | -0.8(3) | -3.6(3) |
| Nb2 | 45.8(3) | 61.1(4) | 72.1(4) | -8.8(3) | -12.8(3) | -1.7(2) |
| Nb6 | 58.6(4) | 71.7(4) | 54.9(3) | 0.8(3) | -0.9(3) | -8.4(3) |
| Nb8 | 50.0(3) | 72.2(4) | 61.0(3) | 3.5(3) | 0.2(3) | -5.4(3) |
| Nb5 | 64.6(4) | 61.1(4) | 55.0(3) | 1.6(3) | 12.3(3) | -9.6(3) |
| O1 | 41(2) | 57(3) | 60(3) | -6(2) | -7.0(19) | 2.4(19) |
| O4 | 50(2) | 63(3) | 46(2) | -2(2) | 8.1(18) | -9(2) |
| O6 | 54(3) | 65(3) | 61(3) | -5(2) | -11(2) | -6(2) |
| O8 | 43(2) | 68(3) | 58(3) | -5(2) | -2.7(19) | -3(2) |
| O10 | 63(3) | 63(3) | 52(2) | -2(2) | 4(2) | -15(2) |
| O12 | 42(2) | 72(3) | 60(3) | -9(2) | -1.3(19) | -10(2) |
| O14 | 46(2) | 72(3) | 52(2) | 8(2) | -0.8(19) | -8(2) |
| O16 | 57(3) | 64(3) | 51(2) | 8(2) | 1(2) | -8(2) |
| O18 | 49(2) | 72(3) | 57(3) | -12(2) | -10(2) | -3(2) |
| O20 | 49(3) | 66(3) | 64(3) | 5(2) | 9(2) | -15(2) |
| O22 | 58(3) | 61(3) | 61(3) | 3(2) | 11(2) | -5(2) |
| O24 | 48(3) | 64(3) | 71(3) | -10(2) | -3(2) | 1(2) |
| O26 | 59(3) | 73(3) | 66(3) | 3(3) | 17(2) | -6(2) |
| O28 | 70(3) | 75(3) | 54(3) | 5(2) | 10(2) | -16(3) |
| O30 | 43(2) | 84(4) | 74(3) | -18(3) | -15(2) | -9(2) |
| O32 | 49(3) | 88(4) | 74(3) | -12(3) | 8(2) | -8(3) |
| O34 | 43(2) | 69(3) | 81(3) | -13(3) | -8(2) | 3(2) |
| O36 | 60(3) | 72(3) | 70(3) | -5(3) | -20(2) | -3(3) |
| O38 | 44(3) | 70(3) | 96(4) | -16(3) | -10(2) | 6(2) |
| O19 | 60(3) | 71(3) | 85(4) | -8(3) | -16(3) | 6(2) |
| O5 | 51(3) | 87(4) | 81(4) | -19(3) | 7(2) | 2(3) |
| O21 | 77(3) | 71(3) | 51(3) | 3(2) | 3(2) | -13(3) |
| O11 | 68(3) | 89(4) | 63(3) | 0(3) | -5(2) | -19(3) |
| O23 | 50(3) | 87(4) | 75(3) | 11(3) | 0(2) | -12(3) |
| O3 | 69(3) | 69(3) | 70(3) | 6(3) | 22(3) | -3(3) |
| O25 | 76(3) | 72(3) | 71(3) | -8(3) | 15(3) | -10(3) |
| O13 | 89(4) | 71(3) | 59(3) | -2(3) | 11(3) | -16(3) |
| O27 | 52(3) | 85(4) | 75(3) | 6(3) | 3(2) | -5(3) |
| O7 | 60(3) | 69(3) | 78(3) | -2(3) | -24(3) | -4(2) |
| O29 | 68(3) | 87(4) | 57(3) | -12(3) | 0(2) | -17(3) |
| O15 | 68(3) | 87(4) | 63(3) | -1(3) | -2(3) | -7(3) |
| O31 | 61(3) | 95(4) | 60(3) | 10(3) | -12(2) | -16(3) |
| O2 | 62(3) | 96(4) | 87(4) | 4(3) | 6(3) | -19(3) |
| O33 | 72(3) | 100(4) | 69(3) | 4(3) | -3(3) | -31(3) |
| O17 | 66(3) | 97(4) | 77(3) | -30(3) | 4(3) | -1(3) |
| O35 | 78(4) | 102(5) | 75(4) | 25(3) | -6(3) | 6(3) |
| C1 | 41(3) | 70(5) | 63(4) | -9(3) | -12(3) | 7(3) |
| C4 | 62(4) | 74(5) | 53(4) | -8(4) | 12(3) | -7(4) |
| C6 | 53(4) | 81(5) | 50(4) | -13(3) | 7(3) | -5(4) |
| C8 | 46(4) | 92(6) | 58(4) | -10(4) | -12(3) | 11(4) |
| C10 | 39(3) | 77(5) | 64(4) | -2(4) | -5(3) | 12(3) |
| C12 | 41(3) | 79(5) | 65(4) | -3(4) | -14(3) | 7(3) |
| C14 | 49(4) | 93(6) | 53(4) | -8(4) | 1(3) | 0(4) |
| C16 | 49(4) | 73(5) | 49(3) | -3(3) | 8(3) | -6(3) |
| C18 | 47(4) | 91(6) | 57(4) | -4(4) | -1(3) | -8(4) |
| C20 | 46(4) | 97(6) | 59(4) | -10(4) | 3(3) | -2(4) |
| C22 | 48(4) | 117(6) | 59(4) | -22(4) | -2(3) | -7(4) |
| C24 | 50(4) | 91(6) | 57(4) | -13(4) | 11(3) | -8(4) |
| C26 | 62(5) | 89(6) | 78(5) | -13(5) | 20(4) | -2(4) |
| C28 | 56(4) | 92(6) | 49(4) | 3(4) | -4(3) | -9(4) |
| C30 | 53(4) | 125(6) | 54(4) | -5(4) | 4(3) | 23(4) |
| C32 | 65(5) | 85(6) | 72(5) | 0(4) | -17(4) | 4(4) |
| C34 | 51(4) | 132(6) | 53(4) | -4(4) | 4(3) | 13(4) |
| C36 | 46(4) | 128(6) | 62(4) | -12(4) | -2(3) | -10(4) |
| C38 | 49(4) | 102(7) | 86(6) | -20(5) | 3(4) | 4(4) |
| C40 | 73(5) | 86(6) | 85(6) | 3(5) | 20(4) | -1(5) |
| C42 | 86(6) | 107(8) | 69(5) | -26(5) | -2(4) | -7(5) |
| C44 | 63(5) | 90(6) | 97(6) | -3(5) | -1(4) | 22(5) |
| C46 | 74(6) | 103(7) | 76(5) | 2(5) | -19(4) | -8(5) |
| C48 | 82(6) | 101(7) | 97(7) | 8(6) | 23(5) | 13(5) |
| C50 | 81(6) | 112(7) | 57(4) | -9(5) | -4(4) | -26(5) |
| C52 | 61(5) | 128(9) | 76(6) | -9(6) | 10(4) | 8(5) |
| C54 | 72(5) | 108(8) | 81(6) | -10(5) | 28(5) | -4(5) |
| O9 | 105(6) | 245(13) | 110(7) | -12(7) | -27(5) | 16(7) |
| C56 | 55(5) | 148(10) | 95(7) | -17(7) | 1(5) | 28(6) |
| C58 | 55(5) | 115(8) | 103(7) | -27(6) | 2(4) | 6(5) |
| C60 | 96(7) | 95(7) | 89(6) | 17(6) | -26(5) | -16(6) |
| C62 | 67(5) | 119(8) | 70(5) | 13(5) | -9(4) | 15(5) |
| O37 | 118(7) | 282(15) | 111(7) | -18(8) | 39(6) | -21(8) |
| C64 | 61(5) | 134(9) | 96(7) | -32(7) | -21(5) | -11(5) |
| C66 | 86(6) | 144(10) | 62(5) | -27(6) | 0(4) | -16(7) |
| C68 | 91(7) | 122(9) | 69(5) | -8(6) | -8(5) | -25(6) |
| C70 | 51(4) | 165(8) | 85(6) | -6(6) | 7(4) | -4(5) |
| C72 | 81(6) | 110(8) | 77(6) | -7(6) | -25(5) | 14(6) |
| C74 | 93(7) | 140(10) | 69(5) | 17(6) | 28(5) | -42(7) |
| C76 | 101(8) | 223(13) | 80(7) | 14(7) | -9(6) | 57(9) |
| C78 | 80(6) | 165(9) | 55(4) | 6(5) | -8(4) | -13(6) |
| C80 | 75(5) | 141(7) | 100(7) | -16(7) | 10(5) | 34(5) |
| C82 | 57(5) | 185(10) | 75(5) | 15(6) | 10(4) | -10(5) |
| C84 | 105(8) | 112(8) | 85(7) | -36(6) | -6(6) | -17(6) |
| C86 | 75(6) | 114(9) | 126(9) | -9(7) | 4(6) | 32(6) |
| C88 | 62(5) | 227(12) | 85(6) | -12(8) | 6(5) | 32(7) |
| C90 | 102(13) | 58(7) | 87(11) | -5(6) | 14(9) | -11(8) |
| C92 | 65(5) | 190(10) | 68(5) | -5(6) | -8(4) | -10(6) |
| C94 | 62(5) | 110(6) | 126(8) | -38(6) | -8(5) | 2(5) |
| C96 | 125(9) | 75(6) | 231(14) | -15(8) | -97(10) | 15(6) |
| C98 | 121(8) | 120(6) | 62(5) | 2(5) | 6(5) | 42(6) |
| C100 | 170(30) | 89(15) | 106(15) | 4(12) | 48(16) | 28(18) |
| C25 | 107(8) | 135(7) | 86(6) | -28(6) | -17(6) | -26(7) |
| C51 | 87(7) | 215(11) | 59(5) | -1(6) | -7(5) | -11(8) |
| C13 | 116(9) | 92(8) | 205(14) | -10(9) | -85(9) | 15(7) |
| C53 | 76(7) | 222(13) | 114(9) | -50(9) | 8(6) | 10(7) |
| C27 | 64(6) | 241(13) | 92(7) | 5(9) | 15(5) | -1(7) |
| C55 | 77(6) | 224(12) | 77(6) | -32(7) | -9(5) | -18(8) |
| C7 | 54(5) | 219(11) | 97(7) | -16(9) | 9(5) | 0(6) |
| C69 | 81(6) | 188(11) | 138(10) | -17(9) | 23(7) | 48(6) |
| C35 | 133(10) | 178(11) | 89(6) | -44(6) | -18(7) | -19(9) |
| C71 | 68(6) | 145(9) | 164(11) | -63(8) | -2(6) | 10(6) |
| C9 | 155(11) | 155(10) | 76(6) | 16(6) | 0(6) | 63(8) |
| C73 | 169(11) | 139(9) | 120(7) | -62(7) | 28(8) | 3(9) |
| C37 | 85(10) | 131(14) | 134(14) | 14(12) | -29(10) | -46(10) |
| C75 | 96(16) | 74(13) | 143(19) | 1(15) | 47(14) | -24(13) |
| C19 | 79(7) | 237(13) | 75(6) | -5(8) | -15(5) | 5(9) |
| C77 | 160(20) | 112(9) | 160(20) | 41(16) | -25(17) | 0(14) |
| C39 | 150(20) | 109(14) | 160(20) | -12(16) | -33(19) | -39(13) |
| C79 | 220(20) | 300(30) | 180(20) | 20(20) | -10(19) | -190(20) |
| C5 | 220(40) | 140(30) | 360(60) | -70(30) | -70(40) | 20(30) |
| C81 | 103(17) | 71(10) | 79(14) | 2(10) | 24(12) | 3(11) |
| C41 | 100(20) | 53(14) | 116(19) | -3(14) | 56(18) | -17(15) |
| C83 | 70(11) | 58(10) | 47(7) | -7(7) | -6(7) | 1(8) |
| C21 | 40(7) | 74(11) | 72(11) | -5(9) | 4(7) | -12(7) |
| C85 | 138(19) | 51(10) | 54(6) | -1(7) | -27(9) | -38(11) |
| N1 | 270(30) | 130(20) | 59(7) | -13(11) | -32(12) | -70(20) |
| C43 | 50(7) | 60(11) | 145(19) | 41(12) | 38(10) | 4(7) |
| N2 | 53(7) | 150(20) | 300(40) | 80(20) | 46(14) | -5(11) |
| C87 | 170(30) | 100(20) | 180(30) | 50(20) | -40(20) | 0(20) |
| C11 | 126(14) | 128(19) | 130(20) | 50(18) | -19(16) | 32(14) |
| C89 | 169(11) | 139(9) | 120(7) | -62(7) | 28(8) | 3(9) |
| C45 | 169(11) | 139(9) | 120(7) | -62(7) | 28(8) | 3(9) |
| C91 | 169(11) | 139(9) | 120(7) | -62(7) | 28(8) | 3(9) |
| C23 | 140(30) | 89(16) | 220(30) | -50(20) | 100(20) | -41(16) |
| C93 | 109(19) | 86(16) | 140(15) | 9(14) | 49(14) | -25(17) |
| C47 | 190(40) | 160(40) | 170(20) | 50(30) | 50(20) | -30(30) |
| C95 | 200(40) | 100(20) | 250(50) | -20(30) | 40(40) | 0(20) |
| C3 | 134(19) | 95(13) | 170(20) | 14(15) | 21(18) | -21(13) |
| C97 | 139(19) | 240(50) | 230(50) | 40(50) | -30(30) | 50(30) |
| C49 | 180(30) | 160(30) | 170(30) | 90(20) | -30(30) | -20(30) |
| C99 | 124(17) | 98(17) | 113(17) | 48(15) | -41(13) | 15(14) |
| C107 | 163(6) | 221(7) | 91(4) | 36(5) | -9(3) | -46(6) |
| C108 | 163(6) | 221(7) | 91(4) | 36(5) | -9(3) | -46(6) |
| C109 | 163(6) | 221(7) | 91(4) | 36(5) | -9(3) | -46(6) |
| C110 | 163(6) | 221(7) | 91(4) | 36(5) | -9(3) | -46(6) |
| C112 | 163(6) | 221(7) | 91(4) | 36(5) | -9(3) | -46(6) |
| C111 | 163(6) | 221(7) | 91(4) | 36(5) | -9(3) | -46(6) |

Table 4 Bond Lengths for MJP104-3\_150K.

| Atom | Atom | Length/Å |  | Atom | Atom | Length/Å |
| --- | --- | --- | --- | --- | --- | --- |
| Nb1 | O1 | 1.868(5) |  | C14 | C30 | 1.496(11) |
| Nb1 | O12 | 1.896(5) |  | C16 | C24 | 1.478(10) |
| Nb1 | O18 | 1.899(5) |  | C18 | C22 | 1.497(11) |
| Nb1 | O30 | 1.993(5) |  | C20 | C34 | 1.511(11) |
| Nb1 | O34 | 2.172(5) |  | C22 | C82 | 1.376(14) |
| Nb1 | O36 | 2.132(6) |  | C22 | C94 | 1.379(15) |
| Nb7 | O4 | 1.863(4) |  | C24 | C42 | 1.362(13) |
| Nb7 | O16 | 1.914(5) |  | C24 | C50 | 1.365(13) |
| Nb7 | O20 | 1.903(5) |  | C26 | C54 | 1.383(12) |
| Nb7 | O261 | 2.144(5) |  | C28 | C36 | 1.494(11) |
| Nb7 | O28 | 1.992(5) |  | C30 | C92 | 1.384(15) |
| Nb7 | O211 | 2.170(5) |  | C30 | C98 | 1.352(15) |
| Nb4 | O8 | 1.909(5) |  | C32 | C72 | 1.371(12) |
| Nb4 | O18 | 1.916(5) |  | C34 | C70 | 1.360(15) |
| Nb4 | O242 | 1.900(5) |  | C34 | C80 | 1.373(16) |
| Nb4 | O5 | 2.150(5) |  | C36 | C78 | 1.400(15) |
| Nb4 | O29 | 2.148(6) |  | C36 | C25 | 1.374(15) |
| Nb4 | O17 | 1.934(6) |  | C38 | C58 | 1.368(12) |
| Nb3 | O6 | 1.902(5) |  | C40 | C48 | 1.368(13) |
| Nb3 | O8 | 1.908(5) |  | C42 | C84 | 1.406(13) |
| Nb3 | O122 | 1.905(5) |  | C44 | C86 | 1.410(12) |
| Nb3 | O32 | 2.142(6) |  | C46 | C60 | 1.377(13) |
| Nb3 | O15 | 2.152(5) |  | C48 | C52 | 1.363(14) |
| Nb3 | O2 | 1.933(6) |  | C50 | C68 | 1.378(12) |
| Nb2 | O1 | 1.935(5) |  | C52 | C54 | 1.358(15) |
| Nb2 | O6 | 1.905(5) |  | O9 | C64 | 1.143(13) |
| Nb2 | O24 | 1.911(5) |  | C56 | C58 | 1.371(16) |
| Nb2 | O38 | 2.144(5) |  | C56 | C86 | 1.403(17) |
| Nb2 | O19 | 1.912(6) |  | C60 | C62 | 1.375(14) |
| Nb2 | O7 | 2.151(5) |  | C62 | C72 | 1.363(15) |
| Nb6 | O10 | 1.900(5) |  | O37 | C74 | 1.146(13) |
| Nb6 | O141 | 1.903(5) |  | C64 | C57 | 1.601(16) |
| Nb6 | O20 | 1.904(5) |  | C64 | C101 | 1.520(13) |
| Nb6 | O11 | 2.147(5) |  | C66 | C68 | 1.373(16) |
| Nb6 | O23 | 2.149(6) |  | C66 | C84 | 1.380(16) |
| Nb6 | O33 | 1.935(6) |  | C70 | C7 | 1.367(14) |
| Nb8 | O14 | 1.909(5) |  | C74 | C107 | 1.547(12) |
| Nb8 | O16 | 1.891(5) |  | C76 | C9 | 1.36(2) |
| Nb8 | O22 | 1.904(5) |  | C76 | C19 | 1.32(2) |
| Nb8 | O271 | 2.150(5) |  | C78 | C51 | 1.398(15) |
| Nb8 | O311 | 2.141(6) |  | C80 | C69 | 1.400(14) |
| Nb8 | O35 | 1.927(6) |  | C82 | C27 | 1.390(16) |
| Nb5 | O41 | 1.940(5) |  | C88 | C7 | 1.39(2) |
| Nb5 | O10 | 1.914(5) |  | C88 | C69 | 1.35(2) |
| Nb5 | O22 | 1.901(5) |  | C90 | C100 | 1.323(18) |
| Nb5 | O3 | 2.147(5) |  | C92 | C19 | 1.382(16) |
| Nb5 | O25 | 1.906(6) |  | C94 | C71 | 1.398(14) |
| Nb5 | O13 | 2.146(5) |  | C96 | C13 | 1.340(15) |
| O26 | C4 | 1.265(10) |  | C98 | C9 | 1.398(15) |
| O28 | C74 | 1.304(10) |  | C25 | C35 | 1.396(15) |
| O30 | C64 | 1.290(10) |  | C51 | C55 | 1.41(2) |
| O32 | C20 | 1.247(10) |  | C53 | C27 | 1.33(2) |
| O34 | C1 | 1.258(9) |  | C53 | C71 | 1.37(2) |
| O36 | C12 | 1.270(10) |  | C55 | C35 | 1.31(2) |
| O38 | C1 | 1.271(9) |  | C57 | C29 | 1.3900 |
| O19 | C96 | 1.340(12) |  | C57 | C31 | 1.3900 |
| O5 | C20 | 1.269(10) |  | C29 | C59 | 1.3900 |
| O21 | C16 | 1.262(9) |  | C59 | C15 | 1.3900 |
| O11 | C28 | 1.266(10) |  | C15 | C61 | 1.3900 |
| O23 | C18 | 1.243(10) |  | C61 | C31 | 1.3900 |
| O3 | C4 | 1.276(9) |  | C73 | C91 | 1.44(3) |
| O25 | C90 | 1.404(14) |  | C37 | C79 | 1.34(3) |
| O25 | C81 | 1.398(17) |  | C75 | C23 | 1.45(4) |
| O13 | C16 | 1.274(9) |  | C77 | C87 | 1.63(4) |
| O27 | C18 | 1.278(10) |  | C39 | C5 | 1.36(3) |
| O7 | C12 | 1.267(9) |  | C81 | C41 | 1.32(2) |
| O29 | C14 | 1.250(10) |  | C83 | C85 | 1.36(2) |
| O15 | C14 | 1.269(10) |  | C21 | C43 | 1.33(2) |
| O31 | C28 | 1.237(10) |  | C85 | N1 | 1.06(3) |
| O2 | C75 | 1.446(19) |  | C43 | N2 | 1.07(3) |
| O2 | C93 | 1.460(19) |  | C11 | C97 | 1.64(4) |
| O2 | C3 | 1.45(2) |  | C89 | C45 | 1.45(3) |
| O33 | C37 | 1.441(15) |  | C93 | C47 | 1.46(3) |
| O33 | C39 | 1.45(2) |  | C95 | C3 | 1.45(4) |
| O17 | C73 | 1.432(19) |  | C49 | C99 | 1.64(4) |
| O17 | C45 | 1.436(19) |  | C101 | C102 | 1.3900 |
| O35 | C77 | 1.47(2) |  | C101 | C106 | 1.3900 |
| O35 | C11 | 1.46(2) |  | C102 | C103 | 1.3900 |
| O35 | C99 | 1.45(2) |  | C103 | C104 | 1.3900 |
| C1 | C8 | 1.483(10) |  | C104 | C105 | 1.3900 |
| C4 | C6 | 1.483(10) |  | C105 | C106 | 1.3900 |
| C6 | C26 | 1.375(11) |  | C107 | C108 | 1.3900 |
| C6 | C40 | 1.378(12) |  | C107 | C111 | 1.3900 |
| C8 | C38 | 1.357(12) |  | C108 | C109 | 1.3900 |
| C8 | C44 | 1.386(13) |  | C109 | C110 | 1.3900 |
| C10 | C12 | 1.481(10) |  | C110 | C112 | 1.3900 |
| C10 | C32 | 1.370(11) |  | C112 | C111 | 1.3900 |
| C10 | C46 | 1.374(13) |  |  |  |  |

11-X,1-Y,1-Z; 23/2-X,1/2-Y,3/2-Z

Table 5 Bond Angles for MJP104-3\_150K.

| Atom | Atom | Atom | Angle/˚ |  | Atom | Atom | Atom | Angle/˚ |
| --- | --- | --- | --- | --- | --- | --- | --- | --- |
| O1 | Nb1 | O12 | 96.4(2) |  | C12 | O7 | Nb2 | 136.7(5) |
| O1 | Nb1 | O18 | 97.2(2) |  | C14 | O29 | Nb4 | 136.5(5) |
| O1 | Nb1 | O30 | 161.7(2) |  | C14 | O15 | Nb3 | 135.7(6) |
| O1 | Nb1 | O34 | 83.87(19) |  | C28 | O31 | Nb81 | 137.1(5) |
| O1 | Nb1 | O36 | 84.4(2) |  | C75 | O2 | Nb3 | 145.0(16) |
| O12 | Nb1 | O18 | 96.0(2) |  | C93 | O2 | Nb3 | 141(2) |
| O12 | Nb1 | O30 | 94.0(2) |  | C3 | O2 | Nb3 | 156.8(19) |
| O12 | Nb1 | O34 | 90.0(2) |  | C37 | O33 | Nb6 | 141.2(10) |
| O12 | Nb1 | O36 | 172.7(2) |  | C39 | O33 | Nb6 | 149.8(19) |
| O18 | Nb1 | O30 | 96.5(2) |  | C73 | O17 | Nb4 | 137.1(14) |
| O18 | Nb1 | O34 | 173.7(2) |  | C45 | O17 | Nb4 | 146.5(12) |
| O18 | Nb1 | O36 | 91.0(2) |  | C77 | O35 | Nb8 | 132.8(16) |
| O30 | Nb1 | O34 | 81.2(2) |  | C11 | O35 | Nb8 | 144(2) |
| O30 | Nb1 | O36 | 83.4(2) |  | C99 | O35 | Nb8 | 145.4(18) |
| O36 | Nb1 | O34 | 82.9(2) |  | O34 | C1 | O38 | 125.1(6) |
| O4 | Nb7 | O16 | 96.3(2) |  | O34 | C1 | C8 | 117.4(7) |
| O4 | Nb7 | O20 | 97.0(2) |  | O38 | C1 | C8 | 117.5(7) |
| O4 | Nb7 | O261 | 84.4(2) |  | O26 | C4 | O3 | 124.1(7) |
| O4 | Nb7 | O28 | 162.0(2) |  | O26 | C4 | C6 | 117.0(7) |
| O4 | Nb7 | O211 | 83.89(19) |  | O3 | C4 | C6 | 118.9(8) |
| O16 | Nb7 | O261 | 173.0(2) |  | C26 | C6 | C4 | 119.9(8) |
| O16 | Nb7 | O28 | 94.2(2) |  | C26 | C6 | C40 | 118.8(8) |
| O16 | Nb7 | O211 | 90.0(2) |  | C40 | C6 | C4 | 121.2(7) |
| O20 | Nb7 | O16 | 96.1(2) |  | C38 | C8 | C1 | 121.8(8) |
| O20 | Nb7 | O261 | 90.7(2) |  | C38 | C8 | C44 | 118.3(8) |
| O20 | Nb7 | O28 | 96.4(2) |  | C44 | C8 | C1 | 119.9(8) |
| O20 | Nb7 | O211 | 173.6(2) |  | C32 | C10 | C12 | 120.8(8) |
| O261 | Nb7 | O211 | 83.1(2) |  | C32 | C10 | C46 | 118.7(8) |
| O28 | Nb7 | O261 | 83.4(2) |  | C46 | C10 | C12 | 120.4(7) |
| O28 | Nb7 | O211 | 81.5(2) |  | O36 | C12 | C10 | 116.6(7) |
| O8 | Nb4 | O18 | 94.3(2) |  | O7 | C12 | O36 | 124.9(7) |
| O8 | Nb4 | O5 | 83.4(2) |  | O7 | C12 | C10 | 118.5(8) |
| O8 | Nb4 | O29 | 83.6(2) |  | O29 | C14 | O15 | 125.5(7) |
| O8 | Nb4 | O17 | 162.4(2) |  | O29 | C14 | C30 | 117.9(8) |
| O18 | Nb4 | O5 | 173.3(2) |  | O15 | C14 | C30 | 116.5(9) |
| O18 | Nb4 | O29 | 89.4(2) |  | O21 | C16 | O13 | 124.8(7) |
| O18 | Nb4 | O17 | 97.4(2) |  | O21 | C16 | C24 | 118.1(7) |
| O242 | Nb4 | O8 | 94.7(2) |  | O13 | C16 | C24 | 117.1(7) |
| O242 | Nb4 | O18 | 96.5(2) |  | O23 | C18 | O27 | 125.3(7) |
| O242 | Nb4 | O5 | 90.0(2) |  | O23 | C18 | C22 | 118.3(8) |
| O242 | Nb4 | O29 | 174.0(2) |  | O27 | C18 | C22 | 116.4(8) |
| O242 | Nb4 | O17 | 97.0(3) |  | O32 | C20 | O5 | 124.5(7) |
| O29 | Nb4 | O5 | 84.1(2) |  | O32 | C20 | C34 | 117.3(8) |
| O17 | Nb4 | O5 | 83.5(2) |  | O5 | C20 | C34 | 118.2(8) |
| O17 | Nb4 | O29 | 83.4(3) |  | C82 | C22 | C18 | 119.7(10) |
| O6 | Nb3 | O8 | 94.3(2) |  | C82 | C22 | C94 | 119.9(10) |
| O6 | Nb3 | O122 | 96.9(2) |  | C94 | C22 | C18 | 120.4(9) |
| O6 | Nb3 | O32 | 172.5(2) |  | C42 | C24 | C16 | 120.4(8) |
| O6 | Nb3 | O15 | 88.8(2) |  | C42 | C24 | C50 | 118.5(8) |
| O6 | Nb3 | O2 | 96.4(3) |  | C50 | C24 | C16 | 121.1(8) |
| O8 | Nb3 | O32 | 83.9(2) |  | C6 | C26 | C54 | 120.1(9) |
| O8 | Nb3 | O15 | 83.7(2) |  | O11 | C28 | C36 | 117.0(8) |
| O8 | Nb3 | O2 | 163.4(2) |  | O31 | C28 | O11 | 124.7(7) |
| O122 | Nb3 | O8 | 93.8(2) |  | O31 | C28 | C36 | 118.3(8) |
| O122 | Nb3 | O32 | 90.5(2) |  | C92 | C30 | C14 | 119.4(10) |
| O122 | Nb3 | O15 | 173.9(2) |  | C98 | C30 | C14 | 120.5(9) |
| O122 | Nb3 | O2 | 97.5(2) |  | C98 | C30 | C92 | 120.0(10) |
| O32 | Nb3 | O15 | 83.8(2) |  | C10 | C32 | C72 | 121.2(9) |
| O2 | Nb3 | O32 | 83.8(3) |  | C70 | C34 | C20 | 120.1(10) |
| O2 | Nb3 | O15 | 83.9(2) |  | C70 | C34 | C80 | 120.5(9) |
| O1 | Nb2 | O38 | 82.5(2) |  | C80 | C34 | C20 | 119.3(9) |
| O1 | Nb2 | O7 | 83.0(2) |  | C78 | C36 | C28 | 118.7(10) |
| O6 | Nb2 | O1 | 93.0(2) |  | C25 | C36 | C28 | 121.5(9) |
| O6 | Nb2 | O24 | 96.3(2) |  | C25 | C36 | C78 | 119.7(10) |
| O6 | Nb2 | O38 | 171.4(2) |  | C8 | C38 | C58 | 123.2(10) |
| O6 | Nb2 | O19 | 97.4(2) |  | C48 | C40 | C6 | 120.4(9) |
| O6 | Nb2 | O7 | 90.3(2) |  | C24 | C42 | C84 | 121.4(11) |
| O24 | Nb2 | O1 | 92.3(2) |  | C8 | C44 | C86 | 121.1(10) |
| O24 | Nb2 | O38 | 91.2(2) |  | C10 | C46 | C60 | 119.9(9) |
| O24 | Nb2 | O19 | 97.4(2) |  | C52 | C48 | C40 | 120.5(10) |
| O24 | Nb2 | O7 | 172.1(2) |  | C24 | C50 | C68 | 122.1(10) |
| O38 | Nb2 | O7 | 81.9(2) |  | C54 | C52 | C48 | 119.8(9) |
| O19 | Nb2 | O1 | 164.8(2) |  | C52 | C54 | C26 | 120.3(9) |
| O19 | Nb2 | O38 | 85.7(2) |  | C58 | C56 | C86 | 121.4(9) |
| O19 | Nb2 | O7 | 86.0(2) |  | C38 | C58 | C56 | 118.6(10) |
| O10 | Nb6 | O141 | 94.7(2) |  | C62 | C60 | C46 | 120.7(10) |
| O10 | Nb6 | O20 | 96.9(2) |  | C72 | C62 | C60 | 119.0(9) |
| O10 | Nb6 | O11 | 89.7(2) |  | O30 | C64 | C57 | 109.6(15) |
| O10 | Nb6 | O23 | 173.7(2) |  | O30 | C64 | C101 | 114.9(11) |
| O10 | Nb6 | O33 | 97.2(3) |  | O9 | C64 | O30 | 124.3(11) |
| O141 | Nb6 | O20 | 94.2(2) |  | O9 | C64 | C57 | 124.1(15) |
| O141 | Nb6 | O11 | 83.2(2) |  | O9 | C64 | C101 | 120.5(11) |
| O141 | Nb6 | O23 | 83.5(2) |  | C68 | C66 | C84 | 120.9(9) |
| O141 | Nb6 | O33 | 162.1(2) |  | C66 | C68 | C50 | 118.9(11) |
| O20 | Nb6 | O11 | 173.0(2) |  | C34 | C70 | C7 | 120.8(14) |
| O20 | Nb6 | O23 | 89.3(2) |  | C62 | C72 | C32 | 120.2(10) |
| O20 | Nb6 | O33 | 97.4(2) |  | O28 | C74 | C107 | 112.2(9) |
| O11 | Nb6 | O23 | 84.0(2) |  | O37 | C74 | O28 | 125.1(11) |
| O33 | Nb6 | O11 | 83.6(2) |  | O37 | C74 | C107 | 121.8(10) |
| O33 | Nb6 | O23 | 83.2(3) |  | C19 | C76 | C9 | 119.9(13) |
| O14 | Nb8 | O271 | 83.6(2) |  | C51 | C78 | C36 | 118.0(13) |
| O14 | Nb8 | O311 | 83.7(2) |  | C34 | C80 | C69 | 119.3(13) |
| O14 | Nb8 | O35 | 163.4(2) |  | C22 | C82 | C27 | 120.3(14) |
| O16 | Nb8 | O14 | 94.0(2) |  | C66 | C84 | C42 | 118.1(11) |
| O16 | Nb8 | O22 | 96.5(2) |  | C56 | C86 | C44 | 117.3(11) |
| O16 | Nb8 | O271 | 173.8(2) |  | C69 | C88 | C7 | 120.9(12) |
| O16 | Nb8 | O311 | 90.7(2) |  | C100 | C90 | O25 | 117(2) |
| O16 | Nb8 | O35 | 97.5(2) |  | C19 | C92 | C30 | 119.5(14) |
| O22 | Nb8 | O14 | 94.6(2) |  | C22 | C94 | C71 | 118.3(12) |
| O22 | Nb8 | O271 | 89.4(2) |  | C13 | C96 | O19 | 123.3(11) |
| O22 | Nb8 | O311 | 172.8(2) |  | C30 | C98 | C9 | 118.5(13) |
| O22 | Nb8 | O35 | 96.0(3) |  | C36 | C25 | C35 | 120.2(13) |
| O311 | Nb8 | O271 | 83.4(2) |  | C78 | C51 | C55 | 120.9(13) |
| O35 | Nb8 | O271 | 83.7(2) |  | C27 | C53 | C71 | 120.3(13) |
| O35 | Nb8 | O311 | 84.2(3) |  | C53 | C27 | C82 | 120.3(15) |
| O41 | Nb5 | O3 | 83.0(2) |  | C35 | C55 | C51 | 119.3(12) |
| O41 | Nb5 | O13 | 82.9(2) |  | C70 | C7 | C88 | 118.8(14) |
| O10 | Nb5 | O41 | 92.2(2) |  | C29 | C57 | C64 | 119(2) |
| O10 | Nb5 | O3 | 171.6(2) |  | C29 | C57 | C31 | 120.0 |
| O10 | Nb5 | O13 | 90.5(2) |  | C31 | C57 | C64 | 121(2) |
| O22 | Nb5 | O41 | 93.0(2) |  | C59 | C29 | C57 | 120.0 |
| O22 | Nb5 | O10 | 96.9(2) |  | C29 | C59 | C15 | 120.0 |
| O22 | Nb5 | O3 | 90.3(2) |  | C61 | C15 | C59 | 120.0 |
| O22 | Nb5 | O25 | 97.3(2) |  | C15 | C61 | C31 | 120.0 |
| O22 | Nb5 | O13 | 171.7(2) |  | C61 | C31 | C57 | 120.0 |
| O25 | Nb5 | O41 | 164.9(2) |  | C88 | C69 | C80 | 119.5(15) |
| O25 | Nb5 | O10 | 97.4(2) |  | C55 | C35 | C25 | 121.8(16) |
| O25 | Nb5 | O3 | 86.0(2) |  | C53 | C71 | C94 | 120.9(15) |
| O25 | Nb5 | O13 | 85.4(2) |  | C76 | C9 | C98 | 121.0(16) |
| O13 | Nb5 | O3 | 82.1(2) |  | O17 | C73 | C91 | 132(2) |
| Nb1 | O1 | Nb2 | 146.4(3) |  | C79 | C37 | O33 | 117(2) |
| Nb7 | O4 | Nb51 | 146.2(2) |  | O2 | C75 | C23 | 108(2) |
| Nb3 | O6 | Nb2 | 148.0(3) |  | C76 | C19 | C92 | 121.0(15) |
| Nb3 | O8 | Nb4 | 144.9(3) |  | O35 | C77 | C87 | 96(2) |
| Nb6 | O10 | Nb5 | 148.4(3) |  | C5 | C39 | O33 | 114(3) |
| Nb1 | O12 | Nb32 | 145.7(3) |  | C41 | C81 | O25 | 126(3) |
| Nb61 | O14 | Nb8 | 145.2(3) |  | N1 | C85 | C83 | 176(3) |
| Nb8 | O16 | Nb7 | 145.8(3) |  | N2 | C43 | C21 | 174(4) |
| Nb1 | O18 | Nb4 | 144.3(3) |  | O35 | C11 | C97 | 157(5) |
| Nb7 | O20 | Nb6 | 144.4(3) |  | O17 | C45 | C89 | 106(2) |
| Nb5 | O22 | Nb8 | 147.6(3) |  | O2 | C93 | C47 | 157(4) |
| Nb42 | O24 | Nb2 | 148.3(3) |  | O2 | C3 | C95 | 104(3) |
| C4 | O26 | Nb71 | 136.4(5) |  | O35 | C99 | C49 | 99(3) |
| C74 | O28 | Nb7 | 144.6(6) |  | C102 | C101 | C64 | 116.4(12) |
| C64 | O30 | Nb1 | 145.1(7) |  | C102 | C101 | C106 | 120.0 |
| C20 | O32 | Nb3 | 136.9(5) |  | C106 | C101 | C64 | 123.2(12) |
| C1 | O34 | Nb1 | 134.7(5) |  | C101 | C102 | C103 | 120.0 |
| C12 | O36 | Nb1 | 136.2(5) |  | C104 | C103 | C102 | 120.0 |
| C1 | O38 | Nb2 | 137.8(5) |  | C105 | C104 | C103 | 120.0 |
| C96 | O19 | Nb2 | 156.6(7) |  | C104 | C105 | C106 | 120.0 |
| C20 | O5 | Nb4 | 136.7(5) |  | C105 | C106 | C101 | 120.0 |
| C16 | O21 | Nb71 | 135.2(5) |  | C108 | C107 | C74 | 115.5(8) |
| C28 | O11 | Nb6 | 136.7(5) |  | C108 | C107 | C111 | 120.0 |
| C18 | O23 | Nb6 | 136.7(5) |  | C111 | C107 | C74 | 123.8(7) |
| C4 | O3 | Nb5 | 137.3(5) |  | C109 | C108 | C107 | 120.0 |
| C90 | O25 | Nb5 | 152.4(9) |  | C110 | C109 | C108 | 120.0 |
| C81 | O25 | Nb5 | 152.6(13) |  | C109 | C110 | C112 | 120.0 |
| C16 | O13 | Nb5 | 137.4(5) |  | C111 | C112 | C110 | 120.0 |
| C18 | O27 | Nb81 | 135.7(6) |  | C112 | C111 | C107 | 120.0 |

11-X,1-Y,1-Z; 23/2-X,1/2-Y,3/2-Z

Table 6 Torsion Angles for MJP104-3\_150K.

| A | B | C | D | Angle/˚ |  | A | B | C | D | Angle/˚ |
| --- | --- | --- | --- | --- | --- | --- | --- | --- | --- | --- |
| Nb1 | O30 | C64 | O9 | 102.5(15) |  | O15 | C14 | C30 | C98 | 2.4(11) |
| Nb1 | O30 | C64 | C57 | -93.2(18) |  | O311 | Nb8 | O16 | Nb7 | -88.4(5) |
| Nb1 | O30 | C64 | C101 | -83.3(16) |  | O31 | C28 | C36 | C78 | 7.7(12) |
| Nb1 | O34 | C1 | O38 | -6.4(12) |  | O31 | C28 | C36 | C25 | -170.9(9) |
| Nb1 | O34 | C1 | C8 | 173.2(5) |  | O33 | Nb6 | O10 | Nb5 | -173.5(5) |
| Nb1 | O36 | C12 | O7 | 3.5(12) |  | O35 | Nb8 | O16 | Nb7 | -172.7(5) |
| Nb1 | O36 | C12 | C10 | -175.0(5) |  | C1 | C8 | C38 | C58 | -178.9(8) |
| Nb71 | O26 | C4 | O3 | 3.8(13) |  | C1 | C8 | C44 | C86 | 178.2(9) |
| Nb71 | O26 | C4 | C6 | -175.5(5) |  | C4 | C6 | C26 | C54 | 179.8(8) |
| Nb7 | O28 | C74 | O37 | -103.5(15) |  | C4 | C6 | C40 | C48 | -178.8(9) |
| Nb7 | O28 | C74 | C107 | 87.8(14) |  | C6 | C26 | C54 | C52 | -0.1(15) |
| Nb71 | O21 | C16 | O13 | -5.4(12) |  | C6 | C40 | C48 | C52 | -1.7(16) |
| Nb71 | O21 | C16 | C24 | 173.2(5) |  | C8 | C38 | C58 | C56 | -0.2(16) |
| Nb4 | O5 | C20 | O32 | -6.1(14) |  | C8 | C44 | C86 | C56 | 1.5(16) |
| Nb4 | O5 | C20 | C34 | 175.9(5) |  | C10 | C32 | C72 | C62 | 0.6(15) |
| Nb4 | O29 | C14 | O15 | 7.7(13) |  | C10 | C46 | C60 | C62 | -1.5(16) |
| Nb4 | O29 | C14 | C30 | -170.9(5) |  | C12 | C10 | C32 | C72 | -179.7(8) |
| Nb4 | O17 | C73 | C91 | -99(4) |  | C12 | C10 | C46 | C60 | -179.9(9) |
| Nb4 | O17 | C45 | C89 | -176.5(15) |  | C14 | C30 | C92 | C19 | 179.9(9) |
| Nb3 | O32 | C20 | O5 | 8.2(14) |  | C14 | C30 | C98 | C9 | 179.9(9) |
| Nb3 | O32 | C20 | C34 | -173.8(5) |  | C16 | C24 | C42 | C84 | 176.7(8) |
| Nb3 | O15 | C14 | O29 | -6.7(13) |  | C16 | C24 | C50 | C68 | -179.3(8) |
| Nb3 | O15 | C14 | C30 | 172.0(5) |  | C18 | C22 | C82 | C27 | -179.8(10) |
| Nb3 | O2 | C75 | C23 | -168(3) |  | C18 | C22 | C94 | C71 | 178.7(9) |
| Nb3 | O2 | C93 | C47 | 56(13) |  | C20 | C34 | C70 | C7 | -179.2(9) |
| Nb3 | O2 | C3 | C95 | 36(8) |  | C20 | C34 | C80 | C69 | -178.2(10) |
| Nb2 | O38 | C1 | O34 | 3.7(13) |  | C22 | C82 | C27 | C53 | 1(2) |
| Nb2 | O38 | C1 | C8 | -175.9(5) |  | C22 | C94 | C71 | C53 | 1.1(18) |
| Nb2 | O19 | C96 | C13 | -28(4) |  | C24 | C42 | C84 | C66 | 3.6(16) |
| Nb2 | O7 | C12 | O36 | -5.4(12) |  | C24 | C50 | C68 | C66 | 1.4(16) |
| Nb2 | O7 | C12 | C10 | 173.1(5) |  | C26 | C6 | C40 | C48 | -1.1(14) |
| Nb6 | O11 | C28 | O31 | 5.3(13) |  | C28 | C36 | C78 | C51 | 179.6(9) |
| Nb6 | O11 | C28 | C36 | -175.5(5) |  | C28 | C36 | C25 | C35 | 177.6(10) |
| Nb6 | O23 | C18 | O27 | -7.4(13) |  | C30 | C92 | C19 | C76 | -1.0(19) |
| Nb6 | O23 | C18 | C22 | 171.6(5) |  | C30 | C98 | C9 | C76 | 1.3(18) |
| Nb6 | O33 | C37 | C79 | 153(2) |  | C32 | C10 | C12 | O36 | 0.3(11) |
| Nb6 | O33 | C39 | C5 | -43(7) |  | C32 | C10 | C12 | O7 | -178.3(7) |
| Nb81 | O27 | C18 | O23 | 7.4(13) |  | C32 | C10 | C46 | C60 | -1.2(14) |
| Nb81 | O27 | C18 | C22 | -171.6(5) |  | C34 | C70 | C7 | C88 | -3.7(18) |
| Nb81 | O31 | C28 | O11 | -7.1(14) |  | C34 | C80 | C69 | C88 | -1.4(19) |
| Nb81 | O31 | C28 | C36 | 173.8(5) |  | C36 | C78 | C51 | C55 | 2.4(17) |
| Nb8 | O35 | C77 | C87 | -169(2) |  | C36 | C25 | C35 | C55 | 3(2) |
| Nb8 | O35 | C11 | C97 | -11(15) |  | C38 | C8 | C44 | C86 | -2.0(14) |
| Nb8 | O35 | C99 | C49 | -143(3) |  | C40 | C6 | C26 | C54 | 2.0(13) |
| Nb5 | O3 | C4 | O26 | -5.3(13) |  | C40 | C48 | C52 | C54 | 3.6(17) |
| Nb5 | O3 | C4 | C6 | 174.0(5) |  | C42 | C24 | C50 | C68 | 0.3(14) |
| Nb5 | O25 | C90 | C100 | -53(3) |  | C44 | C8 | C38 | C58 | 1.3(14) |
| Nb5 | O25 | C81 | C41 | 49(5) |  | C46 | C10 | C12 | O36 | 179.0(8) |
| Nb5 | O13 | C16 | O21 | 2.8(12) |  | C46 | C10 | C12 | O7 | 0.4(11) |
| Nb5 | O13 | C16 | C24 | -175.9(5) |  | C46 | C10 | C32 | C72 | 1.6(13) |
| O1 | Nb1 | O12 | Nb32 | -6.4(5) |  | C46 | C60 | C62 | C72 | 3.7(16) |
| O1 | Nb1 | O18 | Nb4 | 8.9(5) |  | C48 | C52 | C54 | C26 | -2.7(16) |
| O12 | Nb1 | O1 | Nb2 | -132.9(5) |  | C50 | C24 | C42 | C84 | -2.9(14) |
| O12 | Nb1 | O18 | Nb4 | -88.4(5) |  | O9 | C64 | C57 | C29 | -18(3) |
| O141 | Nb6 | O10 | Nb5 | -6.8(5) |  | O9 | C64 | C57 | C31 | 170.1(15) |
| O14 | Nb8 | O16 | Nb7 | -4.7(5) |  | O9 | C64 | C101 | C102 | -18.8(19) |
| O16 | Nb7 | O4 | Nb51 | 132.8(5) |  | O9 | C64 | C101 | C106 | 168.8(12) |
| O18 | Nb1 | O1 | Nb2 | 130.1(5) |  | C58 | C56 | C86 | C44 | -0.4(17) |
| O18 | Nb1 | O12 | Nb32 | 91.6(5) |  | C60 | C62 | C72 | C32 | -3.3(15) |
| O20 | Nb7 | O4 | Nb51 | -130.3(5) |  | O37 | C74 | C107 | C108 | 19.7(17) |
| O20 | Nb6 | O10 | Nb5 | 88.0(5) |  | O37 | C74 | C107 | C111 | -170.1(12) |
| O22 | Nb8 | O16 | Nb7 | 90.4(5) |  | C64 | C57 | C29 | C59 | -172(3) |
| O261 | Nb7 | O4 | Nb51 | -40.2(5) |  | C64 | C57 | C31 | C61 | 172(3) |
| O26 | C4 | C6 | C26 | 1.8(11) |  | C64 | C101 | C102 | C103 | -172.7(15) |
| O26 | C4 | C6 | C40 | 179.5(8) |  | C64 | C101 | C106 | C105 | 172.2(15) |
| O28 | Nb7 | O4 | Nb51 | 7.6(10) |  | C68 | C66 | C84 | C42 | -1.8(17) |
| O28 | C74 | C107 | C108 | -171.2(7) |  | C70 | C34 | C80 | C69 | 0.0(16) |
| O28 | C74 | C107 | C111 | -1.0(13) |  | C74 | C107 | C108 | C109 | 170.6(10) |
| O30 | Nb1 | O1 | Nb2 | -8.5(10) |  | C74 | C107 | C111 | C112 | -169.8(11) |
| O30 | Nb1 | O12 | Nb32 | -171.4(5) |  | C78 | C36 | C25 | C35 | -1.0(17) |
| O30 | Nb1 | O18 | Nb4 | 176.9(5) |  | C78 | C51 | C55 | C35 | 0(2) |
| O30 | C64 | C57 | C29 | 177.9(15) |  | C80 | C34 | C70 | C7 | 2.6(16) |
| O30 | C64 | C57 | C31 | 6(2) |  | C82 | C22 | C94 | C71 | -1.1(15) |
| O30 | C64 | C101 | C102 | 166.8(9) |  | C84 | C66 | C68 | C50 | -0.6(17) |
| O30 | C64 | C101 | C106 | -5.6(16) |  | C86 | C56 | C58 | C38 | -0.3(17) |
| O32 | C20 | C34 | C70 | -6.7(12) |  | C92 | C30 | C98 | C9 | -0.8(15) |
| O32 | C20 | C34 | C80 | 171.5(9) |  | C94 | C22 | C82 | C27 | -0.1(15) |
| O34 | Nb1 | O1 | Nb2 | -43.7(5) |  | C98 | C30 | C92 | C19 | 0.6(15) |
| O34 | Nb1 | O12 | Nb32 | -90.2(5) |  | C25 | C36 | C78 | C51 | -1.7(15) |
| O34 | C1 | C8 | C38 | 9.7(11) |  | C51 | C55 | C35 | C25 | -3(2) |
| O34 | C1 | C8 | C44 | -170.5(8) |  | C27 | C53 | C71 | C94 | 0(2) |
| O36 | Nb1 | O1 | Nb2 | 39.8(5) |  | C7 | C88 | C69 | C80 | 0(2) |
| O36 | Nb1 | O18 | Nb4 | 93.4(5) |  | C57 | C29 | C59 | C15 | 0.0 |
| O38 | C1 | C8 | C38 | -170.7(8) |  | C29 | C57 | C31 | C61 | 0.0 |
| O38 | C1 | C8 | C44 | 9.1(11) |  | C29 | C59 | C15 | C61 | 0.0 |
| O5 | C20 | C34 | C70 | 171.4(8) |  | C59 | C15 | C61 | C31 | 0.0 |
| O5 | C20 | C34 | C80 | -10.4(12) |  | C15 | C61 | C31 | C57 | 0.0 |
| O211 | Nb7 | O4 | Nb51 | 43.4(5) |  | C31 | C57 | C29 | C59 | 0.0 |
| O21 | C16 | C24 | C42 | -170.0(8) |  | C69 | C88 | C7 | C70 | 2(2) |
| O21 | C16 | C24 | C50 | 9.6(11) |  | C71 | C53 | C27 | C82 | -1(2) |
| O11 | Nb6 | O10 | Nb5 | -90.0(5) |  | C9 | C76 | C19 | C92 | 2(2) |
| O11 | C28 | C36 | C78 | -171.5(8) |  | C19 | C76 | C9 | C98 | -2(2) |
| O11 | C28 | C36 | C25 | 9.9(13) |  | C101 | C102 | C103 | C104 | 0.0 |
| O23 | C18 | C22 | C82 | -1.6(12) |  | C102 | C101 | C106 | C105 | 0.0 |
| O23 | C18 | C22 | C94 | 178.6(8) |  | C102 | C103 | C104 | C105 | 0.0 |
| O3 | C4 | C6 | C26 | -177.5(7) |  | C103 | C104 | C105 | C106 | 0.0 |
| O3 | C4 | C6 | C40 | 0.2(12) |  | C104 | C105 | C106 | C101 | 0.0 |
| O13 | C16 | C24 | C42 | 8.7(11) |  | C106 | C101 | C102 | C103 | 0.0 |
| O13 | C16 | C24 | C50 | -171.7(8) |  | C107 | C108 | C109 | C110 | 0.0 |
| O27 | C18 | C22 | C82 | 177.5(8) |  | C108 | C107 | C111 | C112 | 0.0 |
| O27 | C18 | C22 | C94 | -2.3(11) |  | C108 | C109 | C110 | C112 | 0.0 |
| O29 | C14 | C30 | C92 | 1.9(11) |  | C109 | C110 | C112 | C111 | 0.0 |
| O29 | C14 | C30 | C98 | -178.8(8) |  | C110 | C112 | C111 | C107 | 0.0 |
| O15 | C14 | C30 | C92 | -176.9(8) |  | C111 | C107 | C108 | C109 | 0.0 |

11-X,1-Y,1-Z; 23/2-X,1/2-Y,3/2-Z

Table 7 Hydrogen Atom Coordinates (Å×104) and Isotropic Displacement Parameters (Å2×103) for MJP104-3\_150K.

| Atom | *x* | *y* | *z* | U(eq) |
| --- | --- | --- | --- | --- |
| H26 | 3748.95 | 5703.59 | 5965.41 | 92 |
| H32 | 6536.96 | 3211.34 | 6246.64 | 89 |
| H38 | 5937.68 | 3221.49 | 7536.38 | 95 |
| H40 | 3902.98 | 9071.84 | 5582.88 | 98 |
| H42 | 5059.86 | 9154.45 | 6214.23 | 105 |
| H44 | 6285.13 | 6674.39 | 7553.99 | 100 |
| H46 | 6913.03 | 6571.09 | 6411.9 | 101 |
| H48 | 3422.41 | 9743.36 | 5772.58 | 112 |
| H50 | 5031.85 | 5716.09 | 6565.03 | 100 |
| H52 | 3089.39 | 8372.34 | 6027.36 | 106 |
| H54 | 3259.65 | 6384.6 | 6142.38 | 104 |
| H56 | 5361.75 | 6047.04 | 7736.86 | 119 |
| H58 | 5442.89 | 3930.49 | 7666.61 | 109 |
| H60 | 6725.85 | 7251.11 | 5927.49 | 112 |
| H62 | 6473.02 | 5876.02 | 5588.05 | 102 |
| H66 | 5233.39 | 8571.67 | 7138.61 | 117 |
| H68 | 5170.54 | 6446.43 | 7060.53 | 112 |
| H70 | 8866.44 | 2942.92 | 6702.06 | 120 |
| H72 | 6357.13 | 3883.39 | 5761.95 | 107 |
| H76 | 7217.5 | 4893.45 | 5328.2 | 161 |
| H78 | 5800.51 | 4533.08 | 6371.34 | 120 |
| H80 | 8533.56 | -542.65 | 6643.69 | 127 |
| H82 | 6543.26 | 5343.03 | 4800.98 | 127 |
| H84 | 5163.72 | 9962.42 | 6720.95 | 121 |
| H86 | 5780.27 | 7472.49 | 7678.22 | 126 |
| H88 | 9446.88 | 142.68 | 6462.53 | 149 |
| H90A | 4765.83 | 11187.23 | 5386.6 | 99 |
| H90B | 5024.73 | 10618.53 | 5613 | 99 |
| H92 | 7298.37 | 2153.43 | 5957.72 | 129 |
| H94 | 6365.53 | 2022.61 | 5207.78 | 119 |
| H96A | 7167.42 | 8576.19 | 7191.52 | 173 |
| H96B | 6927.42 | 8267.09 | 7466.02 | 173 |
| H98 | 7696.98 | 5471.72 | 6138.21 | 121 |
| H10A | 4445.27 | 9985.19 | 5739.25 | 182 |
| H10B | 4529.44 | 11427.27 | 5779.76 | 182 |
| H10C | 4727.96 | 10396.31 | 5965.26 | 182 |
| H25 | 5858.63 | 8055.44 | 6034.75 | 131 |
| H51 | 5958.94 | 5289 | 6867.72 | 144 |
| H13A | 6848.51 | 8230.3 | 6844.61 | 206 |
| H13B | 6646.26 | 8894.22 | 7114.63 | 206 |
| H13C | 6627.84 | 7417.65 | 7070.4 | 206 |
| H53 | 7173.45 | 2598.45 | 4721.18 | 165 |
| H27 | 7028.97 | 4602.09 | 4625.35 | 159 |
| H55 | 6043.59 | 7434.23 | 6945.33 | 151 |
| H7 | 9358.7 | 2276.19 | 6537.54 | 148 |
| H29 | 5567.07 | 1011.12 | 7005.04 | 144 |
| H59 | 5266.13 | 477.29 | 7446.6 | 144 |
| H15 | 5514.57 | -110.61 | 7915.49 | 144 |
| H61 | 6063.96 | -164.68 | 7942.83 | 144 |
| H31 | 6364.91 | 369.15 | 7501.27 | 144 |
| H69 | 9041.59 | -1259.33 | 6513.68 | 163 |
| H35 | 5974.42 | 8768.49 | 6540.91 | 160 |
| H71 | 6849.38 | 1296.98 | 5010.55 | 151 |
| H9 | 7514.86 | 6172.39 | 5646.13 | 154 |
| H73A | 7807.38 | -1406.36 | 6266.33 | 171 |
| H73B | 7878.08 | 38.74 | 6247.83 | 171 |
| H37A | 6217.25 | 8536.07 | 5416.5 | 140 |
| H37B | 6362.81 | 7388.23 | 5228.72 | 140 |
| H75A | 8450.31 | 5525.12 | 6491.03 | 126 |
| H75B | 8672.54 | 5084.73 | 6776.05 | 126 |
| H19 | 7121.96 | 2904.29 | 5474.52 | 156 |
| H77A | 4651.18 | 9488.19 | 4244.22 | 173 |
| H77B | 4372.83 | 9537.15 | 4505.55 | 173 |
| H39A | 6174.32 | 8852.23 | 5404.36 | 166 |
| H39B | 6202.56 | 9354.89 | 5050.87 | 166 |
| H79A | 6446.4 | 8551.88 | 4828.42 | 344 |
| H79B | 6314.6 | 9749.88 | 4992.72 | 344 |
| H79C | 6608.5 | 9067.38 | 5133.52 | 344 |
| H5A | 5734.49 | 10042.52 | 5068.06 | 360 |
| H5B | 5668.69 | 9594.92 | 5413.36 | 360 |
| H5C | 5923.59 | 10618.62 | 5349.56 | 360 |
| H81A | 4508.18 | 10668.79 | 5254.58 | 101 |
| H81B | 4818.28 | 11143.79 | 5423.28 | 101 |
| H41A | 4275.3 | 10862.08 | 5637.23 | 135 |
| H41B | 4582.78 | 11466.16 | 5791.57 | 135 |
| H41C | 4517.53 | 10007.08 | 5827.89 | 135 |
| H83A | 3186.91 | 10488.75 | 3613.18 | 88 |
| H83B | 3059.54 | 9098.92 | 3657.5 | 88 |
| H83C | 3429.26 | 9351.52 | 3628.39 | 88 |
| H21A | 6119.41 | 6885.39 | 4076.49 | 93 |
| H21B | 6121.16 | 8004.44 | 4325.21 | 93 |
| H21C | 6167.08 | 6602.9 | 4443.69 | 93 |
| H87A | 4256.98 | 9173.45 | 3825.7 | 221 |
| H87B | 3986.98 | 9113.45 | 4077 | 221 |
| H87C | 4148.78 | 10387.45 | 3999.4 | 221 |
| H11A | 4179.49 | 8386.01 | 3944.34 | 155 |
| H11B | 4171.09 | 9313.31 | 4234.44 | 155 |
| H89A | 7385.11 | -2211.27 | 6286.96 | 214 |
| H89B | 7472.71 | -1199.17 | 6035.96 | 214 |
| H89C | 7667.11 | -2435.47 | 6056.36 | 214 |
| H45A | 7676.73 | -2167.66 | 6627.89 | 171 |
| H45B | 7945.71 | -1458.65 | 6430.87 | 171 |
| H91A | 7716.32 | -839.14 | 5794.15 | 214 |
| H91B | 7544.78 | 415.9 | 5901.84 | 214 |
| H91C | 7372.48 | -895.58 | 5946.89 | 214 |
| H23A | 8805.27 | 7040.52 | 6754.44 | 223 |
| H23B | 8519 | 7140.18 | 6998.05 | 223 |
| H23C | 8465.7 | 7471.99 | 6634.29 | 223 |
| H93A | 8531.77 | 5118.42 | 6599.19 | 134 |
| H93B | 8487.12 | 6485.66 | 6710.53 | 134 |
| H47A | 8601.21 | 6067.96 | 6206.33 | 260 |
| H47B | 8338.25 | 7094.83 | 6277.87 | 260 |
| H47C | 8237.18 | 5738.8 | 6159.78 | 260 |
| H95A | 7868.03 | 6977.18 | 6782.67 | 277 |
| H95B | 8017.63 | 7374.08 | 7104.97 | 277 |
| H95C | 8075.63 | 8179.88 | 6802.47 | 277 |
| H3A | 8295.98 | 6495.27 | 6532.98 | 161 |
| H3B | 8505.83 | 6831.54 | 6837.64 | 161 |
| H97A | 3698.65 | 8716.56 | 3881.19 | 305 |
| H97B | 3640.35 | 8009.07 | 4198.79 | 305 |
| H97C | 3682.75 | 9457.77 | 4198.79 | 305 |
| H49A | 4328.86 | 9521.24 | 3659.09 | 252 |
| H49B | 4633.48 | 8865.64 | 3805.43 | 252 |
| H49C | 4454.1 | 9926.78 | 3999.53 | 252 |
| H99A | 3999.74 | 8514.85 | 4088.86 | 134 |
| H99B | 4165.9 | 7452 | 3875.88 | 134 |
| H102 | 5588.87 | 1604.06 | 6959.65 | 145 |
| H103 | 5240.73 | 1458.53 | 7385.25 | 145 |
| H104 | 5421.47 | 770.74 | 7876.06 | 145 |
| H105 | 5950.35 | 228.47 | 7941.26 | 145 |
| H106 | 6298.5 | 373.98 | 7515.65 | 145 |
| H108 | 5527.73 | 6132.24 | 3080.43 | 190 |
| H109 | 5097.22 | 6370.94 | 2742.51 | 190 |
| H110 | 4609.81 | 6991.99 | 2944.51 | 190 |
| H112 | 4552.9 | 7374.32 | 3484.44 | 190 |
| H111 | 4983.41 | 7135.63 | 3822.37 | 190 |

Table 8 Atomic Occupancy for MJP104-3\_150K.

| Atom | *Occupancy* |  | Atom | *Occupancy* |  | Atom | *Occupancy* |
| --- | --- | --- | --- | --- | --- | --- | --- |
| C90 | 0.6 |  | H90A | 0.6 |  | H90B | 0.6 |
| C100 | 0.6 |  | H10A | 0.6 |  | H10B | 0.6 |
| H10C | 0.6 |  | C57 | 0.35 |  | C29 | 0.35 |
| H29 | 0.35 |  | C59 | 0.35 |  | H59 | 0.35 |
| C15 | 0.35 |  | H15 | 0.35 |  | C61 | 0.35 |
| H61 | 0.35 |  | C31 | 0.35 |  | H31 | 0.35 |
| C73 | 0.5 |  | H73A | 0.5 |  | H73B | 0.5 |
| C37 | 0.6 |  | H37A | 0.6 |  | H37B | 0.6 |
| C75 | 0.33 |  | H75A | 0.33 |  | H75B | 0.33 |
| C77 | 0.33 |  | H77A | 0.33 |  | H77B | 0.33 |
| C39 | 0.4 |  | H39A | 0.4 |  | H39B | 0.4 |
| C79 | 0.6 |  | H79A | 0.6 |  | H79B | 0.6 |
| H79C | 0.6 |  | C5 | 0.4 |  | H5A | 0.4 |
| H5B | 0.4 |  | H5C | 0.4 |  | C81 | 0.4 |
| H81A | 0.4 |  | H81B | 0.4 |  | C41 | 0.4 |
| H41A | 0.4 |  | H41B | 0.4 |  | H41C | 0.4 |
| C83 | 0.4 |  | H83A | 0.4 |  | H83B | 0.4 |
| H83C | 0.4 |  | C21 | 0.4 |  | H21A | 0.4 |
| H21B | 0.4 |  | H21C | 0.4 |  | C85 | 0.4 |
| N1 | 0.4 |  | C43 | 0.4 |  | N2 | 0.4 |
| C87 | 0.33 |  | H87A | 0.33 |  | H87B | 0.33 |
| H87C | 0.33 |  | C11 | 0.33 |  | H11A | 0.33 |
| H11B | 0.33 |  | C89 | 0.5 |  | H89A | 0.5 |
| H89B | 0.5 |  | H89C | 0.5 |  | C45 | 0.5 |
| H45A | 0.5 |  | H45B | 0.5 |  | C91 | 0.5 |
| H91A | 0.5 |  | H91B | 0.5 |  | H91C | 0.5 |
| C23 | 0.33 |  | H23A | 0.33 |  | H23B | 0.33 |
| H23C | 0.33 |  | C93 | 0.33 |  | H93A | 0.33 |
| H93B | 0.33 |  | C47 | 0.33 |  | H47A | 0.33 |
| H47B | 0.33 |  | H47C | 0.33 |  | C95 | 0.33 |
| H95A | 0.33 |  | H95B | 0.33 |  | H95C | 0.33 |
| C3 | 0.33 |  | H3A | 0.33 |  | H3B | 0.33 |
| C97 | 0.33 |  | H97A | 0.33 |  | H97B | 0.33 |
| H97C | 0.33 |  | C49 | 0.33 |  | H49A | 0.33 |
| H49B | 0.33 |  | H49C | 0.33 |  | C99 | 0.33 |
| H99A | 0.33 |  | H99B | 0.33 |  | C101 | 0.65 |
| C102 | 0.65 |  | H102 | 0.65 |  | C103 | 0.65 |
| H103 | 0.65 |  | C104 | 0.65 |  | H104 | 0.65 |
| C105 | 0.65 |  | H105 | 0.65 |  | C106 | 0.65 |
| H106 | 0.65 |  |  |  |  |  |

Experimental

Single crystals of C83.56H82.3N0.8Nb8O38
[MJP104-3\_150K]
were
[].
A suitable crystal was selected and
[The crystal was mounted on a MITIGEN holder in perfluoroether oil]
on a
STOE STADIVARI
diffractometer. The crystal was kept at 150 K during data collection.
Using Olex2 [1], the structure was solved with the
SHELXT
[2] structure solution program using
Intrinsic Phasing
and refined with the
SHELXL
[3] refinement package using
Least Squares
minimisation.

1. Dolomanov, O.V., Bourhis, L.J., Gildea, R.J, Howard, J.A.K. & Puschmann, H.
   (2009), J. Appl. Cryst. 42, 339-341.
2. Sheldrick, G.M. (2015). Acta Cryst. A71, 3-8.
3. Sheldrick, G.M. (2015). Acta Cryst. C71, 3-8.

Crystal structure determination of
[MJP104-3\_150K]

**Crystal Data**
for C83.56H82.3N0.8Nb8O38 (*M*=2449.00 g/mol):
monoclinic, space group I2/a (no. 15),
*a* = 42.5266(5) Å, *b* = 10.7503(2) Å, *c* = 42.4305(6) Å, *β* = 89.9780(10)°,
*V*= 19398.1(5) Å3,
*Z* = 8,
*T* = 150 K,
μ(GaKα) = 5.494 mm-1,
*Dcalc* = 1.677 g/cm3,
127791 reflections measured (5.12° ≤ 2Θ ≤ 111.526°),
18778 unique (*R*int = 0.0637, Rsigma = 0.0284) which were used in all calculations.
The final *R*1 was 0.0684
(I > 2σ(I)) and *wR*2 was 0.1991 (all data).

Refinement model description

Number of restraints - 762,
number of constraints - unknown.

Details:

```
1. Fixed Uiso
```

This report has been created with Olex2, compiled on
2023.08.24 svn.re1ec1418 for OlexSys. Please
let us know
if there are any errors or if you would like to have additional features.
